# Integrative bioinformatics and experimental validation unveil CRISP3 as a hypoxia-, epithelial mesenchymal transition-, and immune-related prognostic biomarker and therapeutic target in breast cancer

**DOI:** 10.3389/fimmu.2025.1634399

**Published:** 2025-10-22

**Authors:** Yuanyuan Ren, Yirong Li, Zhen Wang, Yue Cui, Zhiying Xing, Yaning Zhang, Nan Cao, Yi Yu, Yahuan Guo, Xu Li

**Affiliations:** ^1^ Key Laboratory of Resource Biology and Biotechnology in Western China, Ministry of Education, College of Life Sciences, Northwest University, Xi’an, Shaanxi, China; ^2^ School of Medicine, Northwest University, Xi’an, Shaanxi, China; ^3^ Shaanxi Provincial Cancer Hospital, 6th Department of Internal Medicine, Xi’an, Shaanxi, China

**Keywords:** breast cancer, hypoxia, epithelial-mesenchymal transition, prognosis, model, immunotherapy

## Abstract

**Introduction:**

Breast cancer (BC) remains a widespread malignancy and ranks as the second leading cause of cancer-related mortality among women worldwide. Hypoxia, epithelial-mesenchymal transition (EMT), and immune-related processes have been increasingly recognized as critical contributors to BC pathogenesis. However, a prognostic model integrating hypoxia-, EMT-, and immune-related genes (HEMTIRGs) to predict BC outcomes has not yet been established.

**Methods:**

Gene expression datasets of BC patients were obtained from The Cancer Genome Atlas (TCGA) and Gene Expression Omnibus (GEO). Prognostic genes were identified using Least Absolute Shrinkage and Selection Operator (LASSO) Cox regression analysis. A prognostic model was developed based on these genes. Immune infiltration was assessed using CIBERSORT and ssGSEA analyses. Immunotherapy response was predicted using the tumor immune dysfunction and exclusion (TIDE) algorithm. Functional roles of HEMTIRGs in BC malignancy were validated through *in vitro* experiments.

**Results:**

In this study, four HEMTIRGs (PAX7, DCD, CRISP3, and FGG) were identified and used to develop a prognostic model. Patients were stratified into high- and low-risk groups based on median risk scores. A nomogram based on this model accurately predicted overall survival (OS), consistent with the observed outcomes. Notably, patients in the high-risk group exhibited increased immune cell infiltration but a lower predicted response to immunotherapy. Immunohistochemistry (IHC) further confirmed that HEMTIRGs expression levels were strongly associated with breast cancer, with CRISP3 showing the most pronounced upregulation. *In vitro* functional assays demonstrated that CRISP3 promoted malignant phenotypes of breast cancer cells under hypoxic conditions through activation of the IL-17/AKT signaling pathway.

**Conclusion:**

This study establishes a novel HEMTIRGs-based prognostic model for BC, offering a robust tool for predicting patient prognosis and immunotherapy efficacy. Additionally, our findings provide new insights into BC pathogenesis, highlighting potential therapeutic targets.

## 1 Introduction

Breast cancer (BC) remains the most common cancer among women worldwide, accounting for approximately 31% of all female cancers (1). Despite significant advancements in early detection and treatment, BC recurrence remains a major challenge, contributing to increased mortality risk (2). Recent advances in surgical strategies, systemic therapies, and prognostic assessment have improved the management of BC (3–5). However, a major limitation in current clinical practice remains the lack of reliable approaches to accurately predict prognosis, identify high-risk patients, and evaluate responses to immunotherapy. To address this gap, the present study develops a novel prognostic signature by integrating genes associated with hypoxia, epithelial–mesenchymal transition (EMT), and immune regulation.

Hypoxia, characterized by oxygen deprivation, is a common feature of solid tumors and drives cancer progression through enhanced invasion, angiogenesis, metabolic reprogramming, and metastasis (6–9). In BC, hypoxia is associated with increased invasiveness, drug resistance, and poor prognosis (10). Hypoxia-inducible factor 1 (HIF-1), a central regulator of hypoxic responses, is overexpressed in BC and correlates clinically with elevated mortality and metastatic rates (11). Notably, HIF-1α overexpression in BC tumors serves as an independent predictor of poor survival (12).

Notably, hypoxia stress can induce EMT by activating the HIFs signaling pathway, thereby enhancing tumor cell migration, invasion, and adaptation to the tumor microenvironment (13–16). EMT facilitates tumor cell dissemination to distant organs to establish secondary tumors, ultimately driving metastasis (17). In BC, EMT activation is prominent in HER2-positive breast cancer stem cells, which exhibit radioresistance, drug resistance, and poor clinical outcomes, while EMT inhibition may mitigate treatment resistance and metastasis by suppressing HER2 expression (18). Moreover, hypoxia upregulates Slug and Snail, which suppress E-cadherin expression, a key hallmark of EMT, further reinforcing BC invasiveness (15, 19).

Beyond hypoxia and EMT, immune-related mechanisms also play a crucial role in BC progression, including immune evasion, chronic inflammation, and immune cell infiltration (20). In triple-negative breast cancer (TNBC), hypoxia disrupts immune function by engaging HIF-1α with histone deacetylase 1 (HDAC1) and polycomb repressive complex 2 (PRC2), leading to suppression of T and NK cell activity (21). Furthermore, studies have shown that HIF-1α overexpression in T cells promotes T cell exhaustion and weakens antitumor immunity in metastasis models (22). Additionally, EMT has been implicated in immune evasion, as it upregulates immune checkpoints, enhances metastatic potential, and contributes to therapy resistance (23).

Together, hypoxia, EMT, and immune suppression are interconnected processes that form a synergistic network, which cooperatively modulates BC malignancy. Although several prognostic models have incorporated hypoxia- and immunity-related factors, particularly in TNBC, offering improved stratification for targeted therapies (24–26), the accuracy in patient risk stratification remains limited, and the underlying mechanistic insights remain incomplete. Currently, no prognostic model has yet integrated hypoxia-, EMT-, and immune-related genes (HEMTIRGs) into a unified framework. Given the established biological crosstalk among these processes, we aimed to innovatively integrate them into a single model to capture a more holistic view of BC, thereby improving the precision of prognostic stratification and informing potential therapeutic strategies.

In this study, we identified four key HEMTIRGs through a comprehensive bioinformatics analysis. A prognostic model based on this HEMTIRGs signature in a BC patient cohort was developed and validated. We also constructed a nomogram to predict overall survival (OS). Additionally, an *in vitro* experiment verified the biological functions of HEMTIRGs in promoting BC malignancy. Our findings offer novel insights into the molecular mechanisms underlying BC pathogenesis associated with hypoxia, EMT, and immune responses, with potential implications for clinical prognosis, risk stratification, and immunotherapy response assessment.

## 2 Materials and methods

### 2.1 Acquisition of hypoxia-, EMT- and immune-related gene sets

A total of 200 hypoxia-related genes were obtained from the Molecular Signatures Database (http://www.gsea-msigdb.org/gsea/msigdb/index.jsp) (**Supplementary Table S1**). EMT-related genes were retrieved from the dbEMT 2.0 database (https://bioinfo-minzhao.org/dbemt/dbemt1/index.html) (**Supplementary Table S2**). Additionally, the immune-related gene set was obtained from the GeneCards database (https://www.genecards.org/) using the search term “Immune” with a relevance score>2 (**Supplementary Table S3**).

### 2.2 Data collection

RNA sequencing data and clinical information for The Cancer Genome Atlas Breast Invasive Carcinoma (TCGA-BRCA) cohort were collected from the TCGA database (https://portal.gdc.cancer.gov/). Samples without detailed expression and clinical data or with a follow-up duration of 0 days were excluded, resulting in 1,037 BRCA and 99 normal samples for training (27). For model validation, the GSE20685 BRCA cohort was accessed from the Gene Expression Omnibus (GEO) database (https://www.ncbi.nlm.nih.gov/geo/). Expression profiles were log2-transformed and normalized, with the average expression level used for duplicate genes. Batch effects were corrected using the “ComBat” function from the “sva” package (https://bioconductor.org/packages/release/bioc/html/sva.html) (v3.50.0) in R software version 4.2.1 (28).

### 2.3 Consensus clustering analysis

To identify potential molecular subtypes of BC based on HEMTIRGs, we applied the “ConsensusClusterPlus” package (v1.62.0) in R (v4.2.1) to perform consensus clustering on tumor samples (29). The partitioning around medoids (PAM) algorithm was selected with Euclidean distance as the similarity metric. During clustering, 80% of samples were resampled, and the process was repeated 1,000 times to ensure stability and reproducibility. The optimal number of clusters (K) was determined by evaluating the cumulative distribution function (CDF) curve, the relative change in the area under the CDF curve, and the consensus heatmap. According to these criteria, K = 4 was identified as the most robust and biologically meaningful classification, and all tumor samples were subsequently stratified into four molecular subtypes (C1–C4). A random seed (set.seed = 12345) was applied to guarantee reproducibility.

### 2.4 Analysis of hypoxia and EMT features in BC subtypes

Following the determination of the optimal cluster number, we analyzed hypoxia- and EMT-related gene expression profiles within each identified subtype. Gene Set Variation Analysis (GSVA, v1.50.5) was employed to perform single-sample gene set enrichment analysis (ssGSEA), assigning hypoxia and EMT scores to each tumor sample based on predefined gene sets (30). Patients were then stratified into high- and low-hypoxia/EMT subgroups using the median score within each subtype.

### 2.5 Identification of differentially expressed genes

The “edgeR” package (v4.0.16) was used to analyze differentially expressed genes (DEGs) between high and low hypoxia/EMT subgroups, as well as between TCGA BRCA and normal tissues (27). Genes were classified as differentially expressed based on an adjusted p-value (adj.p)< 0.05 and |log2FoldChange| > 1.

### 2.6 Identification and validation of the prognostic HEMTIRGs signature

To identify genes significantly associated with overall survival (OS), univariate Cox proportional hazards regression analysis was performed for each HEMTIRG in the TCGA training cohort (28, 31). Subsequently, LASSO Cox regression analysis was applied using the “glmnet” package (version 4.1-8) to refine the selection of prognostic HEMTIRGs (32, 33). The optimal lambda value was determined, and the corresponding HEMTIRGs were identified. A prognostic risk score was then calculated for each patient using the following formula:


$$Risk\;Score=\sum expr_{i}*coef_{i}$$

where “coef” represents the regression coefficient for each HEMTIRG, and “expr” denotes its respective expression level. Patients in the TCGA BRCA cohort were stratified into high-risk and low-risk groups based on the median risk score. To evaluate whether the HEMTIRGs-based prognostic model functions as an independent prognostic factor, univariate and multivariate Cox proportional hazards regression analyses were performed in conjunction with clinical variables. Kaplan-Meier (K-M) survival curves were constructed to compare survival outcomes between risk groups, with statistical differences assessed using the log-rank test. The prognostic accuracy of the model was further evaluated using ROC curve analysis, with results visualized *via* the “timeROC” package (v0.4) (34). Model discrimination was quantified by the area under the curve (AUC).

### 2.7 Development and validation of the nomogram

A prognostic nomogram was developed using the “rms” package (v6.8.0) in R to estimate 1-, 3-, and 5-year overall survival (OS) probabilities for BC patients. The predictive accuracy of the nomogram was assessed using calibration curves and the Concordance Index (C-Index). Calibration curves visually compared the predicted survival probabilities with actual outcomes, while the C-Index provided a quantitative measure of model performance, with the AUC indicating its discriminatory ability.

### 2.8 Drug sensitivity profiling and analysis

Drug sensitivity prediction was conducted using the calcPhenotype function in the OncoPredict package40. Training data were obtained from two pharmacogenomic resources: GDSC241, used to evaluate the sensitivity of breast cancer samples to commonly applied chemotherapeutics, and CTRP, applied to assess the sensitivity of EMT-promoting compounds (35, 36). For all compounds tested, lower predicted scores represent higher sensitivity of the samples.

### 2.9 Gene set enrichment analysis

To explore biological pathways associated with high- and low-risk BC patients, GSEA was performed using the “clusterProfiler” package (v4.10.1) (37, 38). Genes were ranked based on their signal-to-noise ratio, and enrichment analysis was conducted using the KEGG pathway database (c2.cp.kegg.v7.5.1.entrez.gmt). Pathways with a normalized enrichment score (|NES|) > 1 and p-value< 0.05 were considered significantly enriched.

### 2.10 Functional enrichment analysis

To explore the biological significance of HEMTIRGs, we conducted functional enrichment analysis using the “clusterProfiler” package (v4.10.1) in R (37). This included Gene Ontology (GO) and Kyoto Encyclopedia of Genes and Genomes (KEGG) pathway enrichment to characterize the functional profiles of HEMTIRGs. An enrichment p-value of less than 0.05 was considered statistically significant (39, 40).

### 2.11 Immune landscape analysis

To analyze the immune landscape of BC, tumor gene expression profiles were examined to estimate the proportions of immune and stromal cells within the tumor microenvironment (TME). CIBERSORT was employed to deconvolute immune cell composition, identifying the relative abundances of 22 distinct immune cell types (41). Additionally, single-sample gene set enrichment analysis (ssGSEA) from the “GSVA” package (v1.50.5) was used to estimate the infiltration levels of 28 different immune cell types (42).

### 2.12 Immunotherapy responses analysis

To evaluate the potential of the HEMTIRGs-based model in predicting immunotherapy response, the Tumor Immune Dysfunction and Exclusion (TIDE) algorithm was applied. TIDE is a computational tool that assesses tumor immune evasion and predicts responses to immune checkpoint inhibitors (ICIs) based on multiple biomarker signatures. TIDE scores were obtained from the Harvard TIDE database (http://tide.dfci.harvard.edu/) (43).

### 2.13 Immunohistochemical staining analysis of HEMTIRGs protein in BC samples

To examine protein expression levels of HEMTIRGs in BC tissues, immunohistochemical (IHC) staining images were retrieved from the Human Protein Atlas (HPA) database (https://www.proteinatlas.org/).

### 2.14 Reagents

Reagents were purchased or obtained from the following sources: rabbit anti-E-Cadherin antibody (A20798, ABclonal, China), rabbit anti-Vimentin antibody (A2584, ABclonal, China), rabbit anti-Snail antibody (A5243, ABclonal, China), rabbit anti-phospho-AKT (Ser 473) (#4060s, CST, USA), mouse anti-AKT antibody (60203-2-Ig, Proteintech Wuhan, China), mouse anti-Beta Actin antibody (66009-1-Ig, Proteintech Wuhan, China), NeutraKine^®^ IL-17A Mouse McAb (69021-1-Ig, Proteintech Wuhan, China), and goat anti-rabbit IgG (H+L) secondary antibody Alexa Fluor^®^ 488 conjugate (ABclonal, China). Plasmids (PSPAX2, PMD2.G) used in this experiment were purchased from Vector Builder (China). Cell Counting Kit-8 assay was purchased from Beyotime (Cat No.C0038, Beyotime, China).

### 2.15 Cell culture

The MDA-MB-231 and MDA-MB-468 triple-negative breast cancer (TNBC) cell lines were purchased from ATCC (American Type Culture Collection). Cells were cultured in Dulbecco’s Modified Eagle Medium (DMEM) (Sigma, USA) supplemented with 10% fetal bovine serum (FBS) (Sigma, USA), 100 U/mL penicillin, and 100 μg/mL streptomycin. Cultures were maintained in a humidified incubator at 37°C with 5% CO_2_.

For hypoxia treatment, cells were incubated in a controlled hypoxic chamber with 1% O_2_, 5% CO_2_, and 94% N_2_, while normoxic conditions were maintained at 21% O_2_ and 5% CO_2_. IL-17 neutralizing antibody (nAb) treatment was administered at a final concentration of 10 μg/mL.

### 2.16 RNA extraction and quantitative real-time polymerase chain reaction

Total RNA was extracted using Trizol (ThermoFisher, Waltham, MA, USA) according to the manufacturer’s instructions, and 1 μg of total RNA from each cell line was used to transcribe into cDNA using TransScript^®^ One-Step gDNA Removal and cDNA Synthesis SuperMix (Beijing Quanshijin Biotechnology Co., Ltd., Beijing, China). Quantitative real-time PCR (qRT-PCR) was performed using the PerfectStart Green qPCR SuperMix on the Bio-Rad CFX96 RealTime PCR system (Bio-Rad, US) with the program of 94°C for 30 s, 45 cycles of 94°C for 5 s and 60°C for 30 s. The relative gene expression levels were calculated using the 2-^ΔΔ^Ct method with β-actin as an internal control. The primer sequences used in this experiment were listed in **Table 1**.

**Table 1:** List of primers used for qRT-PCR (h: human).

Target gene	Forward	Reverse
hHIF1A	TATGAGCCAGAAGAACTTTTAGGC	CACCTCTTTTGGCAAGCATCCTG
hCRISP3	TGTCAAGTGCCTCCAGCTCATG	CACATCCAACGAGGTATGAAGAG
hPAX7	GGAGGATGAAGCGGACAAGAAG	AGGTCAGGTTCCGACTCCACAT
hFGG	GAAGGCAACTGTGCTGAACAGG	CCATTAGGAGTAGATGCTTTTGAG
hDCD	GGTTAGCCAGACAGGCACCAAA	CACGCTTTCTAGATCTTCGACTG
hβ-actin	CACCATTGGCAATGAGCGGTTC	AGGTCTTTGCGGATGTCCACGT

### 2.17 Colony formation assay

Approximately 500 cells per group were seeded in six-well plates in triplicate and incubated at 37°C with 5% CO_2_ for about two weeks, until visible cell colonies were observed under a microscope. The colonies were then fixed with 4% paraformaldehyde for 20 minutes, washed twice with PBS, and stained with 0.2% crystal violet solution (Sigma, St. Louis, MO, USA) for 10 minutes. After staining, the plates were washed three times with PBS, air-dried, photographed, and quantified using ImageJ software.

### 2.18 Cell counting kit 8 assay

Cell proliferation was assessed using the Cell Counting Kit-8 (CCK-8). Cells from each group were seeded in 96-well plates at a density of 5 × 10³ cells per well and incubated at 37°C with 5% CO_2_ for 24, 48, 72, and 96 hours. At each time point, 10 μL of CCK-8 solution was added to each well, followed by incubation for 2 hours in the dark. The absorbance at 450 nm was measured using a microplate reader (Bio-Rad Laboratories, Hercules, CA, USA).

### 2.19 Wound healing assay

A scratch wound assay was performed to assess the migration ability of BC cells *in vitro*. Horizontal guidelines were drawn on the back of a six-well plate using a marker, and cells were seeded into the plate for culture. Once the cells reached ~80% confluence, a sterile 200 μL pipette tip was used to create a vertical scratch across the monolayer, perpendicular to the horizontal guidelines. The cells were then washed with PBS to remove debris, and the complete medium was replaced with DMEM containing 0.2% FBS. The cells were incubated at 37°C with 5% CO_2_ for 24 hours. Images of the wound area were captured at 0 hours and 24 hours using a light microscope (Olympus Corp., Tokyo, Japan), and the scratch area was quantified using ImageJ.

### 2.20 Transwell assay

To evaluate cell invasion, 100 μL of diluted Matrigel was added to the upper chamber of the transwell insert and incubated at 37°C with 5% CO_2_ for 2–4 hours to allow the gel to solidify. Subsequently, 200 μL of serum-free medium containing 5 × 10^4^ cells was added to the upper chamber, while 600 μL of complete medium containing 20% FBS was placed in the lower chamber. The transwell plates were incubated at 37°C with 5% CO_2_ for 24 hours. After incubation, the transwell chambers were washed three times with PBS, and non-invading cells in the upper chamber were removed with a cotton swab. The membranes were then fixed with 4% methanol for 20 minutes, washed three times with PBS, stained with 0.25% crystal violet for 30 minutes, washed again three times with PBS, air-dried, and photographed for analysis.

### 2.21 Enzyme‐linked immunosorbent assay

The concentration of IL-17 in the culture supernatant was measured using an ELISA kit (RK00397, ABclonal, China), following the manufacturer’s instructions. Blank control wells were included in the assay. The absorbance at 450 nm was measured using a microplate reader, and the IL-17 concentration was determined based on a standard curve.

### 2.22 Lentivirus generation and transduction

HEK-293T cells were co-transfected with the packaging plasmid (psPAX2), envelope plasmid (pMD2.G), and the PLKO.1-TRC plasmid with targeted shRNA sequences for knockdown using Lipo6000™ Transfection Reagent to produce the corresponding lentivirus. After culturing the transfected HEK-293T cells for 48 h, the supernatant was removed and centrifuged for 5 min at 1000 rpm to extract virus particles. Subsequently, lentivirus-infected cells were screened for cells expressing the relevant antibiotic resistance gene in a growth medium supplemented with 2 μg/mL puromycin. To interfere with CRISP3 expression, short hairpin RNA (shRNA) oligos of CRISP3 were cloned into pLKO.1-TRC. The boldface sequences below represent the targeting sequences for hCRISP3-1-shRNA and hCRISP3-2-shRNA (only the sense strand is shown):

hCRISP3-1-shRNA-F:

5’- CCGG**CAGTAACCCAAAGGATCGAAT**CTCGAGATTCGATCCTTTGGGTTACTGTTTTTG-3’

hCRISP3-2-shRNA -F:

5’- CCGG**GTGCAATTACAGACACAGTAA**CTCGAGTTACTGTGTCTGTAATTGCACTTTTTG-3’

### 2.23 Statistical analysis

Statistical significance between two sets of data was assessed using the Student’s t-test, while comparisons among more than two groups were evaluated through analysis of one-way ANOVA. Univariate Cox analysis was employed to identify genes with prognostic significance. Kaplan-Meier (K-M) survival curves were constructed and compared using the log-rank test. All statistical analyses were performed using R version 4.2.1 (https://www.r-project.org/) along with appropriate packages. Statistically significant differences are indicated by asterisks (*p< 0.05; **p< 0.01; ***p< 0.001). All experimental data are presented as mean ± SEM of four independent replicates.

## 3 Results

### 3.1 Identification of HEMTIRGs in the BRCA cohort

#### 3.1.1 Consensus clustering and subgroup classification

**Figure 1:**
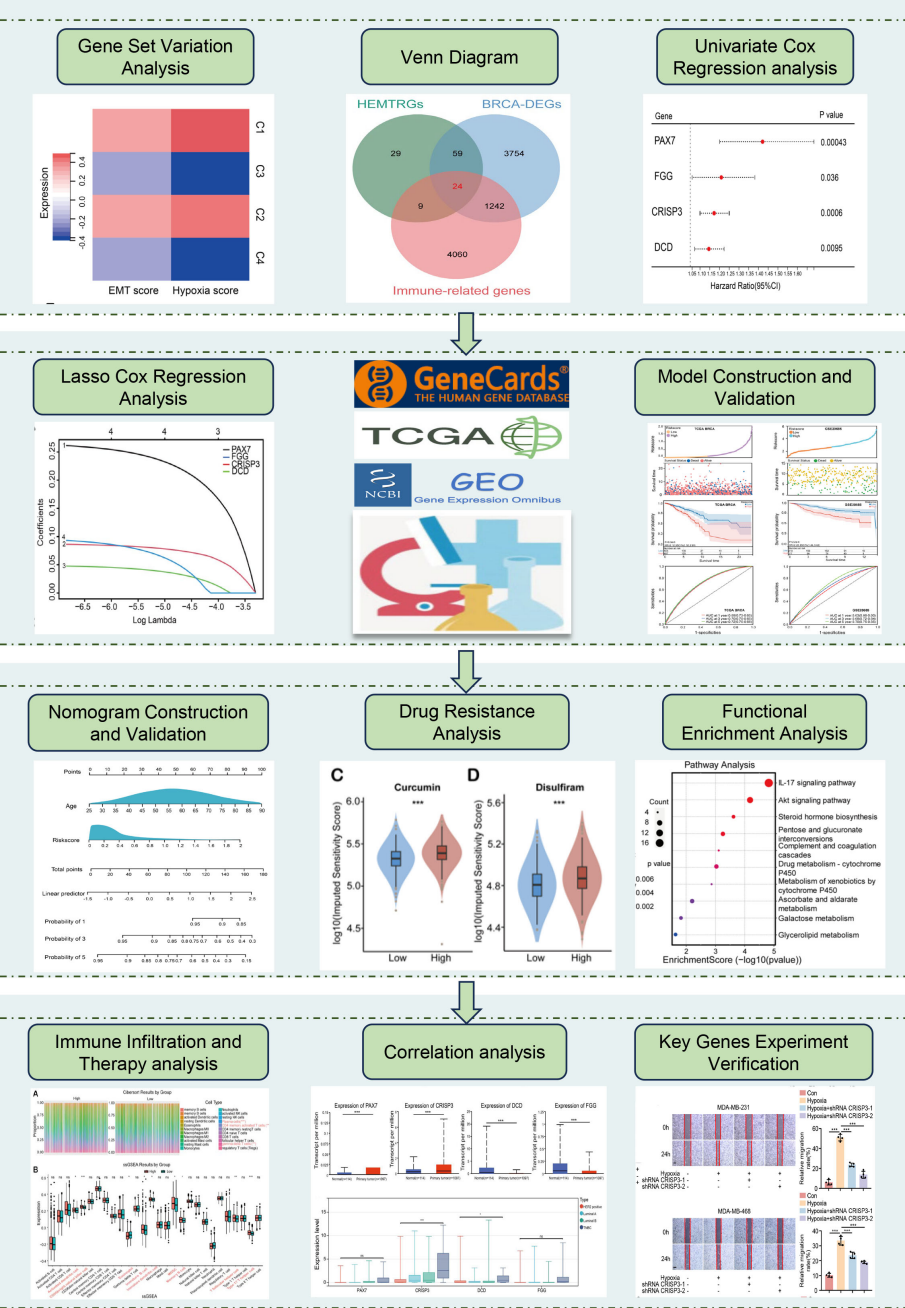
The flowchart of this study.

**Figure 2:**
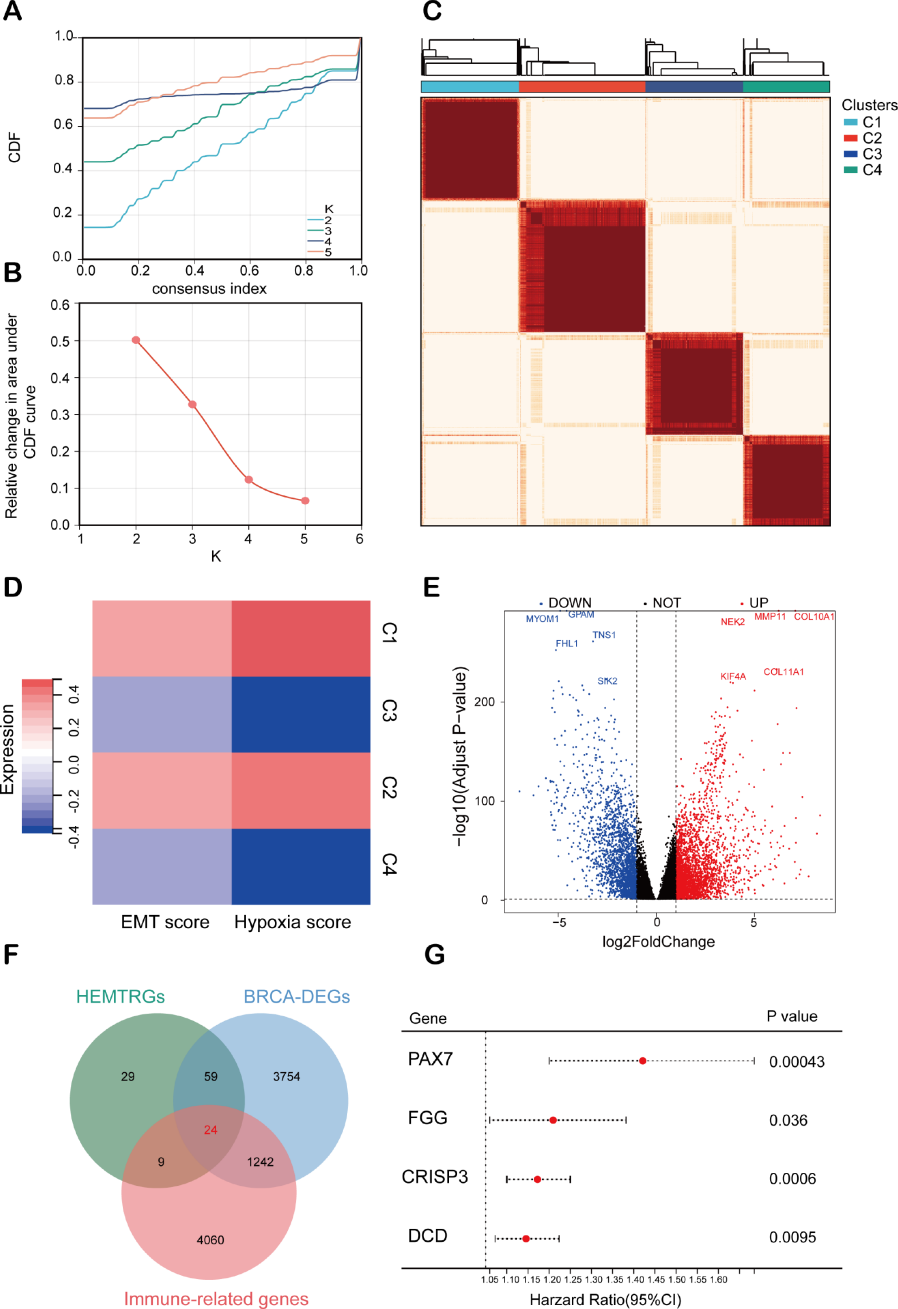
Consensus clustering analysis for identifying HEMTIRGs. **(A)** Consensus clustering CDF for k = 2-5. **(B)** Relative change in area under the CDF curve for k = 2-5. **(C)** Consensus clustering matrix for k = 4. **(D)** Heatmap of hypoxia scores and EMT scores for the 4 subgroups. **(E)** Volcano plot of the 5078 DEGs. **(F)** The Venn diagram of analysis of HEMTRGs, BRCA-DEGs, and immune-related genes. **(G)** Forest plot of the univariate Cox regression analysis.

The study design is illustrated in **Figure 1**. To identify hypoxia- and EMT-related genes in BC, consensus clustering was performed on RNA sequencing data from 1,037 BC cases in the TCGA database (**Supplementary Table S4**). **Supplementary Figures S1A–D** display the Kaplan-Meier survival curves and log-rank tests for various clinicopathological parameters, including overall stage, tumor (T), metastasis (M), and node (N) classifications. The cumulative distribution curve (**Figure 2A**) and area under the distribution curve (**Figure 2B**) indicated that the highest group consistency was achieved at k=4. Accordingly, the 1,037 BRCA samples were classified into four distinct subtypes (C1, C2, C3, and C4), as confirmed by the consensus matrix (**Figure 2C**). Subsequently, gene set variation analysis (GSVA) was conducted to assess hypoxia and EMT scores across the four subtypes. The results showed that C1 and C2 exhibited significantly higher hypoxia and EMT scores than C3 and C4 (**Figure 2D**). Based on this, C1 and C2 were designated as the high hypoxia/EMT group, while C3 and C4 were categorized as the low hypoxia/EMT group. This classification led to the identification of 121 differentially expressed genes (DEGs) associated with hypoxia and EMT (**Supplementary Table S5**).

#### 3.1.2 Identification of HEMTIRGs and prognostic risk genes

Additionally, analysis of the TCGA cohort identified 5,079 BC-specific DEGs (BRCA-DEGs), with 2,940 upregulated and 2,138 downregulated genes, as shown in the volcano plot (**Figure 2E**). By intersecting hypoxia- and EMT-related DEGs (**Supplementary Table S5**), BRCA-DEGs (**Supplementary Table S6**), and 5,335 immune-related genes (**Supplementary Table S3**), we identified 24 differentially expressed genes associated with hypoxia, EMT, and immune function (HEMTIRGs) (**Figure 2F**). Univariate Cox regression analysis was then conducted to evaluate the prognostic significance of the 24 HEMTIRGs in overall survival (OS). Among these, PAX7 (paired box 7), FGG (fibrinogen gamma chain), CRISP3 (cysteine-rich secretory protein 3), and DCD (dermcidin) were significantly correlated with OS and identified as risk genes (HR > 1, p< 0.05) (**Figure 2G**). Further investigation focused on the copy number variations (CNVs) of HEMTIRGs, aiming to explore potential associations between CNVs and mRNA expression levels in TCGA BRCA samples. The expected copy number for each gene is 2; values above 2 are categorized as “GAIN” and those below 2 as “LOSS.” We observed a significant amplification of DCD copy numbers, which was associated with an increase in mRNA expression (**Supplementary Figure S2A**). In contrast, PAX7 and FGG showed relatively low CNV frequencies, and no CNVs were detected for CRISP3 (**Supplementary Figure S2A**).

### 3.2 Development and evaluation of a prognostic model based on HEMTIRGs in the TCGA cohort and the GEO cohort

#### 3.2.1 Construction and validation of the HEMTIRGs-based prognostic model

**Figure 3:**
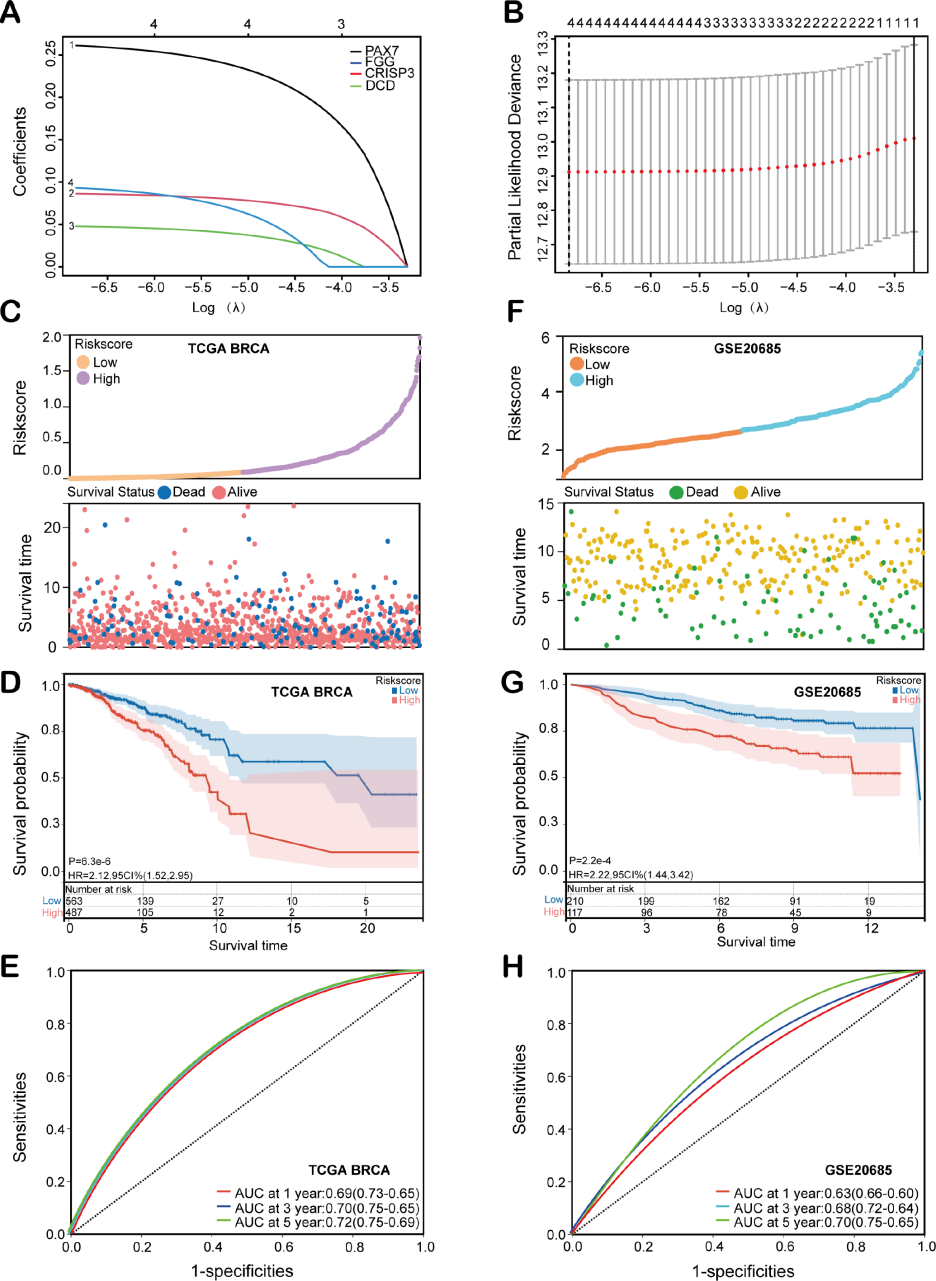
Construction and evaluation of the HEMTIRGs-based prognostic model. **(A)** LASSO coefficient profiles of the four HEMTIRGs. **(B)** Coefficient profile plot generated against the log (λ) sequence in the LASSO model, with the optimal λ value indicated by a vertical black dotted line. **(C)** Distribution of risk score (top), and survival status with survival time (bottom) in the TCGA BRCA training cohort. Patients were stratified into high-risk (purple) and low-risk (yellow) groups according to the median risk score, while survival status is indicated as alive (red) or dead (blue). **(D)** Kaplan-Meier curves of HEMTIRGs in the TCGA BRCA training cohort. **(E)** The time-dependent ROC curves of the HEMTIRGs in the TCGA BRCA training cohort. **(F)** The distribution of the risk score (top), and survival status with survival time (bottom) in the GSE20685 validation cohort. Patients were stratified into high-risk (orange) and low-risk (blue) groups according to the median risk score, while survival status is indicated as alive (yellow) or dead (green). **(G)** Kaplan-Meier curves of the HEMTIRGs in the GSE20685 validation cohort. **(H)** The time-dependent ROC curves of the HEMTIRGs in the GSE20685 validation cohort.

To evaluate the prognostic value of the identified HEMTIRGs in the BC, we conducted LASSO Cox regression analysis, which identified PAX7, FGG, CRISP3, and DCD as key prognostic genes with optimal logarithmic lambda values (λ = 0.001) (**Figures 3A, B**). A risk score for each patient in the TCGA cohort was then computed using the following formula: Risk score = 0.26 * PAX7 + 0.093 * FGG + 0.086 * CRISP3 + 0.048 * DCD. The TCGA training cohort was then stratified into low-risk (n = 519) and high-risk (n = 518) groups based on the median risk score. High-risk patients exhibited higher risk scores and shorter survival times compared to low-risk patients (**Figure 3C**). Kaplan-Meier survival analysis further demonstrated a significantly higher survival probability for the low-risk group compared to the high-risk group (p< 0.001, HR = 2.21, 95% CI: 1.52-2.95) (**Figure 3D**). Receiver operating characteristic (ROC) analysis revealed 1-, 3-, and 5-year survival probabilities of 0.69, 0.70, and 0.72, respectively (**Figure 3E**). The prognostic accuracy of the model was validated in the GEO cohort (GSE20685), which showed consistent results with the TCGA training cohort (**Figures 3F–H**). Several prognostic models have been previously proposed for predicting survival outcomes in BC patients.

#### 3.2.2 Evaluation of model performance relative to established prognostic models

We next conducted a comparative analysis of our model against three previously established models: Model 1 (Lu et al.) (44), Model 2 (Liu et al.) (45), and Model 3 (Gong et al.) (46), using both the concordance index (C-index) and decision curve analysis (DCA) (**Supplementary Figures S3A, B**). The C-index comparison revealed that the HEMTIRGs-model exhibited the optimal prediction ability for overall survival (OS) probabilities, achieving the highest C-index value (0.712) compared to the other three models (**Supplementary Figure S3A**). Furthermore, DCA results demonstrated that our model provided superior clinical performance, offering greater net clinical benefit compared to the other models (**Supplementary Figure S3B**). These findings suggest that the HEMTIRGs-based prognostic model demonstrates exceptional accuracy, reliability, and performance in clinically predicting the OS of BC patients.

### 3.3 Construction and validation of the nomogram

**Figure 4:**
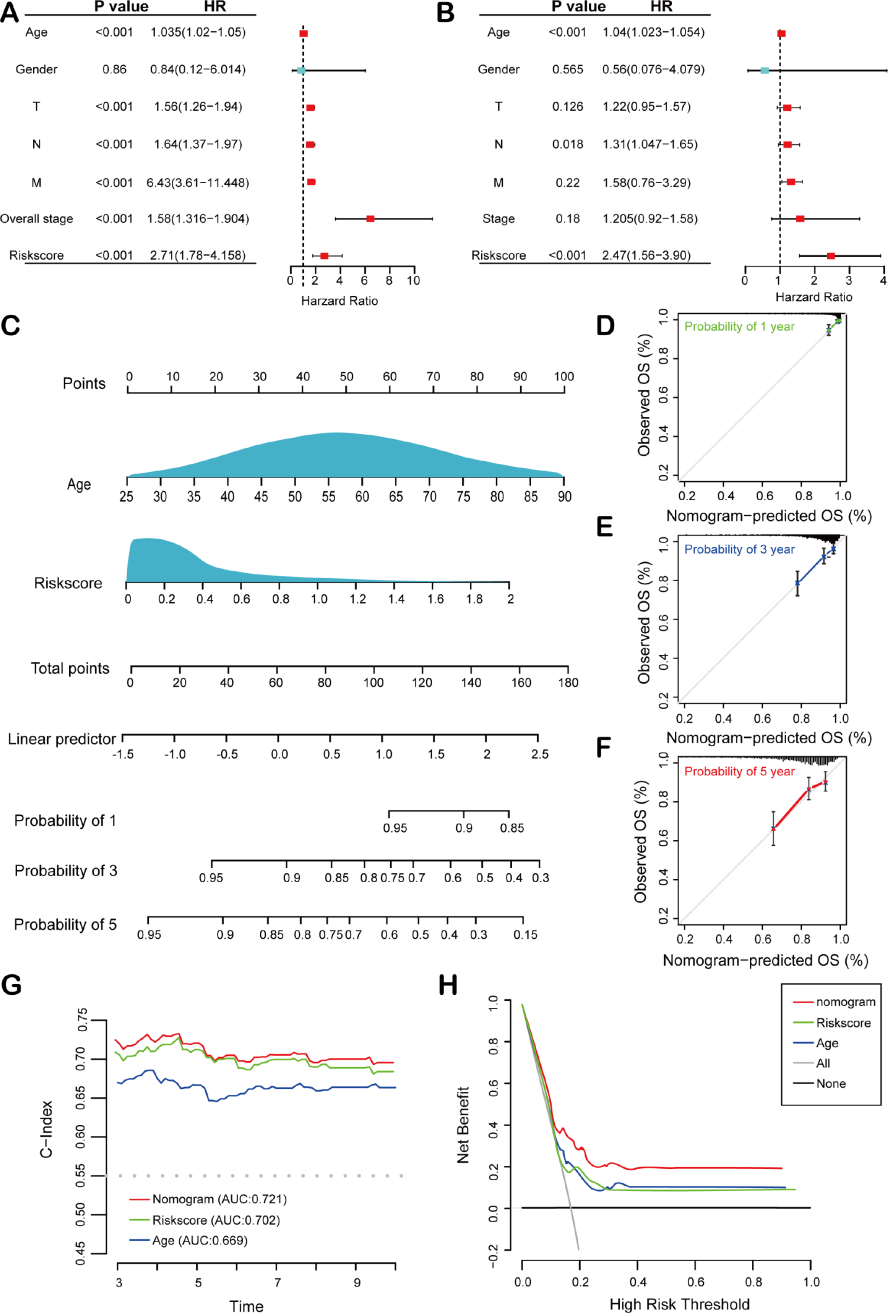
Construction and validation of the nomogram. Forest plots of the **(A)** univariate and **(B)** multivariate Cox regression analysis in the TCGA BRCA cohort. **(C)** The nomogram was developed based on age and risk score. The calibration plots were generated to predict the **(D)** 1-year, **(E)** 3-year, and **(F)** 5-year OS rates. The X-axis displays the nomogram-predicted survival, and the y-axis represents the actual survival. **(G)** The concordance index curves of the nomogram, risk score, and age. **(H)** DCA curves for nomogram, risk score, and age.

To assess the independent predictive ability of the risk score, we performed both univariate and multivariate Cox regression analyses. Univariate Cox regression analysis revealed significant associations between overall survival (OS) and multiple clinicopathological variables, including age (p< 0.001, HR = 1.035, 95% CI = 1.021-1.050), T stage (p< 0.001, HR = 1.56, 95% CI = 1.26-1.94), N stage (p< 0.001, HR = 1.64, 95% CI = 1.37-1.97), M stage (p< 0.001, HR = 6.43, 95% CI = 3.61-11.45), tumor stage (p< 0.001, HR = 1.58, 95% CI = 1.32-1.90), and risk score (p< 0.001, HR = 2.71, 95% CI = 1.78-4.16) (**Figure 4A**). Furthermore, multivariate Cox regression analysis confirmed that both age (p< 0.001, HR = 1.039, 95% CI = 1.02-1.05) and risk score (p< 0.001, HR = 2.47, 95% CI = 1.56-3.90) were significant independent prognostic factors for OS in BC patients within the TCGA training cohort (**Figure 4B**). Next, a HEMTIRGs-nomogram was developed to predict 1-, 3-, and 5-year OS in the TCGA BRCA cohort, incorporating two independent risk factors (age and risk score) (**Figure 4C**). Calibration curves demonstrated that the predicted survival rates of the nomogram were consistent with the observed survival rates at 1, 3, and 5 years (**Figures 4D–F**). To assess the clinical decision value of the nomogram compared to other clinical indicators, we calculated the concordance index (C-index) and decision curve analysis (DCA). The nomogram achieved a C-index of 0.721, outperforming risk score (AUC = 0.702) and age (AUC = 0.669) in predicting OS (**Figure 4G**). Furthermore, DCA revealed that the nomogram yielded higher net benefits than other indicators in clinical practice (**Figure 4H**). These results demonstrate that the nomogram based on HEMTIRGs provides significantly higher predictive accuracy than individual clinical indicators, underscoring its potential as a valuable tool for clinical decision-making.

### 3.4 Clinicopathological analysis of HEMTIRGs in BC

**Figure 5:**
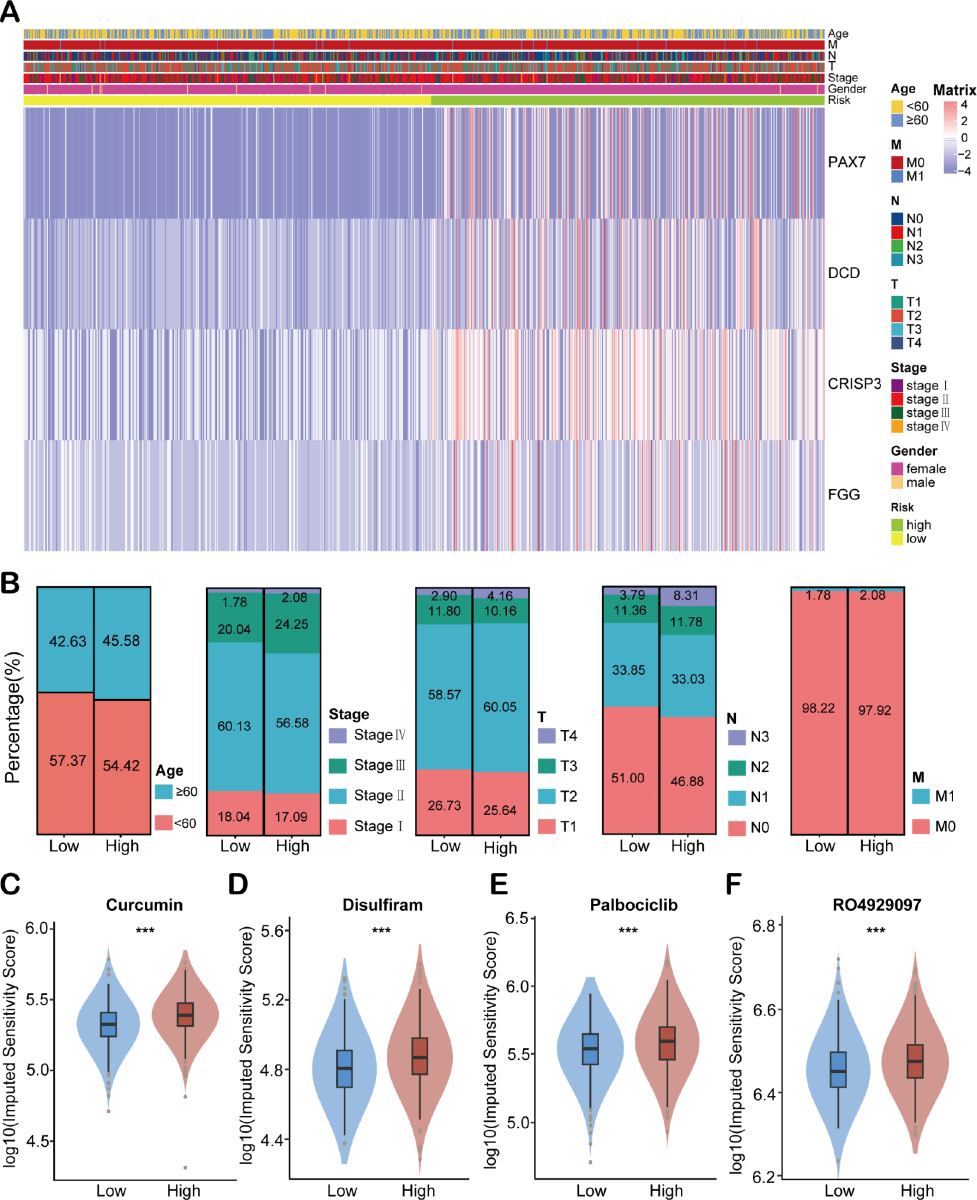
Clinical correlation analysis of HEMTIRGs. **(A)** The heatmap of HEMTIRGs expression associated with age, T, N, M, stage, gender, and risk scores. **(B)** Distribution of clinicopathological characteristics (age, overall stage, T stage, N stage, and M stage) between high- and low-risk groups. **(C–F)** Drug sensitivity analysis between high- and low-risk groups for EMT inhibitors, including **(C)** curcumin, **(D)** disulfiram, **(E)** palbociclib, and **(F)** RO4929097. Statistical significance was determined by an unpaired Student’s t-test. ***p < 0.001.

To investigate the association between HEMTIRGs expression and clinicopathological characteristics in BC patients, we generated a heatmap illustrating gene expression patterns (**Figure 5A**). The heatmap demonstrated that the high-risk group exhibited a significantly higher proportion of patients with advanced age (≥60 years), advanced stage (stage IV), and advanced TNM grade (T4, N3, and M1) (**Figure 5B**). Furthermore, differential drug sensitivity analysis revealed that patients in the low-risk group exhibited significantly increased sensitivity to several commonly used EMT-targeting inhibitors, such as curcumin (47), disulfiram (48), palbociclib (49), and RO4929097 (50) as compared to high-risk patients (**Figures 5C–F**). These findings suggest that EMT inhibition may represent a promising therapeutic strategy, particularly for low-risk patients who demonstrate greater responsiveness to such agents.

### 3.5 Exploration of molecular functions and signaling pathways associated with HEMTIRGs using GSEA, GO, and KEGG analyses

**Figure 6:**
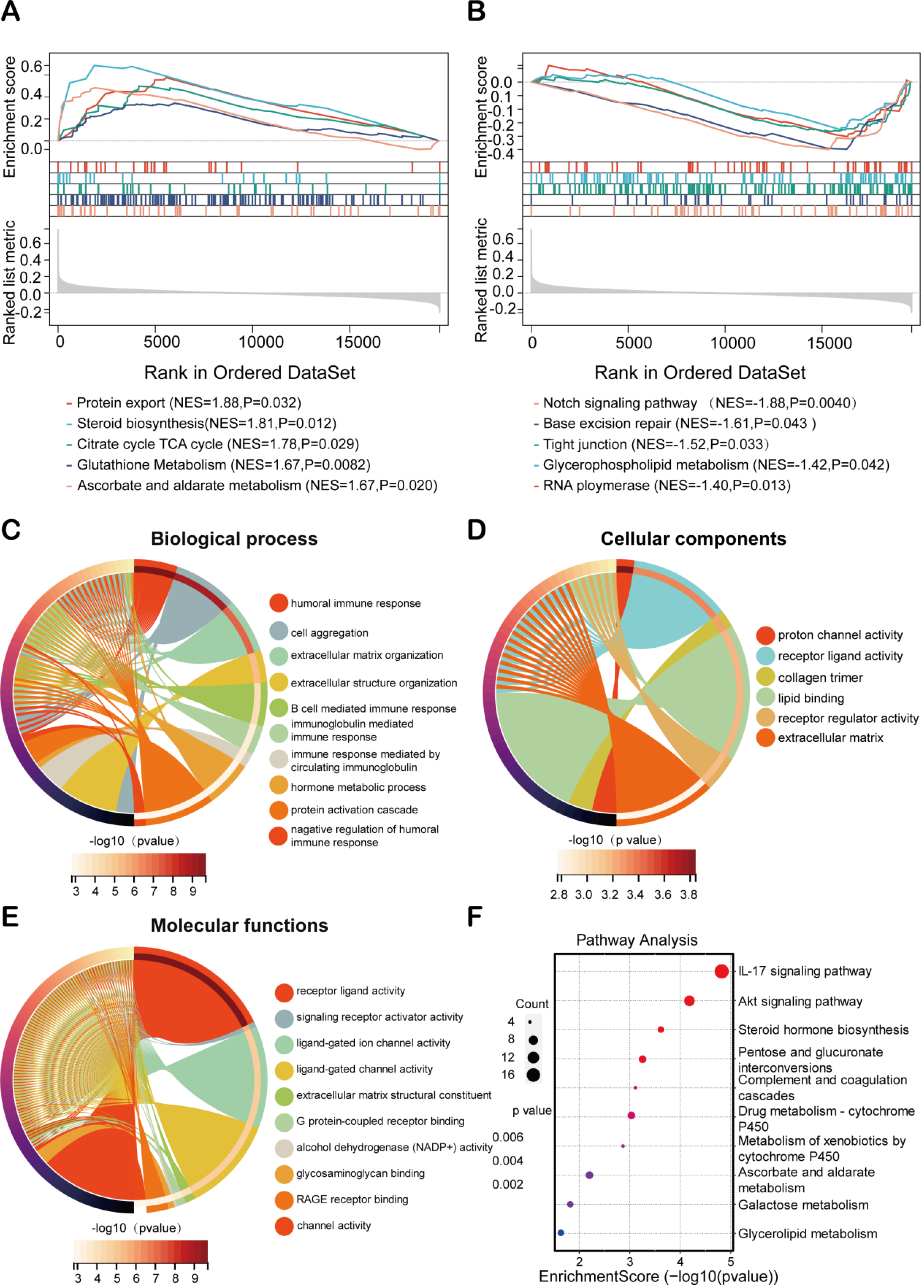
Exploration on molecular functions and signaling pathways of HEMTIRGs. Enrichment plot of the DEGs between the **(A)** high- and **(B)** low-risk groups using GSEA (Gene Set Enrichment Analysis). **(C–E)** GO (Gene Ontology) analysis, including **(C)** BP (Biological Processes), **(D)** CC (Cellular Components), and **(E)** MF (Molecular Functions). **(F)** KEGG (Kyoto Encyclopedia of Genes and Genomes) analysis.

To elucidate the biological functions associated with HEMTIRGs in high-risk and low-risk groups, we performed Gene Set Enrichment Analysis (GSEA). All the enriched KEGG pathways were listed in **Supplementary Table S7**. GSEA results identified 30 significantly enriched pathways in the high-risk group, including protein export (NES = 1.88, p = 0.032), steroid biosynthesis (NES = 1.81, p = 0.012), citrate cycle (TCA cycle) (NES = 1.78, p = 0.029), glutathione metabolism (NES = 1.67, p = 0.0082), and ascorbate and aldarate metabolism (NES = 1.67, p = 0.020), all of which are implicated in tumorigenesis (**Figure 6A**). In the low-risk group, 15 pathways were significantly enriched, including the Notch signaling pathway (NES = -1.88, p = 0.0040), base excision repair (NES = -1.61, p = 0.043), tight junction (NES = -1.52, p = 0.033), glycerophospholipid metabolism (NES = -1.42, p = 0.042), and RNA polymerase (NES = -1.40, p = 0.013) (**Figure 6B**). Next, we analyzed differences in biological processes and pathways between the two risk groups based on the HEMTIRGs signature. The DEGs between the high- and low-risk groups were identified using adjusted p-value (adj. p)< 0.05 and |log_2_FoldChange| > 1 as cutoffs (**Supplementary Table S8**). Gene Ontology (GO) enrichment and KEGG pathway analyses revealed 295 biological processes (BPs), 6 cellular components (CCs), and 49 molecular functions (MFs) (**Supplementary Table S9**). The top 10 enriched BPs, CCs, and MFs are illustrated in **Figures 6C–E**. KEGG pathway analysis identified 15 significantly enriched pathways (**Supplementary Table S10**), with the IL-17 and AKT signaling pathway showing a high degree of enrichment (**Figure 6F**).

### 3.6 Immune status analysis for BC based on HEMTIRGs

**Figure 7:**
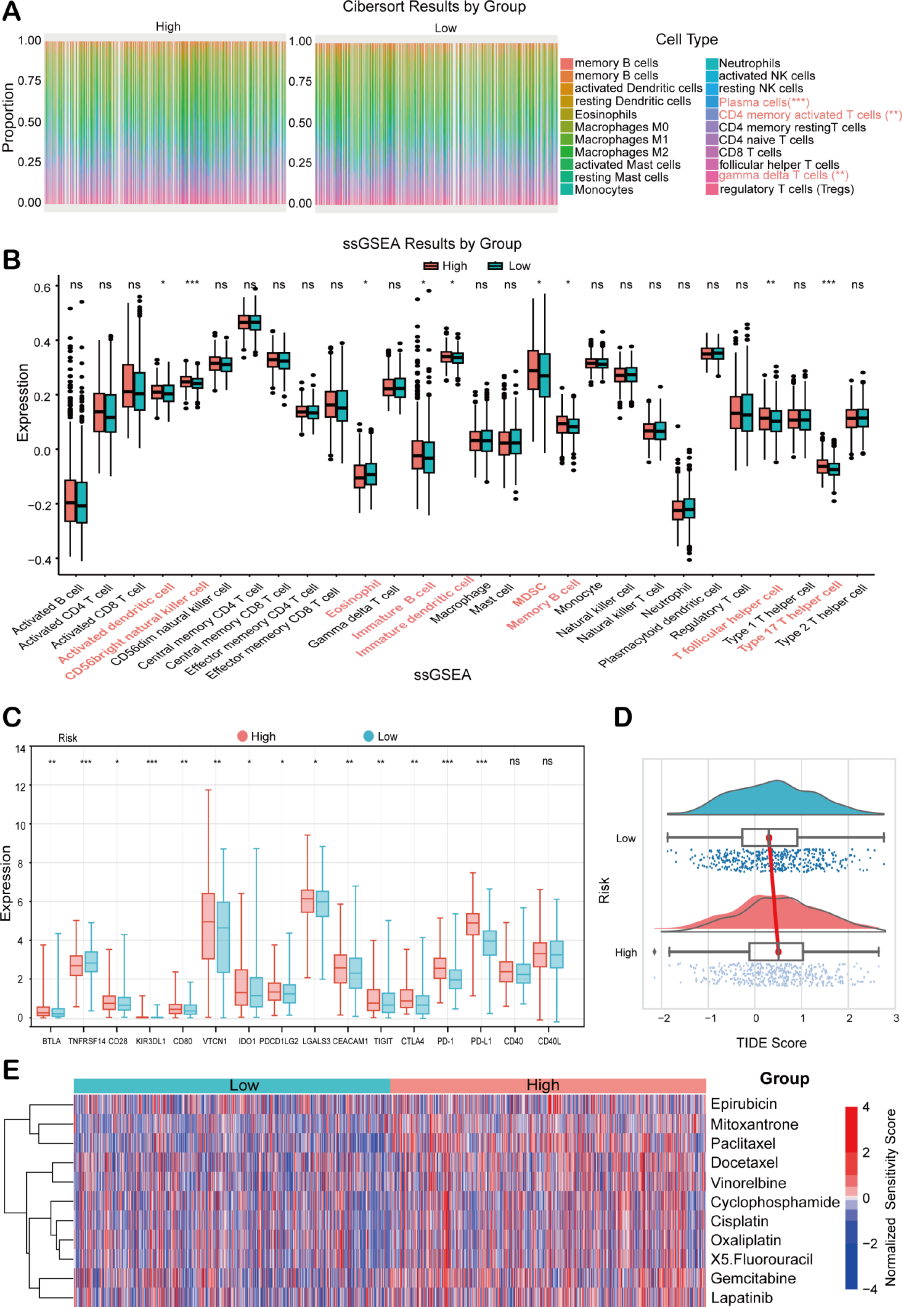
Analysis of the immune status in BC patients combined with prognostic modeling. **(A)** The relative proportions of 22 tumor-infiltrating immune cell types in high- and low-risk groups of the TCGA BRCA cohort. **(B)** ssGSEA scores in the high- and low-risk groups. **(C)** Immune checkpoint expression analysis between high- and low-risk groups. **(D)** TIDE score analysis between high- and low-risk groups. **(E)** Chemotherapeutic drug sensitivity analysis between high- and low-risk patients. Statistical significance was determined by an unpaired Student’s t-test. *p < 0.05, **p < 0.01, ***p < 0.001, ns, not significant.

To comprehensively characterize the immune status analysis based on HEMTIRGs within the context of risk stratification in BC, CIBERSORT and single-sample Gene Set Enrichment Analysis (ssGSEA) were performed in high-risk and low-risk groups. The CIBERSORT algorithm was used to assess the distribution of 22 immune cell types, revealing significantly higher infiltration levels of plasma cells (p< 0.001), activated memory CD4^+^ T cells, and γδ T cells (p< 0.001) in the high-risk group compared to the low-risk group (**Figure 7A**). These results suggest a positive correlation between risk score and the infiltration of these immune cell types in the high-risk group. Additionally, ssGSEA analysis indicated significant upregulation of genes associated with 9 immune cell subtypes (activated dendritic cell, CD56bright natural killer cell, eosinophil, immature B cell, immature dendritic cell, MDSC, memory B cell, T follicular helper cell, Type 17 T helper cell) in the high-risk group compared to the low-risk group (**Figure 7B**), underscoring a more pronounced immune cell infiltration in the high-risk cohort. We next compared the expression levels of 16 immune checkpoint molecules between these two groups. The high-risk group showed significantly higher expression of BTLA, CD28, KIR3DL1, CD80, VTCN1, IDO1, PDCD1LG2, LGALS3, CEACAM1, TIGIT, CTLA4, PD-1, and PD-L1, whereas TNFRSF14 expression was markedly lower in this group (**Figure 7C**). To assess the predictive potential of our model for immunotherapy response, we conducted a Tumor Immune Dysfunction and Exclusion (TIDE) analysis. The high-risk group, with its elevated TIDE score, exhibited a significantly poorer response to immunotherapy compared to the low-risk group (**Figure 7D**). Furthermore, drug sensitivity analysis revealed that high-risk patients exhibited reduced responsiveness to multiple conventional chemotherapeutic agents, including epirubicin, mitoxantrone, paclitaxel, docetaxel, vinorelbine, cyclophosphamide, cisplatin, oxaliplatin, 5-fluorouracil, gemcitabine, and lapatinib (**Figure 7E**). This pattern suggests a broader therapy resistance in the high-risk subgroup, potentially reflecting more aggressive tumor biology and underscoring the need to explore alternative therapeutic strategies for these patients.

### 3.7 Expression analysis of HEMTIRGs in the TCGA BRCA cohort

**Figure 8:**
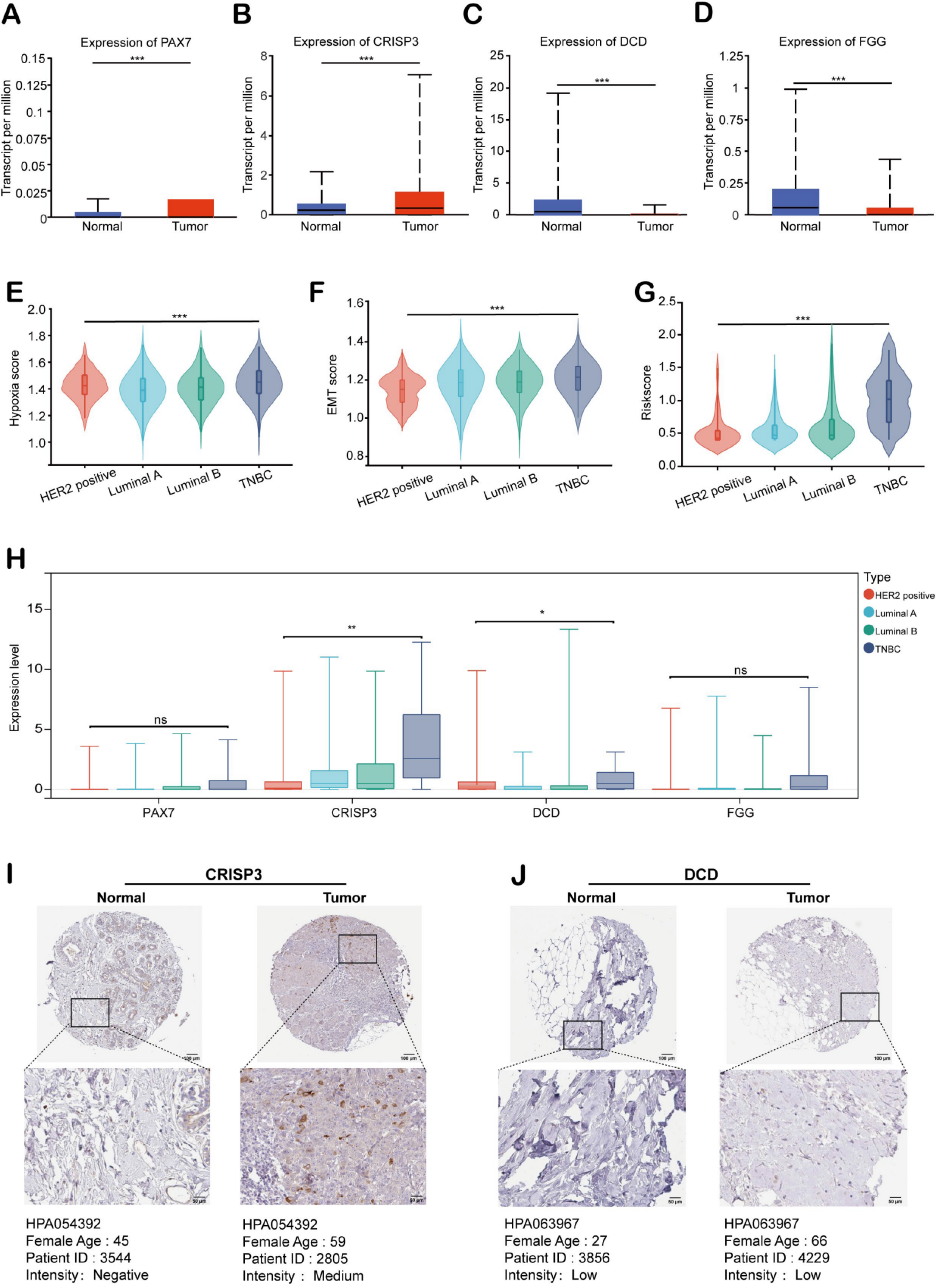
Expression analysis of HEMTIRGs in the TCGA BRCA cohort. Expression of **(A)** PAX7, **(B)** CRISP3, **(C)** DCD, and **(D)** FGG in BC and normal tissues for the TCGA BRCA cohort. **(E)** Hypoxia, **(F)** EMT, and **(G)** hypoxia-EMT risk scores for four subtypes of BC. **(H)** Expression of PAX7, CRISP3, DCD, and FGG in four subtypes of BC. **(I, J)** Immunohistochemical images of CRISP3 and DCD protein in normal and BC tissues. Protein expression was quantified based on the percentage of immunopositive cells and categorized as follows: negative (0%), weakly positive (<25%), moderately positive (25-75%), or strongly positive (>75%). Statistical significance was determined using one-way ANOVA. *p< 0.05, **p < 0.01, ***p < 0.001, ns, not significant.

We evaluated the expression patterns of HEMTIRGs in BC and adjacent normal tissues from the TCGA BRCA cohort. PAX7 and CRISP3 were significantly upregulated, while DCD and FGG were markedly downregulated in BC samples (**Figures 8A–D**). To further investigate HEMTIRGs expression across BC subtypes, we employed GSEA to assess differences in hypoxia, EMT, and hypoxia-EMT risk scores in four BC subtypes: hormone receptor-positive (Luminal A and Luminal B), HER2-positive, and triple-negative BC (TNBC), revealing that TNBC exhibited consistently higher scores for these features compared to the other subtypes (**Figures 8E–G**). Among the four BC subtypes, CRISP3 and DCD expression were significantly elevated in TNBC (**Figure 8H**). To confirm these findings at the protein level, we performed immunohistochemical (IHC) analysis using data from the Human Protein Atlas (HPA). The results corroborated the transcriptional data, revealing significantly higher CRISP3 protein expression in BC tissue compared to normal tissue, whereas DCD protein expression showed no significant difference (**Figures 8I, J**). These findings highlight the critical role of CRISP3 in BC pathogenesis, particularly in TNBC.

### 3.8 *In vitro* functional validation of CRISP3 in BC cells

**Figure 9:**
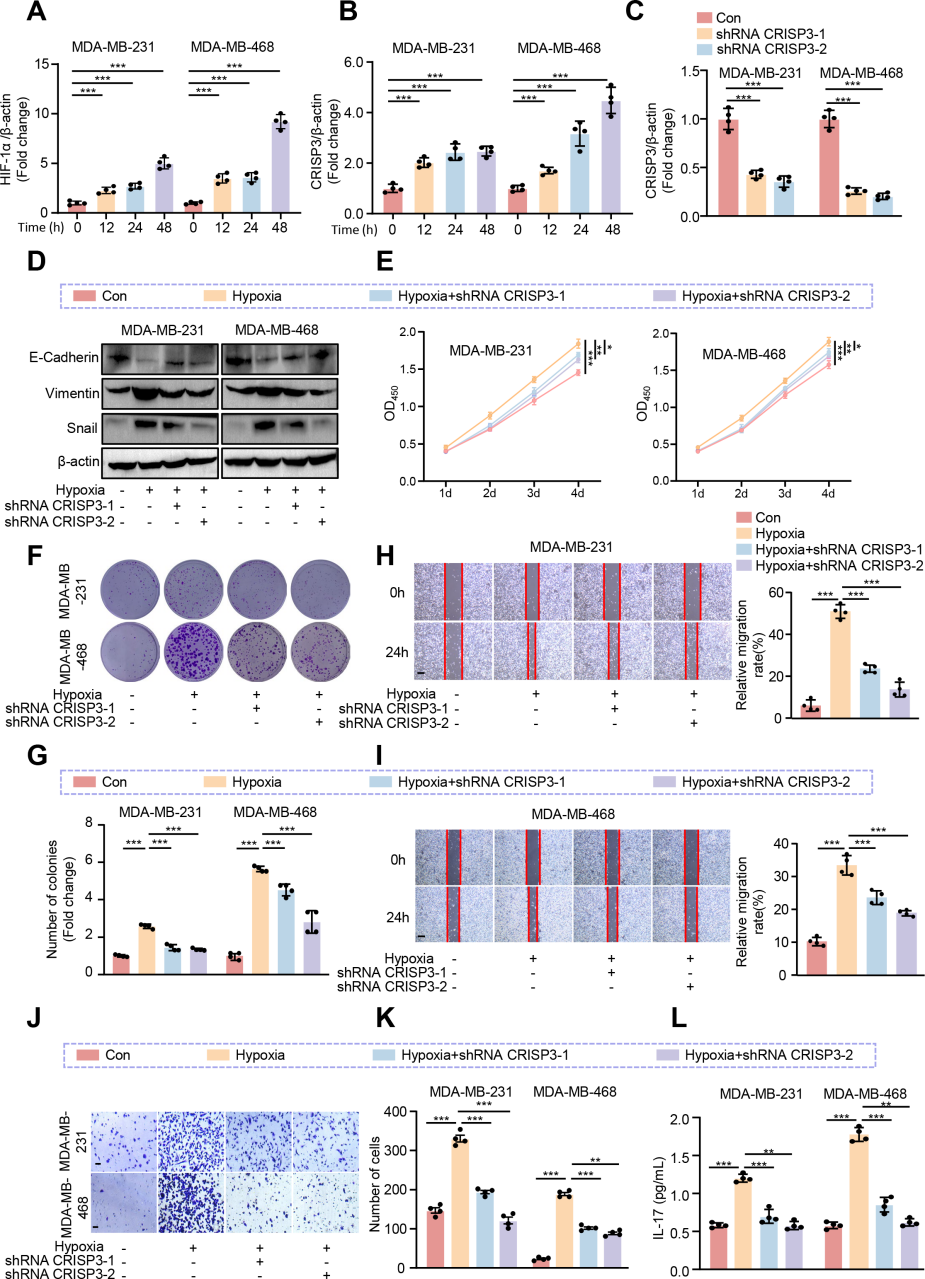
CRISP3 knockdown attenuates hypoxia-induced TNBC cell proliferation, migration, and EMT. MDA-MB-231 and MDA-MB-468 cells were transduced with pLKO.1-TRC shRNA as control (Con) or pLKO.1-CRISP3 shRNA to silence CRISP3 (shRNA CRISP3). qRT-PCR analysis of the mRNA expression of **(A)** HIF-1α and **(B)** CRISP3 under hypoxia conditions in MDA-MB-231 and MDA-MB-468 cells. **(C)** qRT-PCR analysis of CRISP3 silencing efficiency in MDA-MB-231 and MDA-MB-468 cells. **(D)** Immunoblotting analysis of EMT markers. **(E)** The CCK8 assay, and **(F–G)** colony formation assay were employed to evaluate cell proliferation. **(H, I)** Wound healing assay, and **(J–K)** transwell assay were conducted for assessment of migrative ability. **(L)** ELISA was performed to measure the level of IL-17 in the conditioned medium of MDA-MB-231 and MDA-MB-468 cells. Statistical significance was determined using one-way ANOVA. Data are presented as mean ± SEM (n = 4). *p < 0.05, **p < 0.01, ***p < 0.001. Scale bar = 0.1 mm.

To explore and validate the hypoxia responses of CRISP3 in TNBC cells, MDA-MB-231 and MDA-MB-468 TNBC cell lines were exposed to hypoxic conditions for 0, 12, 24, and 48 hours, as confirmed by the remarkable upregulation of HIF-1α mRNA expression (**Figure 9A**). qRT-PCR analysis revealed a remarkable upregulation of CRISP3 in TNBC cells under hypoxic conditions (**Figure 9B**). In contrast, PAX7, FGG, and DCD showed only modest changes in mRNA expression in response to hypoxia (**Supplementary Figures S4A–C**), indicating that CRISP3 is the most robustly hypoxia-inducible gene among the identified HEMTIRGs. To further validate the function and action mechanism of CRISP3 in TNBC cells, we applied lentivirus-mediated shRNA to knockdown CRISP3, as validated by qRT-PCR (**Figure 9C**). Next, we silenced CRISP3 under hypoxic conditions in MDA-MB-231 and MDA-MB-468 cells to evaluate the EMT process. Notably, CRISP3 knockdown significantly suppressed the expression of vimentin and snail while restoring E-cadherin expression under hypoxia (**Figure 9D**, **Supplementary Figures S5A–C**). Consistently, CCK-8 and colony formation assays demonstrated that CRISP3 depletion significantly reduced hypoxia-induced cell proliferation (**Figures 9E–G**) in both TNBC cell lines. Moreover, wound healing assays and transwell invasion revealed that CRISP3 knockdown led to a significant reduction in cell migration (**Figures 9H, I**) and invasion (**Figures 9J, K**) under hypoxic conditions. Intriguingly, IL-17 levels were elevated in the supernatants of hypoxia-treated TNBC cells, whereas CRISP3 knockdown significantly attenuated this elevation in IL-17 production (**Figure 9L**). These findings indicate that CRISP3 may promote TNBC cell proliferation, migration, invasion, as well as EMT by enhancing IL-17 production under hypoxic conditions.

### 3.9 CRISP3 promotes the pro-carcinogenic progression through activation of the IL-17/AKT signaling axis in BC cells

**Figure 10:**
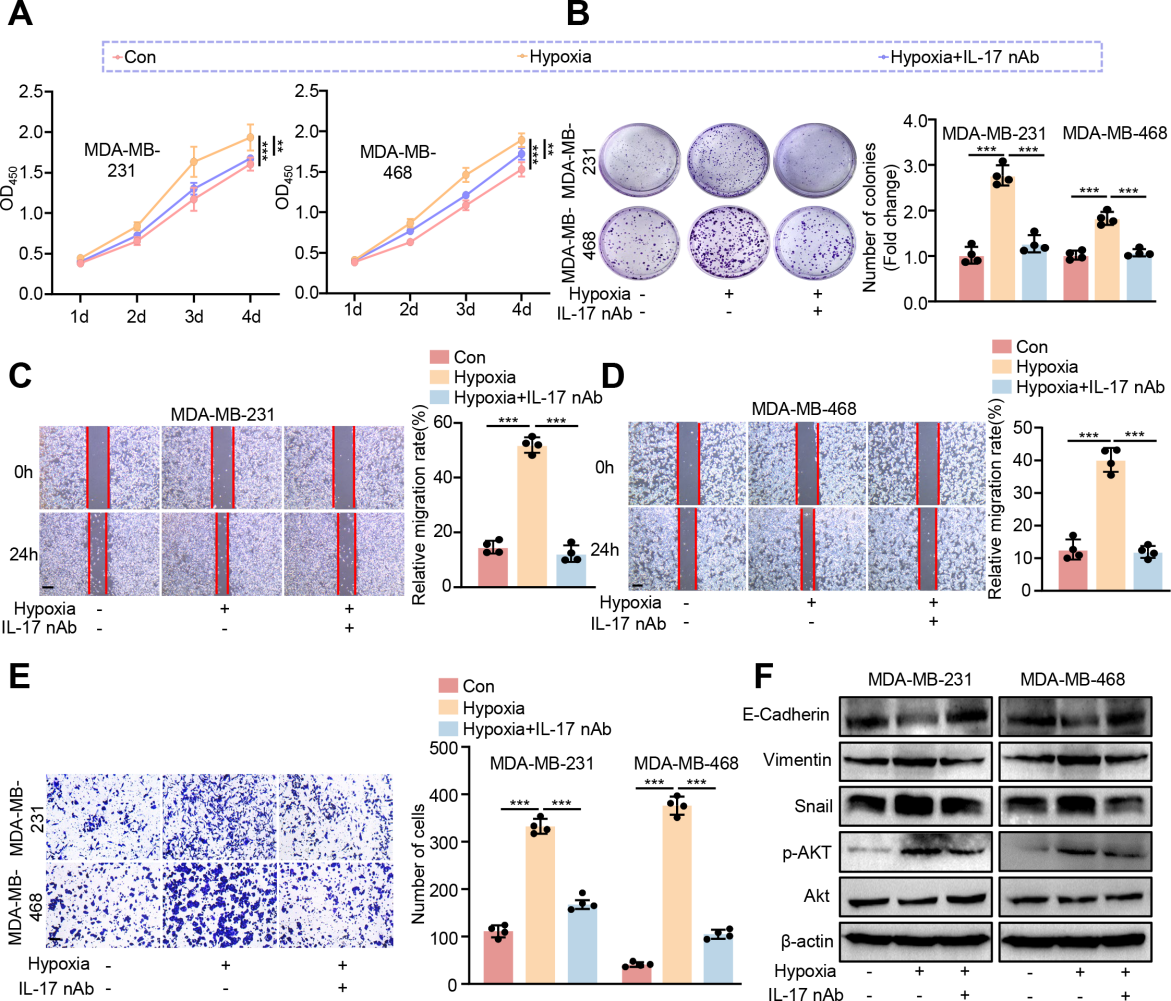
Blocking IL-17 prevents hypoxia-induced pro-carcinogenic progression in BC cells. MDA-MB-231 and MDA-MB-468 cells were exposed to hypoxic conditions (1% O_2_, 5% CO_2_, and 94% N_2_) and subsequently treated with IL-17 neutralizing antibody (nAb) at a final concentration of 10 μg/mL. **(A)** CCK8 assay. **(B)** Colony formation assay. **(C, D)** Wound healing assay. **(E)** The transwell assay. **(F)** Immunoblotting analysis of EMT markers, AKT, and p-AKT. Statistical significance was determined using one-way ANOVA. Data are presented as mean ± SEM (n = 4). **p < 0.01, ***p < 0.001. Scale bar = 0.1 mm.

To further verify whether CRISP3 drives BC progression through elevation of IL-17 production, we treated TNBC cell lines exposed to hypoxia with IL-17 neutralizing antibodies (IL-17 nAb). Consistently, we found that IL-17 neutralizing antibody treatment significantly inhibited hypoxia-induced cell viability (**Figure 10A**), proliferation (**Figure 10B**), migration (**Figures 10C, D**), and invasion (**Figure 10E**) of MDA-MB-231 and MDA-MB-468 cells. Moreover, we observed that IL-17 nAb significantly prevented hypoxia-induced EMT processes in MDA-MB-231 and MDA-MB-468 cell lines, as demonstrated by elevated E-cadherin and reduced vimentin and snail expression levels as compared to the hypoxia treatment group (**Figure 10F**, **Supplementary Figures S5D–F**). In our KEGG pathway enrichment analysis, we found that the AKT signaling pathway was also significantly enriched. Accumulating evidence has demonstrated that hypoxia is strongly implicated with activation of AKT signaling in a variety of cell types (51–53), and IL-17 can exert tumor-promoting effects through activation of the AKT pathway (54–56), which prompts us to consider whether CRISP3 drives the pro-carcinogenic progression through activation of the IL-17/AKT signaling axis in BC cells. As expected, IL-17 nAb was prominent to prevent hypoxia-induced AKT activation (**Figure 10F**), with quantification data shown in **Supplementary Figure S5G**.

## 4 Discussion

BC remains the most prevalent malignancy among women globally, yet challenges persist in achieving personalized treatment and accurate prognostic evaluation. Advances in bioinformatics have enabled the identification of numerous aberrantly expressed oncogenes, which hold potential as prognostic signatures in BC (57, 58). However, the prognostic potential gene signature based on HEMTIRGs has remained largely unexplored. In this study, we identified four key HEMTIRGs and developed a prognostic model that demonstrated high reliability and accuracy in predicting patient outcomes and guiding immunotherapy in BC. Notably, comprehensive bioinformatics analyses combined with *in vitro* experiments confirmed the strong involvement of HEMTIRGs in BC pathogenesis, with strong associations to hypoxia, EMT, and immune regulation. These findings highlight the HEMTIRGs signature as a promising biomarker for prognosis, survival risk stratification, and the development of personalized treatment strategies in BC.

Here, we identified four HEMTIRGs, including PAX7, DCD, CRISP3, and FGG, in the BRCA cohort. Based on this HEMTIRGs signature, we developed a novel prognostic risk evaluation model using LASSO Cox regression analysis. In previous studies, the prognostic model solely based on hypoxia-associated markers (46), EMT-related lncRNAs (59), or immune cell signatures (45) has been established. In this study, our HEMTIRGs-based model integrates hypoxia, EMT, and immune-related genes, offering a distinct approach with unique advantages for prognostic prediction. To assess its performance, we conducted a comparative analysis with other existing models, including those based on ferroptosis-related genes (44), hypoxia-associated markers (46), and immune cell signatures (45). Our results demonstrate that the HEMTIRGs model outperforms these alternatives in accuracy and reliability for predicting overall survival (OS) in BC patients. Additionally, we constructed an HEMTIRGs-based nomogram to predict 1-, 3-, and 5-year OS in the TCGA BRCA cohort. Calibration curves revealed a strong concordance between predicted and observed OS at these intervals, indicating that the nomogram provides significantly greater predictive accuracy than individual clinical indicators alone. Artificial intelligence (AI) is increasingly transforming breast imaging, with the potential to enhance diagnostic efficiency (60). In further studies, an extended nomogram model based on HEMTIRGs combining with AI and clinical features could be developed to facilitate cancer detection and support clinical decision-making. In clinical practice, the HEMTIRG-derived risk score could be incorporated into routine molecular testing or immunohistochemistry to stratify breast cancer patients into high- and low-risk groups. Such stratification may guide preoperative treatment planning, postoperative surveillance scheduling, and the selection of immunotherapy or combination regimens. Moreover, the nomogram established in this study could be embedded into clinical workflows or electronic medical record systems as a decision-support tool, providing oncologists with individualized survival predictions to improve prognosis assessment and treatment planning. In addition, immunohistochemical analysis confirmed aberrant expression of HEMTIRGs in BC tissue samples as compared to the normal group, underscoring their potential as diagnostic biomarkers for BC diagnosis. Nevertheless, the clinical applicability of HEMTIRGs for BC prognosis and diagnosis warrants further validation in prospective and multicenter studies.

To investigate the potential mechanisms through which HEMTIRGs modulate the malignant progression of BC, we examined the biological processes and signaling pathways of HEMTIRGs in both high-risk and low-risk groups. GSEA, GO, and KEGG analyses reveal that HEMTIRGs were significantly associated with several BC-related pathways, including steroid biosynthesis (61), citrate cycle TCA cycle (62), notch signaling pathway (63), tight junction (64), glycerophospholipid metabolism (65), humoral immune response (66), Akt signaling pathway (67) and IL-17 signaling pathway (68). Mechanistically, HEMTIRGs may contribute to the pathogenesis of BC by regulating hypoxia, EMT, and immune responses through the enriched pathways, which provides novel perspectives to elucidate the molecular mechanisms driving BC pathogenesis and offer potential targets for therapeutic intervention.

Tumor-infiltrating immune cells play a critical role in BC progression by infiltrating the tumor microenvironment (TME) and interacting with cancer cells and other immune components to promote malignant phenotypes (69). In this present study, we performed CIBERSORT and ssGSEA analyses to assess the involvement of immune cell infiltration in BC, revealing significantly elevated levels of immune cell populations, including CD4^+^ T cells, γδ T cells, and dendritic cells, in the high-risk group. Elevated immune cell infiltration within the TME may promote the progression of BC, thereby contributing to the poorer prognosis observed in high-risk patients. Additionally, tumor-infiltrating immune cells have been recognized as prognostic markers for chemotherapy response and survival in BC (70). Moreover, we observed that the expression levels of 13 immune checkpoint molecules (BTLA, CD28, KIR3DL1, CD80, VTCN1, IDO1, PDCDLG2, LGALS3, CEACAM1, TIGIT, CTLA4, PD-1, and PD-L1) were significantly elevated in the high-risk group, while TNFRSF14 expression was notably reduced. Several of these molecules have been previously implicated in BC development and progression, including CD28 (71), CD80 (72), VTCN1 (73), IDO1 (74), PDCDLG2 (75), TIGIT (76), PD-1, and PD-L1 (77). Whereas the roles of BTLA, KIR3DL1, LGALS3, CEACAM1, and TNFSF4 in modulating BC remain poorly understood, highlighting the need for further investigation.

Notably, our experimental work validated that CRISP3 is a key HEMTIRG that modulates the biological functions of breast cancer cells under hypoxic conditions through the IL-17/AKT signaling pathway. For the first time, we found that CRISP3 expression was significantly upregulated in BC cell lines following hypoxic exposure, and CRISP3 depletion significantly attenuated hypoxia-induced cell proliferation, migration, invasion, and EMT, which is in line with the prognostic prediction model based on HEMTIRGs. Consistently, in patients with mammary carcinoma, it has been found that higher expression of CRISP3 was connected to a significantly decreased disease-free survival and overall survival (78). A significant higher mRNA and protein levels of CRISP3 were seen in T-47D as well as SK-BR-3 human breast cancer cell lines compared with those in other types of mammary carcinoma cells, and knockdown of CRISP3 resulted in weakened migration or invasion abilities (78). Furthermore, our study identifies the IL-17/AKT signaling axis as the key pathway through which CRISP3 drives hypoxia-induced BC progression. IL-17 has been implicated in tumor-associated inflammation, immune evasion, and EMT through activation of AKT (55, 56). CRISP3 may facilitate IL-17 secretion or signaling, promoting an immunosuppressive and pro-metastatic environment. The observed attenuation of hypoxia-induced proliferation and EMT upon CRISP3 depletion suggests that CRISP3 may amplify AKT phosphorylation, reinforcing its oncogenic function in BC. Notably, CRISP3-mediated upregulation of IL-17 in BC cells (**Figure 9L**) may contribute to the enrichment of Th17 and γδ T lymphocytes observed in the high-risk group (**Figure 7**), suggesting a potential feed-forward loop between tumor-intrinsic signaling and immune microenvironment remodeling. As IL-17 is a signature cytokine of these cell types, CRISP3 may both promote tumor cell proliferation *via* IL-17/AKT signaling and facilitate the recruitment or expansion of IL-17–producing immune cells, thereby reinforcing an immunosuppressive tumor microenvironment. This dual mechanism highlights the role of CRISP3 in linking hypoxia-driven tumor progression to an adverse immune milieu, ultimately contributing to the poor prognosis observed in high-risk patients. Although the functional role of PAX7, FGG, and DCD in modulating BC malignant progression has not been validated in this study, emerging evidence suggests that they may be directly or indirectly involved in BC pathogenesis associated with hypoxia and EMT. PAX7, as a transcription factor, has been implicated in EMT (79), a critical process for cancer cell migration and invasion. It may drive Snail, Slug, and ZEB1/2 expression, leading to E-cadherin suppression and vimentin upregulation, hallmarks of EMT. FGG is a key component of fibrinogen, traditionally involved in blood clotting and wound healing. However, recent studies suggest that FGG plays a crucial role in tumor progression, particularly in promoting angiogenesis, metastasis, and immune suppression. FGG has been identified and characterized as a potential prognostic gene for predicting overall survival in hepatocellular carcinoma (HCC) patients, which enhances HCC cell migration and invasion by activating EMT (80). Moreover, silencing FGG in lung squamous cell carcinoma (LUSC) tissue notably altered the extent of immune infiltration, particularly affecting the infiltration of M1-type macrophages derived from THP-1 cell polarization (80). DCD, a secreted antimicrobial peptide, has been found to be dysregulated in a subset of breast tumors (81). Patients with DCD-positive breast cancer have worse prognostic features (81). Bancovik et al. revealed that DCD modulated its oncogenic role in breast cancer by the ERBB signaling (81). Taken together, these evidences confirm that these HEMTIRGs (CRISP3, PAX7, FGG, and DCD) are closely related to hypoxia, EMT, and tumor immunity, which in turn contribute to the regulation of BC pathogenesis and prognosis.

Although our study developed a novel prognostic risk model for BC based on HEMTIRGs, demonstrating strong accuracy and reliability, certain limitations remain. In particular, the functional role of CRISP3 in hypoxia-induced BC progression was only validated *in vitro*. Future studies should include *in vivo* models to strengthen these mechanistic insights. Additionally, future research should focus on validating the model’s predictive performance and its effectiveness in stratifying BC patients, forecasting prognosis, and assessing immunotherapy responses in larger clinical cohorts. Finally, the exact molecular mechanisms through which HEMTIRGs contribute to BC pathogenesis and progression require further investigation to fully elucidate their role in BC development and potential therapeutic targeting.

## 5 Conclusion

In conclusion, we identified a novel and reliable prognostic HEMTIRGs signature through bioinformatics analysis of hypoxia-, EMT-, and immune-related genes in the BC training cohort. A prognostic risk model based on HEMTIRGs effectively stratified BC patients, and demonstrated excellent reliability and accuracy in predicting BC prognosis and assessing immunotherapy efficacy, offering valuable insights for risk assessment, personalized treatment strategies, and clinical decision-making in BC management. Furthermore, our findings enhance the understanding of hypoxia- and EMT-driven mechanisms underlying BC progression and prognosis, highlighting novel therapeutic targets for BC treatment.

## Data Availability

The original contributions presented in the study are included in the article/**Supplementary Material**. Further inquiries can be directed to the corresponding author/s.
