# Supplementary material for: Integrative bioinformatics and experimental validation unveil CRISP3 as a hypoxia-, epithelial mesenchymal transition-, and immune-related prognostic biomarker and therapeutic target in breast cancer
Source: Front Immunol. 2025 Oct 22;16:1634399. doi: 10.3389/fimmu.2025.1634399 (PMC12585952; doi:10.3389/fimmu.2025.1634399)
Supplement: Supplementary file 1 [file Table1.docx]

**Supplementary Table S1. The list of hypoxia-related genes.**

| **Gene Symbol** | **Description** | **Category** | **Uniprot ID** | **Gifts** | **GC ID** |
| --- | --- | --- | --- | --- | --- |
| ADM | Adrenomedullin | Protein Coding | P35318 | 57 | GC11P023149 |
| ADORA2B | Adenosine A2b Receptor | Protein Coding | P29275 | 60 | GC17P164335 |
| AK4 | Adenylate Kinase 4 | Protein Coding | P27144 | 50 | GC01P065147 |
| AKAP12 | A-Kinase Anchoring Protein 12 | Protein Coding | Q02952 | 51 | GC06P151239 |
| ALDOA | Aldolase, Fructose-Bisphosphate A | Protein Coding | P04075 | 62 | GC16P030064 |
| ALDOB | Aldolase, Fructose-Bisphosphate B | Protein Coding | P05062 | 59 | GC09M101420 |
| ALDOC | Aldolase, Fructose-Bisphosphate C | Protein Coding | P09972 | 50 | GC17M100469 |
| AMPD3 | Adenosine Monophosphate Deaminase 3 | Protein Coding | Q01432 | 56 | GC11P023152 |
| ANGPTL4 | Angiopoietin Like 4 | Protein Coding | Q9BY76 | 56 | GC19P008363 |
| ANKZF1 | Ankyrin Repeat And Zinc Finger Peptidyl TRNA Hydrolase 1 | Protein Coding | Q9H8Y5 | 47 | GC02P219229 |
| ANXA2 | Annexin A2 | Protein Coding | P07355 | 60 | GC15M060347 |
| ATF3 | Activating Transcription Factor 3 | Protein Coding | P18847 | 57 | GC01P212565 |
| ATP7A | ATPase Copper Transporting Alpha | Protein Coding | Q04656 | 59 | GC0XP078277 |
| B3GALT6 | Beta-1,3-Galactosyltransferase 6 | Protein Coding | Q96L58 | 52 | GC01P001232 |
| B4GALNT2 | Beta-1,4-N-Acetyl-Galactosaminyltransferase 2 (SID Blood Group) | Protein Coding | Q8NHY0 | 53 | GC17P165493 |
| BCAN | Brevican | Protein Coding | Q96GW7 | 55 | GC01P156641 |
| BCL2 | BCL2 Apoptosis Regulator | Protein Coding | P10415 | 64 | GC18M063123 |
| BGN | Biglycan | Protein Coding | P21810 | 57 | GC0XP153494 |
| BHLHE40 | Basic Helix-Loop-Helix Family Member E40 | Protein Coding | O14503 | 53 | GC03P005086 |
| BNIP3L | BCL2 Interacting Protein 3 Like | Protein Coding | O60238 | 55 | GC08P026383 |
| BRS3 | Bombesin Receptor Subtype 3 | Protein Coding | P32247 | 54 | GC0XP136545 |
| BTG1 | BTG Anti-Proliferation Factor 1 | Protein Coding | P62324 | 53 | GC12M092140 |
| CA12 | Carbonic Anhydrase 12 | Protein Coding | O43570 | 59 | GC15M063321 |
| CASP6 | Caspase 6 | Protein Coding | P55212 | 61 | GC04M109688 |
| CAV1 | Caveolin 1 | Protein Coding | Q03135 | 61 | GC07P116524 |
| KLF6 | KLF Transcription Factor 6 | Protein Coding | Q99612 | 56 | GC10M003779 |
| KLF7 | KLF Transcription Factor 7 | Protein Coding | O75840 | 51 | GC02M207074 |
| KLHL24 | Kelch Like Family Member 24 | Protein Coding | Q6TFL4 | 44 | GC03P183635 |
| LALBA | Lactalbumin Alpha | Protein Coding | P00709 | 52 | GC12M048567 |
| LARGE | LARGE Xylosyl- And Glucuronyltransferase | Protein Coding | O95461 | 53 | GC22M088338 |
| LDHA | Lactate Dehydrogenase A | Protein Coding | P00338 | 63 | GC11P018394 |
| LDHC | Lactate Dehydrogenase C | Protein Coding | P07864 | 53 | GC11P023553 |
| LOX | Lysyl Oxidase | Protein Coding | P28300 | 60 | GC05M122063 |
| LXN | Latexin | Protein Coding | Q9BS40 | 44 | GC03M158645 |
| MAFF | MAF BZIP Transcription Factor F | Protein Coding | Q9ULX9 | 49 | GC22P038200 |
| MAP3K1 | Mitogen-Activated Protein Kinase Kinase Kinase 1 | Protein Coding | Q13233 | 63 | GC05P056815 |
| MIF | Macrophage Migration Inhibitory Factor | Protein Coding | P14174 | 60 | GC22P023894 |
| MT1E | Metallothionein 1E | Protein Coding | P04732 | 52 | GC16P056625 |
| MT2A | Metallothionein 2A | Protein Coding | P02795 | 56 | GC16P129478 |
| MXI1 | MAX Interactor 1, Dimerization Protein | Protein Coding | P50539 | 53 | GC10P129309 |
| MYH9 | Myosin Heavy Chain 9 | Protein Coding | P35579 | 60 | GC22M036281 |
| NAGK | N-Acetylglucosamine Kinase | Protein Coding | Q9UJ70 | 51 | GC02P071064 |
| NCAN | Neurocan | Protein Coding | O14594 | 56 | GC19P161679 |
| NDRG1 | N-Myc Downstream Regulated 1 | Protein Coding | Q92597 | 57 | GC08M133237 |
| NDST1 | N-Deacetylase And N-Sulfotransferase 1 | Protein Coding | P52848 | 56 | GC05P161202 |
| NDST2 | N-Deacetylase And N-Sulfotransferase 2 | Protein Coding | P52849 | 49 | GC10M073801 |
| NEDD4L | NEDD4 Like E3 Ubiquitin Protein Ligase | Protein Coding | Q96PU5 | 57 | GC18P058044 |
| NFIL3 | Nuclear Factor, Interleukin 3 Regulated | Protein Coding | Q16649 | 47 | GC09M126468 |
| NR3C1 | Nuclear Receptor Subfamily 3 Group C Member 1 | Protein Coding | P04150 | 62 | GC05M143277 |
| P4HA1 | Prolyl 4-Hydroxylase Subunit Alpha 1 | Protein Coding | P13674 | 55 | GC10M073007 |
| ENO1 | Enolase 1 | Protein Coding | P06733 | 62 | GC01M008861 |
| ENO2 | Enolase 2 | Protein Coding | P09104 | 60 | GC12P006913 |
| ENO3 | Enolase 3 | Protein Coding | P13929 | 61 | GC17P004948 |
| ERO1L | Endoplasmic Reticulum Oxidoreductase 1 Alpha | Protein Coding | Q96HE7 | 50 | GC14M052640 |
| ERRFI1 | ERBB Receptor Feedback Inhibitor 1 | Protein Coding | Q9UJM3 | 50 | GC01M008004 |
| ETS1 | ETS Proto-Oncogene 1, Transcription Factor | Protein Coding | P14921 | 61 | GC11M128458 |
| EXT1 | Exostosin Glycosyltransferase 1 | Protein Coding | Q16394 | 60 | GC08M117798 |
| F3 | Coagulation Factor III, Tissue Factor | Protein Coding | P13726 | 58 | GC01M095087 |
| FAM162A | Family With Sequence Similarity 162 Member A | Protein Coding | Q96A26 | 41 | GC03P122384 |
| FBP1 | Fructose-Bisphosphatase 1 | Protein Coding | P09467 | 62 | GC09M094603 |
| FOS | Fos Proto-Oncogene, AP-1 Transcription Factor Subunit | Protein Coding | P01100 | 64 | GC14P075278 |
| FOSL2 | FOS Like 2, AP-1 Transcription Factor Subunit | Protein Coding | P15408 | 56 | GC02P028392 |
| FOXO3 | Forkhead Box O3 | Protein Coding | O43524 | 61 | GC06P108559 |
| GAA | Alpha Glucosidase | Protein Coding | P10253 | 63 | GC17P080101 |
| GALK1 | Galactokinase 1 | Protein Coding | P51570 | 60 | GC17M075751 |
| GAPDH | Glyceraldehyde-3-Phosphate Dehydrogenase | Protein Coding | P04406 | 64 | GC12P080272 |
| GAPDHS | Glyceraldehyde-3-Phosphate Dehydrogenase, Spermatogenic | Protein Coding | O14556 | 55 | GC19P161954 |
| GBE1 | 1,4-Alpha-Glucan Branching Enzyme 1 | Protein Coding | Q04446 | 56 | GC03M081489 |
| GCK | Glucokinase | Protein Coding | P35557 | 62 | GC07M045798 |
| GCNT2 | Glucosaminyl (N-Acetyl) Transferase 2 (I Blood Group) | Protein Coding | Q8N0V5 | 56 | GC06P010492 |
| GLRX | Glutaredoxin | Protein Coding | P35754 | 56 | GC05M095752 |
| GPC1 | Glypican 1 | Protein Coding | P35052 | 56 | GC02P240435 |
| GPC3 | Glypican 3 | Protein Coding | P51654 | 59 | GC0XM133535 |
| GPC4 | Glypican 4 | Protein Coding | O75487 | 56 | GC0XM133300 |
| GPI | Glucose-6-Phosphate Isomerase | Protein Coding | P06744 | 59 | GC19P034359 |
| PPP1R3C | Protein Phosphatase 1 Regulatory Subunit 3C | Protein Coding | Q9UQK1 | 52 | GC10M091628 |
| PRDX5 | Peroxiredoxin 5 | Protein Coding | P30044 | 57 | GC11P114946 |
| PRKCA | Protein Kinase C Alpha | Protein Coding | P17252 | 65 | GC17P066302 |
| PRKCDBP | Caveolae Associated Protein 3 | Protein Coding | Q969G5 | 48 | GC11M016037 |
| PTRF | Caveolae Associated Protein 1 | Protein Coding | Q6NZI2 | 53 | GC17M100983 |
| PYGM | Glycogen Phosphorylase, Muscle Associated | Protein Coding | P11217 | 60 | GC11M064746 |
| RBPJ | Recombination Signal Binding Protein For Immunoglobulin Kappa J Region | Protein Coding | Q06330 | 59 | GC04P026105 |
| RORA | RAR Related Orphan Receptor A | Protein Coding | P35398 | 61 | GC15M060488 |
| RRAGD | Ras Related GTP Binding D | Protein Coding | Q9NQL2 | 52 | GC06M089364 |
| S100A4 | S100 Calcium Binding Protein A4 | Protein Coding | P26447 | 57 | GC01M153543 |
| SAP30 | Sin3A Associated Protein 30 | Protein Coding | O75446 | 50 | GC04P173369 |
| SCARB1 | Scavenger Receptor Class B Member 1 | Protein Coding | Q8WTV0 | 60 | GC12M124776 |
| SDC2 | Syndecan 2 | Protein Coding | P34741 | 55 | GC08P096499 |
| SDC3 | Syndecan 3 | Protein Coding | O75056 | 54 | GC01M030869 |
| SDC4 | Syndecan 4 | Protein Coding | P31431 | 55 | GC20M045325 |
| SELENBP1 | Selenium Binding Protein 1 | Protein Coding | Q13228 | 54 | GC01M151364 |
| SERPINE1 | Serpin Family E Member 1 | Protein Coding | P05121 | 63 | GC07P101127 |
| SIAH2 | Siah E3 Ubiquitin Protein Ligase 2 | Protein Coding | O43255 | 55 | GC03M150741 |
| SLC25A1 | Solute Carrier Family 25 Member 1 | Protein Coding | P53007 | 58 | GC22M087890 |
| SLC2A1 | Solute Carrier Family 2 Member 1 | Protein Coding | P11166 | 66 | GC01M042925 |
| SLC2A3 | Solute Carrier Family 2 Member 3 | Protein Coding | P11169 | 61 | GC12M007919 |
| SLC2A5 | Solute Carrier Family 2 Member 5 | Protein Coding | P22732 | 55 | GC01M035975 |
| SLC37A4 | Solute Carrier Family 37 Member 4 | Protein Coding | O43826 | 53 | GC11M143843 |
| SLC6A6 | Solute Carrier Family 6 Member 6 | Protein Coding | P31641 | 58 | GC03P014402 |
| SRPX | Sushi Repeat Containing Protein X-Linked | Protein Coding | P78539 | 47 | GC0XM038149 |
| CCNG2 | Cyclin G2 | Protein Coding | Q16589 | 49 | GC04P077214 |
| CCRN4L | Nocturnin | Protein Coding | Q9UK39 | 40 | GC04P139016 |
| CDKN1A | Cyclin Dependent Kinase Inhibitor 1A | Protein Coding | P38936 | 63 | GC06P190835 |
| CDKN1B | Cyclin Dependent Kinase Inhibitor 1B | Protein Coding | P46527 | 61 | GC12P080490 |
| CDKN1C | Cyclin Dependent Kinase Inhibitor 1C | Protein Coding | P49918 | 59 | GC11M015881 |
| CHST2 | Carbohydrate Sulfotransferase 2 | Protein Coding | Q9Y4C5 | 43 | GC03P143119 |
| CHST3 | Carbohydrate Sulfotransferase 3 | Protein Coding | Q7LGC8 | 55 | GC10P071964 |
| CITED2 | Cbp/P300 Interacting Transactivator With Glu/Asp Rich Carboxy-Terminal Domain 2 | Protein Coding | Q99967 | 55 | GC06M139371 |
| COL5A1 | Collagen Type V Alpha 1 Chain | Protein Coding | P20908 | 59 | GC09P134641 |
| CP | Ceruloplasmin | Protein Coding | P00450 | 62 | GC03M149162 |
| CSRP2 | Cysteine And Glycine Rich Protein 2 | Protein Coding | Q16527 | 52 | GC12M076859 |
| CTGF | Cellular Communication Network Factor 2 | Protein Coding | P29279 | 59 | GC06M131948 |
| CXCR4 | C-X-C Motif Chemokine Receptor 4 | Protein Coding | P61073 | 65 | GC02M136114 |
| CXCR7 | Atypical Chemokine Receptor 3 | Protein Coding | P25106 | 53 | GC02P236537 |
| CYR61 | Cellular Communication Network Factor 1 | Protein Coding | O00622 | 52 | GC01P089746 |
| DCN | Decorin | Protein Coding | P07585 | 60 | GC12M091140 |
| DDIT3 | DNA Damage Inducible Transcript 3 | Protein Coding | P35638 | 59 | GC12M057516 |
| DDIT4 | DNA Damage Inducible Transcript 4 | Protein Coding | Q9NX09 | 56 | GC10P072273 |
| DPYSL4 | Dihydropyrimidinase Like 4 | Protein Coding | O14531 | 47 | GC10P132220 |
| DTNA | Dystrobrevin Alpha | Protein Coding | Q9Y4J8 | 56 | GC18P034493 |
| DUSP1 | Dual Specificity Phosphatase 1 | Protein Coding | P28562 | 59 | GC05M172768 |
| EDN2 | Endothelin 2 | Protein Coding | P20800 | 52 | GC01M041478 |
| EFNA1 | Ephrin A1 | Protein Coding | P20827 | 56 | GC01P155127 |
| EFNA3 | Ephrin A3 | Protein Coding | P52797 | 55 | GC01P155078 |
| EGFR | Epidermal Growth Factor Receptor | Protein Coding | P00533 | 68 | GC07P055019 |
| P4HA2 | Prolyl 4-Hydroxylase Subunit Alpha 2 | Protein Coding | O15460 | 57 | GC05M132193 |
| PAM | Peptidylglycine Alpha-Amidating Monooxygenase | Protein Coding | P19021 | 56 | GC05P102753 |
| PCK1 | Phosphoenolpyruvate Carboxykinase 1 | Protein Coding | P35558 | 60 | GC20P057561 |
| PDGFB | Platelet Derived Growth Factor Subunit B | Protein Coding | P01127 | 63 | GC22M089269 |
| PDK1 | Pyruvate Dehydrogenase Kinase 1 | Protein Coding | Q15118 | 60 | GC02P172555 |
| PDK3 | Pyruvate Dehydrogenase Kinase 3 | Protein Coding | Q15120 | 58 | GC0XP024465 |
| PFKFB3 | 6-Phosphofructo-2-Kinase/Fructose-2,6-Biphosphatase 3 | Protein Coding | Q16875 | 56 | GC10P006144 |
| PFKL | Phosphofructokinase, Liver Type | Protein Coding | P17858 | 57 | GC21P044300 |
| PFKP | Phosphofructokinase, Platelet | Protein Coding | Q01813 | 57 | GC10P003066 |
| PGAM2 | Phosphoglycerate Mutase 2 | Protein Coding | P15259 | 55 | GC07M044062 |
| PGF | Placental Growth Factor | Protein Coding | P49763 | 56 | GC14M074941 |
| PGK1 | Phosphoglycerate Kinase 1 | Protein Coding | P00558 | 62 | GC0XP078278 |
| PGM1 | Phosphoglucomutase 1 | Protein Coding | P36871 | 60 | GC01P063593 |
| PGM2 | Phosphoglucomutase 2 | Protein Coding | Q96G03 | 53 | GC04P040054 |
| PHKG1 | Phosphorylase Kinase Catalytic Subunit Gamma 1 | Protein Coding | Q16816 | 55 | GC07M056080 |
| PIM1 | Pim-1 Proto-Oncogene, Serine/Threonine Kinase | Protein Coding | P11309 | 62 | GC06P190843 |
| PKLR | Pyruvate Kinase L/R | Protein Coding | P30613 | 59 | GC01M155289 |
| PKP1 | Plakophilin 1 | Protein Coding | Q13835 | 52 | GC01P201283 |
| PLAC8 | Placenta Associated 8 | Protein Coding | Q9NZF1 | 43 | GC04M083090 |
| PLAUR | Plasminogen Activator, Urokinase Receptor | Protein Coding | Q03405 | 57 | GC19M043646 |
| PLIN2 | Perilipin 2 | Protein Coding | Q99541 | 55 | GC09M019328 |
| PNRC1 | Proline Rich Nuclear Receptor Coactivator 1 | Protein Coding | Q12796 | 43 | GC06P089080 |
| PPARGC1A | PPARG Coactivator 1 Alpha | Protein Coding | Q9UBK2 | 60 | GC04M023755 |
| PPFIA4 | PTPRF Interacting Protein Alpha 4 | Protein Coding | O75335 | 44 | GC01P203026 |
| PPP1R15A | Protein Phosphatase 1 Regulatory Subunit 15A | Protein Coding | O75807 | 53 | GC19P048872 |
| GRHPR | Glyoxylate And Hydroxypyruvate Reductase | Protein Coding | Q9UBQ7 | 58 | GC09P078116 |
| GYS1 | Glycogen Synthase 1 | Protein Coding | P13807 | 62 | GC19M112793 |
| HAS1 | Hyaluronan Synthase 1 | Protein Coding | Q92839 | 52 | GC19M112936 |
| HDLBP | High Density Lipoprotein Binding Protein | Protein Coding | Q00341 | 54 | GC02M241227 |
| HEXA | Hexosaminidase Subunit Alpha | Protein Coding | P06865 | 59 | GC15M072340 |
| HK1 | Hexokinase 1 | Protein Coding | P19367 | 63 | GC10P069269 |
| HK2 | Hexokinase 2 | Protein Coding | P52789 | 59 | GC02P075619 |
| HMOX1 | Heme Oxygenase 1 | Protein Coding | P09601 | 65 | GC22P035380 |
| HOXB9 | Homeobox B9 | Protein Coding | P17482 | 51 | GC17M048621 |
| HS3ST1 | Heparan Sulfate-Glucosamine 3-Sulfotransferase 1 | Protein Coding | O14792 | 52 | GC04M011394 |
| HSPA5 | Heat Shock Protein Family A (Hsp70) Member 5 | Protein Coding | P11021 | 62 | GC09M125234 |
| IDS | Iduronate 2-Sulfatase | Protein Coding | P22304 | 60 | GC0XM149476 |
| IER3 | Immediate Early Response 3 | Protein Coding | P46695 | 49 | GC06M030743 |
| IGFBP1 | Insulin Like Growth Factor Binding Protein 1 | Protein Coding | P08833 | 55 | GC07P052244 |
| IGFBP3 | Insulin Like Growth Factor Binding Protein 3 | Protein Coding | P17936 | 59 | GC07M045912 |
| IL6 | Interleukin 6 | Protein Coding | P05231 | 63 | GC07P022725 |
| ILVBL | 2-Hydroxyacyl-CoA Lyase 2 | Protein Coding | A1L0T0 | 46 | GC19M112057 |
| INHA | Inhibin Subunit Alpha | Protein Coding | P05111 | 57 | GC02P219569 |
| IRS2 | Insulin Receptor Substrate 2 | Protein Coding | Q9Y4H2 | 59 | GC13M109752 |
| ISG20 | Interferon Stimulated Exonuclease Gene 20 | Protein Coding | Q96AZ6 | 51 | GC15P088635 |
| JMJD6 | Jumonji Domain Containing 6, Arginine Demethylase And Lysine Hydroxylase | Protein Coding | Q6NYC1 | 55 | GC17M101862 |
| JUN | Jun Proto-Oncogene, AP-1 Transcription Factor Subunit | Protein Coding | P05412 | 63 | GC01M058780 |
| KDELR3 | KDEL Endoplasmic Reticulum Protein Retention Receptor 3 | Protein Coding | O43731 | 43 | GC22P038468 |
| KDM3A | Lysine Demethylase 3A | Protein Coding | Q9Y4C1 | 55 | GC02P100409 |
| KIF5A | Kinesin Family Member 5A | Protein Coding | Q12840 | 58 | GC12P081699 |
| STBD1 | Starch Binding Domain 1 | Protein Coding | O95210 | 46 | GC04P076306 |
| STC1 | Stanniocalcin 1 | Protein Coding | P52823 | 53 | GC08M023841 |
| STC2 | Stanniocalcin 2 | Protein Coding | O76061 | 53 | GC05M173314 |
| SULT2B1 | Sulfotransferase Family 2B Member 1 | Protein Coding | O00204 | 54 | GC19P048552 |
| TES | Testin LIM Domain Protein | Protein Coding | Q9UGI8 | 49 | GC07P116210 |
| TGFB3 | Transforming Growth Factor Beta 3 | Protein Coding | P10600 | 60 | GC14M075958 |
| TGFBI | Transforming Growth Factor Beta Induced | Protein Coding | Q15582 | 57 | GC05P136033 |
| TGM2 | Transglutaminase 2 | Protein Coding | P21980 | 60 | GC20M038127 |
| TIPARP | TCDD Inducible Poly(ADP-Ribose) Polymerase | Protein Coding | Q7Z3E1 | 48 | GC03P156673 |
| TKTL1 | Transketolase Like 1 | Protein Coding | P51854 | 50 | GC0XP154295 |
| TMEM45A | Transmembrane Protein 45A | Protein Coding | Q9NWC5 | 37 | GC03P100492 |
| TNFAIP3 | TNF Alpha Induced Protein 3 | Protein Coding | P21580 | 62 | GC06P137866 |
| TPBG | Trophoblast Glycoprotein | Protein Coding | Q13641 | 54 | GC06P191220 |
| TPD52 | Tumor Protein D52 | Protein Coding | P55327 | 52 | GC08M080031 |
| TPI1 | Triosephosphate Isomerase 1 | Protein Coding | P60174 | 59 | GC12P006867 |
| TPST2 | Tyrosylprotein Sulfotransferase 2 | Protein Coding | O60704 | 50 | GC22M088152 |
| UGP2 | UDP-Glucose Pyrophosphorylase 2 | Protein Coding | Q16851 | 56 | GC02P063840 |
| VEGFA | Vascular Endothelial Growth Factor A | Protein Coding | P15692 | 61 | GC06P043770 |
| VHL | Von Hippel-Lindau Tumor Suppressor | Protein Coding | P40337 | 60 | GC03P028787 |
| VLDLR | Very Low Density Lipoprotein Receptor | Protein Coding | P98155 | 61 | GC09P002611 |
| WISP2 | Cellular Communication Network Factor 5 | Protein Coding | O76076 | 47 | GC20P056234 |
| WSB1 | WD Repeat And SOCS Box Containing 1 | Protein Coding | Q9Y6I7 | 47 | GC17P027294 |
| XPNPEP1 | X-Prolyl Aminopeptidase 1 | Protein Coding | Q9NQW7 | 50 | GC10M109864 |
| ZFP36 | ZFP36 Ring Finger Protein | Protein Coding | P26651 | 50 | GC19P039406 |
| ZNF292 | Zinc Finger Protein 292 | Protein Coding | O60281 | 49 | GC06P191281 |

**Supplementary Table S2. The list of EMT-related genes.**

| **Gene Symbol** | **Description** | **Category** | **Uniprot ID** | **Gifts** | **GC ID** |
| --- | --- | --- | --- | --- | --- |
| TGFB1 | Transforming Growth Factor Beta 1 | Protein Coding | P01137 | 65 | GC19M041301 |
| EHMT2 | Euchromatic Histone Lysine Methyltransferase 2 | Protein Coding | Q96KQ7 | 58 | GC06M031879 |
| VDR | Vitamin D Receptor | Protein Coding | P11473 | 62 | GC12M047841 |
| PLAC8 | Placenta Associated 8 | Protein Coding | Q9NZF1 | 43 | GC04M083090 |
| EGR1 | Early Growth Response 1 | Protein Coding | P18146 | 58 | GC05P138465 |
| KLF5 | KLF Transcription Factor 5 | Protein Coding | Q13887 | 58 | GC13P073054 |
| CEACAM1 | CEA Cell Adhesion Molecule 1 | Protein Coding | P13688 | 58 | GC19M042507 |
| DKK3 | Dickkopf Wnt Signaling Pathway Inhibitor 3 | Protein Coding | Q9UBP4 | 53 | GC11M016206 |
| SNAI1 | Snail Family Transcriptional Repressor 1 | Protein Coding | O95863 | 57 | GC20P049982 |
| PROKR1 | Prokineticin Receptor 1 | Protein Coding | Q8TCW9 | 47 | GC02P068643 |
| CCR2 | C-C Motif Chemokine Receptor 2 | Protein Coding | P41597 | 58 | GC03P066708 |
| TFDP3 | Transcription Factor Dp Family Member 3 | Protein Coding | Q5H9I0 | 38 | GC0XM133216 |
| KLF8 | KLF Transcription Factor 8 | Protein Coding | O95600 | 50 | GC0XP061900 |
| ST14 | ST14 Transmembrane Serine Protease Matriptase | Protein Coding | Q9Y5Y6 | 60 | GC11P130159 |
| RPS6KB1 | Ribosomal Protein S6 Kinase B1 | Protein Coding | P23443 | 63 | GC17P059893 |
| BBC3 | BCL2 Binding Component 3 | Protein Coding | Q96PG8 | 53 | GC19M047220 |
| ZEB1 | Zinc Finger E-Box Binding Homeobox 1 | Protein Coding | P37275 | 61 | GC10P031318 |
| WASF3 | WASP Family Member 3 | Protein Coding | Q9UPY6 | 50 | GC13P029238 |
| PCSK1 | Proprotein Convertase Subtilisin/Kexin Type 1 | Protein Coding | P29120 | 62 | GC05M096391 |
| NES | Nestin | Protein Coding | P48681 | 53 | GC01M156668 |
| SREBF1 | Sterol Regulatory Element Binding Transcription Factor 1 | Protein Coding | P36956 | 61 | GC17M017810 |
| ALX4 | ALX Homeobox 4 | Protein Coding | Q9H161 | 51 | GC11M044238 |
| LYPD3 | LY6/PLAUR Domain Containing 3 | Protein Coding | O95274 | 47 | GC19M043460 |
| CDH1 | Cadherin 1 | Protein Coding | P12830 | 61 | GC16P068737 |
| CFTR | CF Transmembrane Conductance Regulator | Protein Coding | P13569 | 66 | GC07P117287 |
| NR2C2 | Nuclear Receptor Subfamily 2 Group C Member 2 | Protein Coding | P49116 | 57 | GC03P014947 |
| ING4 | Inhibitor Of Growth Family Member 4 | Protein Coding | Q9UNL4 | 50 | GC12M006650 |
| SOX3 | SRY-Box Transcription Factor 3 | Protein Coding | P41225 | 54 | GC0XM140502 |
| RNH1 | Ribonuclease/Angiogenin Inhibitor 1 | Protein Coding | P13489 | 50 | GC11M015782 |
| GJB2 | Gap Junction Protein Beta 2 | Protein Coding | P29033 | 58 | GC13M020187 |
| TWIST1 | Twist Family BHLH Transcription Factor 1 | Protein Coding | Q15672 | 59 | GC07M019020 |
| KHDRBS1 | KH RNA Binding Domain Containing, Signal Transduction Associated 1 | Protein Coding | Q07666 | 55 | GC01P032013 |
| TIMP2 | TIMP Metallopeptidase Inhibitor 2 | Protein Coding | P16035 | 54 | GC17M078852 |
| ANGPTL4 | Angiopoietin Like 4 | Protein Coding | Q9BY76 | 56 | GC19P008363 |
| SON | SON DNA And RNA Binding Protein | Protein Coding | P18583 | 52 | GC21P033542 |
| CCND1 | Cyclin D1 | Protein Coding | P24385 | 65 | GC11P069641 |
| AMHR2 | Anti-Mullerian Hormone Receptor Type 2 | Protein Coding | Q16671 | 59 | GC12P053423 |
| CTNNB1 | Catenin Beta 1 | Protein Coding | P35222 | 66 | GC03P041194 |
| TRIM16 | Tripartite Motif Containing 16 | Protein Coding | O95361 | 39 | GC17M015627 |
| TGFBR1 | Transforming Growth Factor Beta Receptor 1 | Protein Coding | P36897 | 66 | GC09P109923 |
| PCMT1 | Protein-L-Isoaspartate (D-Aspartate) O-Methyltransferase | Protein Coding | P22061 | 51 | GC06P149749 |
| NUAK1 | NUAK Family Kinase 1 | Protein Coding | O60285 | 52 | GC12M106063 |
| SOD2 | Superoxide Dismutase 2 | Protein Coding | P04179 | 60 | GC06M159669 |
| KDM5A | Lysine Demethylase 5A | Protein Coding | P29375 | 56 | GC12M000280 |
| GH1 | Growth Hormone 1 | Protein Coding | P01241 | 56 | GC17M063917 |
| STAT3 | Signal Transducer And Activator Of Transcription 3 | Protein Coding | P40763 | 66 | GC17M042313 |
| CEBPB | CCAAT Enhancer Binding Protein Beta | Protein Coding | P17676 | 58 | GC20P050190 |
| TGFA | Transforming Growth Factor Alpha | Protein Coding | P01135 | 59 | GC02M070447 |
| GMNN | Geminin DNA Replication Inhibitor | Protein Coding | O75496 | 57 | GC06P024779 |
| DCLK1 | Doublecortin Like Kinase 1 | Protein Coding | O15075 | 59 | GC13M035768 |
| ACTA2 | Actin Alpha 2, Smooth Muscle | Protein Coding | P62736 | 60 | GC10M088935 |
| GATA6 | GATA Binding Protein 6 | Protein Coding | Q92908 | 60 | GC18P022169 |
| SNAI2 | Snail Family Transcriptional Repressor 2 | Protein Coding | O43623 | 55 | GC08M048917 |
| CEBPA | CCAAT Enhancer Binding Protein Alpha | Protein Coding | P49715 | 60 | GC19M033299 |
| TERT | Telomerase Reverse Transcriptase | Protein Coding | O14746 | 64 | GC05M001253 |
| PCDH9 | Protocadherin 9 | Protein Coding | Q9HC56 | 53 | GC13M066302 |
| CLDN1 | Claudin 1 | Protein Coding | O95832 | 59 | GC03M190305 |
| STK33 | Serine/Threonine Kinase 33 | Protein Coding | Q9BYT3 | 53 | GC11M016118 |
| RAF1 | Raf-1 Proto-Oncogene, Serine/Threonine Kinase | Protein Coding | P04049 | 67 | GC03M012583 |
| GATA1 | GATA Binding Protein 1 | Protein Coding | P15976 | 59 | GC0XP048786 |
| HIF1A | Hypoxia Inducible Factor 1 Subunit Alpha | Protein Coding | Q16665 | 62 | GC14P061695 |
| HOXB13 | Homeobox B13 | Protein Coding | Q92826 | 55 | GC17M101298 |
| PBX3 | PBX Homeobox 3 | Protein Coding | P40426 | 50 | GC09P125747 |
| RUNX2 | RUNX Family Transcription Factor 2 | Protein Coding | Q13950 | 60 | GC06P190902 |
| SLIT3 | Slit Guidance Ligand 3 | Protein Coding | O75094 | 55 | GC05M168661 |
| PTX3 | Pentraxin 3 | Protein Coding | P26022 | 55 | GC03P157436 |
| GAPDH | Glyceraldehyde-3-Phosphate Dehydrogenase | Protein Coding | P04406 | 64 | GC12P080272 |
| AKT1 | AKT Serine/Threonine Kinase 1 | Protein Coding | P31749 | 66 | GC14M104769 |
| TACC3 | Transforming Acidic Coiled-Coil Containing Protein 3 | Protein Coding | Q9Y6A5 | 55 | GC04P031616 |
| PARD6A | Par-6 Family Cell Polarity Regulator Alpha | Protein Coding | Q9NPB6 | 50 | GC16P129773 |
| TET1 | Tet Methylcytosine Dioxygenase 1 | Protein Coding | Q8NFU7 | 52 | GC10P068560 |
| SLC2A1 | Solute Carrier Family 2 Member 1 | Protein Coding | P11166 | 66 | GC01M042925 |
| CIP2A | Cellular Inhibitor Of PP2A | Protein Coding | Q8TCG1 | 48 | GC03M108619 |
| SIN3A | SIN3 Transcription Regulator Family Member A | Protein Coding | Q96ST3 | 59 | GC15M075369 |
| CDX2 | Caudal Type Homeobox 2 | Protein Coding | Q99626 | 55 | GC13M027962 |
| TCF4 | Transcription Factor 4 | Protein Coding | P15884 | 59 | GC18M055222 |
| NEUROG3 | Neurogenin 3 | Protein Coding | Q9Y4Z2 | 50 | GC10M069571 |
| TP73 | Tumor Protein P73 | Protein Coding | O15350 | 57 | GC01P003652 |
| SKP1 | S-Phase Kinase Associated Protein 1 | Protein Coding | P63208 | 56 | GC05M134148 |
| MTA3 | Metastasis Associated 1 Family Member 3 | Protein Coding | Q9BTC8 | 53 | GC02P042494 |
| LETMD1 | LETM1 Domain Containing 1 | Protein Coding | Q6P1Q0 | 48 | GC12P051047 |
| ZEB2 | Zinc Finger E-Box Binding Homeobox 2 | Protein Coding | O60315 | 61 | GC02M144384 |
| FAM3C | FAM3 Metabolism Regulating Signaling Molecule C | Protein Coding | Q92520 | 50 | GC07M121382 |
| BSG | Basigin (Ok Blood Group) | Protein Coding | P35613 | 57 | GC19P000571 |
| IL22 | Interleukin 22 | Protein Coding | Q9GZX6 | 55 | GC12M068248 |
| THBD | Thrombomodulin | Protein Coding | P07204 | 58 | GC20M023026 |
| SKI | SKI Proto-Oncogene | Protein Coding | P12755 | 57 | GC01P082947 |
| BRMS1 | BRMS1 Transcriptional Repressor And Anoikis Regulator | Protein Coding | Q9HCU9 | 49 | GC11M143029 |
| SMAD3 | SMAD Family Member 3 | Protein Coding | P84022 | 66 | GC15P067063 |
| CDKN2A | Cyclin Dependent Kinase Inhibitor 2A | Protein Coding | Q8N726 | 63 | GC09M021967 |
| SPRR2A | Small Proline Rich Protein 2A | Protein Coding | P35326 | 43 | GC01M168536 |
| PRDX1 | Peroxiredoxin 1 | Protein Coding | Q06830 | 62 | GC01M045943 |
| TGM2 | Transglutaminase 2 | Protein Coding | P21980 | 60 | GC20M038127 |
| ST8SIA1 | ST8 Alpha-N-Acetyl-Neuraminide Alpha-2,8-Sialyltransferase 1 | Protein Coding | Q92185 | 53 | GC12M022063 |
| ARMC8 | Armadillo Repeat Containing 8 | Protein Coding | Q8IUR7 | 44 | GC03P138187 |
| EGFR | Epidermal Growth Factor Receptor | Protein Coding | P00533 | 68 | GC07P055019 |
| CDK5 | Cyclin Dependent Kinase 5 | Protein Coding | Q00535 | 66 | GC07M151053 |
| SPP1 | Secreted Phosphoprotein 1 | Protein Coding | P10451 | 57 | GC04P087975 |
| TGFBR3 | Transforming Growth Factor Beta Receptor 3 | Protein Coding | Q03167 | 60 | GC01M091680 |
| ST6GAL1 | ST6 Beta-Galactoside Alpha-2,6-Sialyltransferase 1 | Protein Coding | P15907 | 56 | GC03P186930 |
| TXN2 | Thioredoxin 2 | Protein Coding | Q99757 | 55 | GC22M036467 |
| SKIL | SKI Like Proto-Oncogene | Protein Coding | P12757 | 55 | GC03P170357 |
| OPRM1 | Opioid Receptor Mu 1 | Protein Coding | P35372 | 62 | GC06P192519 |
| TGFBR2 | Transforming Growth Factor Beta Receptor 2 | Protein Coding | P37173 | 65 | GC03P030610 |
| SIAH2 | Siah E3 Ubiquitin Protein Ligase 2 | Protein Coding | O43255 | 55 | GC03M150741 |
| SPDEF | SAM Pointed Domain Containing ETS Transcription Factor | Protein Coding | O95238 | 50 | GC06M111697 |
| SMAD2 | SMAD Family Member 2 | Protein Coding | Q15796 | 65 | GC18M047809 |
| SMURF2 | SMAD Specific E3 Ubiquitin Protein Ligase 2 | Protein Coding | Q9HAU4 | 55 | GC17M064542 |
| TGFB3 | Transforming Growth Factor Beta 3 | Protein Coding | P10600 | 60 | GC14M075958 |
| SIAH1 | Siah E3 Ubiquitin Protein Ligase 1 | Protein Coding | Q8IUQ4 | 58 | GC16M054276 |
| PTHLH | Parathyroid Hormone Like Hormone | Protein Coding | P12272 | 57 | GC12M037271 |
| FSCN2 | Fascin Actin-Bundling Protein 2, Retinal | Protein Coding | O14926 | 50 | GC17P081515 |
| SFRP2 | Secreted Frizzled Related Protein 2 | Protein Coding | Q96HF1 | 53 | GC04M153780 |
| STIM1 | Stromal Interaction Molecule 1 | Protein Coding | Q13586 | 62 | GC11P022826 |
| SHC1 | SHC Adaptor Protein 1 | Protein Coding | P29353 | 57 | GC01M154962 |
| PBXIP1 | PBX Homeobox Interacting Protein 1 | Protein Coding | Q96AQ6 | 46 | GC01M154944 |
| CBY1 | Chibby 1, Beta Catenin Antagonist | Protein Coding | Q9Y3M2 | 52 | GC22P038656 |
| NOTCH1 | Notch Receptor 1 | Protein Coding | P46531 | 66 | GC09M139438 |
| CXCL5 | C-X-C Motif Chemokine Ligand 5 | Protein Coding | P42830 | 53 | GC04M073995 |
| SRF | Serum Response Factor | Protein Coding | P11831 | 56 | GC06P043171 |
| CPEB1 | Cytoplasmic Polyadenylation Element Binding Protein 1 | Protein Coding | Q9BZB8 | 53 | GC15M082543 |
| PAK5 | P21 (RAC1) Activated Kinase 5 | Protein Coding | Q9P286 | 56 | GC20M010928 |
| CERS6 | Ceramide Synthase 6 | Protein Coding | Q6ZMG9 | 51 | GC02P168491 |
| CCL5 | C-C Motif Chemokine Ligand 5 | Protein Coding | P13501 | 56 | GC17M035871 |
| NUCB2 | Nucleobindin 2 | Protein Coding | P80303 | 50 | GC11P023500 |
| BRCA1 | BRCA1 DNA Repair Associated | Protein Coding | P38398 | 63 | GC17M043044 |
| AKTIP | AKT Interacting Protein | Protein Coding | Q9H8T0 | 46 | GC16M055056 |
| CTNNBIP1 | Catenin Beta Interacting Protein 1 | Protein Coding | Q9NSA3 | 53 | GC01M009848 |
| NR5A2 | Nuclear Receptor Subfamily 5 Group A Member 2 | Protein Coding | O00482 | 57 | GC01P199996 |
| ILK | Integrin Linked Kinase | Protein Coding | Q13418 | 59 | GC11P022985 |
| FGF19 | Fibroblast Growth Factor 19 | Protein Coding | O95750 | 57 | GC11M143141 |
| BDNF | Brain Derived Neurotrophic Factor | Protein Coding | P23560 | 62 | GC11M027654 |
| ROR1 | Receptor Tyrosine Kinase Like Orphan Receptor 1 | Protein Coding | Q01973 | 56 | GC01P063774 |
| SPARC | Secreted Protein Acidic And Cysteine Rich | Protein Coding | P09486 | 62 | GC05M151661 |
| SFTPC | Surfactant Protein C | Protein Coding | P11686 | 52 | GC08P022156 |
| KLK6 | Kallikrein Related Peptidase 6 | Protein Coding | Q92876 | 57 | GC19M050958 |
| FSHR | Follicle Stimulating Hormone Receptor | Protein Coding | P23945 | 59 | GC02M048962 |
| GSK3B | Glycogen Synthase Kinase 3 Beta | Protein Coding | P49841 | 64 | GC03M119821 |
| RBM8A | RNA Binding Motif Protein 8A | Protein Coding | Q9Y5S9 | 53 | GC01M145921 |
| BCL2 | BCL2 Apoptosis Regulator | Protein Coding | P10415 | 64 | GC18M063123 |
| NTRK3 | Neurotrophic Receptor Tyrosine Kinase 3 | Protein Coding | Q16288 | 66 | GC15M087859 |
| SOX9 | SRY-Box Transcription Factor 9 | Protein Coding | P48436 | 57 | GC17P072121 |
| SOX17 | SRY-Box Transcription Factor 17 | Protein Coding | Q9H6I2 | 55 | GC08P054457 |
| PRSS8 | Serine Protease 8 | Protein Coding | Q16651 | 57 | GC16M053902 |
| IL27 | Interleukin 27 | Protein Coding | Q8NEV9 | 49 | GC16M053079 |
| NFKB1 | Nuclear Factor Kappa B Subunit 1 | Protein Coding | P19838 | 66 | GC04P102501 |
| MRC2 | Mannose Receptor C-Type 2 | Protein Coding | Q9UBG0 | 53 | GC17P062627 |
| RB1 | RB Transcriptional Corepressor 1 | Protein Coding | P06400 | 62 | GC13P048303 |
| NPPA | Natriuretic Peptide A | Protein Coding | P01160 | 57 | GC01M036050 |
| SOX4 | SRY-Box Transcription Factor 4 | Protein Coding | Q06945 | 56 | GC06P021593 |
| SEMA4A | Semaphorin 4A | Protein Coding | Q9H3S1 | 55 | GC01P156147 |
| PROP1 | PROP Paired-Like Homeobox 1 | Protein Coding | O75360 | 50 | GC05M177992 |
| ZMYND8 | Zinc Finger MYND-Type Containing 8 | Protein Coding | Q9ULU4 | 52 | GC20M047209 |
| IL6 | Interleukin 6 | Protein Coding | P05231 | 63 | GC07P022725 |
| GAB2 | GRB2 Associated Binding Protein 2 | Protein Coding | Q9UQC2 | 55 | GC11M078215 |
| PTPN11 | Protein Tyrosine Phosphatase Non-Receptor Type 11 | Protein Coding | Q06124 | 67 | GC12P112418 |
| FSCN1 | Fascin Actin-Bundling Protein 1 | Protein Coding | Q16658 | 57 | GC07P005592 |
| ELSPBP1 | Epididymal Sperm Binding Protein 1 | Protein Coding | Q96BH3 | 40 | GC19P047994 |
| MAP2K1 | Mitogen-Activated Protein Kinase Kinase 1 | Protein Coding | Q02750 | 67 | GC15P066386 |
| CCNDBP1 | Cyclin D1 Binding Protein 1 | Protein Coding | O95273 | 47 | GC15P043185 |
| HMGA2 | High Mobility Group AT-Hook 2 | Protein Coding | P52926 | 57 | GC12P065824 |
| CD151 | CD151 Molecule (Raph Blood Group) | Protein Coding | P48509 | 55 | GC11P022706 |
| NOTCH4 | Notch Receptor 4 | Protein Coding | Q99466 | 60 | GC06M111570 |
| SIX1 | SIX Homeobox 1 | Protein Coding | Q15475 | 55 | GC14M060643 |
| GOLPH3 | Golgi Phosphoprotein 3 | Protein Coding | Q9H4A6 | 52 | GC05M032124 |
| ERBIN | Erbb2 Interacting Protein | Protein Coding | Q96RT1 | 52 | GC05P065884 |
| PADI4 | Peptidyl Arginine Deiminase 4 | Protein Coding | Q9UM07 | 58 | GC01P017308 |
| CXCR4 | C-X-C Motif Chemokine Receptor 4 | Protein Coding | P61073 | 65 | GC02M136114 |
| HDAC9 | Histone Deacetylase 9 | Protein Coding | Q9UKV0 | 63 | GC07P018086 |
| NOTCH3 | Notch Receptor 3 | Protein Coding | Q9UM47 | 64 | GC19M015159 |
| SDC1 | Syndecan 1 | Protein Coding | P18827 | 56 | GC02M020200 |
| CX3CL1 | C-X3-C Motif Chemokine Ligand 1 | Protein Coding | P78423 | 57 | GC16P057372 |
| BTBD7 | BTB Domain Containing 7 | Protein Coding | Q9P203 | 40 | GC14M093237 |
| MMD | Monocyte To Macrophage Differentiation Associated | Protein Coding | Q15546 | 40 | GC17M055392 |
| TP53 | Tumor Protein P53 | Protein Coding | P04637 | 66 | GC17M007661 |
| SEMA3E | Semaphorin 3E | Protein Coding | O15041 | 56 | GC07M083363 |
| CEMIP | Cell Migration Inducing Hyaluronidase 1 | Protein Coding | Q8WUJ3 | 48 | GC15P080779 |
| NME1 | NME/NM23 Nucleoside Diphosphate Kinase 1 | Protein Coding | P15531 | 60 | GC17P165542 |
| SATB1 | SATB Homeobox 1 | Protein Coding | Q01826 | 56 | GC03M029344 |
| CCL25 | C-C Motif Chemokine Ligand 25 | Protein Coding | O15444 | 49 | GC19P008052 |
| PRKAA1 | Protein Kinase AMP-Activated Catalytic Subunit Alpha 1 | Protein Coding | Q13131 | 59 | GC05M040759 |
| SCRIB | Scribble Planar Cell Polarity Protein | Protein Coding | C0HLS1 | 52 | GC08M147554 |
| EGF | Epidermal Growth Factor | Protein Coding | P01133 | 64 | GC04P109912 |
| VGLL4 | Vestigial Like Family Member 4 | Protein Coding | Q14135 | 43 | GC03M012217 |
| SALL4 | Spalt Like Transcription Factor 4 | Protein Coding | Q9UJQ4 | 56 | GC20M051784 |
| NKX6-1 | NK6 Homeobox 1 | Protein Coding | P78426 | 52 | GC04M084491 |
| BCL2L1 | BCL2 Like 1 | Protein Coding | Q07817 | 63 | GC20M031664 |
| CCL22 | C-C Motif Chemokine Ligand 22 | Protein Coding | O00626 | 49 | GC16P129511 |
| TRIM62 | Tripartite Motif Containing 62 | Protein Coding | Q9BVG3 | 44 | GC01M037615 |
| SUZ12 | SUZ12 Polycomb Repressive Complex 2 Subunit | Protein Coding | Q15022 | 55 | GC17P031937 |
| MTDH | Metadherin | Protein Coding | Q86UE4 | 52 | GC08P097644 |
| CD63 | CD63 Molecule | Protein Coding | P08962 | 57 | GC12M060634 |
| TMPRSS4 | Transmembrane Serine Protease 4 | Protein Coding | Q9NRS4 | 52 | GC11P118077 |
| NFIL3 | Nuclear Factor, Interleukin 3 Regulated | Protein Coding | Q16649 | 47 | GC09M126468 |
| OVOL2 | Ovo Like Zinc Finger 2 | Protein Coding | Q9BRP0 | 46 | GC20M017956 |
| CCL3 | C-C Motif Chemokine Ligand 3 | Protein Coding | P10147 | 54 | GC17M036088 |
| RNF111 | Ring Finger Protein 111 | Protein Coding | Q6ZNA4 | 48 | GC15P202034 |
| NCSTN | Nicastrin | Protein Coding | Q92542 | 62 | GC01P160343 |
| MAPK1 | Mitogen-Activated Protein Kinase 1 | Protein Coding | P28482 | 66 | GC22M021759 |
| MICAL2 | Microtubule Associated Monooxygenase, Calponin And LIM Domain Containing 2 | Protein Coding | O94851 | 50 | GC11P023248 |
| MAPK8 | Mitogen-Activated Protein Kinase 8 | Protein Coding | P45983 | 63 | GC10P048306 |
| NFIC | Nuclear Factor I C | Protein Coding | P08651 | 52 | GC19P003314 |
| SERPINB4 | Serpin Family B Member 4 | Protein Coding | P48594 | 49 | GC18M063637 |
| IL17RD | Interleukin 17 Receptor D | Protein Coding | Q8NFM7 | 56 | GC03M057089 |
| ARHGEF12 | Rho Guanine Nucleotide Exchange Factor 12 | Protein Coding | Q9NZN5 | 53 | GC11P120336 |
| CD44 | CD44 Molecule (IN Blood Group) | Protein Coding | P16070 | 62 | GC11P035139 |
| ISG15 | ISG15 Ubiquitin Like Modifier | Protein Coding | P05161 | 59 | GC01P001001 |
| PRKCA | Protein Kinase C Alpha | Protein Coding | P17252 | 65 | GC17P066302 |
| MYL2 | Myosin Light Chain 2 | Protein Coding | P10916 | 61 | GC12M112099 |
| NDRG2 | NDRG Family Member 2 | Protein Coding | Q9UN36 | 50 | GC14M021016 |
| ATXN1 | Ataxin 1 | Protein Coding | P54253 | 56 | GC06M016299 |
| PLAUR | Plasminogen Activator, Urokinase Receptor | Protein Coding | Q03405 | 57 | GC19M043646 |
| NEDD4L | NEDD4 Like E3 Ubiquitin Protein Ligase | Protein Coding | Q96PU5 | 57 | GC18P058044 |
| BMI1 | BMI1 Proto-Oncogene, Polycomb Ring Finger | Protein Coding | P35226 | 57 | GC10P022422 |
| PITPNM1 | Phosphatidylinositol Transfer Protein Membrane Associated 1 | Protein Coding | O00562 | 50 | GC11M143075 |
| MYD88 | MYD88 Innate Immune Signal Transduction Adaptor | Protein Coding | Q99836 | 61 | GC03P042445 |
| EIF5A2 | Eukaryotic Translation Initiation Factor 5A2 | Protein Coding | Q9GZV4 | 50 | GC03M170888 |
| SALL1 | Spalt Like Transcription Factor 1 | Protein Coding | Q9NSC2 | 56 | GC16M051135 |
| PKD1 | Polycystin 1, Transient Receptor Potential Channel Interacting | Protein Coding | P98161 | 58 | GC16M052152 |
| FMR1 | Fragile X Messenger Ribonucleoprotein 1 | Protein Coding | Q06787 | 57 | GC0XP148000 |
| PTEN | Phosphatase And Tensin Homolog | Protein Coding | P60484 | 65 | GC10P128607 |
| CXCL14 | C-X-C Motif Chemokine Ligand 14 | Protein Coding | O95715 | 48 | GC05M135617 |
| TUG1 | Taurine Up-Regulated 1 | Protein Coding | A0A6I8PU40 | 33 | GC22P030969 |
| MYCN | MYCN Proto-Oncogene, BHLH Transcription Factor | Protein Coding | P04198 | 59 | GC02P016028 |
| PARD3 | Par-3 Family Cell Polarity Regulator | Protein Coding | Q8TEW0 | 55 | GC10M037534 |
| S100P | S100 Calcium Binding Protein P | Protein Coding | P25815 | 51 | GC04P031866 |
| CDK14 | Cyclin Dependent Kinase 14 | Protein Coding | O94921 | 49 | GC07P098916 |
| EXOC7 | Exocyst Complex Component 7 | Protein Coding | Q9UPT5 | 55 | GC17M101840 |
| MTOR | Mechanistic Target Of Rapamycin Kinase | Protein Coding | P42345 | 68 | GC01M011106 |
| ONECUT2 | One Cut Homeobox 2 | Protein Coding | O95948 | 46 | GC18P057450 |
| SEMA4C | Semaphorin 4C | Protein Coding | Q9C0C4 | 44 | GC02M096859 |
| MYBL2 | MYB Proto-Oncogene Like 2 | Protein Coding | P10244 | 55 | GC20P043667 |
| PRKCI | Protein Kinase C Iota | Protein Coding | P41743 | 59 | GC03P170222 |
| S100A9 | S100 Calcium Binding Protein A9 | Protein Coding | P06702 | 56 | GC01P153357 |
| PFN2 | Profilin 2 | Protein Coding | P35080 | 52 | GC03M149964 |
| FLT1 | Fms Related Receptor Tyrosine Kinase 1 | Protein Coding | P17948 | 64 | GC13M028300 |
| VIM | Vimentin | Protein Coding | P08670 | 63 | GC10P017227 |
| ROCK2 | Rho Associated Coiled-Coil Containing Protein Kinase 2 | Protein Coding | O75116 | 58 | GC02M011635 |
| PIN1 | Peptidylprolyl Cis/Trans Isomerase, NIMA-Interacting 1 | Protein Coding | Q13526 | 60 | GC19P009835 |
| MYB | MYB Proto-Oncogene, Transcription Factor | Protein Coding | P10242 | 62 | GC06P135181 |
| AXL | AXL Receptor Tyrosine Kinase | Protein Coding | P30530 | 65 | GC19P041219 |
| S100A8 | S100 Calcium Binding Protein A8 | Protein Coding | P05109 | 55 | GC01M168551 |
| SUFU | SUFU Negative Regulator Of Hedgehog Signaling | Protein Coding | Q9UMX1 | 55 | GC10P128980 |
| FLNA | Filamin A | Protein Coding | P21333 | 62 | GC0XM154348 |
| RAC1 | Rac Family Small GTPase 1 | Protein Coding | P63000 | 62 | GC07P021715 |
| RASAL2 | RAS Protein Activator Like 2 | Protein Coding | Q9UJF2 | 50 | GC01P180856 |
| PGF | Placental Growth Factor | Protein Coding | P49763 | 56 | GC14M074941 |
| TRIM37 | Tripartite Motif Containing 37 | Protein Coding | O94972 | 53 | GC17M101477 |
| FBXW7 | F-Box And WD Repeat Domain Containing 7 | Protein Coding | Q969H0 | 59 | GC04M152321 |
| S100A6 | S100 Calcium Binding Protein A6 | Protein Coding | P06703 | 55 | GC01M168562 |
| IL23A | Interleukin 23 Subunit Alpha | Protein Coding | Q9NPF7 | 50 | GC12P081650 |
| KDM6B | Lysine Demethylase 6B | Protein Coding | O15054 | 57 | GC17P007834 |
| MMP9 | Matrix Metallopeptidase 9 | Protein Coding | P14780 | 66 | GC20P046008 |
| EIF2AK3 | Eukaryotic Translation Initiation Factor 2 Alpha Kinase 3 | Protein Coding | Q9NZJ5 | 63 | GC02M088556 |
| SERPINF1 | Serpin Family F Member 1 | Protein Coding | P36955 | 57 | GC17P163867 |
| MUC2 | Mucin 2, Oligomeric Mucus/Gel-Forming | Protein Coding | Q02817 | 52 | GC11P001074 |
| TRIM33 | Tripartite Motif Containing 33 | Protein Coding | Q9UPN9 | 55 | GC01M114392 |
| S100A2 | S100 Calcium Binding Protein A2 | Protein Coding | P29034 | 54 | GC01M153561 |
| MAGEC2 | MAGE Family Member C2 | Protein Coding | Q9UBF1 | 45 | GC0XM142202 |
| PLXND1 | Plexin D1 | Protein Coding | Q9Y4D7 | 55 | GC03M129555 |
| MMP2 | Matrix Metallopeptidase 2 | Protein Coding | P08253 | 66 | GC16P129449 |
| MAP4K4 | Mitogen-Activated Protein Kinase Kinase Kinase Kinase 4 | Protein Coding | O95819 | 59 | GC02P101696 |
| GOLM1 | Golgi Membrane Protein 1 | Protein Coding | Q8NBJ4 | 52 | GC09M086026 |
| MST1R | Macrophage Stimulating 1 Receptor | Protein Coding | Q04912 | 62 | GC03M055066 |
| PAK1 | P21 (RAC1) Activated Kinase 1 | Protein Coding | Q13153 | 63 | GC11M143344 |
| ROS1 | ROS Proto-Oncogene 1, Receptor Tyrosine Kinase | Protein Coding | P08922 | 60 | GC06M117287 |
| HOOK1 | Hook Microtubule Tethering Protein 1 | Protein Coding | Q9UJC3 | 50 | GC01P059814 |
| PEG10 | Paternally Expressed 10 | Protein Coding | Q86TG7 | 53 | GC07P094656 |
| GLI1 | GLI Family Zinc Finger 1 | Protein Coding | P08151 | 60 | GC12P081689 |
| QKI | QKI, KH Domain Containing RNA Binding | Protein Coding | Q96PU8 | 55 | GC06P163414 |
| PAWR | Pro-Apoptotic WT1 Regulator | Protein Coding | Q96IZ0 | 54 | GC12M079574 |
| MSN | Moesin | Protein Coding | P26038 | 60 | GC0XP065588 |
| OSM | Oncostatin M | Protein Coding | P13725 | 56 | GC22M030262 |
| RORC | RAR Related Orphan Receptor C | Protein Coding | P51449 | 56 | GC01M151806 |
| PDCD1 | Programmed Cell Death 1 | Protein Coding | Q15116 | 60 | GC02M241849 |
| SNW1 | SNW Domain Containing 1 | Protein Coding | Q13573 | 54 | GC14M077717 |
| MET | MET Proto-Oncogene, Receptor Tyrosine Kinase | Protein Coding | P08581 | 67 | GC07P116672 |
| HAND1 | Heart And Neural Crest Derivatives Expressed 1 | Protein Coding | O96004 | 53 | GC05M154475 |
| ROR2 | Receptor Tyrosine Kinase Like Orphan Receptor 2 | Protein Coding | Q01974 | 60 | GC09M126471 |
| EXOC4 | Exocyst Complex Component 4 | Protein Coding | Q96A65 | 53 | GC07P133253 |
| CRIM1 | Cysteine Rich Transmembrane BMP Regulator 1 | Protein Coding | Q9NZV1 | 46 | GC02P036355 |
| ALDOB | Aldolase, Fructose-Bisphosphate B | Protein Coding | P05062 | 59 | GC09M101420 |
| SMAD4 | SMAD Family Member 4 | Protein Coding | Q13485 | 66 | GC18P051028 |
| CYP7B1 | Cytochrome P450 Family 7 Subfamily B Member 1 | Protein Coding | O75881 | 58 | GC08M064587 |
| NOTCH2 | Notch Receptor 2 | Protein Coding | Q04721 | 65 | GC01M119911 |
| BCL6 | BCL6 Transcription Repressor | Protein Coding | P41182 | 59 | GC03M187721 |
| EGFL7 | EGF Like Domain Multiple 7 | Protein Coding | Q9UHF1 | 48 | GC09P136658 |
| CEP164 | Centrosomal Protein 164 | Protein Coding | Q9UPV0 | 53 | GC11P117314 |
| KLF4 | KLF Transcription Factor 4 | Protein Coding | O43474 | 60 | GC09M107484 |
| MUC16 | Mucin 16, Cell Surface Associated | Protein Coding | Q8WXI7 | 52 | GC19M008848 |
| NODAL | Nodal Growth Differentiation Factor | Protein Coding | Q96S42 | 54 | GC10M070431 |
| MSX2 | Msh Homeobox 2 | Protein Coding | P35548 | 57 | GC05P174724 |
| RGS3 | Regulator Of G Protein Signaling 3 | Protein Coding | P49796 | 48 | GC09P155477 |
| PCBP1 | Poly(RC) Binding Protein 1 | Protein Coding | Q15365 | 55 | GC02P070087 |
| FKBP5 | FKBP Prolyl Isomerase 5 | Protein Coding | Q13451 | 62 | GC06M111704 |
| WNT5A | Wnt Family Member 5A | Protein Coding | P41221 | 63 | GC03M055465 |
| FOXP2 | Forkhead Box P2 | Protein Coding | O15409 | 56 | GC07P114086 |
| ATM | ATM Serine/Threonine Kinase | Protein Coding | Q13315 | 66 | GC11P108222 |
| MMP19 | Matrix Metallopeptidase 19 | Protein Coding | Q99542 | 59 | GC12M055835 |
| MMP14 | Matrix Metallopeptidase 14 | Protein Coding | P50281 | 64 | GC14P060441 |
| RDX | Radixin | Protein Coding | P35241 | 61 | GC11M109864 |
| F11R | F11 Receptor | Protein Coding | Q9Y624 | 54 | GC01M160995 |
| PHLDA1 | Pleckstrin Homology Like Domain Family A Member 1 | Protein Coding | Q8WV24 | 46 | GC12M076025 |
| MYC | MYC Proto-Oncogene, BHLH Transcription Factor | Protein Coding | P01106 | 65 | GC08P127735 |
| ZFYVE9 | Zinc Finger FYVE-Type Containing 9 | Protein Coding | O95405 | 55 | GC01P052142 |
| MUC4 | Mucin 4, Cell Surface Associated | Protein Coding | Q99102 | 52 | GC03M195746 |
| MMP13 | Matrix Metallopeptidase 13 | Protein Coding | P45452 | 62 | GC11M102942 |
| SERPINE1 | Serpin Family E Member 1 | Protein Coding | P05121 | 63 | GC07P101127 |
| XRN2 | 5'-3' Exoribonuclease 2 | Protein Coding | Q9H0D6 | 53 | GC20P021303 |
| SMAD7 | SMAD Family Member 7 | Protein Coding | O15105 | 56 | GC18M048919 |
| MMP7 | Matrix Metallopeptidase 7 | Protein Coding | P09237 | 60 | GC11M143590 |
| MMP11 | Matrix Metallopeptidase 11 | Protein Coding | P24347 | 57 | GC22P023768 |
| RBP2 | Retinol Binding Protein 2 | Protein Coding | P50120 | 50 | GC03M139452 |
| FURIN | Furin, Paired Basic Amino Acid Cleaving Enzyme | Protein Coding | P09958 | 61 | GC15P090868 |
| FHL2 | Four And A Half LIM Domains 2 | Protein Coding | Q14192 | 58 | GC02M105357 |
| SIRT1 | Sirtuin 1 | Protein Coding | Q96EB6 | 62 | GC10P067884 |
| KL | Klotho | Protein Coding | Q9UEF7 | 60 | GC13P033016 |
| FOXO4 | Forkhead Box O4 | Protein Coding | P98177 | 55 | GC0XP071095 |
| MMP8 | Matrix Metallopeptidase 8 | Protein Coding | P22894 | 60 | GC11M143591 |
| SENP2 | SUMO Specific Peptidase 2 | Protein Coding | Q9HC62 | 46 | GC03P185582 |
| PEBP1 | Phosphatidylethanolamine Binding Protein 1 | Protein Coding | P30086 | 59 | GC12P118218 |
| FHL1 | Four And A Half LIM Domains 1 | Protein Coding | Q13642 | 59 | GC0XP136146 |
| LHX2 | LIM Homeobox 2 | Protein Coding | P50458 | 50 | GC09P124001 |
| MAP3K4 | Mitogen-Activated Protein Kinase Kinase Kinase 4 | Protein Coding | Q9Y6R4 | 54 | GC06P160991 |
| MLLT3 | MLLT3 Super Elongation Complex Subunit | Protein Coding | P42568 | 51 | GC09M020341 |
| NTN4 | Netrin 4 | Protein Coding | Q9HB63 | 52 | GC12M095657 |
| P2RY2 | Purinergic Receptor P2Y2 | Protein Coding | P41231 | 57 | GC11P115400 |
| SCUBE3 | Signal Peptide, CUB Domain And EGF Like Domain Containing 3 | Protein Coding | Q8IX30 | 53 | GC06P190815 |
| CCR7 | C-C Motif Chemokine Receptor 7 | Protein Coding | P32248 | 57 | GC17M100916 |
| IGSF8 | Immunoglobulin Superfamily Member 8 | Protein Coding | Q969P0 | 47 | GC01M168796 |
| MAP3K3 | Mitogen-Activated Protein Kinase Kinase Kinase 3 | Protein Coding | Q99759 | 59 | GC17P063622 |
| ASCL2 | Achaete-Scute Family BHLH Transcription Factor 2 | Protein Coding | Q99929 | 45 | GC11M015868 |
| RASA1 | RAS P21 Protein Activator 1 | Protein Coding | P20936 | 60 | GC05P087267 |
| NFKBIA | NFKB Inhibitor Alpha | Protein Coding | P25963 | 63 | GC14M035401 |
| FCN2 | Ficolin 2 | Protein Coding | Q15485 | 57 | GC09P134864 |
| PROM1 | Prominin 1 | Protein Coding | O43490 | 60 | GC04M015965 |
| NREP | Neuronal Regeneration Related Protein | Protein Coding | Q16612 | 42 | GC05M111662 |
| MDM2 | MDM2 Proto-Oncogene | Protein Coding | Q00987 | 66 | GC12P068808 |
| MITF | Melanocyte Inducing Transcription Factor | Protein Coding | O75030 | 61 | GC03P069740 |
| PRUNE1 | Prune Exopolyphosphatase 1 | Protein Coding | Q86TP1 | 52 | GC01P151008 |
| NF1 | Neurofibromin 1 | Protein Coding | P21359 | 60 | GC17P031094 |
| TGFB2 | Transforming Growth Factor Beta 2 | Protein Coding | P61812 | 63 | GC01P218345 |
| CD14 | CD14 Molecule | Protein Coding | P08571 | 58 | GC05M140631 |
| CXCL9 | C-X-C Motif Chemokine Ligand 9 | Protein Coding | Q07325 | 52 | GC04M076001 |
| CXCL16 | C-X-C Motif Chemokine Ligand 16 | Protein Coding | Q9H2A7 | 50 | GC17M004733 |
| NCL | Nucleolin | Protein Coding | P19338 | 57 | GC02M231453 |
| FBP1 | Fructose-Bisphosphatase 1 | Protein Coding | P09467 | 62 | GC09M094603 |
| PIK3CA | Phosphatidylinositol-4,5-Bisphosphate 3-Kinase Catalytic Subunit Alpha | Protein Coding | P42336 | 65 | GC03P179148 |
| NOG | Noggin | Protein Coding | Q13253 | 59 | GC17P056593 |
| MEF2D | Myocyte Enhancer Factor 2D | Protein Coding | Q14814 | 57 | GC01M156463 |
| PTPRZ1 | Protein Tyrosine Phosphatase Receptor Type Z1 | Protein Coding | P23471 | 59 | GC07P121873 |
| CEACAM6 | CEA Cell Adhesion Molecule 6 | Protein Coding | P40199 | 53 | GC19P041750 |
| FASN | Fatty Acid Synthase | Protein Coding | P49327 | 62 | GC17M082078 |
| LRRFIP1 | LRR Binding FLII Interacting Protein 1 | Protein Coding | Q32MZ4 | 47 | GC02P237627 |
| MDM4 | MDM4 Regulator Of P53 | Protein Coding | O15151 | 59 | GC01P204516 |
| NKX3-2 | NK3 Homeobox 2 | Protein Coding | P78367 | 48 | GC04M013542 |
| FBLN1 | Fibulin 1 | Protein Coding | P23142 | 56 | GC22P045502 |
| RHOA | Ras Homolog Family Member A | Protein Coding | P61586 | 62 | GC03M049359 |
| MARVELD3 | MARVEL Domain Containing 3 | Protein Coding | Q96A59 | 43 | GC16P071626 |
| ART1 | ADP-Ribosyltransferase 1 | Protein Coding | P52961 | 45 | GC11P022823 |
| PTPN14 | Protein Tyrosine Phosphatase Non-Receptor Type 14 | Protein Coding | Q15678 | 55 | GC01M214348 |
| ALDH1A1 | Aldehyde Dehydrogenase 1 Family Member A1 | Protein Coding | P00352 | 60 | GC09M072900 |
| AR | Androgen Receptor | Protein Coding | P10275 | 65 | GC0XP067544 |
| ARHGEF2 | Rho/Rac Guanine Nucleotide Exchange Factor 2 | Protein Coding | Q92974 | 59 | GC01M155946 |
| SMAD9 | SMAD Family Member 9 | Protein Coding | O15198 | 58 | GC13M036844 |
| PTPN6 | Protein Tyrosine Phosphatase Non-Receptor Type 6 | Protein Coding | P29350 | 63 | GC12P080294 |
| F3 | Coagulation Factor III, Tissue Factor | Protein Coding | P13726 | 58 | GC01M095087 |
| CD274 | CD274 Molecule | Protein Coding | Q9NZQ7 | 59 | GC09P005450 |
| SMC3 | Structural Maintenance Of Chromosomes 3 | Protein Coding | Q9UQE7 | 60 | GC10P110567 |
| ARRB1 | Arrestin Beta 1 | Protein Coding | P49407 | 57 | GC11M143283 |
| SCUBE2 | Signal Peptide, CUB Domain And EGF Like Domain Containing 2 | Protein Coding | Q9NQ36 | 51 | GC11M009019 |
| F2RL2 | Coagulation Factor II Thrombin Receptor Like 2 | Protein Coding | O00254 | 52 | GC05M076615 |
| FOXM1 | Forkhead Box M1 | Protein Coding | Q08050 | 56 | GC12M002857 |
| LATS1 | Large Tumor Suppressor Kinase 1 | Protein Coding | O95835 | 57 | GC06M149658 |
| LEP | Leptin | Protein Coding | P41159 | 59 | GC07P128241 |
| EPB41L5 | Erythrocyte Membrane Protein Band 4.1 Like 5 | Protein Coding | Q9HCM4 | 45 | GC02P120013 |
| MMP3 | Matrix Metallopeptidase 3 | Protein Coding | P08254 | 62 | GC11M102835 |
| F2 | Coagulation Factor II, Thrombin | Protein Coding | P00734 | 63 | GC11P049467 |
| YAP1 | Yes1 Associated Transcriptional Regulator | Protein Coding | P46937 | 61 | GC11P102110 |
| NMI | N-Myc And STAT Interactor | Protein Coding | Q13287 | 47 | GC02M151270 |
| L1CAM | L1 Cell Adhesion Molecule | Protein Coding | P32004 | 60 | GC0XM153864 |
| PTN | Pleiotrophin | Protein Coding | P21246 | 53 | GC07M137227 |
| MMP1 | Matrix Metallopeptidase 1 | Protein Coding | P03956 | 62 | GC11M143594 |
| ETV1 | ETS Variant Transcription Factor 1 | Protein Coding | P50549 | 57 | GC07M013891 |
| SRC | SRC Proto-Oncogene, Non-Receptor Tyrosine Kinase | Protein Coding | P12931 | 63 | GC20P037344 |
| RHOC | Ras Homolog Family Member C | Protein Coding | P08134 | 55 | GC01M112701 |
| STIM2 | Stromal Interaction Molecule 2 | Protein Coding | Q9P246 | 52 | GC04P032386 |
| ASCL1 | Achaete-Scute Family BHLH Transcription Factor 1 | Protein Coding | P50553 | 56 | GC12P102957 |
| ETS2 | ETS Proto-Oncogene 2, Transcription Factor | Protein Coding | P15036 | 53 | GC21P038805 |
| PSTPIP1 | Proline-Serine-Threonine Phosphatase Interacting Protein 1 | Protein Coding | O43586 | 55 | GC15P076993 |
| MTUS1 | Microtubule Associated Scaffold Protein 1 | Protein Coding | Q9ULD2 | 47 | GC08M017643 |
| MGAT3 | Beta-1,4-Mannosyl-Glycoprotein 4-Beta-N-Acetylglucosaminyltransferase | Protein Coding | Q09327 | 51 | GC22P097421 |
| ETS1 | ETS Proto-Oncogene 1, Transcription Factor | Protein Coding | P14921 | 61 | GC11M128458 |
| SOCS3 | Suppressor Of Cytokine Signaling 3 | Protein Coding | O14543 | 57 | GC17M078356 |
| BIRC5 | Baculoviral IAP Repeat Containing 5 | Protein Coding | O15392 | 59 | GC17P078214 |
| MDK | Midkine | Protein Coding | P21741 | 57 | GC11P046380 |
| ESRRB | Estrogen Related Receptor Beta | Protein Coding | O95718 | 61 | GC14P076310 |
| HS6ST2 | Heparan Sulfate 6-O-Sulfotransferase 2 | Protein Coding | Q96MM7 | 50 | GC0XM132626 |
| HSPA5 | Heat Shock Protein Family A (Hsp70) Member 5 | Protein Coding | P11021 | 62 | GC09M125234 |
| MCL1 | MCL1 Apoptosis Regulator, BCL2 Family Member | Protein Coding | Q07820 | 62 | GC01M168398 |
| ALAD | Aminolevulinate Dehydratase | Protein Coding | P13716 | 59 | GC09M113386 |
| JAK2 | Janus Kinase 2 | Protein Coding | O60674 | 66 | GC09P004985 |
| CCNG2 | Cyclin G2 | Protein Coding | Q16589 | 49 | GC04P077214 |
| HOXA13 | Homeobox A13 | Protein Coding | P31271 | 52 | GC07M028281 |
| ERN1 | Endoplasmic Reticulum To Nucleus Signaling 1 | Protein Coding | O75460 | 60 | GC17M064039 |
| WWTR1 | WW Domain Containing Transcription Regulator 1 | Protein Coding | Q9GZV5 | 50 | GC03M149517 |
| CDKL2 | Cyclin Dependent Kinase Like 2 | Protein Coding | Q92772 | 45 | GC04M075576 |
| ANXA2 | Annexin A2 | Protein Coding | P07355 | 60 | GC15M060347 |
| ERF | ETS2 Repressor Factor | Protein Coding | P50548 | 52 | GC19M042247 |
| CDH2 | Cadherin 2 | Protein Coding | P19022 | 64 | GC18M039248 |
| BTRC | Beta-Transducin Repeat Containing E3 Ubiquitin Protein Ligase | Protein Coding | Q9Y297 | 57 | GC10P101354 |
| JUNB | JunB Proto-Oncogene, AP-1 Transcription Factor Subunit | Protein Coding | P17275 | 53 | GC19P012791 |
| PDCD4 | Programmed Cell Death 4 | Protein Coding | Q53EL6 | 55 | GC10P110871 |
| EPHB3 | EPH Receptor B3 | Protein Coding | P54753 | 59 | GC03P184561 |
| TNF | Tumor Necrosis Factor | Protein Coding | P01375 | 63 | GC06P190708 |
| CCND2 | Cyclin D2 | Protein Coding | P30279 | 60 | GC12P080230 |
| ITGA5 | Integrin Subunit Alpha 5 | Protein Coding | P08648 | 61 | GC12M060600 |
| GATA3 | GATA Binding Protein 3 | Protein Coding | P23771 | 63 | GC10P008045 |
| RAB22A | RAB22A, Member RAS Oncogene Family | Protein Coding | Q9UL26 | 43 | GC20P058309 |
| EPHA8 | EPH Receptor A8 | Protein Coding | P29322 | 51 | GC01P022563 |
| CXCL12 | C-X-C Motif Chemokine Ligand 12 | Protein Coding | P48061 | 59 | GC10M044370 |
| TIMELESS | Timeless Circadian Regulator | Protein Coding | Q9UNS1 | 53 | GC12M056416 |
| IGFBP7 | Insulin Like Growth Factor Binding Protein 7 | Protein Coding | Q16270 | 59 | GC04M057030 |
| ALK | ALK Receptor Tyrosine Kinase | Protein Coding | Q9UM73 | 64 | GC02M029389 |
| CAMK1D | Calcium/Calmodulin Dependent Protein Kinase ID | Protein Coding | Q8IU85 | 53 | GC10P012349 |
| EPHA4 | EPH Receptor A4 | Protein Coding | P54764 | 63 | GC02M221418 |
| RELA | RELA Proto-Oncogene, NF-KB Subunit | Protein Coding | Q04206 | 64 | GC11M065653 |
| NR1I2 | Nuclear Receptor Subfamily 1 Group I Member 2 | Protein Coding | O75469 | 56 | GC03P119780 |
| HSPB1 | Heat Shock Protein Family B (Small) Member 1 | Protein Coding | P04792 | 65 | GC07P076302 |
| FLOT2 | Flotillin 2 | Protein Coding | Q14254 | 51 | GC17M100473 |
| TBX20 | T-Box Transcription Factor 20 | Protein Coding | Q9UMR3 | 52 | GC07M035237 |
| EP300 | EP300 Lysine Acetyltransferase | Protein Coding | Q09472 | 65 | GC22P097487 |
| MAPK3 | Mitogen-Activated Protein Kinase 3 | Protein Coding | P27361 | 62 | GC16M053300 |
| KAT2B | Lysine Acetyltransferase 2B | Protein Coding | Q92831 | 62 | GC03P028992 |
| HSPA4 | Heat Shock Protein Family A (Hsp70) Member 4 | Protein Coding | P34932 | 56 | GC05P133148 |
| FOXO3 | Forkhead Box O3 | Protein Coding | O43524 | 61 | GC06P108559 |
| POGLUT1 | Protein O-Glucosyltransferase 1 | Protein Coding | Q8NBL1 | 53 | GC03P119468 |
| ENO1 | Enolase 1 | Protein Coding | P06733 | 62 | GC01M008861 |
| INPP4B | Inositol Polyphosphate-4-Phosphatase Type II B | Protein Coding | O15327 | 52 | GC04M142023 |
| HOXB7 | Homeobox B7 | Protein Coding | P09629 | 52 | GC17M048607 |
| FOXC1 | Forkhead Box C1 | Protein Coding | Q12948 | 55 | GC06P002371 |
| PROX1 | Prospero Homeobox 1 | Protein Coding | Q92786 | 56 | GC01P213983 |
| ENG | Endoglin | Protein Coding | P17813 | 60 | GC09M132959 |
| SCEL | Sciellin | Protein Coding | O95171 | 43 | GC13P077535 |
| FOXA2 | Forkhead Box A2 | Protein Coding | Q9Y261 | 60 | GC20M022581 |
| FGFR2 | Fibroblast Growth Factor Receptor 2 | Protein Coding | P21802 | 68 | GC10M121478 |
| AJAP1 | Adherens Junctions Associated Protein 1 | Protein Coding | Q9UKB5 | 43 | GC01P004654 |
| EMP3 | Epithelial Membrane Protein 3 (MAM Blood Group) | Protein Coding | P54852 | 47 | GC19P048321 |
| CXCL8 | C-X-C Motif Chemokine Ligand 8 | Protein Coding | P10145 | 56 | GC04P073740 |
| TNFRSF11A | TNF Receptor Superfamily Member 11a | Protein Coding | Q9Y6Q6 | 59 | GC18P062325 |
| HMOX1 | Heme Oxygenase 1 | Protein Coding | P09601 | 65 | GC22P035380 |
| FGF2 | Fibroblast Growth Factor 2 | Protein Coding | P09038 | 59 | GC04P122826 |
| PRKCQ | Protein Kinase C Theta | Protein Coding | Q04759 | 62 | GC10M007310 |
| FBXO45 | F-Box Protein 45 | Protein Coding | P0C2W1 | 44 | GC03P196568 |
| HGF | Hepatocyte Growth Factor | Protein Coding | P14210 | 65 | GC07M081699 |
| TNFSF10 | TNF Superfamily Member 10 | Protein Coding | P50591 | 59 | GC03M172505 |
| HDGF | Heparin Binding Growth Factor | Protein Coding | P51858 | 52 | GC01M168720 |
| AKT2 | AKT Serine/Threonine Kinase 2 | Protein Coding | P31751 | 66 | GC19M040230 |
| HDAC8 | Histone Deacetylase 8 | Protein Coding | Q9BY41 | 60 | GC0XM072329 |
| RPL22L1 | Ribosomal Protein L22 Like 1 | Protein Coding | Q6P5R6 | 46 | GC03M170864 |
| FN1 | Fibronectin 1 | Protein Coding | P02751 | 63 | GC02M215360 |
| TNFSF12 | TNF Superfamily Member 12 | Protein Coding | O43508 | 51 | GC17P164144 |
| HAS2 | Hyaluronan Synthase 2 | Protein Coding | Q92819 | 55 | GC08M121612 |
| EPAS1 | Endothelial PAS Domain Protein 1 | Protein Coding | Q99814 | 63 | GC02P046293 |
| PAG1 | Phosphoprotein Membrane Anchor With Glycosphingolipid Microdomains 1 | Protein Coding | Q9NWQ8 | 50 | GC08M080967 |
| ELK3 | ETS Transcription Factor ELK3 | Protein Coding | P41970 | 45 | GC12P096194 |
| TWIST2 | Twist Family BHLH Transcription Factor 2 | Protein Coding | Q8WVJ9 | 54 | GC02P238848 |
| CBR1 | Carbonyl Reductase 1 | Protein Coding | P16152 | 57 | GC21P036069 |
| SENP1 | SUMO Specific Peptidase 1 | Protein Coding | Q9P0U3 | 55 | GC12M048042 |
| DNMT1 | DNA Methyltransferase 1 | Protein Coding | P26358 | 66 | GC19M010133 |
| KDM3A | Lysine Demethylase 3A | Protein Coding | Q9Y4C1 | 55 | GC02P100409 |
| ELK1 | ETS Transcription Factor ELK1 | Protein Coding | P19419 | 58 | GC0XM047635 |
| TP63 | Tumor Protein P63 | Protein Coding | Q9H3D4 | 58 | GC03P189598 |
| EED | Embryonic Ectoderm Development | Protein Coding | O75530 | 56 | GC11P115731 |
| RGCC | Regulator Of Cell Cycle | Protein Coding | Q9H4X1 | 47 | GC13P041457 |
| DAB2 | DAB Adaptor Protein 2 | Protein Coding | P98082 | 56 | GC05M039371 |
| FOXJ2 | Forkhead Box J2 | Protein Coding | Q9P0K8 | 41 | GC12P008032 |
| CYP4Z1 | Cytochrome P450 Family 4 Subfamily Z Member 1 | Protein Coding | Q86W10 | 44 | GC01P085823 |
| NANOG | Nanog Homeobox | Protein Coding | Q9H9S0 | 50 | GC12P007787 |
| ABCC3 | ATP Binding Cassette Subfamily C Member 3 | Protein Coding | O15438 | 58 | GC17P050634 |
| CUX1 | Cut Like Homeobox 1 | Protein Coding | Q13948 | 57 | GC07P101815 |
| PRKCE | Protein Kinase C Epsilon | Protein Coding | Q02156 | 61 | GC02P045651 |
| ELAVL1 | ELAV Like RNA Binding Protein 1 | Protein Coding | Q15717 | 55 | GC19M007958 |
| VEGFA | Vascular Endothelial Growth Factor A | Protein Coding | P15692 | 61 | GC06P043770 |
| CBLB | Cbl Proto-Oncogene B | Protein Coding | Q13191 | 59 | GC03M105655 |
| GPC3 | Glypican 3 | Protein Coding | P51654 | 59 | GC0XM133535 |
| CTNND1 | Catenin Delta 1 | Protein Coding | O60716 | 58 | GC11P113599 |
| PRKAA2 | Protein Kinase AMP-Activated Catalytic Subunit Alpha 2 | Protein Coding | P54646 | 62 | GC01P056645 |
| EIF5A | Eukaryotic Translation Initiation Factor 5A | Protein Coding | P63241 | 56 | GC17P007306 |
| SP1 | Sp1 Transcription Factor | Protein Coding | P08047 | 60 | GC12P053380 |
| EIF3I | Eukaryotic Translation Initiation Factor 3 Subunit I | Protein Coding | Q13347 | 49 | GC01P032221 |
| USP22 | Ubiquitin Specific Peptidase 22 | Protein Coding | Q9UPT9 | 53 | GC17M020999 |
| CTBP1 | C-Terminal Binding Protein 1 | Protein Coding | Q13363 | 60 | GC04M001211 |
| FERMT1 | FERM Domain Containing Kindlin 1 | Protein Coding | Q9BQL6 | 53 | GC20M006074 |
| EIF4G1 | Eukaryotic Translation Initiation Factor 4 Gamma 1 | Protein Coding | Q04637 | 59 | GC03P184314 |
| S100A4 | S100 Calcium Binding Protein A4 | Protein Coding | P26447 | 57 | GC01M153543 |
| IRS2 | Insulin Receptor Substrate 2 | Protein Coding | Q9Y4H2 | 59 | GC13M109752 |
| FOXO1 | Forkhead Box O1 | Protein Coding | Q12778 | 64 | GC13M040555 |
| MAPK14 | Mitogen-Activated Protein Kinase 14 | Protein Coding | Q16539 | 65 | GC06P190827 |
| EIF4E | Eukaryotic Translation Initiation Factor 4E | Protein Coding | P06730 | 62 | GC04M098879 |
| PTK2 | Protein Tyrosine Kinase 2 | Protein Coding | Q05397 | 62 | GC08M140657 |
| SCIN | Scinderin | Protein Coding | Q9Y6U3 | 50 | GC07P012570 |
| DKK1 | Dickkopf Wnt Signaling Pathway Inhibitor 1 | Protein Coding | O94907 | 56 | GC10P052314 |
| NLRP3 | NLR Family Pyrin Domain Containing 3 | Protein Coding | Q96P20 | 62 | GC01P247692 |
| LGR4 | Leucine Rich Repeat Containing G Protein-Coupled Receptor 4 | Protein Coding | Q9BXB1 | 52 | GC11M027365 |
| EIF2S1 | Eukaryotic Translation Initiation Factor 2 Subunit Alpha | Protein Coding | P05198 | 58 | GC14P067359 |
| MAPK7 | Mitogen-Activated Protein Kinase 7 | Protein Coding | Q13164 | 61 | GC17P164512 |
| IKBKG | Inhibitor Of Nuclear Factor Kappa B Kinase Regulatory Subunit Gamma | Protein Coding | Q9Y6K9 | 60 | GC0XP154541 |
| EFEMP1 | EGF Containing Fibulin Extracellular Matrix Protein 1 | Protein Coding | Q12805 | 56 | GC02M055865 |
| BRF2 | BRF2 General Transcription Factor IIIB Subunit | Protein Coding | Q9HAW0 | 49 | GC08M037843 |
| MUC1 | Mucin 1, Cell Surface Associated | Protein Coding | P15941 | 61 | GC01M155185 |
| PIK3R3 | Phosphoinositide-3-Kinase Regulatory Subunit 3 | Protein Coding | Q92569 | 57 | GC01M046041 |
| VWCE | Von Willebrand Factor C And EGF Domains | Protein Coding | Q96DN2 | 40 | GC11M061258 |
| LYN | LYN Proto-Oncogene, Src Family Tyrosine Kinase | Protein Coding | P07948 | 63 | GC08P055879 |
| AGR2 | Anterior Gradient 2, Protein Disulphide Isomerase Family Member | Protein Coding | O95994 | 52 | GC07M017630 |
| IMP3 | IMP U3 Small Nucleolar Ribonucleoprotein 3 | Protein Coding | Q9NV31 | 46 | GC15M075639 |
| EFNB2 | Ephrin B2 | Protein Coding | P52799 | 59 | GC13M106489 |
| HMGB1 | High Mobility Group Box 1 | Protein Coding | P09429 | 63 | GC13M030456 |
| AJUBA | Ajuba LIM Protein | Protein Coding | Q96IF1 | 50 | GC14M022971 |
| F2R | Coagulation Factor II Thrombin Receptor | Protein Coding | P25116 | 59 | GC05P076716 |
| LTBP1 | Latent Transforming Growth Factor Beta Binding Protein 1 | Protein Coding | Q14766 | 57 | GC02P032946 |
| NDRG1 | N-Myc Downstream Regulated 1 | Protein Coding | Q92597 | 57 | GC08M133237 |
| PTPA | Protein Phosphatase 2 Phosphatase Activator | Protein Coding | Q15257 | 58 | GC09P156015 |
| EEF1D | Eukaryotic Translation Elongation Factor 1 Delta | Protein Coding | P29692 | 53 | GC08M143579 |
| EZH2 | Enhancer Of Zeste 2 Polycomb Repressive Complex 2 Subunit | Protein Coding | Q15910 | 66 | GC07M148807 |
| PPM1D | Protein Phosphatase, Mg2+/Mn2+ Dependent 1D | Protein Coding | O15297 | 60 | GC17P060600 |
| ELF5 | E74 Like ETS Transcription Factor 5 | Protein Coding | Q9UKW6 | 47 | GC11M034500 |
| LRP6 | LDL Receptor Related Protein 6 | Protein Coding | O75581 | 59 | GC12M037073 |
| CDKN1B | Cyclin Dependent Kinase Inhibitor 1B | Protein Coding | P46527 | 61 | GC12P080490 |
| PPP1R8 | Protein Phosphatase 1 Regulatory Subunit 8 | Protein Coding | Q12972 | 49 | GC01P027830 |
| EDNRA | Endothelin Receptor Type A | Protein Coding | P25101 | 62 | GC04P147480 |
| ESR1 | Estrogen Receptor 1 | Protein Coding | P03372 | 68 | GC06P151656 |
| GEMIN2 | Gem Nuclear Organelle Associated Protein 2 | Protein Coding | O14893 | 51 | GC14P039114 |
| EPHA2 | EPH Receptor A2 | Protein Coding | P29317 | 65 | GC01M016124 |
| LMNB1 | Lamin B1 | Protein Coding | P20700 | 59 | GC05P126776 |
| CD24 | CD24 Molecule | Protein Coding | P25063 | 47 | GC06M106969 |
| GIPC2 | GIPC PDZ Domain Containing Family Member 2 | Protein Coding | Q8TF65 | 47 | GC01P086701 |
| ECT2 | Epithelial Cell Transforming 2 | Protein Coding | Q9H8V3 | 53 | GC03P172750 |
| EDN1 | Endothelin 1 | Protein Coding | P05305 | 61 | GC06P014983 |
| LIMS1 | LIM Zinc Finger Domain Containing 1 | Protein Coding | P48059 | 53 | GC02P108534 |
| TNFSF15 | TNF Superfamily Member 15 | Protein Coding | O95150 | 56 | GC09M114784 |
| POMC | Proopiomelanocortin | Protein Coding | P01189 | 61 | GC02M025160 |
| DVL2 | Dishevelled Segment Polarity Protein 2 | Protein Coding | O14641 | 59 | GC17M007225 |
| CAV1 | Caveolin 1 | Protein Coding | Q03135 | 61 | GC07P116524 |
| SEMA7A | Semaphorin 7A (JohnMiltonHagen Blood Group) | Protein Coding | O75326 | 56 | GC15M074409 |
| JAG1 | Jagged Canonical Notch Ligand 1 | Protein Coding | P78504 | 63 | GC20M010637 |
| LIF | LIF Interleukin 6 Family Cytokine | Protein Coding | P15018 | 56 | GC22M030240 |
| GDF15 | Growth Differentiation Factor 15 | Protein Coding | Q99988 | 56 | GC19P161671 |
| ERRFI1 | ERBB Receptor Feedback Inhibitor 1 | Protein Coding | Q9UJM3 | 50 | GC01M008004 |
| AGTR1 | Angiotensin II Receptor Type 1 | Protein Coding | P30556 | 65 | GC03P148697 |
| SOX2 | SRY-Box Transcription Factor 2 | Protein Coding | P48431 | 58 | GC03P181711 |
| LOXL3 | Lysyl Oxidase Like 3 | Protein Coding | P58215 | 57 | GC02M074532 |
| DLX2 | Distal-Less Homeobox 2 | Protein Coding | Q07687 | 50 | GC02M172099 |
| LGALS9 | Galectin 9 | Protein Coding | O00182 | 51 | GC17P027629 |
| TP53INP1 | Tumor Protein P53 Inducible Nuclear Protein 1 | Protein Coding | Q96A56 | 49 | GC08M094925 |
| MBD3 | Methyl-CpG Binding Domain Protein 3 | Protein Coding | O95983 | 53 | GC19M111548 |
| DNMT3B | DNA Methyltransferase 3 Beta | Protein Coding | Q9UBC3 | 63 | GC20P032762 |
| BMP7 | Bone Morphogenetic Protein 7 | Protein Coding | P18075 | 60 | GC20M057168 |
| HINT2 | Histidine Triad Nucleotide Binding Protein 2 | Protein Coding | Q9BX68 | 46 | GC09M035812 |
| CTSL | Cathepsin L | Protein Coding | P07711 | 60 | GC09P087728 |
| LAMC2 | Laminin Subunit Gamma 2 | Protein Coding | Q13753 | 59 | GC01P183186 |
| TM4SF5 | Transmembrane 4 L Six Family Member 5 | Protein Coding | O14894 | 39 | GC17P004771 |
| PLS3 | Plastin 3 | Protein Coding | P13797 | 54 | GC0XP115560 |
| DDX5 | DEAD-Box Helicase 5 | Protein Coding | P17844 | 59 | GC17M064498 |
| BMP4 | Bone Morphogenetic Protein 4 | Protein Coding | P12644 | 62 | GC14M053949 |
| SPZ1 | Spermatogenic Leucine Zipper 1 | Protein Coding | Q9BXG8 | 40 | GC05P080319 |
| PARP1 | Poly(ADP-Ribose) Polymerase 1 | Protein Coding | P09874 | 65 | GC01M226360 |
| WNT3A | Wnt Family Member 3A | Protein Coding | P56704 | 60 | GC01P233813 |
| PLAGL2 | PLAG1 Like Zinc Finger 2 | Protein Coding | Q9UPG8 | 47 | GC20M032192 |
| DAPK1 | Death Associated Protein Kinase 1 | Protein Coding | P53355 | 62 | GC09P087497 |
| SHH | Sonic Hedgehog Signaling Molecule | Protein Coding | Q15465 | 63 | GC07M155799 |
| TEAD2 | TEA Domain Transcription Factor 2 | Protein Coding | Q15562 | 47 | GC19M112808 |
| CRKL | CRK Like Proto-Oncogene, Adaptor Protein | Protein Coding | P46109 | 59 | GC22P020917 |
| RHOG | Ras Homolog Family Member G | Protein Coding | P84095 | 54 | GC11M015922 |
| PLA2G4A | Phospholipase A2 Group IVA | Protein Coding | P47712 | 62 | GC01P186798 |
| LMNA | Lamin A/C | Protein Coding | P02545 | 63 | GC01P156082 |
| CYP3A5 | Cytochrome P450 Family 3 Subfamily A Member 5 | Protein Coding | P20815 | 58 | GC07M099648 |
| PTGS2 | Prostaglandin-Endoperoxide Synthase 2 | Protein Coding | P35354 | 63 | GC01M186671 |
| FOXN1 | Forkhead Box N1 | Protein Coding | O15353 | 52 | GC17P028506 |
| FERMT2 | FERM Domain Containing Kindlin 2 | Protein Coding | Q96AC1 | 50 | GC14M052857 |
| NANOGP8 | Nanog Homeobox Retrogene P8 | Protein Coding | Q6NSW7 | 33 | GC15M035083 |
| NRP1 | Neuropilin 1 | Protein Coding | O14786 | 61 | GC10M033177 |
| PIK3R1 | Phosphoinositide-3-Kinase Regulatory Subunit 1 | Protein Coding | P27986 | 65 | GC05P068215 |
| LGALS3 | Galectin 3 | Protein Coding | P17931 | 57 | GC14P055124 |
| PEBP4 | Phosphatidylethanolamine Binding Protein 4 | Protein Coding | Q96S96 | 43 | GC08M022713 |
| POU5F1 | POU Class 5 Homeobox 1 | Protein Coding | Q01860 | 60 | GC06M111459 |
| CUL3 | Cullin 3 | Protein Coding | Q13618 | 60 | GC02M224470 |
| TXNIP | Thioredoxin Interacting Protein | Protein Coding | Q9H3M7 | 53 | GC01M145992 |
| KRT19 | Keratin 19 | Protein Coding | P08727 | 56 | GC17M041523 |
| ACTL6A | Actin Like 6A | Protein Coding | O96019 | 53 | GC03P179562 |
| SERPINI1 | Serpin Family I Member 1 | Protein Coding | Q99574 | 58 | GC03P167735 |
| LASP1 | LIM And SH3 Protein 1 | Protein Coding | Q14847 | 53 | GC17P038869 |
| ZNF746 | Zinc Finger Protein 746 | Protein Coding | Q6NUN9 | 48 | GC07M149472 |
| PRRX1 | Paired Related Homeobox 1 | Protein Coding | P54821 | 53 | GC01P170662 |
| KAT8 | Lysine Acetyltransferase 8 | Protein Coding | Q9H7Z6 | 57 | GC16P128967 |
| SPRY2 | Sprouty RTK Signaling Antagonist 2 | Protein Coding | O43597 | 60 | GC13M080335 |
| RHOB | Ras Homolog Family Member B | Protein Coding | P62745 | 57 | GC02P020447 |
| CUL4A | Cullin 4A | Protein Coding | Q13619 | 56 | GC13P113208 |
| ABCB1 | ATP Binding Cassette Subfamily B Member 1 | Protein Coding | P08183 | 64 | GC07M087504 |
| LAMA5 | Laminin Subunit Alpha 5 | Protein Coding | O15230 | 56 | GC20M062307 |
| CYP1B1 | Cytochrome P450 Family 1 Subfamily B Member 1 | Protein Coding | Q16678 | 60 | GC02M038066 |
| KRAS | KRAS Proto-Oncogene, GTPase | Protein Coding | P01116 | 66 | GC12M037206 |
| SPOP | Speckle Type BTB/POZ Protein | Protein Coding | O43791 | 56 | GC17M049598 |
| ADAM10 | ADAM Metallopeptidase Domain 10 | Protein Coding | O14672 | 66 | GC15M058588 |
| KMT5A | Lysine Methyltransferase 5A | Protein Coding | Q9NQR1 | 53 | GC12P140388 |
| FZD7 | Frizzled Class Receptor 7 | Protein Coding | O75084 | 56 | GC02P205045 |
| STK26 | Serine/Threonine Kinase 26 | Protein Coding | Q9P289 | 54 | GC0XP132023 |
| KIT | KIT Proto-Oncogene, Receptor Tyrosine Kinase | Protein Coding | P10721 | 66 | GC04P054657 |
| CMTM8 | CKLF Like MARVEL Transmembrane Domain Containing 8 | Protein Coding | Q8IZV2 | 43 | GC03P032238 |
| ID1 | Inhibitor Of DNA Binding 1 | Protein Coding | P41134 | 54 | GC20P055608 |
| MARVELD1 | MARVEL Domain Containing 1 | Protein Coding | Q9BSK0 | 42 | GC10P097713 |
| TRIM28 | Tripartite Motif Containing 28 | Protein Coding | Q13263 | 57 | GC19P058544 |
| KRT18 | Keratin 18 | Protein Coding | P05783 | 62 | GC12P052948 |
| FOSL1 | FOS Like 1, AP-1 Transcription Factor Subunit | Protein Coding | P15407 | 57 | GC11M143008 |
| WWOX | WW Domain Containing Oxidoreductase | Protein Coding | Q9NZC7 | 59 | GC16P078099 |
| CD82 | CD82 Molecule | Protein Coding | P27701 | 56 | GC11P044564 |
| CSPG4 | Chondroitin Sulfate Proteoglycan 4 | Protein Coding | Q6UVK1 | 60 | GC15M075674 |
| ITCH | Itchy E3 Ubiquitin Protein Ligase | Protein Coding | Q96J02 | 60 | GC20P034363 |
| NAMPT | Nicotinamide Phosphoribosyltransferase | Protein Coding | P43490 | 60 | GC07M106248 |
| KRT17 | Keratin 17 | Protein Coding | Q04695 | 58 | GC17M041619 |
| ADAM12 | ADAM Metallopeptidase Domain 12 | Protein Coding | O43184 | 58 | GC10M126012 |
| UIMC1 | Ubiquitin Interaction Motif Containing 1 | Protein Coding | Q96RL1 | 54 | GC05M176905 |
| JARID2 | Jumonji And AT-Rich Interaction Domain Containing 2 | Protein Coding | Q92833 | 55 | GC06P015246 |
| VCAN | Versican | Protein Coding | P13611 | 59 | GC05P083471 |
| FGFR1 | Fibroblast Growth Factor Receptor 1 | Protein Coding | P11362 | 68 | GC08M038400 |
| YPEL3 | Yippee Like 3 | Protein Coding | P61236 | 47 | GC16M053297 |
| KRT8 | Keratin 8 | Protein Coding | P05787 | 59 | GC12M052897 |
| PDGFD | Platelet Derived Growth Factor D | Protein Coding | Q9GZP0 | 55 | GC11M103907 |
| CYB5R1 | Cytochrome B5 Reductase 1 | Protein Coding | Q9UHQ9 | 52 | GC01M202964 |
| ITGB4 | Integrin Subunit Beta 4 | Protein Coding | P16144 | 62 | GC17P075721 |
| CSK | C-Terminal Src Kinase | Protein Coding | P41240 | 59 | GC15P074782 |
| POSTN | Periostin | Protein Coding | Q15063 | 56 | GC13M037562 |
| CAPZA1 | Capping Actin Protein Of Muscle Z-Line Subunit Alpha 1 | Protein Coding | P52907 | 52 | GC01P112619 |
| HDAC6 | Histone Deacetylase 6 | Protein Coding | Q9UBN7 | 66 | GC0XP048801 |
| KRT7 | Keratin 7 | Protein Coding | P08729 | 56 | GC12P052232 |
| VASH2 | Vasohibin 2 | Protein Coding | Q86V25 | 41 | GC01P212950 |
| PDGFRB | Platelet Derived Growth Factor Receptor Beta | Protein Coding | P09619 | 66 | GC05M150113 |
| ITGA3 | Integrin Subunit Alpha 3 | Protein Coding | P26006 | 60 | GC17P050055 |
| CSF2 | Colony Stimulating Factor 2 | Protein Coding | P04141 | 59 | GC05P132073 |
| FOXQ1 | Forkhead Box Q1 | Protein Coding | Q9C009 | 42 | GC06P001312 |
| CAPNS1 | Calpain Small Subunit 1 | Protein Coding | P04632 | 55 | GC19P161977 |
| ZBTB33 | Zinc Finger And BTB Domain Containing 33 | Protein Coding | Q86T24 | 50 | GC0XP120250 |
| KIF5B | Kinesin Family Member 5B | Protein Coding | P33176 | 60 | GC10M037506 |
| TBL1XR1 | TBL1X/Y Related 1 | Protein Coding | Q9BZK7 | 58 | GC03M177019 |
| ACKR4 | Atypical Chemokine Receptor 4 | Protein Coding | Q9NPB9 | 44 | GC03P132597 |
| ITGA2 | Integrin Subunit Alpha 2 | Protein Coding | P17301 | 59 | GC05P052989 |
| CRP | C-Reactive Protein | Protein Coding | P02741 | 59 | GC01M168779 |
| MTA1 | Metastasis Associated 1 | Protein Coding | Q13330 | 55 | GC14P105419 |
| SMC1A | Structural Maintenance Of Chromosomes 1A | Protein Coding | Q14683 | 60 | GC0XM053374 |
| CDC42 | Cell Division Cycle 42 | Protein Coding | P60953 | 63 | GC01P022052 |
| KDR | Kinase Insert Domain Receptor | Protein Coding | P35968 | 67 | GC04M055078 |
| TNFAIP8L2 | TNF Alpha Induced Protein 8 Like 2 | Protein Coding | Q6P589 | 39 | GC01P151156 |
| PDGFB | Platelet Derived Growth Factor Subunit B | Protein Coding | P01127 | 63 | GC22M089269 |
| AQP5 | Aquaporin 5 | Protein Coding | P55064 | 59 | GC12P049961 |
| CRMP1 | Collapsin Response Mediator Protein 1 | Protein Coding | Q14194 | 53 | GC04M006883 |
| GRHL2 | Grainyhead Like Transcription Factor 2 | Protein Coding | Q6ISB3 | 52 | GC08P101492 |
| USP9X | Ubiquitin Specific Peptidase 9 X-Linked | Protein Coding | Q93008 | 60 | GC0XP041085 |
| HS3ST3B1 | Heparan Sulfate-Glucosamine 3-Sulfotransferase 3B1 | Protein Coding | Q9Y662 | 50 | GC17P014301 |
| KCNN4 | Potassium Calcium-Activated Channel Subfamily N Member 4 | Protein Coding | O15554 | 60 | GC19M112612 |
| YWHAG | Tyrosine 3-Monooxygenase/Tryptophan 5-Monooxygenase Activation Protein Gamma | Protein Coding | P61981 | 60 | GC07M082147 |
| LIMA1 | LIM Domain And Actin Binding 1 | Protein Coding | Q9UHB6 | 49 | GC12M050175 |
| AQP3 | Aquaporin 3 (Gill Blood Group) | Protein Coding | Q92482 | 59 | GC09M033431 |
| FOXR2 | Forkhead Box R2 | Protein Coding | Q6PJQ5 | 37 | GC0XP055623 |
| TRPS1 | Transcriptional Repressor GATA Binding 1 | Protein Coding | Q9UHF7 | 56 | GC08M115408 |
| VANGL1 | VANGL Planar Cell Polarity Protein 1 | Protein Coding | Q8TAA9 | 55 | GC01P115641 |
| SETDB1 | SET Domain Bifurcated Histone Lysine Methyltransferase 1 | Protein Coding | Q15047 | 56 | GC01P150926 |
| KCNH1 | Potassium Voltage-Gated Channel Subfamily H Member 1 | Protein Coding | O95259 | 58 | GC01M210678 |
| YY1 | YY1 Transcription Factor | Protein Coding | P25490 | 60 | GC14P100238 |
| PRRX2 | Paired Related Homeobox 2 | Protein Coding | Q99811 | 43 | GC09P129665 |
| IL11 | Interleukin 11 | Protein Coding | P20809 | 53 | GC19M055364 |
| CREBBP | CREB Binding Lysine Acetyltransferase | Protein Coding | Q92793 | 68 | GC16M052313 |
| ADAM17 | ADAM Metallopeptidase Domain 17 | Protein Coding | P78536 | 63 | GC02M009488 |
| TRIM11 | Tripartite Motif Containing 11 | Protein Coding | Q96F44 | 50 | GC01M228393 |
| HDAC4 | Histone Deacetylase 4 | Protein Coding | P56524 | 65 | GC02M239048 |
| JAK3 | Janus Kinase 3 | Protein Coding | P52333 | 65 | GC19M017824 |
| XBP1 | X-Box Binding Protein 1 | Protein Coding | P17861 | 59 | GC22M028794 |
| PDE4A | Phosphodiesterase 4A | Protein Coding | P27815 | 58 | GC19P010416 |
| CXCR2 | C-X-C Motif Chemokine Receptor 2 | Protein Coding | P25025 | 63 | GC02P218125 |
| ADM | Adrenomedullin | Protein Coding | P35318 | 57 | GC11P023149 |
| WNT5B | Wnt Family Member 5B | Protein Coding | Q9H1J7 | 55 | GC12P001529 |
| UBE3C | Ubiquitin Protein Ligase E3C | Protein Coding | Q15386 | 53 | GC07P157138 |
| JAG2 | Jagged Canonical Notch Ligand 2 | Protein Coding | Q9Y219 | 57 | GC14M105140 |
| WT1 | WT1 Transcription Factor | Protein Coding | P19544 | 60 | GC11M032365 |
| UBR5 | Ubiquitin Protein Ligase E3 Component N-Recognin 5 | Protein Coding | O95071 | 54 | GC08M102252 |
| MACC1 | MET Transcriptional Regulator MACC1 | Protein Coding | Q6ZN28 | 45 | GC07M020140 |
| KLF6 | KLF Transcription Factor 6 | Protein Coding | Q99612 | 56 | GC10M003779 |
| AURKA | Aurora Kinase A | Protein Coding | O14965 | 65 | GC20M056370 |
| IFT88 | Intraflagellar Transport 88 | Protein Coding | Q13099 | 53 | GC13P021420 |
| BAG3 | BAG Cochaperone 3 | Protein Coding | O95817 | 55 | GC10P119651 |
| ITGB6 | Integrin Subunit Beta 6 | Protein Coding | P18564 | 60 | GC02M160099 |
| TNC | Vitamin D Receptor | Protein Coding | P11473 | 62 | GC12M047841 |
| COL8A2 | Placenta Associated 8 | Protein Coding | Q9NZF1 | 43 | GC04M083090 |
| BMP2 | Tenascin C | Protein Coding | P24821 | 62 | GC09M115019 |
| SETD7 | Collagen Type VIII Alpha 2 Chain | Protein Coding | P25067 | 51 | GC01M036095 |
| MAGED1 | Bone Morphogenetic Protein 2 | Protein Coding | P12643 | 58 | GC20P006767 |
| ITGB3 | SET Domain Containing 7, Histone Lysine Methyltransferase | Protein Coding | Q8WTS6 | 56 | GC04M139495 |
| HES1 | MAGE Family Member D1 | Protein Coding | Q9Y5V3 | 53 | GC0XP051803 |
| COL8A1 | Integrin Subunit Beta 3 | Protein Coding | P05106 | 65 | GC17P165432 |
| CCL18 | C-C Motif Chemokine Receptor 2 | Protein Coding | P41597 | 58 | GC03P066708 |
| PTP4A2 | Transcription Factor Dp Family Member 3 | Protein Coding | Q5H9I0 | 38 | GC0XM133216 |
| CD36 | Hes Family BHLH Transcription Factor 1 | Protein Coding | Q14469 | 57 | GC03P194136 |
| IRS1 | Collagen Type VIII Alpha 1 Chain | Protein Coding | P27658 | 52 | GC03P099638 |
| PRMT1 | C-C Motif Chemokine Ligand 18 | Protein Coding | P55774 | 49 | GC17P036064 |
| KLF17 | Protein Tyrosine Phosphatase 4A2 | Protein Coding | Q12974 | 51 | GC01M037544 |
| CCL2 | CD36 Molecule (CD36 Blood Group) | Protein Coding | P16671 | 63 | GC07P080369 |
| MED28 | Insulin Receptor Substrate 1 | Protein Coding | P35568 | 60 | GC02M226731 |
| ABCG2 | Proprotein Convertase Subtilisin/Kexin Type 1 | Protein Coding | P29120 | 62 | GC05M096391 |
| IRF6 | Protein Arginine Methyltransferase 1 | Protein Coding | Q99873 | 62 | GC19P049675 |
| HRAS | KLF Transcription Factor 17 | Protein Coding | Q5JT82 | 46 | GC01P085572 |
| CNTN1 | C-C Motif Chemokine Ligand 2 | Protein Coding | P13500 | 62 | GC17P034255 |
| ROCK1 | Mediator Complex Subunit 28 | Protein Coding | Q9H204 | 47 | GC04P032211 |
| ELL3 | ATP Binding Cassette Subfamily G Member 2 (JR Blood Group) | Protein Coding | Q9UNQ0 | 62 | GC04M088090 |
| NTN1 | Interferon Regulatory Factor 6 | Protein Coding | O14896 | 56 | GC01M209785 |
| AQP9 | Nuclear Receptor Subfamily 2 Group C Member 2 | Protein Coding | P49116 | 57 | GC03P014947 |
| HOXB9 | Inhibitor Of Growth Family Member 4 | Protein Coding | Q9UNL4 | 50 | GC12M006650 |
| MSI2 | HRas Proto-Oncogene, GTPase | Protein Coding | P01112 | 65 | GC11M015781 |
| NUBPL | Contactin 1 | Protein Coding | Q12860 | 59 | GC12P040692 |
| ADIPOQ | Rho Associated Coiled-Coil Containing Protein Kinase 1 | Protein Coding | Q13464 | 62 | GC18M039163 |
| ITGA6 | Elongation Factor For RNA Polymerase II 3 | Protein Coding | Q9HB65 | 39 | GC15M049859 |
| HOXA10 | Netrin 1 | Protein Coding | O95631 | 58 | GC17P164218 |
| CCR6 | Aquaporin 9 | Protein Coding | O43315 | 55 | GC15P058138 |
| ESRP1 | TIMP Metallopeptidase Inhibitor 2 | Protein Coding | P16035 | 54 | GC17M078852 |
| FBXO11 | Angiopoietin Like 4 | Protein Coding | Q9BY76 | 56 | GC19P008363 |
| RNF8 | Homeobox B9 | Protein Coding | P17482 | 51 | GC17M048621 |
| FOXK2 | Musashi RNA Binding Protein 2 | Protein Coding | Q96DH6 | 50 | GC17P057255 |
| HMGB3 | NUBP Iron-Sulfur Cluster Assembly Factor, Mitochondrial | Protein Coding | Q8TB37 | 52 | GC14P031489 |
| CCR5 | Adiponectin, C1Q And Collagen Domain Containing | Protein Coding | Q15848 | 59 | GC03P186842 |
| PPARG | Integrin Subunit Alpha 6 | Protein Coding | P23229 | 62 | GC02P172245 |
| MICALL2 | Transforming Growth Factor Beta Receptor 1 | Protein Coding | P36897 | 66 | GC09P109923 |
| ACVR1 | Protein-L-Isoaspartate (D-Aspartate) O-Methyltransferase | Protein Coding | P22061 | 51 | GC06P149749 |
| IL18 | Homeobox A10 | Protein Coding | P31260 | 53 | GC07M028280 |
| HIC1 | C-C Motif Chemokine Receptor 6 | Protein Coding | P51684 | 56 | GC06P167111 |
| CLK2 | Epithelial Splicing Regulatory Protein 1 | Protein Coding | Q6NXG1 | 49 | GC08P094641 |
| NTRK2 | F-Box Protein 11 | Protein Coding | Q86XK2 | 53 | GC02M047789 |
| LIN28A | Ring Finger Protein 8 | Protein Coding | O76064 | 55 | GC06P190846 |
| CCNA2 | Forkhead Box K2 | Protein Coding | Q01167 | 46 | GC17P082519 |
| IL6R | Transforming Growth Factor Alpha | Protein Coding | P01135 | 59 | GC02M070447 |
| HDAC2 | Geminin DNA Replication Inhibitor | Protein Coding | O75496 | 57 | GC06P024779 |
| LRG1 | High Mobility Group Box 3 | Protein Coding | O15347 | 53 | GC0XP150980 |
| EPCAM | C-C Motif Chemokine Receptor 5 | Protein Coding | P51681 | 59 | GC03P066709 |
| TRPM8 | Peroxisome Proliferator Activated Receptor Gamma | Protein Coding | P37231 | 65 | GC03P012287 |
| SQSTM1 | MICAL Like 2 | Protein Coding | Q8IY33 | 46 | GC07M004092 |
| IL4 | Activin A Receptor Type 1 | Protein Coding | Q04771 | 64 | GC02M157736 |
| CKS2 | Interleukin 18 | Protein Coding | Q14116 | 56 | GC11M112143 |
| DDR1 | Telomerase Reverse Transcriptase | Protein Coding | O14746 | 64 | GC05M001253 |
| SPHK1 | Protocadherin 9 | Protein Coding | Q9HC56 | 53 | GC13M066302 |
| FAS | HIC ZBTB Transcriptional Repressor 1 | Protein Coding | Q14526 | 53 | GC17P002054 |
| GSN | CDC Like Kinase 2 | Protein Coding | P49760 | 55 | GC01M155262 |
| BATF2 | Neurotrophic Receptor Tyrosine Kinase 2 | Protein Coding | Q16620 | 66 | GC09P084668 |
| ZYX | Lin-28 Homolog A | Protein Coding | Q9H9Z2 | 54 | GC01P026410 |
| HDAC3 | Cyclin A2 | Protein Coding | P20248 | 58 | GC04M121816 |
| IGFBP3 | Interleukin 6 Receptor | Protein Coding | P08887 | 63 | GC01P154405 |
| UHRF1 | PBX Homeobox 3 | Protein Coding | P40426 | 50 | GC09P125747 |
| CTHRC1 | Histone Deacetylase 2 | Protein Coding | Q92769 | 66 | GC06M113933 |
| ZNF217 | Leucine Rich Alpha-2-Glycoprotein 1 | Protein Coding | P02750 | 49 | GC19M111669 |
| MCRIP1 | Epithelial Cell Adhesion Molecule | Protein Coding | P16422 | 61 | GC02P047345 |
| CIRBP | Transient Receptor Potential Cation Channel Subfamily M Member 8 | Protein Coding | Q7Z2W7 | 58 | GC02P233917 |
| PCGF2 | Sequestosome 1 | Protein Coding | Q13501 | 62 | GC05P179806 |
| NRP2 | Interleukin 4 | Protein Coding | P05112 | 59 | GC05P132673 |
| IRGM | Par-6 Family Cell Polarity Regulator Alpha | Protein Coding | Q9NPB6 | 50 | GC16P129773 |
| HIPK2 | CDC28 Protein Kinase Regulatory Subunit 2 | Protein Coding | P33552 | 49 | GC09P089311 |
| MGLL | Discoidin Domain Receptor Tyrosine Kinase 1 | Protein Coding | Q08345 | 59 | GC06P190691 |
| ZNF143 | Sphingosine Kinase 1 | Protein Coding | Q9NYA1 | 59 | GC17P076376 |
| RUNX3 | Fas Cell Surface Death Receptor | Protein Coding | P25445 | 63 | GC10P128620 |
| NANOS3 | Transcription Factor 4 | Protein Coding | P15884 | 59 | GC18M055222 |
| GRN | Neurogenin 3 | Protein Coding | Q9Y4Z2 | 50 | GC10M069571 |
| VSIG4 | Gelsolin | Protein Coding | P06396 | 61 | GC09P121201 |
| STC2 | Basic Leucine Zipper ATF-Like Transcription Factor 2 | Protein Coding | Q8N1L9 | 42 | GC11M064987 |
| IDH2 | Zyxin | Protein Coding | Q15942 | 56 | GC07P143381 |
| GPR32 | Histone Deacetylase 3 | Protein Coding | O15379 | 63 | GC05M141620 |
| EGLN3 | Insulin Like Growth Factor Binding Protein 3 | Protein Coding | P17936 | 59 | GC07M045912 |
| LOXL2 | Basigin (Ok Blood Group) | Protein Coding | P35613 | 57 | GC19P000571 |
| RUNX1 | Interleukin 22 | Protein Coding | Q9GZX6 | 55 | GC12M068248 |
| IDH1 | Ubiquitin Like With PHD And Ring Finger Domains 1 | Protein Coding | Q96T88 | 53 | GC19P161111 |
| ANG | Collagen Triple Helix Repeat Containing 1 | Protein Coding | Q96CG8 | 52 | GC08P103371 |
| PKP3 | Zinc Finger Protein 217 | Protein Coding | O75362 | 50 | GC20M053567 |
| LGALS1 | MAPK Regulated Corepressor Interacting Protein 1 | Protein Coding | C9JLW8 | 38 | GC17M102026 |
| MZF1 | Small Proline Rich Protein 2A | Protein Coding | P35326 | 43 | GC01M168536 |
| TNFSF11 | Peroxiredoxin 1 | Protein Coding | Q06830 | 62 | GC01M045943 |
| ID2 | Cold Inducible RNA Binding Protein | Protein Coding | Q14011 | 49 | GC19P001259 |
| GLI2 | Polycomb Group Ring Finger 2 | Protein Coding | P35227 | 51 | GC17M038733 |
| RASSF1 | Neuropilin 2 | Protein Coding | O60462 | 57 | GC02P205681 |
| JUN | Immunity Related GTPase M | Protein Coding | A1A4Y4 | 44 | GC05P150846 |
| ZFP36 | Secreted Phosphoprotein 1 | Protein Coding | P10451 | 57 | GC04P087975 |
| BHLHE40 | Homeodomain Interacting Protein Kinase 2 | Protein Coding | Q9H2X6 | 57 | GC07M139561 |
| IRF8 | Monoglyceride Lipase | Protein Coding | Q99685 | 57 | GC03M127689 |
| GJB1 | Zinc Finger Protein 143 | Protein Coding | P52747 | 49 | GC11P023124 |
| FSTL1 | RUNX Family Transcription Factor 3 | Protein Coding | Q13761 | 56 | GC01M024899 |
| ITGB1 | Nanos C2HC-Type Zinc Finger 3 | Protein Coding | P60323 | 47 | GC19P013862 |
| WNT11 | SKI Like Proto-Oncogene | Protein Coding | P12757 | 55 | GC03P170357 |
| LGR5 | Opioid Receptor Mu 1 | Protein Coding | P35372 | 62 | GC06P192519 |
| RAB43 | Granulin Precursor | Protein Coding | P28799 | 60 | GC17P044345 |
| NOX1 | V-Set And Immunoglobulin Domain Containing 4 | Protein Coding | Q9Y279 | 48 | GC0XM066021 |
| PTP4A3 | Stanniocalcin 2 | Protein Coding | O76061 | 53 | GC05M173314 |
| HDAC1 | Isocitrate Dehydrogenase (NADP(+)) 2 | Protein Coding | P48735 | 65 | GC15M090083 |
| WNT6 | SMAD Specific E3 Ubiquitin Protein Ligase 2 | Protein Coding | Q9HAU4 | 55 | GC17M064542 |
| HAVCR2 | G Protein-Coupled Receptor 32 | Protein Coding | O75388 | 39 | GC19P050770 |
| HTN1 | Egl-9 Family Hypoxia Inducible Factor 3 | Protein Coding | Q9H6Z9 | 59 | GC14M033924 |
| LRIG1 | Lysyl Oxidase Like 2 | Protein Coding | Q9Y4K0 | 59 | GC08M023296 |
| GREM1 | RUNX Family Transcription Factor 1 | Protein Coding | Q01196 | 62 | GC21M034787 |
| VTN | Isocitrate Dehydrogenase (NADP(+)) 1 | Protein Coding | O75874 | 65 | GC02M208236 |
| AFAP1L2 | Secreted Frizzled Related Protein 2 | Protein Coding | Q96HF1 | 53 | GC04M153780 |
| HSP90AA1 | Angiogenin | Protein Coding | P03950 | 57 | GC14P060243 |
| SLC39A6 | Plakophilin 3 | Protein Coding | Q9Y446 | 49 | GC11P022599 |
| FOXN3 | Galectin 1 | Protein Coding | P09382 | 56 | GC22P037675 |
| KDM1A | Myeloid Zinc Finger 1 | Protein Coding | P28698 | 44 | GC19M113215 |
| VSNL1 | TNF Superfamily Member 11 | Protein Coding | O14788 | 62 | GC13P042562 |
| DYRK2 | Inhibitor Of DNA Binding 2 | Protein Coding | Q02363 | 56 | GC02P008678 |
| HSPB2 | C-X-C Motif Chemokine Ligand 5 | Protein Coding | P42830 | 53 | GC04M073995 |
| FZD2 | GLI Family Zinc Finger 2 | Protein Coding | P10070 | 60 | GC02P120735 |
| PIM2 | Ras Association Domain Family Member 1 | Protein Coding | Q9NS23 | 56 | GC03M050329 |
| AGER | Jun Proto-Oncogene, AP-1 Transcription Factor Subunit | Protein Coding | P05412 | 63 | GC01M058780 |
| VHL | ZFP36 Ring Finger Protein | Protein Coding | P26651 | 50 | GC19P039406 |
| PITPNM3 | Basic Helix-Loop-Helix Family Member E40 | Protein Coding | O14503 | 53 | GC03P005086 |
| BIRC2 | Interferon Regulatory Factor 8 | Protein Coding | Q02556 | 59 | GC16P130495 |
| FUT4 | C-C Motif Chemokine Ligand 5 | Protein Coding | P13501 | 56 | GC17M035871 |
| EHD1 | Nucleobindin 2 | Protein Coding | P80303 | 50 | GC11P023500 |
| NUMB | Gap Junction Protein Beta 1 | Protein Coding | P08034 | 60 | GC0XP071212 |
| VCP | Follistatin Like 1 | Protein Coding | Q12841 | 53 | GC03M120392 |
| AXIN2 | Integrin Subunit Beta 1 | Protein Coding | P05556 | 63 | GC10M037521 |
| HRG | Wnt Family Member 11 | Protein Coding | O96014 | 56 | GC11M076186 |
| BRD4 | Leucine Rich Repeat Containing G Protein-Coupled Receptor 5 | Protein Coding | O75473 | 56 | GC12P071439 |
| PPARGC1A | RAB43, Member RAS Oncogene Family | Protein Coding | Q86YS6 | 45 | GC03M132637 |
| WNT1 | Brain Derived Neurotrophic Factor | Protein Coding | P23560 | 62 | GC11M027654 |
| UCP2 | Receptor Tyrosine Kinase Like Orphan Receptor 1 | Protein Coding | Q01973 | 56 | GC01P063774 |
| AXIN1 | NADPH Oxidase 1 | Protein Coding | Q9Y5S8 | 57 | GC0XM100843 |
| HPGD | Protein Tyrosine Phosphatase 4A3 | Protein Coding | O75365 | 54 | GC08P141391 |
| SIRT3 | Histone Deacetylase 1 | Protein Coding | Q13547 | 64 | GC01P032292 |
| PGRMC1 | Wnt Family Member 6 | Protein Coding | Q9Y6F9 | 53 | GC02P218859 |
| EZR | Hepatitis A Virus Cellular Receptor 2 | Protein Coding | Q8TDQ0 | 58 | GC05M157063 |
| UCHL3 | Histatin 1 | Protein Coding | P15515 | 44 | GC04P070191 |
| NCOA3 | BCL2 Apoptosis Regulator | Protein Coding | P10415 | 64 | GC18M063123 |
| HOXD9 | Neurotrophic Receptor Tyrosine Kinase 3 | Protein Coding | Q16288 | 66 | GC15M087859 |
| DICER1 | Leucine Rich Repeats And Immunoglobulin Like Domains 1 | Protein Coding | Q96JA1 | 52 | GC03M066379 |
| HPSE | Gremlin 1, DAN Family BMP Antagonist | Protein Coding | O60565 | 58 | GC15P201431 |
| TLR4 | Vitronectin | Protein Coding | P04004 | 57 | GC17M100467 |
| TYRO3 | Actin Filament Associated Protein 1 Like 2 | Protein Coding | Q8N4X5 | 48 | GC10M114281 |
| ACTN4 | Heat Shock Protein 90 Alpha Family Class A Member 1 | Protein Coding | P07900 | 65 | GC14M102080 |
| APBB1 | RB Transcriptional Corepressor 1 | Protein Coding | P06400 | 62 | GC13P048303 |
| SATB2 | Natriuretic Peptide A | Protein Coding | P01160 | 57 | GC01M036050 |
| CCR9 | Solute Carrier Family 39 Member 6 | Protein Coding | Q13433 | 53 | GC18M036108 |
| TCF3 | Forkhead Box N3 | Protein Coding | O00409 | 47 | GC14M125316 |
| TYMS | Lysine Demethylase 1A | Protein Coding | O60341 | 62 | GC01P023019 |
| ESRP2 | Visinin Like 1 | Protein Coding | P62760 | 55 | GC02P017539 |
| HOXA9 | Dual Specificity Tyrosine Phosphorylation Regulated Kinase 2 | Protein Coding | Q92630 | 57 | GC12P081965 |
| BOP1 | Heat Shock Protein Family B (Small) Member 2 | Protein Coding | Q16082 | 52 | GC11P116385 |
| MTHFD2 | Protein Tyrosine Phosphatase Non-Receptor Type 11 | Protein Coding | Q06124 | 67 | GC12P112418 |
| STK11 | Frizzled Class Receptor 2 | Protein Coding | Q14332 | 59 | GC17P044557 |
| TXN | Pim-2 Proto-Oncogene, Serine/Threonine Kinase | Protein Coding | Q9P1W9 | 58 | GC0XM048913 |
| PEAK1 | Advanced Glycosylation End-Product Specific Receptor | Protein Coding | Q15109 | 59 | GC06M032180 |
| HNRNPAB | Von Hippel-Lindau Tumor Suppressor | Protein Coding | P40337 | 60 | GC03P028787 |
| EPB41L3 | PITPNM Family Member 3 | Protein Coding | Q9BZ71 | 50 | GC17M006451 |
| KDM5B | Baculoviral IAP Repeat Containing 2 | Protein Coding | Q13490 | 61 | GC11P102347 |
| TUFM | Notch Receptor 4 | Protein Coding | Q99466 | 60 | GC06M111570 |
| CA9 | Fucosyltransferase 4 | Protein Coding | P22083 | 53 | GC11P116008 |
| NR4A1 | EH Domain Containing 1 | Protein Coding | Q9H4M9 | 53 | GC11M142939 |
| KDM4B | NUMB Endocytic Adaptor Protein | Protein Coding | P49757 | 58 | GC14M073275 |
| GNA13 | Valosin Containing Protein | Protein Coding | P55072 | 62 | GC09M035946 |
| CCL21 | Axin 2 | Protein Coding | Q9Y2T1 | 62 | GC17M065528 |
| C5AR1 | Histidine Rich Glycoprotein | Protein Coding | P04196 | 55 | GC03P186660 |
| YWHAZ | Notch Receptor 3 | Protein Coding | Q9UM47 | 64 | GC19M015159 |
| HK2 | Bromodomain Containing 4 | Protein Coding | O60885 | 60 | GC19M112059 |
| FGF9 | PPARG Coactivator 1 Alpha | Protein Coding | Q9UBK2 | 60 | GC04M023755 |
| LEFTY1 | Wnt Family Member 1 | Protein Coding | P04628 | 59 | GC12P081263 |
| CCL20 | Uncoupling Protein 2 | Protein Coding | P55851 | 58 | GC11M073974 |
| PHLDA2 | Axin 1 | Protein Coding | O15169 | 60 | GC16M000287 |
| KDM6A | 15-Hydroxyprostaglandin Dehydrogenase | Protein Coding | P15428 | 58 | GC04M174490 |
| HIP1 | Cell Migration Inducing Hyaluronidase 1 | Protein Coding | Q8WUJ3 | 48 | GC15P080779 |
| FGF1 | NME/NM23 Nucleoside Diphosphate Kinase 1 | Protein Coding | P15531 | 60 | GC17P165542 |
| PDPN | Sirtuin 3 | Protein Coding | Q9NTG7 | 60 | GC11M000215 |
| LEF1 | Progesterone Receptor Membrane Component 1 | Protein Coding | O00264 | 57 | GC0XP119236 |
| TSC2 | Ezrin | Protein Coding | P15311 | 59 | GC06M158765 |
| TUFT1 | Ubiquitin C-Terminal Hydrolase L3 | Protein Coding | P15374 | 56 | GC13P075591 |
| EHD2 | Nuclear Receptor Coactivator 3 | Protein Coding | Q9Y6Q9 | 61 | GC20P047501 |
| ETV4 | Homeobox D9 | Protein Coding | P28356 | 48 | GC02P176122 |
| CXCL13 | Spalt Like Transcription Factor 4 | Protein Coding | Q9UJQ4 | 56 | GC20M051784 |
| PAX2 | NK6 Homeobox 1 | Protein Coding | P78426 | 52 | GC04M084491 |
| TSC1 | Dicer 1, Ribonuclease III | Protein Coding | Q9UPY3 | 61 | GC14M095086 |
| TPBG | Heparanase | Protein Coding | Q9Y251 | 57 | GC04M083292 |
| ANXA5 | Toll Like Receptor 4 | Protein Coding | O00206 | 63 | GC09P117704 |
| ESRRA | TYRO3 Protein Tyrosine Kinase | Protein Coding | Q06418 | 60 | GC15P041557 |
| FBLN5 | Actinin Alpha 4 | Protein Coding | O43707 | 59 | GC19P038647 |
| DDR2 | Amyloid Beta Precursor Protein Binding Family B Member 1 | Protein Coding | O00213 | 56 | GC11M016043 |
| TP53BP2 | Transmembrane Serine Protease 4 | Protein Coding | Q9NRS4 | 52 | GC11P118077 |
| ESR2 | Nuclear Factor, Interleukin 3 Regulated | Protein Coding | Q16649 | 47 | GC09M126468 |
| CEACAM5 | SATB Homeobox 2 | Protein Coding | Q9UPW6 | 57 | GC02M199269 |
| YBX1 | C-C Motif Chemokine Receptor 9 | Protein Coding | P51686 | 51 | GC03P066697 |
| TRPC5 | Transcription Factor 3 | Protein Coding | P15923 | 59 | GC19M001609 |
| TLE1 | Thymidylate Synthetase | Protein Coding | P04818 | 63 | GC18P000657 |
| ANXA1 | Epithelial Splicing Regulatory Protein 2 | Protein Coding | Q9H6T0 | 47 | GC16M068229 |
| ERG | Homeobox A9 | Protein Coding | P31269 | 51 | GC07M027162 |
| FST | Mitogen-Activated Protein Kinase 8 | Protein Coding | P45983 | 63 | GC10P048306 |
| MCAM | Nuclear Factor I C | Protein Coding | P08651 | 52 | GC19P003314 |
| ACTG2 | BOP1 Ribosomal Biogenesis Factor | Protein Coding | Q14137 | 46 | GC08M144262 |
| TIMP1 | Methylenetetrahydrofolate Dehydrogenase (NADP+ Dependent) 2, Methenyltetrahydrofolate Cyclohydrolase | Protein Coding | P13995 | 55 | GC02P074186 |
| REPIN1 | Serine/Threonine Kinase 11 | Protein Coding | Q15831 | 62 | GC19P001177 |
| EPS8 | Thioredoxin | Protein Coding | P10599 | 59 | GC09M110243 |
| MAD2L2 | Pseudopodium Enriched Atypical Kinase 1 | Protein Coding | Q9H792 | 44 | GC15M077100 |
| TP53BP1 | Heterogeneous Nuclear Ribonucleoprotein A/B | Protein Coding | Q99729 | 49 | GC05P178204 |
| TIAM1 | Protein Kinase C Alpha | Protein Coding | P17252 | 65 | GC17P066302 |
| OLA1 | Myosin Light Chain 2 | Protein Coding | P10916 | 61 | GC12M112099 |
| EPO | Erythrocyte Membrane Protein Band 4.1 Like 3 | Protein Coding | Q9Y2J2 | 53 | GC18M005392 |
| TAB1 | Lysine Demethylase 5B | Protein Coding | Q9UGL1 | 57 | GC01M202696 |
| TNXB | Tu Translation Elongation Factor, Mitochondrial | Protein Coding | P49411 | 57 | GC16M053150 |
| TGFB1I1 | Carbonic Anhydrase 9 | Protein Coding | Q16790 | 59 | GC09P035673 |
| CARD10 | Nuclear Receptor Subfamily 4 Group A Member 1 | Protein Coding | P22736 | 60 | GC12P052022 |
| EPHB2 | MYD88 Innate Immune Signal Transduction Adaptor | Protein Coding | Q99836 | 61 | GC03P042445 |
| RACK1 | Lysine Demethylase 4B | Protein Coding | O94953 | 58 | GC19P004969 |
| TNS1 | G Protein Subunit Alpha 13 | Protein Coding | Q14344 | 55 | GC17M065009 |
| TEAD1 | C-C Motif Chemokine Ligand 21 | Protein Coding | O00585 | 55 | GC09M034709 |
| GSK3A | Complement C5a Receptor 1 | Protein Coding | P21730 | 59 | GC19P047290 |
| GKN2 | Tyrosine 3-Monooxygenase/Tryptophan 5-Monooxygenase Activation Protein Zeta | Protein Coding | P63104 | 61 | GC08M100991 |
| PAK4 | Hexokinase 2 | Protein Coding | P52789 | 59 | GC02P075619 |
| TLN1 | Taurine Up-Regulated 1 | Protein Coding | A0A6I8PU40 | 33 | GC22P030969 |
| TCF21 | MYCN Proto-Oncogene, BHLH Transcription Factor | Protein Coding | P04198 | 59 | GC02P016028 |
| BRD7 | Fibroblast Growth Factor 9 | Protein Coding | P31371 | 56 | GC13P021671 |
| TET3 | Left-Right Determination Factor 1 | Protein Coding | O75610 | 52 | GC01M227879 |
| CDKN1A | C-C Motif Chemokine Ligand 20 | Protein Coding | P78556 | 55 | GC02P227846 |
| TLE4 | Pleckstrin Homology Like Domain Family A Member 2 | Protein Coding | Q53GA4 | 49 | GC11M002928 |
| TBK1 | Lysine Demethylase 6A | Protein Coding | O15550 | 60 | GC0XP044873 |
| AHR | Huntingtin Interacting Protein 1 | Protein Coding | O00291 | 55 | GC07M075533 |
| SPRY1 | Semaphorin 4C | Protein Coding | Q9C0C4 | 44 | GC02M096859 |
| TJP1 | MYB Proto-Oncogene Like 2 | Protein Coding | P10244 | 55 | GC20P043667 |
| FHOD1 | Fibroblast Growth Factor 1 | Protein Coding | P05230 | 60 | GC05M142555 |
| E2F1 | Podoplanin | Protein Coding | Q86YL7 | 55 | GC01P013583 |
| MSLN | Lymphoid Enhancer Binding Factor 1 | Protein Coding | Q9UJU2 | 62 | GC04M108047 |
| TIMP3 | TSC Complex Subunit 2 | Protein Coding | P49815 | 64 | GC16P128054 |
| MAP3K7 | Tuftelin 1 | Protein Coding | Q9NNX1 | 50 | GC01P179795 |
| GRIN1 | EH Domain Containing 2 | Protein Coding | Q9NZN4 | 48 | GC19P047713 |
| HBEGF | Peptidylprolyl Cis/Trans Isomerase, NIMA-Interacting 1 | Protein Coding | Q13526 | 60 | GC19P009835 |
| PSME3 | MYB Proto-Oncogene, Transcription Factor | Protein Coding | P10242 | 62 | GC06P135181 |
| NR2F2 | ETS Variant Transcription Factor 4 | Protein Coding | P43268 | 55 | GC17M043527 |
| STAT5B | C-X-C Motif Chemokine Ligand 13 | Protein Coding | O43927 | 53 | GC04P077511 |
| ANPEP | Paired Box 2 | Protein Coding | Q02962 | 58 | GC10P100735 |
| DLX4 | TSC Complex Subunit 1 | Protein Coding | Q92574 | 61 | GC09M132891 |
| LOX | Trophoblast Glycoprotein | Protein Coding | Q13641 | 54 | GC06P191220 |
| TFCP2 | Annexin A5 | Protein Coding | P08758 | 59 | GC04M121667 |
| STAT5A | Placental Growth Factor | Protein Coding | P49763 | 56 | GC14M074941 |
| DAB2IP | Tripartite Motif Containing 37 | Protein Coding | O94972 | 53 | GC17M101477 |
| CDK3 | Estrogen Related Receptor Alpha | Protein Coding | P11474 | 59 | GC11P064305 |
| LCN2 | Fibulin 5 | Protein Coding | Q9UBX5 | 59 | GC14M091869 |
| STAT1 | Discoidin Domain Receptor Tyrosine Kinase 2 | Protein Coding | Q16832 | 63 | GC01P180385 |
| LAMA1 | Tumor Protein P53 Binding Protein 2 | Protein Coding | Q13625 | 50 | GC01M223779 |
| PAQR3 | Serpin Family F Member 1 | Protein Coding | P36955 | 57 | GC17P163867 |
| G3BP1 | Mucin 2, Oligomeric Mucus/Gel-Forming | Protein Coding | Q02817 | 52 | GC11P001074 |
| IL17A | Estrogen Receptor 2 | Protein Coding | Q92731 | 61 | GC14M064084 |
| BRAF | CEA Cell Adhesion Molecule 5 | Protein Coding | P06731 | 56 | GC19P162154 |
| CAVIN1 | Y-Box Binding Protein 1 | Protein Coding | P67809 | 54 | GC01P042682 |
| CTSZ | Transient Receptor Potential Cation Channel Subfamily C Member 5 | Protein Coding | Q9UL62 | 54 | GC0XM111774 |
| TRAP1 | TLE Family Member 1, Transcriptional Corepressor | Protein Coding | Q04724 | 56 | GC09M081583 |
| IL1B | Annexin A1 | Protein Coding | P04083 | 60 | GC09P073151 |
| SRI | Golgi Membrane Protein 1 | Protein Coding | Q8NBJ4 | 52 | GC09M086026 |
| GPI | Macrophage Stimulating 1 Receptor | Protein Coding | Q04912 | 62 | GC03M055066 |
| GLIPR2 | ETS Transcription Factor ERG | Protein Coding | P11308 | 59 | GC21M038367 |
| CDH13 | Follistatin | Protein Coding | P19883 | 61 | GC05P053480 |
| IGF1R | Melanoma Cell Adhesion Molecule | Protein Coding | P43121 | 54 | GC11M119308 |
| SPOCK1 | Actin Gamma 2, Smooth Muscle | Protein Coding | P63267 | 56 | GC02P073892 |
| GOLGA2 | TIMP Metallopeptidase Inhibitor 1 | Protein Coding | P01033 | 57 | GC0XP061588 |
| SLC30A7 | Replication Initiator 1 | Protein Coding | Q9BWE0 | 46 | GC07P150368 |
| CDH11 | Pro-Apoptotic WT1 Regulator | Protein Coding | Q96IZ0 | 54 | GC12M079574 |
| IGF1 | Moesin | Protein Coding | P26038 | 60 | GC0XP065588 |
| SOX5 | EGFR Pathway Substrate 8, Signaling Adaptor | Protein Coding | Q12929 | 56 | GC12M037119 |
| GLRX | Mitotic Arrest Deficient 2 Like 2 | Protein Coding | Q9UI95 | 55 | GC01M036044 |
| RBFOX3 | Tumor Protein P53 Binding Protein 1 | Protein Coding | Q12888 | 56 | GC15M043403 |
| NR1H3 | TIAM Rac1 Associated GEF 1 | Protein Coding | Q13009 | 57 | GC21M031118 |
| HSF1 | Obg Like ATPase 1 | Protein Coding | Q9NTK5 | 53 | GC02M174072 |
| TCF7 | Erythropoietin | Protein Coding | P01588 | 55 | GC07P100720 |
| BMP6 | TGF-Beta Activated Kinase 1 (MAP3K7) Binding Protein 1 | Protein Coding | Q15750 | 55 | GC22P097418 |
| GLS | Tenascin XB | Protein Coding | P22105 | 56 | GC06M111563 |
| CSNK2B | Transforming Growth Factor Beta 1 Induced Transcript 1 | Protein Coding | O43294 | 53 | GC16P128992 |
| MICA | Caspase Recruitment Domain Family Member 10 | Protein Coding | Q9BWT7 | 49 | GC22M088429 |
| HNF4A | EPH Receptor B2 | Protein Coding | P29323 | 65 | GC01P022710 |
| SKP2 | Receptor For Activated C Kinase 1 | Protein Coding | P63244 | 56 | GC05M183748 |
| EML4 | Tensin 1 | Protein Coding | Q9HBL0 | 52 | GC02M217799 |
| CRYAB | TEA Domain Transcription Factor 1 | Protein Coding | P28347 | 58 | GC11P012674 |
| OCLN | Glycogen Synthase Kinase 3 Alpha | Protein Coding | P49840 | 63 | GC19M112567 |
| FOXA1 | Nodal Growth Differentiation Factor | Protein Coding | Q96S42 | 54 | GC10M070431 |
| TBX3 | Gastrokine 2 | Protein Coding | Q86XP6 | 44 | GC02M068945 |
| SIM2 | P21 (RAC1) Activated Kinase 4 | Protein Coding | O96013 | 61 | GC19P039125 |
| GLO1 | Talin 1 | Protein Coding | Q9Y490 | 56 | GC09M035696 |
| DNAJB6 | Transcription Factor 21 | Protein Coding | O43680 | 48 | GC06P133889 |
| FOXC2 | Bromodomain Containing 7 | Protein Coding | Q9NPI1 | 54 | GC16M050313 |
| TBX2 | ATM Serine/Threonine Kinase | Protein Coding | Q13315 | 66 | GC11P108222 |
| SDC2 | Matrix Metallopeptidase 19 | Protein Coding | Q99542 | 59 | GC12M055835 |
| GLS2 | Tet Methylcytosine Dioxygenase 3 | Protein Coding | O43151 | 53 | GC02P074280 |
| CRK | Cyclin Dependent Kinase Inhibitor 1A | Protein Coding | P38936 | 63 | GC06P190835 |
| ERBB2 | TLE Family Member 4, Transcriptional Corepressor | Protein Coding | Q04727 | 53 | GC09P079571 |
| CCL19 | TANK Binding Kinase 1 | Protein Coding | Q9UHD2 | 61 | GC12P081858 |
| AGO2 | Mucin 4, Cell Surface Associated | Protein Coding | Q99102 | 52 | GC03M195746 |
| CREB1 | Matrix Metallopeptidase 13 | Protein Coding | P45452 | 62 | GC11M102942 |
| CDH5 | Aryl Hydrocarbon Receptor | Protein Coding | P35869 | 60 | GC07P016916 |
| CLU | Sprouty RTK Signaling Antagonist 1 | Protein Coding | O43609 | 53 | GC04P123396 |
| HDAC5 | Tight Junction Protein 1 | Protein Coding | Q07157 | 58 | GC15M029699 |
| CLDN7 | Formin Homology 2 Domain Containing 1 | Protein Coding | Q9Y613 | 48 | GC16M067931 |
| FRAT1 | Matrix Metallopeptidase 7 | Protein Coding | P09237 | 60 | GC11M143590 |
| UHRF2 | Matrix Metallopeptidase 11 | Protein Coding | P24347 | 57 | GC22P023768 |
| CLDN4 | E2F Transcription Factor 1 | Protein Coding | Q01094 | 57 | GC20M033675 |
| PDCD6IP | Mesothelin | Protein Coding | Q13421 | 56 | GC16P128004 |
| MIR655 | TIMP Metallopeptidase Inhibitor 3 | Protein Coding | P35625 | 56 | GC22P097244 |
| LINC-ROR | Mitogen-Activated Protein Kinase Kinase Kinase 7 | Protein Coding | O43318 | 62 | GC06M090513 |
| MIR300 | Glutamate Ionotropic Receptor NMDA Type Subunit 1 | Protein Coding | Q05586 | 63 | GC09P137138 |
| MIR487B | Forkhead Box O4 | Protein Coding | P98177 | 55 | GC0XP071095 |
| MIR630 | Matrix Metallopeptidase 8 | Protein Coding | P22894 | 60 | GC11M143591 |
| MIR590 | Heparin Binding EGF Like Growth Factor | Protein Coding | Q99075 | 57 | GC05M140332 |
| MIR200C | Proteasome Activator Subunit 3 | Protein Coding | P61289 | 51 | GC17P042824 |
| MIR506 | Nuclear Receptor Subfamily 2 Group F Member 2 | Protein Coding | P24468 | 61 | GC15P096325 |
| MIR497 | Signal Transducer And Activator Of Transcription 5B | Protein Coding | P51692 | 62 | GC17M042199 |
| DUXAP9 | Alanyl Aminopeptidase, Membrane | Protein Coding | P15144 | 62 | GC15M089784 |
| MIR494 | Mitogen-Activated Protein Kinase Kinase Kinase 4 | Protein Coding | Q9Y6R4 | 54 | GC06P160991 |
| MIR200B | MLLT3 Super Elongation Complex Subunit | Protein Coding | P42568 | 51 | GC09M020341 |
| NPTN-IT1 | Distal-Less Homeobox 4 | Protein Coding | Q92988 | 51 | GC17P049968 |
| MIR489 | Lysyl Oxidase | Protein Coding | P28300 | 60 | GC05M122063 |
| SPRY4-IT1 | Transcription Factor CP2 | Protein Coding | Q12800 | 53 | GC12M051093 |
| MIR424 | Signal Transducer And Activator Of Transcription 5A | Protein Coding | P42229 | 63 | GC17P042287 |
| MIR200A | Mitogen-Activated Protein Kinase Kinase Kinase 3 | Protein Coding | Q99759 | 59 | GC17P063622 |
| ZEB2-AS1 | Achaete-Scute Family BHLH Transcription Factor 2 | Protein Coding | Q99929 | 45 | GC11M015868 |
| MIR382 | DAB2 Interacting Protein | Protein Coding | Q5VWQ8 | 52 | GC09P155579 |
| MIR1236 | Cyclin Dependent Kinase 3 | Protein Coding | Q00526 | 48 | GC17P165999 |
| MIR381 | Lipocalin 2 | Protein Coding | P80188 | 58 | GC09P128149 |
| MIR21 | Signal Transducer And Activator Of Transcription 1 | Protein Coding | P42224 | 65 | GC02M190908 |
| MIR675 | Laminin Subunit Alpha 1 | Protein Coding | P25391 | 57 | GC18M006941 |
| NOV | MDM2 Proto-Oncogene | Protein Coding | Q00987 | 66 | GC12P068808 |
| MIR491 | Melanocyte Inducing Transcription Factor | Protein Coding | O75030 | 61 | GC03P069740 |
| MIR485 | Progestin And AdipoQ Receptor Family Member 3 | Protein Coding | Q6TCH7 | 44 | GC04M078887 |
| MKL1 | G3BP Stress Granule Assembly Factor 1 | Protein Coding | Q13283 | 53 | GC05P151771 |
| MEG3 | Interleukin 17A | Protein Coding | Q16552 | 56 | GC06P052186 |
| MIR375 | B-Raf Proto-Oncogene, Serine/Threonine Kinase | Protein Coding | P15056 | 67 | GC07M140798 |
| MIR345 | Caveolae Associated Protein 1 | Protein Coding | Q6NZI2 | 53 | GC17M100983 |
| MIR361 | C-X-C Motif Chemokine Ligand 9 | Protein Coding | Q07325 | 52 | GC04M076001 |
| MIR331 | Cathepsin Z | Protein Coding | Q9UBR2 | 56 | GC20M059013 |
| MIR326 | TNF Receptor Associated Protein 1 | Protein Coding | Q12931 | 57 | GC16M052311 |
| MIR9-1 | Interleukin 1 Beta | Protein Coding | P01584 | 60 | GC02M112829 |
| HAS2-AS1 | Sorcin | Protein Coding | P30626 | 53 | GC07M088205 |
| SLC9A3R1 | Glucose-6-Phosphate Isomerase | Protein Coding | P06744 | 59 | GC19P034359 |
| MIR30C2 | Myocyte Enhancer Factor 2D | Protein Coding | Q14814 | 57 | GC01M156463 |
| MIR30C1 | GLI Pathogenesis Related 2 | Protein Coding | Q9H4G4 | 46 | GC09P078087 |
| CTGF | Cadherin 13 | Protein Coding | P55290 | 57 | GC16P082626 |
| MIR29C | Insulin Like Growth Factor 1 Receptor | Protein Coding | P08069 | 68 | GC15P098648 |
| MIR29B1 | SPARC (Osteonectin), Cwcv And Kazal Like Domains Proteoglycan 1 | Protein Coding | Q08629 | 52 | GC05M136975 |
| MIR26A1 | Golgin A2 | Protein Coding | Q08379 | 55 | GC09M128255 |
| ZEB1-AS1 | MDM4 Regulator Of P53 | Protein Coding | O15151 | 59 | GC01P204516 |
| MIR96 | Solute Carrier Family 30 Member 7 | Protein Coding | Q8NEW0 | 51 | GC01P100896 |
| MIR137 | Cadherin 11 | Protein Coding | P55287 | 59 | GC16M064943 |
| MIR31 | Insulin Like Growth Factor 1 | Protein Coding | P05019 | 60 | GC12M102395 |
| MIR130B | SRY-Box Transcription Factor 5 | Protein Coding | P35711 | 56 | GC12M023529 |
| MIR34A | Glutaredoxin | Protein Coding | P35754 | 56 | GC05M095752 |
| MIR222 | ADP-Ribosyltransferase 1 | Protein Coding | P52961 | 45 | GC11P022823 |
| MIR10B | RNA Binding Fox-1 Homolog 3 | Protein Coding | A6NFN3 | 50 | GC17M079089 |
| MIR196B | Nuclear Receptor Subfamily 1 Group H Member 3 | Protein Coding | Q13133 | 59 | GC11P047248 |
| MIR218-1 | Heat Shock Transcription Factor 1 | Protein Coding | Q00613 | 60 | GC08P144291 |
| MIR106B | Transcription Factor 7 | Protein Coding | P36402 | 55 | GC05P135165 |
| MIR373 | Bone Morphogenetic Protein 6 | Protein Coding | P22004 | 57 | GC06P007726 |
| MIR181A1 | Glutaminase | Protein Coding | O94925 | 62 | GC02P190880 |
| MIR106A | SMAD Family Member 9 | Protein Coding | O15198 | 58 | GC13M036844 |
| MIR135B | Casein Kinase 2 Beta | Protein Coding | P67870 | 61 | GC06P190711 |
| MIR194-1 | MHC Class I Polypeptide-Related Sequence A | Protein Coding | Q29983 | 51 | GC06P031399 |
| MIRLET7A1 | Hepatocyte Nuclear Factor 4 Alpha | Protein Coding | P41235 | 62 | GC20P044355 |
| ZFAS1 | S-Phase Kinase Associated Protein 2 | Protein Coding | Q13309 | 57 | GC05P036151 |
| MIR185 | EMAP Like 4 | Protein Coding | Q9HC35 | 53 | GC02P042169 |
| MIR7-1 | Arrestin Beta 1 | Protein Coding | P49407 | 57 | GC11M143283 |
| MIR182 | Crystallin Alpha B | Protein Coding | P02511 | 59 | GC11M111908 |
| MIR30E | Occludin | Protein Coding | Q16625 | 57 | GC05P069492 |
| AOC4P | Forkhead Box A1 | Protein Coding | P55317 | 57 | GC14M037589 |
| MIR181A2 | T-Box Transcription Factor 3 | Protein Coding | O15119 | 56 | GC12M114670 |
| MIR30B | SIM BHLH Transcription Factor 2 | Protein Coding | Q14190 | 50 | GC21P036699 |
| MIR30A | Glyoxalase I | Protein Coding | Q04760 | 57 | GC06M111723 |
| MIR17 | DnaJ Heat Shock Protein Family (Hsp40) Member B6 | Protein Coding | O75190 | 53 | GC07P157335 |
| MIR302A | Forkhead Box C2 | Protein Coding | Q99958 | 57 | GC16P130554 |
| CYR61 | T-Box Transcription Factor 2 | Protein Coding | Q13207 | 59 | GC17P061399 |
| MIR205 | Syndecan 2 | Protein Coding | P34741 | 55 | GC08P096499 |
| MIR143 | Glutaminase 2 | Protein Coding | Q9UI32 | 55 | GC12M056470 |
| MIR29A | CRK Proto-Oncogene, Adaptor Protein | Protein Coding | P46108 | 57 | GC17M001420 |
| MKL2 | Erb-B2 Receptor Tyrosine Kinase 2 | Protein Coding | P04626 | 68 | GC17P039687 |
| MALAT1 | C-C Motif Chemokine Ligand 19 | Protein Coding | Q99731 | 53 | GC09M036136 |
| MIR130A | Argonaute RISC Catalytic Component 2 | Protein Coding | Q9UKV8 | 55 | GC08M141058 |
| MIR26A2 | CAMP Responsive Element Binding Protein 1 | Protein Coding | P16220 | 63 | GC02P207529 |
| MIR522 | Cadherin 5 | Protein Coding | P33151 | 60 | GC16P066366 |
| MIR128-1 | Clusterin | Protein Coding | P10909 | 60 | GC08M027596 |
| MIR25 | Histone Deacetylase 5 | Protein Coding | Q9UQL6 | 61 | GC17M044076 |
| MIR517C | Claudin 7 | Protein Coding | O95471 | 53 | GC17M007259 |
| MIR34C | FRAT Regulator Of Wnt Signaling Pathway 1 | Protein Coding | Q92837 | 46 | GC10P097319 |
| MIR10A | Ubiquitin Like With PHD And Ring Finger Domains 2 | Protein Coding | Q96PU4 | 45 | GC09P006413 |
| MIR24-2 | Claudin 4 | Protein Coding | O14493 | 53 | GC07P073799 |
| MIR520G | Programmed Cell Death 6 Interacting Protein | Protein Coding | Q8WUM4 | 56 | GC03P033798 |
| MIR33A | MicroRNA 655 | RNA Gene | 0 | 17 | GC14P122528 |
| MIR23B | Long Intergenic Non-Protein Coding RNA, Regulator Of Reprogramming | RNA Gene | 0 | 25 | GC18M057054 |
| MIR452 | MicroRNA 300 | RNA Gene | 0 | 16 | GC14P122495 |
| MIR30D | MicroRNA 487b | RNA Gene | 0 | 25 | GC14P122518 |
| MIR217 | MicroRNA 630 | RNA Gene | 0 | 16 | GC15P072587 |
| MIR301A | MicroRNA 590 | RNA Gene | 0 | 28 | GC07P074191 |
| MIR216A | MicroRNA 200c | RNA Gene | 0 | 30 | GC12P080296 |
| MIR27A | MicroRNA 506 | RNA Gene | 0 | 23 | GC0XM147230 |
| MIR211 | MicroRNA 497 | RNA Gene | 0 | 27 | GC17M099943 |
| MIR24-1 | Double Homeobox A Pseudogene 9 | Pseudogene | 0 | 17 | GC14P060080 |
| MIR208A | MicroRNA 494 | RNA Gene | 0 | 26 | GC14P122521 |
| MIR224 | MicroRNA 200b | RNA Gene | 0 | 29 | GC01P001167 |
| MIR221 | NPTN Intronic Transcript 1 | RNA Gene | 0 | 12 | GC15M073566 |
| MIR199A2 | MicroRNA 489 | RNA Gene | 0 | 26 | GC07M093483 |
| MIR223 | SPRY4 Intronic Transcript 1 | RNA Gene | 0 | 21 | GC05M142318 |
| MIR145 | MicroRNA 424 | RNA Gene | 0 | 27 | GC0XM135469 |
| MIR190A | MicroRNA 200a | RNA Gene | 0 | 28 | GC01P082902 |
| MIR218-2 | ZEB2 Antisense RNA 1 | RNA Gene | 0 | 29 | GC02P157816 |
| MIR181B1 | MicroRNA 382 | RNA Gene | 0 | 25 | GC14P122511 |
| MIR206 | MicroRNA 1236 | RNA Gene | 0 | 18 | GC06M111559 |
| MIR153-2 | MicroRNA 381 | RNA Gene | 0 | 27 | GC14P122509 |
| MIR20A | MicroRNA 21 | RNA Gene | 0 | 32 | GC17P059841 |
| MIR152 | MicroRNA 675 | RNA Gene | 0 | 27 | GC11M015854 |
| MIR199A1 | Cellular Communication Network Factor 3 | Protein Coding | P48745 | 52 | GC08P119416 |
| MIR149 | MicroRNA 491 | RNA Gene | 0 | 28 | GC09P020716 |
| MIR193A | MicroRNA 485 | RNA Gene | 0 | 26 | GC14P122516 |
| MIR144 | Myocardin Related Transcription Factor A | Protein Coding | Q969V6 | 52 | GC22M089265 |
| MIR191 | Maternally Expressed 3 | RNA Gene | 0 | 36 | GC14P122485 |
| NEAT1 | MicroRNA 375 | RNA Gene | 0 | 28 | GC02M219001 |
| MIR136 | MicroRNA 345 | RNA Gene | 0 | 27 | GC14P100307 |
| MIR187 | MicroRNA 361 | RNA Gene | 0 | 26 | GC0XM085903 |
| MIR129-1 | MicroRNA 331 | RNA Gene | 0 | 28 | GC12P095308 |
| MIR183 | MicroRNA 326 | RNA Gene | 0 | 30 | GC11M075335 |
| MIR125B1 | MicroRNA 9-1 | RNA Gene | 0 | 29 | GC01M156420 |
| MIR16-1 | HAS2 Antisense RNA 1 | RNA Gene | 0 | 26 | GC08P121639 |
| MIRLET7G | NHERF Family PDZ Scaffold Protein 1 | Protein Coding | O14745 | 56 | GC17P165946 |
| MIR449A | MicroRNA 30c-2 | RNA Gene | 0 | 21 | GC06M112111 |
| MIR15B | MicroRNA 30c-1 | RNA Gene | 0 | 29 | GC01P040757 |
| MIRLET7D | Cellular Communication Network Factor 2 | Protein Coding | P29279 | 59 | GC06M131948 |
| MIR15A | MicroRNA 29c | RNA Gene | 0 | 27 | GC01M207838 |
| MIRLET7B | MicroRNA 29b-1 | RNA Gene | 0 | 28 | GC07M130877 |
| CYTOR | MicroRNA 26a-1 | RNA Gene | 0 | 30 | GC03P037969 |
| MIR153-1 | ZEB1 Antisense RNA 1 | RNA Gene | 0 | 26 | GC10M031166 |
| RMST | MicroRNA 96 | RNA Gene | 0 | 29 | GC07M129774 |
| MIR146A | MicroRNA 137 | RNA Gene | 0 | 27 | GC01M098046 |
| MIR135A1 | MicroRNA 31 | RNA Gene | 0 | 28 | GC09M022373 |
| MIR134 | MicroRNA 130b | RNA Gene | 0 | 27 | GC22P101538 |
| MIR132 | MicroRNA 34a | RNA Gene | 0 | 30 | GC01M009151 |
| HOTAIR | MicroRNA 222 | RNA Gene | 0 | 28 | GC0XM045747 |
| USMG5 | MicroRNA 10b | RNA Gene | 0 | 30 | GC02P176150 |
| MIR124-3 | MicroRNA 196b | RNA Gene | 0 | 29 | GC07M028277 |
| MIR124-2 | MicroRNA 218-1 | RNA Gene | 0 | 26 | GC04P032246 |
| MIR122 | MicroRNA 106b | RNA Gene | 0 | 30 | GC07M107031 |
| MIR101-2 | MicroRNA 373 | RNA Gene | 0 | 28 | GC19P162649 |
| USP17L9P | MicroRNA 181a-1 | RNA Gene | 0 | 27 | GC01M198860 |
| MIR100 | MicroRNA 106a | RNA Gene | 0 | 26 | GC0XM135484 |
| WISP2 | MicroRNA 135b | RNA Gene | 0 | 29 | GC01M205448 |
| H19 | MicroRNA 194-1 | RNA Gene | 0 | 23 | GC01M220118 |
| MIR1271 | MicroRNA Let-7a-1 | RNA Gene | 0 | 30 | GC09P094175 |
| T | ZNFX1 Antisense RNA 1 | RNA Gene | 0 | 29 | GC20P049276 |
| MIR429 | MicroRNA 185 | RNA Gene | 0 | 30 | GC22P096776 |
| H2AFX | MicroRNA 7-1 | RNA Gene | 0 | 24 | GC09M126261 |
| MIR93 | MicroRNA 182 | RNA Gene | 0 | 30 | GC07M129770 |
| MIR204 | MicroRNA 30e | RNA Gene | 0 | 31 | GC01P040754 |
| MIR203A | Amine Oxidase Copper Containing 4, Pseudogene | Pseudogene | 0 | 18 | GC17P165243 |
| WISP3 | MicroRNA 181a-2 | RNA Gene | 0 | 28 | GC09P124692 |
| CPS1-IT1 | MicroRNA 30b | RNA Gene | 0 | 30 | GC08M134800 |
| MIR186 | MicroRNA 30a | RNA Gene | 0 | 27 | GC06M071403 |
| MIR155 | MicroRNA 17 | RNA Gene | 0 | 27 | GC13P091350 |
| MIR148A | MicroRNA 302a | RNA Gene | 0 | 28 | GC04M113209 |
| MIR802 | Cellular Communication Network Factor 1 | Protein Coding | O00622 | 52 | GC01P089746 |
| MIR454 | MicroRNA 205 | RNA Gene | 0 | 27 | GC01P209432 |
| BVES | MicroRNA 143 | RNA Gene | 0 | 31 | GC05P161156 |
| UCA1 | MicroRNA 29a | RNA Gene | 0 | 30 | GC07M130876 |
| MIR663A | Myocardin Related Transcription Factor B | Protein Coding | Q9ULH7 | 46 | GC16P013994 |
| H2AFZ | Metastasis Associated Lung Adenocarcinoma Transcript 1 | RNA Gene | 0 | 33 | GC11P115065 |
| MIR26B | MicroRNA 130a | RNA Gene | 0 | 30 | GC11P057641 |
| MIR23A | MicroRNA 26a-2 | RNA Gene | 0 | 27 | GC12M057824 |
| MIR214 | MicroRNA 522 | RNA Gene | 0 | 18 | GC19P053751 |
| MIR19A | MicroRNA 128-1 | RNA Gene | 0 | 27 | GC02P135665 |
| MIR150 | MicroRNA 25 | RNA Gene | 0 | 28 | GC07M100093 |
| MIR616 | MicroRNA 517c | RNA Gene | 0 | 20 | GC19P162646 |
| MIR141 | MicroRNA 34c | RNA Gene | 0 | 30 | GC11P116366 |
| MIR612 | MicroRNA 10a | RNA Gene | 0 | 30 | GC17M048579 |
| MIR124-1 | MicroRNA 24-2 | RNA Gene | 0 | 27 | GC19M112016 |
| MIR101-1 | MicroRNA 520g | RNA Gene | 0 | 23 | GC19P053722 |
| LINC01186 | MicroRNA 33a | RNA Gene | 0 | 27 | GC22P041900 |
| TUSC7 | MicroRNA 23b | RNA Gene | 0 | 28 | GC09P095085 |
| TDGF1 | MicroRNA 452 | RNA Gene | 0 | 24 | GC0XM151959 |
| MIR92B | MicroRNA 30d | RNA Gene | 0 | 26 | GC08M134804 |
| MIR646 | MicroRNA 217 | RNA Gene | 0 | 27 | GC02M055982 |
| MIR639 | MicroRNA 301a | RNA Gene | 0 | 27 | GC17M059151 |
| MIR638 | MicroRNA 216a | RNA Gene | 0 | 28 | GC02M055988 |
| MIR573 | MicroRNA 27a | RNA Gene | 0 | 31 | GC19M112017 |
| LINC00261 | MicroRNA 211 | RNA Gene | 0 | 30 | GC15M031065 |
| MIR1181 | MicroRNA 24-1 | RNA Gene | 0 | 27 | GC09P095086 |
| TAZ | MicroRNA 208a | RNA Gene | 0 | 28 | GC14M023388 |
| MIR124-2HG | MicroRNA 224 | RNA Gene | 0 | 25 | GC0XM151958 |
| MIR888 | MicroRNA 221 | RNA Gene | 0 | 28 | GC0XM045746 |
| MIR875 | MicroRNA 199a-2 | RNA Gene | 0 | 28 | GC01M172235 |

**Supplementary Table S3. The list of immune-related genes.**

| **Gene Symbol** | **Description** | **Category** | **Uniprot ID** | **Gifts** | **GC ID** |
| --- | --- | --- | --- | --- | --- |
| STAT5 | Signal Transducer And Activator Of Transcription 5B | Protein Coding | P51692 | 62 | GC17M042199 |
| RAG1 | Recombination Activating 1 | Protein Coding | P15918 | 56 | GC11P036554 |
| RAG2 | Recombination Activating 2 | Protein Coding | P55895 | 52 | GC11M036575 |
| CTLA4 | Cytotoxic T-Lymphocyte Associated Protein 4 | Protein Coding | P16410 | 61 | GC02P204352 |
| PLCG2 | Phospholipase C Gamma 2 | Protein Coding | P16885 | 64 | GC16P081779 |
| NFAT5 | Nuclear Factor Of Activated T Cells 5 | Protein Coding | O94916 | 53 | GC16P069565 |
| ACP5 | Acid Phosphatase 5, Tartrate Resistant | Protein Coding | P13686 | 58 | GC19M011574 |
| IL10 | Interleukin 10 | Protein Coding | P22301 | 60 | GC01M206767 |
| MYD88 | MYD88 Innate Immune Signal Transduction Adaptor | Protein Coding | Q99836 | 62 | GC03P038397 |
| IFNG | Interferon Gamma | Protein Coding | P01579 | 63 | GC12M068154 |
| IL2RA | Interleukin 2 Receptor Subunit Alpha | Protein Coding | P01589 | 63 | GC10M006010 |
| BCL10 | BCL10 Immune Signaling Adaptor | Protein Coding | O95999 | 58 | GC01M085265 |
| STAT1 | Signal Transducer And Activator Of Transcription 1 | Protein Coding | P42224 | 66 | GC02M190908 |
| FOXP3 | Forkhead Box P3 | Protein Coding | Q9BZS1 | 60 | GC0XM049250 |
| NOD2 | Nucleotide Binding Oligomerization Domain Containing 2 | Protein Coding | Q9HC29 | 59 | GC16P050693 |
| STAT3 | Signal Transducer And Activator Of Transcription 3 | Protein Coding | P40763 | 66 | GC17M042313 |
| NFKB1 | Nuclear Factor Kappa B Subunit 1 | Protein Coding | P19838 | 66 | GC04P102501 |
| PIK3CD | Phosphatidylinositol-4,5-Bisphosphate 3-Kinase Catalytic Subunit Delta | Protein Coding | O00329 | 66 | GC01P060209 |
| TNFRSF13B | TNF Receptor Superfamily Member 13B | Protein Coding | O14836 | 60 | GC17M016929 |
| JAK3 | Janus Kinase 3 | Protein Coding | P52333 | 65 | GC19M017824 |
| CD40LG | CD40 Ligand | Protein Coding | P29965 | 62 | GC0XP136649 |
| TNF | Tumor Necrosis Factor | Protein Coding | P01375 | 65 | GC06P156244 |
| CD40 | CD40 Molecule | Protein Coding | P25942 | 63 | GC20P046118 |
| FASLG | Fas Ligand | Protein Coding | P48023 | 61 | GC01P172659 |
| TLR4 | Toll Like Receptor 4 | Protein Coding | O00206 | 64 | GC09P117704 |
| JAK1 | Janus Kinase 1 | Protein Coding | P23458 | 66 | GC01M064833 |
| FAS | Fas Cell Surface Death Receptor | Protein Coding | P25445 | 64 | GC10P116129 |
| IL2RG | Interleukin 2 Receptor Subunit Gamma | Protein Coding | P31785 | 60 | GC0XM071111 |
| MEFV | MEFV Innate Immunity Regulator, Pyrin | Protein Coding | O15553 | 56 | GC16M027034 |
| IKBKB | Inhibitor Of Nuclear Factor Kappa B Kinase Subunit Beta | Protein Coding | O14920 | 66 | GC08P042271 |
| CD27 | CD27 Molecule | Protein Coding | P26842 | 59 | GC12P046735 |
| IFIH1 | Interferon Induced With Helicase C Domain 1 | Protein Coding | Q9BYX4 | 61 | GC02M162267 |
| ADA | Adenosine Deaminase | Protein Coding | P00813 | 63 | GC20M044620 |
| RIGI | RNA Sensor RIG-I | Protein Coding | O95786 | 61 | GC09M032545 |
| TLR2 | Toll Like Receptor 2 | Protein Coding | O60603 | 65 | GC04P153684 |
| NLRP3 | NLR Family Pyrin Domain Containing 3 | Protein Coding | Q96P20 | 61 | GC01P247539 |
| STING1 | Stimulator Of Interferon Response CGAMP Interactor 1 | Protein Coding | Q86WV6 | 54 | GC05M139497 |
| TGFB1 | Transforming Growth Factor Beta 1 | Protein Coding | P01137 | 65 | GC19M041301 |
| CD8A | CD8 Subunit Alpha | Protein Coding | P01732 | 61 | GC02M086784 |
| PRF1 | Perforin 1 | Protein Coding | P14222 | 59 | GC10M070597 |
| HLA-DRB1 | Major Histocompatibility Complex, Class II, DR Beta 1 | Protein Coding | P01911 | 59 | GC06M100352 |
| CD19 | CD19 Molecule | Protein Coding | P15391 | 63 | GC16P104821 |
| TYK2 | Tyrosine Kinase 2 | Protein Coding | P29597 | 66 | GC19M010350 |
| IL2 | Interleukin 2 | Protein Coding | P60568 | 58 | GC04M122451 |
| IFNGR1 | Interferon Gamma Receptor 1 | Protein Coding | P15260 | 63 | GC06M137197 |
| NFKB2 | Nuclear Factor Kappa B Subunit 2 | Protein Coding | Q00653 | 65 | GC10P102394 |
| ZAP70 | Zeta Chain Of T Cell Receptor Associated Protein Kinase 70 | Protein Coding | P43403 | 64 | GC02P098600 |
| IL7R | Interleukin 7 Receptor | Protein Coding | P16871 | 59 | GC05P035852 |
| CD79A | CD79a Molecule | Protein Coding | P11912 | 61 | GC19P041877 |
| C3 | Complement C3 | Protein Coding | P01024 | 62 | GC19M006677 |
| WAS | WASP Actin Nucleation Promoting Factor | Protein Coding | P42768 | 61 | GC0XP048676 |
| LINC02605 | Long Intergenic Non-Protein Coding RNA 2605 | RNA Gene |  | 22 | GC08P078838 |
| SYK | Spleen Associated Tyrosine Kinase | Protein Coding | P43405 | 63 | GC09P103553 |
| NFKBIA | NFKB Inhibitor Alpha | Protein Coding | P25963 | 63 | GC14M035401 |
| IL21 | Interleukin 21 | Protein Coding | Q9HBE4 | 56 | GC04M122612 |
| IL6 | Interleukin 6 | Protein Coding | P05231 | 63 | GC07P022725 |
| TNFRSF1A | TNF Receptor Superfamily Member 1A | Protein Coding | P19438 | 62 | GC12M006328 |
| BTK | Bruton Tyrosine Kinase | Protein Coding | Q06187 | 66 | GC0XM101349 |
| SH2D1A | SH2 Domain Containing 1A | Protein Coding | O60880 | 59 | GC0XP124227 |
| CARD11 | Caspase Recruitment Domain Family Member 11 | Protein Coding | Q9BXL7 | 59 | GC07M002906 |
| CD247 | CD247 Molecule | Protein Coding | P20963 | 62 | GC01M167399 |
| CD274 | CD274 Molecule | Protein Coding | Q9NZQ7 | 58 | GC09P005450 |
| IL1RN | Interleukin 1 Receptor Antagonist | Protein Coding | P18510 | 62 | GC02P148590 |
| PTPRC | Protein Tyrosine Phosphatase Receptor Type C | Protein Coding | P08575 | 65 | GC01P198607 |
| IL12RB1 | Interleukin 12 Receptor Subunit Beta 1 | Protein Coding | P42701 | 58 | GC19M018058 |
| ICOS | Inducible T Cell Costimulator | Protein Coding | Q9Y6W8 | 57 | GC02P204354 |
| IL12B | Interleukin 12B | Protein Coding | P29460 | 57 | GC05M159314 |
| IRAK4 | Interleukin 1 Receptor Associated Kinase 4 | Protein Coding | Q9NWZ3 | 62 | GC12P043758 |
| TCIRG1 | T Cell Immune Regulator 1, ATPase H+ Transporting V0 Subunit A3 | Protein Coding | Q13488 | 56 | GC11P099181 |
| IKBKG | Inhibitor Of Nuclear Factor Kappa B Kinase Regulatory Subunit Gamma | Protein Coding | Q9Y6K9 | 60 | GC0XP154541 |
| CR2 | Complement C3d Receptor 2 | Protein Coding | P20023 | 58 | GC01P207454 |
| DOCK8 | Dedicator Of Cytokinesis 8 | Protein Coding | Q8NF50 | 58 | GC09P000312 |
| RIPK1 | Receptor Interacting Serine/Threonine Kinase 1 | Protein Coding | Q13546 | 63 | GC06P008041 |
| CASP8 | Caspase 8 | Protein Coding | Q14790 | 65 | GC02P201233 |
| PIK3R1 | Phosphoinositide-3-Kinase Regulatory Subunit 1 | Protein Coding | P27986 | 65 | GC05P068215 |
| PNP | Purine Nucleoside Phosphorylase | Protein Coding | P00491 | 62 | GC14P051461 |
| ARPC1B | Actin Related Protein 2/3 Complex Subunit 1B | Protein Coding | O15143 | 55 | GC07P099374 |
| ITGB2 | Integrin Subunit Beta 2 | Protein Coding | P05107 | 63 | GC21M044885 |
| KRAS | KRAS Proto-Oncogene, GTPase | Protein Coding | P01116 | 66 | GC12M033285 |
| LRBA | LPS Responsive Beige-Like Anchor Protein | Protein Coding | P50851 | 52 | GC04M150264 |
| ATM | ATM Serine/Threonine Kinase | Protein Coding | Q13315 | 66 | GC11P108223 |
| XIAP | X-Linked Inhibitor Of Apoptosis | Protein Coding | P98170 | 64 | GC0XP123859 |
| PDCD1 | Programmed Cell Death 1 | Protein Coding | Q15116 | 59 | GC02M241849 |
| ITK | IL2 Inducible T Cell Kinase | Protein Coding | Q08881 | 63 | GC05P157158 |
| FCGR3A | Fc Gamma Receptor IIIa | Protein Coding | P08637 | 59 | GC01M161541 |
| IL10RA | Interleukin 10 Receptor Subunit Alpha | Protein Coding | Q13651 | 57 | GC11P118000 |
| AIRE | Autoimmune Regulator | Protein Coding | O43918 | 56 | GC21P044285 |
| CARD9 | Caspase Recruitment Domain Family Member 9 | Protein Coding | Q9H257 | 58 | GC09M136364 |
| TNFAIP3 | TNF Alpha Induced Protein 3 | Protein Coding | P21580 | 62 | GC06P137866 |
| ORAI1 | ORAI Calcium Release-Activated Calcium Modulator 1 | Protein Coding | Q96D31 | 57 | GC12P136103 |
| PRKCD | Protein Kinase C Delta | Protein Coding | Q05655 | 65 | GC03P053156 |
| CD4 | CD4 Molecule | Protein Coding | P01730 | 63 | GC12P006786 |
| SAMHD1 | SAM And HD Domain Containing Deoxynucleoside Triphosphate Triphosphohydrolase 1 | Protein Coding | Q9Y3Z3 | 55 | GC20M036890 |
| CD59 | CD59 Molecule (CD59 Blood Group) | Protein Coding | P13987 | 59 | GC11M033729 |
| LCK | LCK Proto-Oncogene, Src Family Tyrosine Kinase | Protein Coding | P06239 | 64 | GC01P032251 |
| SOCS1 | Suppressor Of Cytokine Signaling 1 | Protein Coding | O15524 | 58 | GC16M027316 |
| TYROBP | Transmembrane Immune Signaling Adaptor TYROBP | Protein Coding | O43914 | 55 | GC19M035904 |
| TLR3 | Toll Like Receptor 3 | Protein Coding | O15455 | 63 | GC04P186059 |
| NLRC4 | NLR Family CARD Domain Containing 4 | Protein Coding | Q9NPP4 | 55 | GC02M032224 |
| CD3G | CD3 Gamma Subunit Of T-Cell Receptor Complex | Protein Coding | P09693 | 59 | GC11P118344 |
| CD3D | CD3 Delta Subunit Of T-Cell Receptor Complex | Protein Coding | P04234 | 59 | GC11M118338 |
| CYBB | Cytochrome B-245 Beta Chain | Protein Coding | P04839 | 62 | GC0XP037780 |
| TERT | Telomerase Reverse Transcriptase | Protein Coding | O14746 | 64 | GC05M001253 |
| RAC2 | Rac Family Small GTPase 2 | Protein Coding | P15153 | 63 | GC22M081568 |
| PRKDC | Protein Kinase, DNA-Activated, Catalytic Subunit | Protein Coding | P78527 | 63 | GC08M047773 |
| CFH | Complement Factor H | Protein Coding | P08603 | 59 | GC01P196621 |
| DCLRE1C | DNA Cross-Link Repair 1C | Protein Coding | Q96SD1 | 56 | GC10M014897 |
| IRF8 | Interferon Regulatory Factor 8 | Protein Coding | Q02556 | 59 | GC16P106703 |
| FADD | Fas Associated Via Death Domain | Protein Coding | Q13158 | 59 | GC11P070203 |
| PTPN11 | Protein Tyrosine Phosphatase Non-Receptor Type 11 | Protein Coding | Q06124 | 66 | GC12P112418 |
| MALT1 | MALT1 Paracaspase | Protein Coding | Q9UDY8 | 60 | GC18P058671 |
| TREX1 | Three Prime Repair Exonuclease 1 | Protein Coding | Q9NSU2 | 55 | GC03P061485 |
| ISG15 | ISG15 Ubiquitin Like Modifier | Protein Coding | P05161 | 59 | GC01P001001 |
| IL10RB | Interleukin 10 Receptor Subunit Beta | Protein Coding | Q08334 | 56 | GC21P033266 |
| IL4 | Interleukin 4 | Protein Coding | P05112 | 59 | GC05P132673 |
| UNC13D | Unc-13 Homolog D | Protein Coding | Q70J99 | 56 | GC17M075827 |
| TP53 | Tumor Protein P53 | Protein Coding | P04637 | 66 | GC17M007661 |
| RNF31 | Ring Finger Protein 31 | Protein Coding | Q96EP0 | 53 | GC14P024146 |
| B2M | Beta-2-Microglobulin | Protein Coding | P61769 | 62 | GC15P044711 |
| CIITA | Class II Major Histocompatibility Complex Transactivator | Protein Coding | P33076 | 56 | GC16P104250 |
| CD81 | CD81 Molecule | Protein Coding | P60033 | 59 | GC11P014123 |
| NCF2 | Neutrophil Cytosolic Factor 2 | Protein Coding | P19878 | 60 | GC01M186360 |
| ELANE | Elastase, Neutrophil Expressed | Protein Coding | P08246 | 63 | GC19P131807 |
| RMRP | RNA Component Of Mitochondrial RNA Processing Endoribonuclease | RNA Gene |  | 33 | GC09M035655 |
| GATA2 | GATA Binding Protein 2 | Protein Coding | P23769 | 62 | GC03M128479 |
| STXBP2 | Syntaxin Binding Protein 2 | Protein Coding | Q15833 | 56 | GC19P132125 |
| LIG4 | DNA Ligase 4 | Protein Coding | P49917 | 59 | GC13M108207 |
| IL17RA | Interleukin 17 Receptor A | Protein Coding | Q96F46 | 59 | GC22P080745 |
| CXCR4 | C-X-C Motif Chemokine Receptor 4 | Protein Coding | P61073 | 65 | GC02M136114 |
| IFNGR2 | Interferon Gamma Receptor 2 | Protein Coding | P38484 | 55 | GC21P033402 |
| EGFR | Epidermal Growth Factor Receptor | Protein Coding | P00533 | 68 | GC07P055019 |
| CYBA | Cytochrome B-245 Alpha Chain | Protein Coding | P13498 | 58 | GC16M088643 |
| STAT2 | Signal Transducer And Activator Of Transcription 2 | Protein Coding | P52630 | 62 | GC12M056341 |
| MAGT1 | Magnesium Transporter 1 | Protein Coding | Q9H0U3 | 53 | GC0XM078277 |
| CD3E | CD3 Epsilon Subunit Of T-Cell Receptor Complex | Protein Coding | P07766 | 59 | GC11P118304 |
| HLA-B | Major Histocompatibility Complex, Class I, B | Protein Coding | P01889 | 59 | GC06M100255 |
| CDC42 | Cell Division Cycle 42 | Protein Coding | P60953 | 63 | GC01P022052 |
| IL17A | Interleukin 17A | Protein Coding | Q16552 | 55 | GC06P052186 |
| IL1B | Interleukin 1 Beta | Protein Coding | P01584 | 60 | GC02M112829 |
| TLR7 | Toll Like Receptor 7 | Protein Coding | Q9NYK1 | 61 | GC0XP012867 |
| IRF7 | Interferon Regulatory Factor 7 | Protein Coding | Q92985 | 60 | GC11M000612 |
| TNFRSF13C | TNF Receptor Superfamily Member 13C | Protein Coding | Q96RJ3 | 58 | GC22M081598 |
| STIM1 | Stromal Interaction Molecule 1 | Protein Coding | Q13586 | 62 | GC11P014188 |
| IL2RB | Interleukin 2 Receptor Subunit Beta | Protein Coding | P14784 | 63 | GC22M081565 |
| C1QA | Complement C1q A Chain | Protein Coding | P02745 | 60 | GC01P060983 |
| RAB27A | RAB27A, Member RAS Oncogene Family | Protein Coding | P51159 | 60 | GC15M055202 |
| CTPS1 | CTP Synthase 1 | Protein Coding | P17812 | 58 | GC01P040979 |
| CD70 | CD70 Molecule | Protein Coding | P32970 | 55 | GC19M100106 |
| HAVCR2 | Hepatitis A Virus Cellular Receptor 2 | Protein Coding | Q8TDQ0 | 58 | GC05M157063 |
| SFTPD | Surfactant Protein D | Protein Coding | P35247 | 56 | GC10M079937 |
| CFP | Complement Factor Properdin | Protein Coding | P27918 | 55 | GC0XM047623 |
| CLEC7A | C-Type Lectin Domain Containing 7A | Protein Coding | Q9BXN2 | 58 | GC12M033111 |
| HLA-DQB1 | Major Histocompatibility Complex, Class II, DQ Beta 1 | Protein Coding | P01920 | 55 | GC06M100361 |
| RBCK1 | RANBP2-Type And C3HC4-Type Zinc Finger Containing 1 | Protein Coding | Q9BYM8 | 54 | GC20P000407 |
| ITGAM | Integrin Subunit Alpha M | Protein Coding | P11215 | 61 | GC16P105014 |
| LAT | Linker For Activation Of T Cells | Protein Coding | O43561 | 59 | GC16P104827 |
| TBK1 | TANK Binding Kinase 1 | Protein Coding | Q9UHD2 | 62 | GC12P067025 |
| CFI | Complement Factor I | Protein Coding | P05156 | 59 | GC04M109732 |
| PSMB8 | Proteasome 20S Subunit Beta 8 | Protein Coding | P28062 | 62 | GC06M032840 |
| CD55 | CD55 Molecule (Cromer Blood Group) | Protein Coding | P08174 | 62 | GC01P207321 |
| CASP10 | Caspase 10 | Protein Coding | Q92851 | 60 | GC02P201182 |
| NRAS | NRAS Proto-Oncogene, GTPase | Protein Coding | P01111 | 63 | GC01M114704 |
| TLR9 | Toll Like Receptor 9 | Protein Coding | Q9NR96 | 58 | GC03M054385 |
| FCGR2B | Fc Gamma Receptor IIb | Protein Coding | P31994 | 61 | GC01P170820 |
| BRCA1 | BRCA1 DNA Repair Associated | Protein Coding | P38398 | 63 | GC17M043044 |
| PGM3 | Phosphoglucomutase 3 | Protein Coding | O95394 | 55 | GC06M101016 |
| IL21R | Interleukin 21 Receptor | Protein Coding | Q9HBE5 | 55 | GC16P104699 |
| IL17F | Interleukin 17F | Protein Coding | Q96PD4 | 53 | GC06M100650 |
| LYN | LYN Proto-Oncogene, Src Family Tyrosine Kinase | Protein Coding | P07948 | 62 | GC08P055879 |
| SMARCAL1 | SWI/SNF Related, Matrix Associated, Actin Dependent Regulator Of Chromatin, Subfamily A Like 1 | Protein Coding | Q9NZC9 | 58 | GC02P216412 |
| IKZF1 | IKAROS Family Zinc Finger 1 | Protein Coding | Q13422 | 61 | GC07P050303 |
| TAP1 | Transporter 1, ATP Binding Cassette Subfamily B Member | Protein Coding | Q03518 | 60 | GC06M100371 |
| C5 | Complement C5 | Protein Coding | P01031 | 59 | GC09M120933 |
| IGHM | Immunoglobulin Heavy Constant Mu | Protein Coding | P01871 | 45 | GC14M122267 |
| PSTPIP1 | Proline-Serine-Threonine Phosphatase Interacting Protein 1 | Protein Coding | O43586 | 55 | GC15P076993 |
| AICDA | Activation Induced Cytidine Deaminase | Protein Coding | Q9GZX7 | 56 | GC12M008602 |
| MSN | Moesin | Protein Coding | P26038 | 60 | GC0XP065588 |
| NCF4 | Neutrophil Cytosolic Factor 4 | Protein Coding | Q15080 | 60 | GC22P036860 |
| STX11 | Syntaxin 11 | Protein Coding | O75558 | 53 | GC06P163498 |
| ITCH | Itchy E3 Ubiquitin Protein Ligase | Protein Coding | Q96J02 | 59 | GC20P034363 |
| ADAM17 | ADAM Metallopeptidase Domain 17 | Protein Coding | P78536 | 63 | GC02M009488 |
| CORO1A | Coronin 1A | Protein Coding | P31146 | 55 | GC16P104933 |
| TTC7A | Tetratricopeptide Repeat Domain 7A | Protein Coding | Q9ULT0 | 51 | GC02P046923 |
| DNMT3B | DNA Methyltransferase 3 Beta | Protein Coding | Q9UBC3 | 64 | GC20P032762 |
| TFRC | Transferrin Receptor | Protein Coding | P02786 | 63 | GC03M196576 |
| IL13 | Interleukin 13 | Protein Coding | P35225 | 56 | GC05P132656 |
| CFB | Complement Factor B | Protein Coding | P00751 | 59 | GC06P031945 |
| RASGRP1 | RAS Guanyl Releasing Protein 1 | Protein Coding | O95267 | 58 | GC15M038488 |
| CD79B | CD79b Molecule | Protein Coding | P40259 | 58 | GC17M063928 |
| MPO | Myeloperoxidase | Protein Coding | P05164 | 65 | GC17M058269 |
| SPINK5 | Serine Peptidase Inhibitor Kazal Type 5 | Protein Coding | Q9NQ38 | 53 | GC05P148025 |
| RELB | RELB Proto-Oncogene, NF-KB Subunit | Protein Coding | Q01201 | 58 | GC19P133242 |
| NHEJ1 | Non-Homologous End Joining Factor 1 | Protein Coding | Q9H9Q4 | 54 | GC02M219180 |
| MBL2 | Mannose Binding Lectin 2 | Protein Coding | P11226 | 59 | GC10M052760 |
| IL36RN | Interleukin 36 Receptor Antagonist | Protein Coding | Q9UBH0 | 54 | GC02P148589 |
| TAP2 | Transporter 2, ATP Binding Cassette Subfamily B Member | Protein Coding | Q03519 | 56 | GC06M032821 |
| BLNK | B Cell Linker | Protein Coding | Q8WV28 | 57 | GC10M096313 |
| IRF3 | Interferon Regulatory Factor 3 | Protein Coding | Q14653 | 58 | GC19M049659 |
| MVK | Mevalonate Kinase | Protein Coding | Q03426 | 60 | GC12P109573 |
| CXCL8 | C-X-C Motif Chemokine Ligand 8 | Protein Coding | P10145 | 56 | GC04P073740 |
| DKC1 | Dyskerin Pseudouridine Synthase 1 | Protein Coding | O60832 | 59 | GC0XP154762 |
| IRGM | Immunity Related GTPase M | Protein Coding | A1A4Y4 | 45 | GC05P150846 |
| NBN | Nibrin | Protein Coding | O60934 | 61 | GC08M089933 |
| HLA-A | Major Histocompatibility Complex, Class I, A | Protein Coding | P04439 | 58 | GC06P156203 |
| RORC | RAR Related Orphan Receptor C | Protein Coding | P51449 | 55 | GC01M151806 |
| C1QB | Complement C1q B Chain | Protein Coding | P02746 | 56 | GC01P022652 |
| C1QC | Complement C1q C Chain | Protein Coding | P02747 | 58 | GC01P022643 |
| POLE | DNA Polymerase Epsilon, Catalytic Subunit | Protein Coding | Q07864 | 60 | GC12M132798 |
| LEP | Leptin | Protein Coding | P41159 | 59 | GC07P128241 |
| PLCG1 | Phospholipase C Gamma 1 | Protein Coding | P19174 | 62 | GC20P041136 |
| BLM | BLM RecQ Like Helicase | Protein Coding | P54132 | 62 | GC15P090717 |
| FOXN1 | Forkhead Box N1 | Protein Coding | O15353 | 52 | GC17P028506 |
| ADA2 | Adenosine Deaminase 2 | Protein Coding | Q9NZK5 | 54 | GC22M017342 |
| IL17RC | Interleukin 17 Receptor C | Protein Coding | Q8NAC3 | 52 | GC03P009917 |
| TNFRSF4 | TNF Receptor Superfamily Member 4 | Protein Coding | P43489 | 56 | GC01M001211 |
| RTEL1 | Regulator Of Telomere Elongation Helicase 1 | Protein Coding | Q9NZ71 | 54 | GC20P068110 |
| HLA-G | Major Histocompatibility Complex, Class I, G | Protein Coding | P17693 | 57 | GC06P156194 |
| NLRP12 | NLR Family Pyrin Domain Containing 12 | Protein Coding | P59046 | 55 | GC19M053793 |
| C1S | Complement C1s | Protein Coding | P09871 | 60 | GC12P046796 |
| FCGR2A | Fc Gamma Receptor IIa | Protein Coding | P12318 | 60 | GC01P161505 |
| BRCA2 | BRCA2 DNA Repair Associated | Protein Coding | P51587 | 60 | GC13P032315 |
| LYST | Lysosomal Trafficking Regulator | Protein Coding | Q99698 | 50 | GC01M235661 |
| ADAR | Adenosine Deaminase RNA Specific | Protein Coding | P55265 | 59 | GC01M165080 |
| WIPF1 | WAS/WASL Interacting Protein Family Member 1 | Protein Coding | O43516 | 54 | GC02M174559 |
| RFX5 | Regulatory Factor X5 | Protein Coding | P48382 | 53 | GC01M151340 |
| CHD7 | Chromodomain Helicase DNA Binding Protein 7 | Protein Coding | Q9P2D1 | 56 | GC08P060678 |
| IFNA1 | Interferon Alpha 1 | Protein Coding | P01562 | 51 | GC09P021872 |
| AK2 | Adenylate Kinase 2 | Protein Coding | P54819 | 59 | GC01M033007 |
| IRF1 | Interferon Regulatory Factor 1 | Protein Coding | P10914 | 60 | GC05M132440 |
| RFXANK | Regulatory Factor X Associated Ankyrin Containing Protein | Protein Coding | O14593 | 50 | GC19P019192 |
| AP3B1 | Adaptor Related Protein Complex 3 Subunit Beta 1 | Protein Coding | O00203 | 57 | GC05M078000 |
| NLRP1 | NLR Family Pyrin Domain Containing 1 | Protein Coding | Q9C000 | 55 | GC17M005499 |
| C2 | Complement C2 | Protein Coding | P06681 | 59 | GC06P031897 |
| CD28 | CD28 Molecule | Protein Coding | P10747 | 59 | GC02P203706 |
| BACH2 | BTB Domain And CNC Homolog 2 | Protein Coding | Q9BYV9 | 54 | GC06M089926 |
| DOCK2 | Dedicator Of Cytokinesis 2 | Protein Coding | Q92608 | 56 | GC05P169637 |
| CREBBP | CREB Binding Protein | Protein Coding | Q92793 | 67 | GC16M027070 |
| EPG5 | Ectopic P-Granules 5 Autophagy Tethering Factor | Protein Coding | Q9HCE0 | 51 | GC18M045800 |
| PTEN | Phosphatase And Tensin Homolog | Protein Coding | P60484 | 65 | GC10P116113 |
| HRAS | HRas Proto-Oncogene, GTPase | Protein Coding | P01112 | 65 | GC11M013218 |
| TRAF3IP2 | TRAF3 Interacting Protein 2 | Protein Coding | O43734 | 55 | GC06M111555 |
| TNFSF12 | TNF Superfamily Member 12 | Protein Coding | O43508 | 51 | GC17P132657 |
| HMGB1 | High Mobility Group Box 1 | Protein Coding | P09429 | 62 | GC13M030456 |
| CD86 | CD86 Molecule | Protein Coding | P42081 | 56 | GC03P122055 |
| TRAF3 | TNF Receptor Associated Factor 3 | Protein Coding | Q13114 | 59 | GC14P118105 |
| SERPING1 | Serpin Family G Member 1 | Protein Coding | P05155 | 59 | GC11P057597 |
| IGKC | Immunoglobulin Kappa Constant | Protein Coding | P01834 | 41 | GC02M092645 |
| TLR8 | Toll Like Receptor 8 | Protein Coding | Q9NR97 | 62 | GC0XP012924 |
| CGAS | Cyclic GMP-AMP Synthase | Protein Coding | Q8N884 | 48 | GC06M100849 |
| CD46 | CD46 Molecule | Protein Coding | P15529 | 60 | GC01P207752 |
| RFXAP | Regulatory Factor X Associated Protein | Protein Coding | O00287 | 46 | GC13P036819 |
| OAS1 | 2'-5'-Oligoadenylate Synthetase 1 | Protein Coding | P00973 | 56 | GC12P113132 |
| TLR1 | Toll Like Receptor 1 | Protein Coding | Q15399 | 60 | GC04M038793 |
| MAVS | Mitochondrial Antiviral Signaling Protein | Protein Coding | Q7Z434 | 52 | GC20P009569 |
| PTPN22 | Protein Tyrosine Phosphatase Non-Receptor Type 22 | Protein Coding | Q9Y2R2 | 59 | GC01M113813 |
| BCL11B | BCL11 Transcription Factor B | Protein Coding | Q9C0K0 | 55 | GC14M099169 |
| MS4A1 | Membrane Spanning 4-Domains A1 | Protein Coding | P11836 | 58 | GC11P098544 |
| AKT1 | AKT Serine/Threonine Kinase 1 | Protein Coding | P31749 | 66 | GC14M104769 |
| HLA-DPB1 | Major Histocompatibility Complex, Class II, DP Beta 1 | Protein Coding | P04440 | 56 | GC06P156281 |
| CSF3R | Colony Stimulating Factor 3 Receptor | Protein Coding | Q99062 | 59 | GC01M036466 |
| HLA-DQA1 | Major Histocompatibility Complex, Class II, DQ Alpha 1 | Protein Coding | P01909 | 55 | GC06P156274 |
| IFNB1 | Interferon Beta 1 | Protein Coding | P01574 | 55 | GC09M021077 |
| UNC93B1 | Unc-93 Homolog B1, TLR Signaling Regulator | Protein Coding | Q9H1C4 | 50 | GC11M067991 |
| IL1A | Interleukin 1 Alpha | Protein Coding | P01583 | 58 | GC02M112773 |
| CFD | Complement Factor D | Protein Coding | P00746 | 56 | GC19P000859 |
| TBX1 | T-Box Transcription Factor 1 | Protein Coding | O43435 | 55 | GC22P080835 |
| PIK3CA | Phosphatidylinositol-4,5-Bisphosphate 3-Kinase Catalytic Subunit Alpha | Protein Coding | P42336 | 65 | GC03P179148 |
| IL12A | Interleukin 12A | Protein Coding | P29459 | 56 | GC03P159988 |
| SBDS | SBDS Ribosome Maturation Factor | Protein Coding | Q9Y3A5 | 55 | GC07M066987 |
| UNG | Uracil DNA Glycosylase | Protein Coding | P13051 | 59 | GC12P109097 |
| IL7 | Interleukin 7 | Protein Coding | P13232 | 55 | GC08M078689 |
| BRAF | B-Raf Proto-Oncogene, Serine/Threonine Kinase | Protein Coding | P15056 | 66 | GC07M140783 |
| C7 | Complement C7 | Protein Coding | P10643 | 55 | GC05P040909 |
| IRF2BP2 | Interferon Regulatory Factor 2 Binding Protein 2 | Protein Coding | Q7Z5L9 | 48 | GC01M234604 |
| TAPBP | TAP Binding Protein | Protein Coding | O15533 | 54 | GC06M033299 |
| STK4 | Serine/Threonine Kinase 4 | Protein Coding | Q13043 | 61 | GC20P044966 |
| EXTL3 | Exostosin Like Glycosyltransferase 3 | Protein Coding | O43909 | 55 | GC08P031690 |
| HELLS | Helicase, Lymphoid Specific | Protein Coding | Q9NRZ9 | 58 | GC10P116226 |
| CD80 | CD80 Molecule | Protein Coding | P33681 | 55 | GC03M119524 |
| HLA-C | Major Histocompatibility Complex, Class I, C | Protein Coding | P10321 | 56 | GC06M100254 |
| SLC37A4 | Solute Carrier Family 37 Member 4 | Protein Coding | O43826 | 54 | GC11M133861 |
| NFE2L2 | NFE2 Like BZIP Transcription Factor 2 | Protein Coding | Q16236 | 63 | GC02M177227 |
| RNF168 | Ring Finger Protein 168 | Protein Coding | Q8IYW5 | 55 | GC03M196468 |
| IL18 | Interleukin 18 | Protein Coding | Q14116 | 56 | GC11M112143 |
| SAMD9L | Sterile Alpha Motif Domain Containing 9 Like | Protein Coding | Q8IVG5 | 49 | GC07M093130 |
| PIK3CG | Phosphatidylinositol-4,5-Bisphosphate 3-Kinase Catalytic Subunit Gamma | Protein Coding | P48736 | 62 | GC07P106865 |
| IL5 | Interleukin 5 | Protein Coding | P05113 | 58 | GC05M132541 |
| FERMT3 | FERM Domain Containing Kindlin 3 | Protein Coding | Q86UX7 | 55 | GC11P098843 |
| IFNAR2 | Interferon Alpha And Beta Receptor Subunit 2 | Protein Coding | P48551 | 61 | GC21P033229 |
| MYC | MYC Proto-Oncogene, BHLH Transcription Factor | Protein Coding | P01106 | 65 | GC08P127735 |
| PVR | PVR Cell Adhesion Molecule | Protein Coding | P15151 | 58 | GC19P133226 |
| MTHFD1 | Methylenetetrahydrofolate Dehydrogenase, Cyclohydrolase And Formyltetrahydrofolate Synthetase 1 | Protein Coding | P11586 | 58 | GC14P064388 |
| FCGR3B | Fc Gamma Receptor IIIb | Protein Coding | O75015 | 55 | GC01M161623 |
| TRAF6 | TNF Receptor Associated Factor 6 | Protein Coding | Q9Y4K3 | 59 | GC11M036467 |
| HAX1 | HCLS1 Associated Protein X-1 | Protein Coding | O00165 | 55 | GC01P170399 |
| CCR5 | C-C Motif Chemokine Receptor 5 | Protein Coding | P51681 | 59 | GC03P061355 |
| MIR155 | MicroRNA 155 | RNA Gene |  | 30 | GC21P025573 |
| TINF2 | TERF1 Interacting Nuclear Factor 2 | Protein Coding | Q9BSI4 | 54 | GC14M027974 |
| RELA | RELA Proto-Oncogene, NF-KB Subunit | Protein Coding | Q04206 | 64 | GC11M065653 |
| IL15 | Interleukin 15 | Protein Coding | P40933 | 55 | GC04P141636 |
| ZBTB24 | Zinc Finger And BTB Domain Containing 24 | Protein Coding | O43167 | 50 | GC06M109476 |
| RC3H1 | Ring Finger And CCCH-Type Domains 1 | Protein Coding | Q5TC82 | 45 | GC01M173931 |
| TRNT1 | TRNA Nucleotidyl Transferase 1 | Protein Coding | Q96Q11 | 54 | GC03P003126 |
| GFI1 | Growth Factor Independent 1 Transcriptional Repressor | Protein Coding | Q99684 | 53 | GC01M092474 |
| ICAM1 | Intercellular Adhesion Molecule 1 | Protein Coding | P05362 | 63 | GC19P132284 |
| IRF5 | Interferon Regulatory Factor 5 | Protein Coding | Q13568 | 59 | GC07P128937 |
| PEPD | Peptidase D | Protein Coding | P12955 | 58 | GC19M033386 |
| C8A | Complement C8 Alpha Chain | Protein Coding | P07357 | 54 | GC01P056854 |
| MAP3K14 | Mitogen-Activated Protein Kinase Kinase Kinase 14 | Protein Coding | Q99558 | 56 | GC17M045263 |
| IGLL1 | Immunoglobulin Lambda Like Polypeptide 1 | Protein Coding | P15814 | 54 | GC22M023573 |
| TRAC | T Cell Receptor Alpha Constant | Protein Coding | P01848 | 38 | GC14P051665 |
| CD14 | CD14 Molecule | Protein Coding | P08571 | 59 | GC05M140631 |
| CCL2 | C-C Motif Chemokine Ligand 2 | Protein Coding | P13500 | 61 | GC17P034255 |
| MASP1 | MBL Associated Serine Protease 1 | Protein Coding | P48740 | 60 | GC03M187216 |
| FCN3 | Ficolin 3 | Protein Coding | O75636 | 55 | GC01M032296 |
| C8B | Complement C8 Beta Chain | Protein Coding | P07358 | 55 | GC01M056929 |
| CXCL10 | C-X-C Motif Chemokine Ligand 10 | Protein Coding | P02778 | 56 | GC04M076021 |
| SLC35C1 | Solute Carrier Family 35 Member C1 | Protein Coding | Q96A29 | 51 | GC11P048336 |
| CEBPE | CCAAT Enhancer Binding Protein Epsilon | Protein Coding | Q15744 | 54 | GC14M023117 |
| JAK2 | Janus Kinase 2 | Protein Coding | O60674 | 66 | GC09P004985 |
| FANCC | FA Complementation Group C | Protein Coding | Q00597 | 60 | GC09M095099 |
| FANCM | FA Complementation Group M | Protein Coding | Q8IYD8 | 54 | GC14P045135 |
| C4A | Complement C4A (Chido/Rodgers Blood Group) | Protein Coding | P0C0L4 | 56 | GC06P156266 |
| NCF1 | Neutrophil Cytosolic Factor 1 | Protein Coding | P14598 | 59 | GC07P090617 |
| NGF | Nerve Growth Factor | Protein Coding | P01138 | 63 | GC01M115285 |
| ETV6 | ETS Variant Transcription Factor 6 | Protein Coding | P41212 | 59 | GC12P011649 |
| KMT2D | Lysine Methyltransferase 2D | Protein Coding | O14686 | 56 | GC12M049018 |
| MPL | MPL Proto-Oncogene, Thrombopoietin Receptor | Protein Coding | P40238 | 60 | GC01P043337 |
| WRAP53 | WD Repeat Containing Antisense To TP53 | Protein Coding | Q9BUR4 | 52 | GC17P132666 |
| ACTB | Actin Beta | Protein Coding | P60709 | 63 | GC07M005726 |
| SP110 | SP110 Nuclear Body Protein | Protein Coding | Q9HB58 | 50 | GC02M230167 |
| CFTR | CF Transmembrane Conductance Regulator | Protein Coding | P13569 | 66 | GC07P117287 |
| CTC1 | CST Telomere Replication Complex Component 1 | Protein Coding | Q2NKJ3 | 48 | GC17M088343 |
| MIF | Macrophage Migration Inhibitory Factor | Protein Coding | P14174 | 61 | GC22P023894 |
| OTULIN | OTU Deubiquitinase With Linear Linkage Specificity | Protein Coding | Q96BN8 | 51 | GC05P015639 |
| BRIP1 | BRCA1 Interacting Helicase 1 | Protein Coding | Q9BX63 | 62 | GC17M061679 |
| MR1 | Major Histocompatibility Complex, Class I-Related | Protein Coding | Q95460 | 52 | GC01P181033 |
| MCM4 | Minichromosome Maintenance Complex Component 4 | Protein Coding | P33991 | 60 | GC08P047975 |
| IGHE | Immunoglobulin Heavy Constant Epsilon | Protein Coding | P01854 | 42 | GC14M122252 |
| SAMD9 | Sterile Alpha Motif Domain Containing 9 | Protein Coding | Q5K651 | 48 | GC07M093099 |
| MYO5A | Myosin VA | Protein Coding | Q9Y4I1 | 56 | GC15M156997 |
| CRP | C-Reactive Protein | Protein Coding | P02741 | 58 | GC01M165279 |
| KLRK1 | Killer Cell Lectin Like Receptor K1 | Protein Coding | P26718 | 52 | GC12M033113 |
| MALAT1 | Metastasis Associated Lung Adenocarcinoma Transcript 1 | RNA Gene |  | 33 | GC11P098972 |
| DOCK11 | Dedicator Of Cytokinesis 11 | Protein Coding | Q5JSL3 | 49 | GC0XP118576 |
| LAMTOR2 | Late Endosomal/Lysosomal Adaptor, MAPK And MTOR Activator 2 | Protein Coding | Q9Y2Q5 | 52 | GC01P156054 |
| TLR5 | Toll Like Receptor 5 | Protein Coding | O60602 | 59 | GC01M223109 |
| TLR6 | Toll Like Receptor 6 | Protein Coding | Q9Y2C9 | 57 | GC04M038828 |
| NOP10 | NOP10 Ribonucleoprotein | Protein Coding | Q9NPE3 | 53 | GC15M041829 |
| STAT4 | Signal Transducer And Activator Of Transcription 4 | Protein Coding | Q14765 | 58 | GC02M191029 |
| LPIN2 | Lipin 2 | Protein Coding | Q92539 | 52 | GC18M003881 |
| C1R | Complement C1r | Protein Coding | P00736 | 59 | GC12M032906 |
| C6 | Complement C6 | Protein Coding | P13671 | 53 | GC05M041142 |
| MRE11 | MRE11 Homolog, Double Strand Break Repair Nuclease | Protein Coding | P49959 | 61 | GC11M133418 |
| FANCA | FA Complementation Group A | Protein Coding | O15360 | 62 | GC16M090235 |
| FCGR2C | Fc Gamma Receptor IIc (Gene/Pseudogene) | Protein Coding | P31995 | 45 | GC01P170815 |
| IRF4 | Interferon Regulatory Factor 4 | Protein Coding | Q15306 | 57 | GC06P000391 |
| UNC119 | Unc-119 Lipid Binding Chaperone | Protein Coding | Q13432 | 54 | GC17M028546 |
| SKIC3 | SKI3 Subunit Of Superkiller Complex | Protein Coding | Q6PGP7 | 51 | GC05M095461 |
| TICAM1 | TIR Domain Containing Adaptor Molecule 1 | Protein Coding | Q8IUC6 | 55 | GC19M004815 |
| SRP72 | Signal Recognition Particle 72 | Protein Coding | O76094 | 52 | GC04P056470 |
| IDO1 | Indoleamine 2,3-Dioxygenase 1 | Protein Coding | P14902 | 59 | GC08P040947 |
| JAGN1 | Jagunal Homolog 1 | Protein Coding | Q8N5M9 | 45 | GC03P009890 |
| CCL5 | C-C Motif Chemokine Ligand 5 | Protein Coding | P13501 | 56 | GC17M035871 |
| MYH7 | Myosin Heavy Chain 7 | Protein Coding | P12883 | 59 | GC14M023412 |
| MOGS | Mannosyl-Oligosaccharide Glucosidase | Protein Coding | Q13724 | 56 | GC02M074461 |
| RPSA | Ribosomal Protein SA | Protein Coding | P08865 | 59 | GC03P039406 |
| CHUK | Component Of Inhibitor Of Nuclear Factor Kappa B Kinase Complex | Protein Coding | O15111 | 63 | GC10M100501 |
| HFE | Homeostatic Iron Regulator | Protein Coding | Q30201 | 58 | GC06P026087 |
| NSMCE3 | NSE3 Homolog, SMC5-SMC6 Complex Component | Protein Coding | Q96MG7 | 50 | GC15M041480 |
| ERCC2 | ERCC Excision Repair 2, TFIIH Core Complex Helicase Subunit | Protein Coding | P18074 | 61 | GC19M045349 |
| G6PD | Glucose-6-Phosphate Dehydrogenase | Protein Coding | P11413 | 63 | GC0XM154610 |
| NHP2 | NHP2 Ribonucleoprotein | Protein Coding | Q9NX24 | 55 | GC05M178149 |
| CLPB | ClpB Family Mitochondrial Disaggregase | Protein Coding | Q9H078 | 56 | GC11M133042 |
| VPS45 | Vacuolar Protein Sorting 45 Homolog | Protein Coding | Q9NRW7 | 51 | GC01P170179 |
| GUSB | Glucuronidase Beta | Protein Coding | P08236 | 62 | GC07M065960 |
| REL | REL Proto-Oncogene, NF-KB Subunit | Protein Coding | Q04864 | 60 | GC02P060881 |
| CSF2 | Colony Stimulating Factor 2 | Protein Coding | P04141 | 58 | GC05P132073 |
| IL23R | Interleukin 23 Receptor | Protein Coding | Q5VWK5 | 56 | GC01P067138 |
| ACD | ACD Shelterin Complex Subunit And Telomerase Recruitment Factor | Protein Coding | Q96AP0 | 54 | GC16M067664 |
| G6PC3 | Glucose-6-Phosphatase Catalytic Subunit 3 | Protein Coding | Q9BUM1 | 53 | GC17P044070 |
| DEF6 | DEF6 Guanine Nucleotide Exchange Factor | Protein Coding | Q9H4E7 | 52 | GC06P156420 |
| HSPG2 | Heparan Sulfate Proteoglycan 2 | Protein Coding | P98160 | 60 | GC01M021822 |
| CDCA7 | Cell Division Cycle Associated 7 | Protein Coding | Q9BWT1 | 52 | GC02P173354 |
| IRAK1 | Interleukin 1 Receptor Associated Kinase 1 | Protein Coding | P51617 | 61 | GC0XM154010 |
| RNASEH2A | Ribonuclease H2 Subunit A | Protein Coding | O75792 | 56 | GC19P132436 |
| HLA-DRA | Major Histocompatibility Complex, Class II, DR Alpha | Protein Coding | P01903 | 58 | GC06P032439 |
| CARD14 | Caspase Recruitment Domain Family Member 14 | Protein Coding | Q9BXL6 | 54 | GC17P134788 |
| RAD51 | RAD51 Recombinase | Protein Coding | Q06609 | 64 | GC15P040694 |
| SLC46A1 | Solute Carrier Family 46 Member 1 | Protein Coding | Q96NT5 | 55 | GC17M088840 |
| CTSC | Cathepsin C | Protein Coding | P53634 | 60 | GC11M133353 |
| PMS2 | PMS1 Homolog 2, Mismatch Repair System Component | Protein Coding | P54278 | 62 | GC07M005973 |
| C4B | Complement C4B (Chido/Rodgers Blood Group) | Protein Coding | P0C0L5 | 55 | GC06P032014 |
| BCL2 | BCL2 Apoptosis Regulator | Protein Coding | P10415 | 63 | GC18M063123 |
| EIF2AK3 | Eukaryotic Translation Initiation Factor 2 Alpha Kinase 3 | Protein Coding | Q9NZJ5 | 63 | GC02M088556 |
| CARMIL2 | Capping Protein Regulator And Myosin 1 Linker 2 | Protein Coding | Q6F5E8 | 47 | GC16P067644 |
| FANCL | FA Complementation Group L | Protein Coding | Q9NW38 | 58 | GC02M058127 |
| CCR1 | C-C Motif Chemokine Receptor 1 | Protein Coding | P32246 | 57 | GC03M046218 |
| MIR146A | MicroRNA 146a | RNA Gene |  | 31 | GC05P160485 |
| RAD50 | RAD50 Double Strand Break Repair Protein | Protein Coding | Q92878 | 62 | GC05P132556 |
| TCIM | Transcriptional And Immune Response Regulator | Protein Coding | Q9NR00 | 43 | GC08P040153 |
| PARN | Poly(A)-Specific Ribonuclease | Protein Coding | O95453 | 56 | GC16M014435 |
| CBL | Cbl Proto-Oncogene | Protein Coding | P22681 | 64 | GC11P119206 |
| USB1 | U6 SnRNA Biogenesis Phosphodiesterase 1 | Protein Coding | Q9BQ65 | 46 | GC16P057999 |
| TGFB3 | Transforming Growth Factor Beta 3 | Protein Coding | P10600 | 61 | GC14M075958 |
| MIR146B | MicroRNA 146b | RNA Gene |  | 30 | GC10P102436 |
| TERC | Telomerase RNA Component | RNA Gene |  | 37 | GC03M169765 |
| VPS13B | Vacuolar Protein Sorting 13 Homolog B | Protein Coding | Q7Z7G8 | 52 | GC08P099128 |
| RNASEH2B | Ribonuclease H2 Subunit B | Protein Coding | Q5TBB1 | 48 | GC13P050909 |
| NEAT1 | Nuclear Paraspeckle Assembly Transcript 1 | RNA Gene |  | 33 | GC11P098966 |
| ERCC6L2 | ERCC Excision Repair 6 Like 2 | Protein Coding | Q5T890 | 50 | GC09P095871 |
| RAD51C | RAD51 Paralog C | Protein Coding | O43502 | 55 | GC17P058692 |
| USP18 | Ubiquitin Specific Peptidase 18 | Protein Coding | Q9UMW8 | 52 | GC22P018149 |
| NEU1 | Neuraminidase 1 | Protein Coding | Q99519 | 59 | GC06M031857 |
| SMARCA4 | SWI/SNF Related, Matrix Associated, Actin Dependent Regulator Of Chromatin, Subfamily A, Member 4 | Protein Coding | P51532 | 64 | GC19P132300 |
| TCF3 | Transcription Factor 3 | Protein Coding | P15923 | 59 | GC19M001609 |
| BUB1B | BUB1 Mitotic Checkpoint Serine/Threonine Kinase B | Protein Coding | O60566 | 63 | GC15P040161 |
| SKIC2 | SKI2 Subunit Of Superkiller Complex | Protein Coding | Q15477 | 54 | GC06P172784 |
| CDH1 | Cadherin 1 | Protein Coding | P12830 | 62 | GC16P068737 |
| FANCD2 | FA Complementation Group D2 | Protein Coding | Q9BXW9 | 59 | GC03P010026 |
| MIR21 | MicroRNA 21 | RNA Gene |  | 33 | GC17P134223 |
| TCN2 | Transcobalamin 2 | Protein Coding | P20062 | 55 | GC22P081250 |
| CTNNB1 | Catenin Beta 1 | Protein Coding | P35222 | 66 | GC03P041194 |
| PALB2 | Partner And Localizer Of BRCA2 | Protein Coding | Q86YC2 | 56 | GC16M023603 |
| COL2A1 | Collagen Type II Alpha 1 Chain | Protein Coding | P02458 | 62 | GC12M047972 |
| CDKN2A | Cyclin Dependent Kinase Inhibitor 2A | Protein Coding | Q8N726 | 63 | GC09M021967 |
| DSG1 | Desmoglein 1 | Protein Coding | Q02413 | 55 | GC18P031318 |
| SMAD5-AS1 | SMAD5 Antisense RNA 1 | RNA Gene | Q9Y6J3 | 30 | GC05M136129 |
| DNAJC21 | DnaJ Heat Shock Protein Family (Hsp40) Member C21 | Protein Coding | Q5F1R6 | 45 | GC05P034929 |
| MIR125A | MicroRNA 125a | RNA Gene |  | 30 | GC19P133539 |
| GATA3 | GATA Binding Protein 3 | Protein Coding | P23771 | 63 | GC10P008045 |
| TNFSF13B | TNF Superfamily Member 13b | Protein Coding | Q9Y275 | 58 | GC13P108251 |
| ERCC4 | ERCC Excision Repair 4, Endonuclease Catalytic Subunit | Protein Coding | Q92889 | 59 | GC16P013920 |
| MAP2K1 | Mitogen-Activated Protein Kinase Kinase 1 | Protein Coding | Q02750 | 67 | GC15P066386 |
| TNFRSF14 | TNF Receptor Superfamily Member 14 | Protein Coding | Q92956 | 56 | GC01P059925 |
| MAPK1 | Mitogen-Activated Protein Kinase 1 | Protein Coding | P28482 | 65 | GC22M021759 |
| CDK4 | Cyclin Dependent Kinase 4 | Protein Coding | P11802 | 66 | GC12M059932 |
| PIEZO1 | Piezo Type Mechanosensitive Ion Channel Component 1 (Er Blood Group) | Protein Coding | Q92508 | 52 | GC16M088715 |
| IL23A | Interleukin 23 Subunit Alpha | Protein Coding | Q9NPF7 | 51 | GC12P065392 |
| NOD1 | Nucleotide Binding Oligomerization Domain Containing 1 | Protein Coding | Q9Y239 | 55 | GC07M030424 |
| FANCE | FA Complementation Group E | Protein Coding | Q9HB96 | 54 | GC06P156426 |
| TET2 | Tet Methylcytosine Dioxygenase 2 | Protein Coding | Q6N021 | 58 | GC04P105145 |
| RNU4ATAC | RNA, U4atac Small Nuclear | RNA Gene |  | 28 | GC02P148731 |
| RUNX1 | RUNX Family Transcription Factor 1 | Protein Coding | Q01196 | 62 | GC21M034787 |
| TBCE | Tubulin Folding Cofactor E | Protein Coding | Q15813 | 52 | GC01P235367 |
| SLX4 | SLX4 Structure-Specific Endonuclease Subunit | Protein Coding | Q8IY92 | 50 | GC16M003581 |
| CAMP | Cathelicidin Antimicrobial Peptide | Protein Coding | P49913 | 53 | GC03P061472 |
| IL22 | Interleukin 22 | Protein Coding | Q9GZX6 | 54 | GC12M068248 |
| CERNA3 | Competing Endogenous LncRNA 3 For MiR-645 | RNA Gene |  | 21 | GC08P056381 |
| CCND1 | Cyclin D1 | Protein Coding | P24385 | 66 | GC11P069641 |
| TNFRSF6B | TNF Receptor Superfamily Member 6b | Protein Coding | O95407 | 54 | GC20P063696 |
| CD209 | CD209 Molecule | Protein Coding | Q9NNX6 | 56 | GC19M007739 |
| EIF2AK2 | Eukaryotic Translation Initiation Factor 2 Alpha Kinase 2 | Protein Coding | P19525 | 62 | GC02M037099 |
| PRTN3 | Proteinase 3 | Protein Coding | P24158 | 59 | GC19P000840 |
| LRRC8A | Leucine Rich Repeat Containing 8 VRAC Subunit A | Protein Coding | Q8IWT6 | 51 | GC09P128882 |
| MAN2B1 | Mannosidase Alpha Class 2B Member 1 | Protein Coding | O00754 | 56 | GC19M100368 |
| FANCG | FA Complementation Group G | Protein Coding | O15287 | 56 | GC09M035073 |
| FANCF | FA Complementation Group F | Protein Coding | Q9NPI8 | 53 | GC11M022600 |
| FANCB | FA Complementation Group B | Protein Coding | Q8NB91 | 52 | GC0XM015698 |
| KIT | KIT Proto-Oncogene, Receptor Tyrosine Kinase | Protein Coding | P10721 | 66 | GC04P054657 |
| ICOSLG | Inducible T Cell Costimulator Ligand | Protein Coding | O75144 | 54 | GC21M054233 |
| ELP1 | Elongator Acetyltransferase Complex Subunit 1 | Protein Coding | O95163 | 56 | GC09M118460 |
| HLA-DPA1 | Major Histocompatibility Complex, Class II, DP Alpha 1 | Protein Coding | P20036 | 52 | GC06M033064 |
| CCL3 | C-C Motif Chemokine Ligand 3 | Protein Coding | P10147 | 54 | GC17M036088 |
| NF1 | Neurofibromin 1 | Protein Coding | P21359 | 60 | GC17P031094 |
| PMM2 | Phosphomannomutase 2 | Protein Coding | O15305 | 58 | GC16P008788 |
| RPS19 | Ribosomal Protein S19 | Protein Coding | P39019 | 60 | GC19P133127 |
| TIRAP | TIR Domain Containing Adaptor Protein | Protein Coding | P58753 | 54 | GC11P126284 |
| XRCC2 | X-Ray Repair Cross Complementing 2 | Protein Coding | O43543 | 52 | GC07M152644 |
| RECQL4 | RecQ Like Helicase 4 | Protein Coding | O94761 | 52 | GC08M147010 |
| DNASE1L3 | Deoxyribonuclease 1L3 | Protein Coding | Q13609 | 55 | GC03M058192 |
| FANCI | FA Complementation Group I | Protein Coding | Q9NVI1 | 54 | GC15P089243 |
| DHFR | Dihydrofolate Reductase | Protein Coding | P00374 | 61 | GC05M080626 |
| NOS2 | Nitric Oxide Synthase 2 | Protein Coding | P35228 | 62 | GC17M027756 |
| HSPD1 | Heat Shock Protein Family D (Hsp60) Member 1 | Protein Coding | P10809 | 61 | GC02M197486 |
| SGPL1 | Sphingosine-1-Phosphate Lyase 1 | Protein Coding | O95470 | 59 | GC10P070815 |
| CDKN1B | Cyclin Dependent Kinase Inhibitor 1B | Protein Coding | P46527 | 61 | GC12P047017 |
| TNFRSF9 | TNF Receptor Superfamily Member 9 | Protein Coding | Q07011 | 57 | GC01M007915 |
| H19 | H19 Imprinted Maternally Expressed Transcript | RNA Gene |  | 36 | GC11M001995 |
| STAT6 | Signal Transducer And Activator Of Transcription 6 | Protein Coding | P42226 | 63 | GC12M057095 |
| UMPS | Uridine Monophosphate Synthetase | Protein Coding | P11172 | 58 | GC03P124730 |
| TMC6 | Transmembrane Channel Like 6 | Protein Coding | Q7Z403 | 54 | GC17M090466 |
| LINC01672 | Long Intergenic Non-Protein Coding RNA 1672 | RNA Gene |  | 19 | GC01P060062 |
| EP300 | E1A Binding Protein P300 | Protein Coding | Q09472 | 65 | GC22P081600 |
| IL4R | Interleukin 4 Receptor | Protein Coding | P24394 | 60 | GC16P104696 |
| RIPK2 | Receptor Interacting Serine/Threonine Kinase 2 | Protein Coding | O43353 | 58 | GC08P089982 |
| IL33 | Interleukin 33 | Protein Coding | O95760 | 52 | GC09P009892 |
| TPI1 | Triosephosphate Isomerase 1 | Protein Coding | P60174 | 59 | GC12P006867 |
| MYOC | Myocilin | Protein Coding | Q99972 | 54 | GC01M171604 |
| IL1R1 | Interleukin 1 Receptor Type 1 | Protein Coding | P14778 | 59 | GC02P102136 |
| INS | Insulin | Protein Coding | P01308 | 61 | GC11M002159 |
| MANBA | Mannosidase Beta | Protein Coding | O00462 | 55 | GC04M102631 |
| STUB1 | STIP1 Homology And U-Box Containing Protein 1 | Protein Coding | Q9UNE7 | 57 | GC16P103993 |
| CR1 | Complement C3b/C4b Receptor 1 (Knops Blood Group) | Protein Coding | P17927 | 57 | GC01P207496 |
| LTF | Lactotransferrin | Protein Coding | P02788 | 58 | GC03M046435 |
| IL6R | Interleukin 6 Receptor | Protein Coding | P08887 | 63 | GC01P154405 |
| CXCL12 | C-X-C Motif Chemokine Ligand 12 | Protein Coding | P48061 | 59 | GC10M044370 |
| IGLP1 | Immune Response To Synthetic Polypeptides 1 | Genetic Locus |  | 2 | GC06U990137 |
| VCAM1 | Vascular Cell Adhesion Molecule 1 | Protein Coding | P19320 | 58 | GC01P100719 |
| ALG1 | ALG1 Chitobiosyldiphosphodolichol Beta-Mannosyltransferase | Protein Coding | Q9BT22 | 57 | GC16P005033 |
| TNFSF11 | TNF Superfamily Member 11 | Protein Coding | O14788 | 62 | GC13P042562 |
| FCGR1A | Fc Gamma Receptor Ia | Protein Coding | P12314 | 55 | GC01P170158 |
| CCL4 | C-C Motif Chemokine Ligand 4 | Protein Coding | P13236 | 52 | GC17P036103 |
| RAC1 | Rac Family Small GTPase 1 | Protein Coding | P63000 | 62 | GC07P015409 |
| DDX41 | DEAD-Box Helicase 41 | Protein Coding | Q9UJV9 | 55 | GC05M177511 |
| HGSNAT | Heparan-Alpha-Glucosaminide N-Acetyltransferase | Protein Coding | Q68CP4 | 48 | GC08P043140 |
| TPP2 | Tripeptidyl Peptidase 2 | Protein Coding | P29144 | 55 | GC13P102596 |
| ADNP | Activity Dependent Neuroprotector Homeobox | Protein Coding | Q9H2P0 | 55 | GC20M050888 |
| GATA1 | GATA Binding Protein 1 | Protein Coding | P15976 | 59 | GC0XP048786 |
| DEAF1 | DEAF1 Transcription Factor | Protein Coding | O75398 | 52 | GC11M000644 |
| BDNF-AS | BDNF Antisense RNA | RNA Gene |  | 31 | GC11P027466 |
| SLC29A3 | Solute Carrier Family 29 Member 3 | Protein Coding | Q9BZD2 | 55 | GC10P073147 |
| GZMB | Granzyme B | Protein Coding | P10144 | 59 | GC14M024630 |
| MMP13 | Matrix Metallopeptidase 13 | Protein Coding | P45452 | 62 | GC11M102942 |
| IL9 | Interleukin 9 | Protein Coding | P15248 | 55 | GC05M135891 |
| CSF3 | Colony Stimulating Factor 3 | Protein Coding | P09919 | 54 | GC17P040015 |
| SEMA3E | Semaphorin 3E | Protein Coding | O15041 | 56 | GC07M083363 |
| CCBE1 | Collagen And Calcium Binding EGF Domains 1 | Protein Coding | Q6UXH8 | 51 | GC18M059430 |
| RAF1 | Raf-1 Proto-Oncogene, Serine/Threonine Kinase | Protein Coding | P04049 | 67 | GC03M012583 |
| MET | MET Proto-Oncogene, Receptor Tyrosine Kinase | Protein Coding | P08581 | 67 | GC07P116672 |
| LOC126861898 | BRD4-Independent Group 4 Enhancer GRCh37_chr14:23893609-23894808 | Functional Element |  | 9 | GC14P052820 |
| THBD | Thrombomodulin | Protein Coding | P07204 | 58 | GC20M023026 |
| CYLD | CYLD Lysine 63 Deubiquitinase | Protein Coding | Q9NQC7 | 60 | GC16P050742 |
| CDSN | Corneodesmosin | Protein Coding | Q15517 | 52 | GC06M031115 |
| COPB1 | COPI Coat Complex Subunit Beta 1 | Protein Coding | P53618 | 52 | GC11M014436 |
| SMARCD2 | SWI/SNF Related, Matrix Associated, Actin Dependent Regulator Of Chromatin, Subfamily D, Member 2 | Protein Coding | Q92925 | 52 | GC17M063832 |
| SMAD4 | SMAD Family Member 4 | Protein Coding | Q13485 | 66 | GC18P051028 |
| MAPK14 | Mitogen-Activated Protein Kinase 14 | Protein Coding | Q16539 | 63 | GC06P156437 |
| ALB | Albumin | Protein Coding | P02768 | 61 | GC04P073397 |
| PCCA | Propionyl-CoA Carboxylase Subunit Alpha | Protein Coding | P05165 | 59 | GC13P100089 |
| HLA-E | Major Histocompatibility Complex, Class I, E | Protein Coding | P13747 | 53 | GC06P156218 |
| TBXAS1 | Thromboxane A Synthase 1 | Protein Coding | P24557 | 61 | GC07P139777 |
| TBX21 | T-Box Transcription Factor 21 | Protein Coding | Q9UL17 | 59 | GC17P047733 |
| CYP27B1 | Cytochrome P450 Family 27 Subfamily B Member 1 | Protein Coding | O15528 | 58 | GC12M059933 |
| GALNS | Galactosamine (N-Acetyl)-6-Sulfatase | Protein Coding | P34059 | 61 | GC16M088813 |
| RPS17 | Ribosomal Protein S17 | Protein Coding | P08708 | 54 | GC15M082536 |
| LAG3 | Lymphocyte Activating 3 | Protein Coding | P18627 | 52 | GC12P046770 |
| PDGFRA | Platelet Derived Growth Factor Receptor Alpha | Protein Coding | P16234 | 66 | GC04P054229 |
| EFL1 | Elongation Factor Like GTPase 1 | Protein Coding | Q7Z2Z2 | 51 | GC15M155758 |
| RET | Ret Proto-Oncogene | Protein Coding | P07949 | 67 | GC10P045251 |
| RNASEH2C | Ribonuclease H2 Subunit C | Protein Coding | Q8TDP1 | 51 | GC11M065714 |
| LY96 | Lymphocyte Antigen 96 | Protein Coding | Q9Y6Y9 | 56 | GC08P073991 |
| WT1 | WT1 Transcription Factor | Protein Coding | P19544 | 61 | GC11M032365 |
| BIRC3 | Baculoviral IAP Repeat Containing 3 | Protein Coding | Q13489 | 58 | GC11P102317 |
| ATP6AP1 | ATPase H+ Transporting Accessory Protein 1 | Protein Coding | Q15904 | 57 | GC0XP154428 |
| TMX2-CTNND1 | TMX2-CTNND1 Readthrough (NMD Candidate) | RNA Gene |  | 26 | GC11P057712 |
| BTNL2 | Butyrophilin Like 2 | Protein Coding | Q9UIR0 | 52 | GC06M032393 |
| ALG12 | ALG12 Alpha-1,6-Mannosyltransferase | Protein Coding | Q9BV10 | 50 | GC22M049859 |
| MIR7-3HG | MIR7-3 Host Gene | RNA Gene | Q8N6C7 | 34 | GC19P132003 |
| KLRD1 | Killer Cell Lectin Like Receptor D1 | Protein Coding | Q13241 | 53 | GC12P010226 |
| TMC8 | Transmembrane Channel Like 8 | Protein Coding | Q8IU68 | 51 | GC17P078130 |
| ITGAL | Integrin Subunit Alpha L | Protein Coding | P20701 | 60 | GC16P030472 |
| SLC39A8 | Solute Carrier Family 39 Member 8 | Protein Coding | Q9C0K1 | 56 | GC04M102252 |
| TOP2B | DNA Topoisomerase II Beta | Protein Coding | Q02880 | 57 | GC03M027595 |
| MTOR | Mechanistic Target Of Rapamycin Kinase | Protein Coding | P42345 | 68 | GC01M011106 |
| MMAB | Metabolism Of Cobalamin Associated B | Protein Coding | Q96EY8 | 55 | GC12M109553 |
| RPS26 | Ribosomal Protein S26 | Protein Coding | P62854 | 55 | GC12P065353 |
| EPCAM | Epithelial Cell Adhesion Molecule | Protein Coding | P16422 | 61 | GC02P047345 |
| LILRB1 | Leukocyte Immunoglobulin Like Receptor B1 | Protein Coding | Q8NHL6 | 54 | GC19P133676 |
| CD27-AS1 | CD27 Antisense RNA 1 | RNA Gene |  | 20 | GC12M006439 |
| TREM2 | Triggering Receptor Expressed On Myeloid Cells 2 | Protein Coding | Q9NZC2 | 56 | GC06M100502 |
| C5AR1 | Complement C5a Receptor 1 | Protein Coding | P21730 | 58 | GC19P047290 |
| NFATC2 | Nuclear Factor Of Activated T Cells 2 | Protein Coding | Q13469 | 59 | GC20M051386 |
| TALDO1 | Transaldolase 1 | Protein Coding | P37837 | 59 | GC11P014054 |
| ALK | ALK Receptor Tyrosine Kinase | Protein Coding | Q9UM73 | 64 | GC02M029190 |
| MC2R | Melanocortin 2 Receptor | Protein Coding | Q01718 | 58 | GC18M036029 |
| KLRC1 | Killer Cell Lectin Like Receptor C1 | Protein Coding | P26715 | 54 | GC12M033118 |
| SLC35A1 | Solute Carrier Family 35 Member A1 | Protein Coding | P78382 | 52 | GC06P087470 |
| MMACHC | Metabolism Of Cobalamin Associated C | Protein Coding | Q9Y4U1 | 55 | GC01P045500 |
| IGLP2 | Immune Response To Synthetic Polypeptides 2 | Genetic Locus |  | 2 | GC06U990138 |
| SLC7A7 | Solute Carrier Family 7 Member 7 | Protein Coding | Q9UM01 | 56 | GC14M022773 |
| RPL11 | Ribosomal Protein L11 | Protein Coding | P62913 | 60 | GC01P023691 |
| TSLP | Thymic Stromal Lymphopoietin | Protein Coding | Q969D9 | 52 | GC05P111070 |
| SPP1 | Secreted Phosphoprotein 1 | Protein Coding | P10451 | 56 | GC04P087975 |
| RPL5 | Ribosomal Protein L5 | Protein Coding | P46777 | 59 | GC01P092855 |
| PSMA3 | Proteasome 20S Subunit Alpha 3 | Protein Coding | P25788 | 57 | GC14P058244 |
| ANGPT2 | Angiopoietin 2 | Protein Coding | O15123 | 61 | GC08M006499 |
| CHD1 | Chromodomain Helicase DNA Binding Protein 1 | Protein Coding | O14646 | 56 | GC05M098853 |
| LTA | Lymphotoxin Alpha | Protein Coding | P01374 | 55 | GC06P156242 |
| RPL35A | Ribosomal Protein L35a | Protein Coding | P18077 | 54 | GC03P199778 |
| GCSIR | GPR55 Cis Regulatory Suppressor Of Immune Response RNA | RNA Gene |  | 13 | GC02P230888 |
| RBM8A | RNA Binding Motif Protein 8A | Protein Coding | Q9Y5S9 | 54 | GC01M145921 |
| EZH2 | Enhancer Of Zeste 2 Polycomb Repressive Complex 2 Subunit | Protein Coding | Q15910 | 66 | GC07M148807 |
| TRPV4 | Transient Receptor Potential Cation Channel Subfamily V Member 4 | Protein Coding | Q9HBA0 | 61 | GC12M109783 |
| TNFRSF1B | TNF Receptor Superfamily Member 1B | Protein Coding | P20333 | 61 | GC01P060401 |
| MIR34A | MicroRNA 34a | RNA Gene |  | 31 | GC01M019769 |
| RPS24 | Ribosomal Protein S24 | Protein Coding | P62847 | 55 | GC10P078033 |
| MMUT | Methylmalonyl-CoA Mutase | Protein Coding | P22033 | 58 | GC06M049430 |
| UBE2T | Ubiquitin Conjugating Enzyme E2 T | Protein Coding | Q9NPD8 | 55 | GC01M202332 |
| BCL6 | BCL6 Transcription Repressor | Protein Coding | P41182 | 59 | GC03M187721 |
| CCDC40 | Coiled-Coil Domain 40 Molecular Ruler Complex Subunit | Protein Coding | Q4G0X9 | 47 | GC17P134782 |
| COLEC11 | Collectin Subfamily Member 11 | Protein Coding | Q9BWP8 | 52 | GC02P003594 |
| FBXL4 | F-Box And Leucine Rich Repeat Protein 4 | Protein Coding | Q9UKA2 | 51 | GC06M098868 |
| MAPK3 | Mitogen-Activated Protein Kinase 3 | Protein Coding | P27361 | 62 | GC16M045720 |
| CASP1 | Caspase 1 | Protein Coding | P29466 | 61 | GC11M105025 |
| NFASC | Neurofascin | Protein Coding | O94856 | 56 | GC01P204828 |
| ARG1 | Arginase 1 | Protein Coding | P05089 | 62 | GC06P158145 |
| DEFB4A | Defensin Beta 4A | Protein Coding | O15263 | 48 | GC08P010295 |
| MAD2L2 | Mitotic Arrest Deficient 2 Like 2 | Protein Coding | Q9UI95 | 55 | GC01M011658 |
| INPP5D | Inositol Polyphosphate-5-Phosphatase D | Protein Coding | Q92835 | 58 | GC02P233059 |
| LCIIAR | Lung Cancer Immune Cell Infiltration Associated LncRNA | RNA Gene |  | 17 | GC15P177162 |
| TSC2 | TSC Complex Subunit 2 | Protein Coding | P49815 | 64 | GC16P104050 |
| HBA2 | Hemoglobin Subunit Alpha 2 | Protein Coding | P69905 | 52 | GC16P103972 |
| CSF2RB | Colony Stimulating Factor 2 Receptor Subunit Beta | Protein Coding | P32927 | 58 | GC22P036913 |
| CCL11 | C-C Motif Chemokine Ligand 11 | Protein Coding | P51671 | 58 | GC17P034285 |
| ERBB2 | Erb-B2 Receptor Tyrosine Kinase 2 | Protein Coding | P04626 | 68 | GC17P039687 |
| IGHG1 | Immunoglobulin Heavy Constant Gamma 1 (G1m Marker) | Protein Coding | P01857 | 45 | GC14M105736 |
| MAP3K7 | Mitogen-Activated Protein Kinase Kinase Kinase 7 | Protein Coding | O43318 | 63 | GC06M090513 |
| JUN | Jun Proto-Oncogene, AP-1 Transcription Factor Subunit | Protein Coding | P05412 | 62 | GC01M058780 |
| FLT3 | Fms Related Receptor Tyrosine Kinase 3 | Protein Coding | P36888 | 66 | GC13M028003 |
| COG6 | Component Of Oligomeric Golgi Complex 6 | Protein Coding | Q9Y2V7 | 51 | GC13P039655 |
| STN1 | STN1 Subunit Of CST Complex | Protein Coding | Q9H668 | 52 | GC10M104222 |
| IFNA2 | Interferon Alpha 2 | Protein Coding | P01563 | 55 | GC09M021384 |
| HYDIN | HYDIN Axonemal Central Pair Apparatus Protein | Protein Coding | Q4G0P3 | 51 | GC16M073865 |
| IGAT | Immune Response To Synthetic Polypeptide--IRGAT | Genetic Locus |  | 1 | GC06U990139 |
| ISCW | Immune Suppression To Streptococcal Antigen | Genetic Locus |  | 1 | GC06U990140 |
| AGA | Aspartylglucosaminidase | Protein Coding | P20933 | 58 | GC04M177430 |
| PSMB9 | Proteasome 20S Subunit Beta 9 | Protein Coding | P28065 | 59 | GC06P156277 |
| SPAG1 | Sperm Associated Antigen 1 | Protein Coding | Q07617 | 52 | GC08P100157 |
| PGLYRP1 | Peptidoglycan Recognition Protein 1 | Protein Coding | O75594 | 49 | GC19M101151 |
| PCCB | Propionyl-CoA Carboxylase Subunit Beta | Protein Coding | P05166 | 59 | GC03P136250 |
| CP | Ceruloplasmin | Protein Coding | P00450 | 61 | GC03M149162 |
| KIR3DL1 | Killer Cell Immunoglobulin Like Receptor, Three Ig Domains And Long Cytoplasmic Tail 1 | Protein Coding | P43629 | 51 | GC19P133684 |
| SCNN1B | Sodium Channel Epithelial 1 Subunit Beta | Protein Coding | P51168 | 61 | GC16P023278 |
| MIR223 | MicroRNA 223 | RNA Gene |  | 30 | GC0XP066018 |
| ARID1A | AT-Rich Interaction Domain 1A | Protein Coding | O14497 | 58 | GC01P026693 |
| RPS10 | Ribosomal Protein S10 | Protein Coding | P46783 | 55 | GC06M100435 |
| TNFSF4 | TNF Superfamily Member 4 | Protein Coding | P23510 | 54 | GC01M173183 |
| SHARPIN | SHANK Associated RH Domain Interactor | Protein Coding | Q9H0F6 | 50 | GC08M144098 |
| CPN1 | Carboxypeptidase N Subunit 1 | Protein Coding | P15169 | 55 | GC10M100042 |
| XDH | Xanthine Dehydrogenase | Protein Coding | P47989 | 60 | GC02M031334 |
| SLC39A4 | Solute Carrier Family 39 Member 4 | Protein Coding | Q6P5W5 | 54 | GC08M144409 |
| STAT5A | Signal Transducer And Activator Of Transcription 5A | Protein Coding | P42229 | 62 | GC17P042287 |
| POLA1 | DNA Polymerase Alpha 1, Catalytic Subunit | Protein Coding | P09884 | 58 | GC0XP024693 |
| LCP2 | Lymphocyte Cytosolic Protein 2 | Protein Coding | Q13094 | 55 | GC05M170246 |
| IL6ST | Interleukin 6 Cytokine Family Signal Transducer | Protein Coding | P40189 | 62 | GC05M055935 |
| LOC130058479 | ATAC-STARR-Seq Lymphoblastoid Silent Region 7198 | Functional Element |  | 9 | GC16P110470 |
| COPA | COPI Coat Complex Subunit Alpha | Protein Coding | P53621 | 55 | GC01M160288 |
| NOTCH1 | Notch Receptor 1 | Protein Coding | P46531 | 65 | GC09M138976 |
| AHR | Aryl Hydrocarbon Receptor | Protein Coding | P35869 | 60 | GC07P016916 |
| RFWD3 | Ring Finger And WD Repeat Domain 3 | Protein Coding | Q6PCD5 | 51 | GC16M074621 |
| SMARCB1 | SWI/SNF Related, Matrix Associated, Actin Dependent Regulator Of Chromatin, Subfamily B, Member 1 | Protein Coding | Q12824 | 59 | GC22P023786 |
| CEBPA | CCAAT Enhancer Binding Protein Alpha | Protein Coding | P49715 | 59 | GC19M033299 |
| FAT4 | FAT Atypical Cadherin 4 | Protein Coding | Q6V0I7 | 51 | GC04P125315 |
| DNAAF5 | Dynein Axonemal Assembly Factor 5 | Protein Coding | Q86Y56 | 47 | GC07P000726 |
| PTPN6 | Protein Tyrosine Phosphatase Non-Receptor Type 6 | Protein Coding | P29350 | 62 | GC12P046789 |
| DNAAF2 | Dynein Axonemal Assembly Factor 2 | Protein Coding | Q9NVR5 | 47 | GC14M049625 |
| CCNO | Cyclin O | Protein Coding | P22674 | 53 | GC05M055231 |
| RPL26 | Ribosomal Protein L26 | Protein Coding | P61254 | 55 | GC17M008377 |
| STK11 | Serine/Threonine Kinase 11 | Protein Coding | Q15831 | 63 | GC19P001177 |
| IL3 | Interleukin 3 | Protein Coding | P08700 | 57 | GC05P132060 |
| TRPS1 | Transcriptional Repressor GATA Binding 1 | Protein Coding | Q9UHF7 | 56 | GC08M115408 |
| COG7 | Component Of Oligomeric Golgi Complex 7 | Protein Coding | P83436 | 50 | GC16M023388 |
| SDCCAG8 | SHH Signaling And Ciliogenesis Regulator SDCCAG8 | Protein Coding | Q86SQ7 | 53 | GC01P243255 |
| GAS5 | Growth Arrest Specific 5 | RNA Gene |  | 32 | GC01M173947 |
| MBP | Myelin Basic Protein | Protein Coding | P02686 | 57 | GC18M076978 |
| VIPAS39 | VPS33B Interacting Protein, Apical-Basolateral Polarity Regulator, Spe-39 Homolog | Protein Coding | Q9H9C1 | 48 | GC14M077426 |
| DNAAF11 | Dynein Axonemal Assembly Factor 11 | Protein Coding | Q86X45 | 47 | GC08M136816 |
| CCR2 | C-C Motif Chemokine Receptor 2 | Protein Coding | P41597 | 58 | GC03P061354 |
| DNASE2 | Deoxyribonuclease 2, Lysosomal | Protein Coding | O00115 | 53 | GC19M012875 |
| PTX3 | Pentraxin 3 | Protein Coding | P26022 | 55 | GC03P157436 |
| DNAL1 | Dynein Axonemal Light Chain 1 | Protein Coding | Q4LDG9 | 51 | GC14P073644 |
| CAVIN1 | Caveolae Associated Protein 1 | Protein Coding | Q6NZI2 | 54 | GC17M089365 |
| IL18R1 | Interleukin 18 Receptor 1 | Protein Coding | Q13478 | 56 | GC02P102311 |
| TRIM21 | Tripartite Motif Containing 21 | Protein Coding | P19474 | 55 | GC11M004384 |
| VDR | Vitamin D Receptor | Protein Coding | P11473 | 61 | GC12M047841 |
| FYN | FYN Proto-Oncogene, Src Family Tyrosine Kinase | Protein Coding | P06241 | 60 | GC06M111660 |
| NF2 | NF2, Moesin-Ezrin-Radixin Like (MERLIN) Tumor Suppressor | Protein Coding | P35240 | 62 | GC22P029603 |
| ODAD3 | Outer Dynein Arm Docking Complex Subunit 3 | Protein Coding | A5D8V7 | 47 | GC19M100303 |
| AKT2 | AKT Serine/Threonine Kinase 2 | Protein Coding | P31751 | 66 | GC19M040230 |
| IL37 | Interleukin 37 | Protein Coding | Q9NZH6 | 50 | GC02P148587 |
| F12 | Coagulation Factor XII | Protein Coding | P00748 | 61 | GC05M177402 |
| CCDC39 | Coiled-Coil Domain 39 Molecular Ruler Complex Subunit | Protein Coding | Q9UFE4 | 48 | GC03M180602 |
| MASP2 | MBL Associated Serine Protease 2 | Protein Coding | O00187 | 59 | GC01M019809 |
| SRC | SRC Proto-Oncogene, Non-Receptor Tyrosine Kinase | Protein Coding | P12931 | 63 | GC20P037344 |
| TGFB2 | Transforming Growth Factor Beta 2 | Protein Coding | P61812 | 64 | GC01P218345 |
| MAPK8 | Mitogen-Activated Protein Kinase 8 | Protein Coding | P45983 | 62 | GC10P048306 |
| LOC129935461 | ATAC-STARR-Seq Lymphoblastoid Active Region 17016 | Functional Element |  | 5 | GC02P203871 |
| LILRB2 | Leukocyte Immunoglobulin Like Receptor B2 | Protein Coding | Q8N423 | 51 | GC19M101520 |
| TLR10 | Toll Like Receptor 10 | Protein Coding | Q9BXR5 | 50 | GC04M038773 |
| CCR6 | C-C Motif Chemokine Receptor 6 | Protein Coding | P51684 | 55 | GC06P167111 |
| DNAAF1 | Dynein Axonemal Assembly Factor 1 | Protein Coding | Q8NEP3 | 47 | GC16P084145 |
| RPS29 | Ribosomal Protein S29 | Protein Coding | P62273 | 53 | GC14M049570 |
| SLC11A1 | Solute Carrier Family 11 Member 1 | Protein Coding | P49279 | 58 | GC02P218382 |
| SOD1 | Superoxide Dismutase 1 | Protein Coding | P00441 | 66 | GC21P031659 |
| CD244 | CD244 Molecule | Protein Coding | Q9BZW8 | 56 | GC01M160830 |
| CSF1 | Colony Stimulating Factor 1 | Protein Coding | P09603 | 57 | GC01P109917 |
| DNAI1 | Dynein Axonemal Intermediate Chain 1 | Protein Coding | Q9UI46 | 54 | GC09P034457 |
| LAMP1 | Lysosomal Associated Membrane Protein 1 | Protein Coding | P11279 | 57 | GC13P113297 |
| SOCS3 | Suppressor Of Cytokine Signaling 3 | Protein Coding | O14543 | 56 | GC17M078356 |
| MIR17 | MicroRNA 17 | RNA Gene |  | 28 | GC13P091350 |
| SH2B3 | SH2B Adaptor Protein 3 | Protein Coding | Q9UQQ2 | 58 | GC12P111405 |
| CXCL9 | C-X-C Motif Chemokine Ligand 9 | Protein Coding | Q07325 | 51 | GC04M076001 |
| TAFAZZIN | Tafazzin, Phospholipid-Lysophospholipid Transacylase | Protein Coding | Q16635 | 54 | GC0XP154968 |
| CHAMP1 | Chromosome Alignment Maintaining Phosphoprotein 1 | Protein Coding | Q96JM3 | 50 | GC13P114314 |
| HLA-DRB5 | Major Histocompatibility Complex, Class II, DR Beta 5 | Protein Coding | Q30154 | 52 | GC06M100348 |
| DGKE | Diacylglycerol Kinase Epsilon | Protein Coding | P52429 | 58 | GC17P056834 |
| HLA-DRB3 | Major Histocompatibility Complex, Class II, DR Beta 3 | Protein Coding | P79483 | 37 | GC06Mn03715 |
| IFNG-AS1 | IFNG Antisense RNA 1 | RNA Gene |  | 27 | GC12P067989 |
| VPS33B | VPS33B Late Endosome And Lysosome Associated | Protein Coding | Q9H267 | 54 | GC15M090998 |
| NR3C1 | Nuclear Receptor Subfamily 3 Group C Member 1 | Protein Coding | P04150 | 62 | GC05M143277 |
| RN7SK | RNA Component Of 7SK Nuclear Ribonucleoprotein | RNA Gene |  | 26 | GC06P052995 |
| SELL | Selectin L | Protein Coding | P14151 | 55 | GC01M169690 |
| SERAC1 | Serine Active Site Containing 1 | Protein Coding | Q96JX3 | 49 | GC06M158109 |
| CD69 | CD69 Molecule | Protein Coding | Q07108 | 54 | GC12M033105 |
| DNAH11 | Dynein Axonemal Heavy Chain 11 | Protein Coding | Q96DT5 | 55 | GC07P021543 |
| RSPH9 | Radial Spoke Head Component 9 | Protein Coding | Q9H1X1 | 48 | GC06P156506 |
| IRF9 | Interferon Regulatory Factor 9 | Protein Coding | Q00978 | 57 | GC14P024161 |
| IKBKE | Inhibitor Of Nuclear Factor Kappa B Kinase Subunit Epsilon | Protein Coding | Q14164 | 58 | GC01P206470 |
| DNAI2 | Dynein Axonemal Intermediate Chain 2 | Protein Coding | Q9GZS0 | 50 | GC17P074274 |
| DNAAF4 | Dynein Axonemal Assembly Factor 4 | Protein Coding | Q8WXU2 | 48 | GC15M156302 |
| DNAH5 | Dynein Axonemal Heavy Chain 5 | Protein Coding | Q8TE73 | 51 | GC05M013695 |
| IFI16 | Interferon Gamma Inducible Protein 16 | Protein Coding | Q16666 | 55 | GC01P158999 |
| TRAF2 | TNF Receptor Associated Factor 2 | Protein Coding | Q12933 | 58 | GC09P136881 |
| ATR | ATR Serine/Threonine Kinase | Protein Coding | Q13535 | 66 | GC03M142449 |
| MMAA | Metabolism Of Cobalamin Associated A | Protein Coding | Q8IVH4 | 50 | GC04P145647 |
| IL27RA | Interleukin 27 Receptor Subunit Alpha | Protein Coding | Q6UWB1 | 52 | GC19P014031 |
| IGF1 | Insulin Like Growth Factor 1 | Protein Coding | P05019 | 61 | GC12M102395 |
| IDH1 | Isocitrate Dehydrogenase (NADP(+)) 1 | Protein Coding | O75874 | 65 | GC02M208236 |
| BCL2L1 | BCL2 Like 1 | Protein Coding | Q07817 | 62 | GC20M031664 |
| MIR142 | MicroRNA 142 | RNA Gene |  | 30 | GC17M058331 |
| PLA2G6 | Phospholipase A2 Group VI | Protein Coding | O60733 | 59 | GC22M083204 |
| HTR1A | 5-Hydroxytryptamine Receptor 1A | Protein Coding | P08908 | 58 | GC05M063960 |
| IL12RB2 | Interleukin 12 Receptor Subunit Beta 2 | Protein Coding | Q99665 | 56 | GC01P067307 |
| ALOX5 | Arachidonate 5-Lipoxygenase | Protein Coding | P09917 | 62 | GC10P045374 |
| HCK | HCK Proto-Oncogene, Src Family Tyrosine Kinase | Protein Coding | P08631 | 61 | GC20P032052 |
| HBA1 | Hemoglobin Subunit Alpha 1 | Protein Coding | P69905 | 55 | GC16P103971 |
| CXCR2 | C-X-C Motif Chemokine Receptor 2 | Protein Coding | P25025 | 62 | GC02P218125 |
| SPI1 | Spi-1 Proto-Oncogene | Protein Coding | P17947 | 56 | GC11M132469 |
| IVD | Isovaleryl-CoA Dehydrogenase | Protein Coding | P26440 | 56 | GC15P040405 |
| LZTR1 | Leucine Zipper Like Post Translational Regulator 1 | Protein Coding | Q8N653 | 55 | GC22P080906 |
| CSF2RA | Colony Stimulating Factor 2 Receptor Subunit Alpha | Protein Coding | P15509 | 56 | GC0XP002675 |
| ACTA1 | Actin Alpha 1, Skeletal Muscle | Protein Coding | P68133 | 61 | GC01M230430 |
| A2ML1 | Alpha-2-Macroglobulin Like 1 | Protein Coding | A8K2U0 | 51 | GC12P008822 |
| MGP | Matrix Gla Protein | Protein Coding | P08493 | 55 | GC12M033183 |
| RPS7 | Ribosomal Protein S7 | Protein Coding | P62081 | 54 | GC02P003575 |
| PPARG | Peroxisome Proliferator Activated Receptor Gamma | Protein Coding | P37231 | 65 | GC03P012287 |
| SLC35A2 | Solute Carrier Family 35 Member A2 | Protein Coding | P78381 | 52 | GC0XM048903 |
| ZMYND10 | Zinc Finger MYND-Type Containing 10 | Protein Coding | O75800 | 50 | GC03M054335 |
| ZNF341 | Zinc Finger Protein 341 | Protein Coding | Q9BYN7 | 45 | GC20P033731 |
| DNAH1 | Dynein Axonemal Heavy Chain 1 | Protein Coding | Q9P2D7 | 46 | GC03P061631 |
| CXCR3 | C-X-C Motif Chemokine Receptor 3 | Protein Coding | P49682 | 57 | GC0XM071615 |
| FOXC2 | Forkhead Box C2 | Protein Coding | Q99958 | 55 | GC16P106769 |
| MITF | Melanocyte Inducing Transcription Factor | Protein Coding | O75030 | 61 | GC03P069740 |
| RPL15 | Ribosomal Protein L15 | Protein Coding | P61313 | 56 | GC03P023916 |
| CCR7 | C-C Motif Chemokine Receptor 7 | Protein Coding | P32248 | 57 | GC17M089298 |
| XIST | X Inactive Specific Transcript | RNA Gene |  | 32 | GC0XM073820 |
| ODAD2 | Outer Dynein Arm Docking Complex Subunit 2 | Protein Coding | Q5T2S8 | 47 | GC10M028460 |
| DRC1 | Dynein Regulatory Complex Subunit 1 | Protein Coding | Q96MC2 | 45 | GC02P026401 |
| MIR145 | MicroRNA 145 | RNA Gene |  | 32 | GC05P149430 |
| LBP | Lipopolysaccharide Binding Protein | Protein Coding | P18428 | 55 | GC20P038346 |
| DICER1 | Dicer 1, Ribonuclease III | Protein Coding | Q9UPY3 | 61 | GC14M095086 |
| S100A9 | S100 Calcium Binding Protein A9 | Protein Coding | P06702 | 56 | GC01P153357 |
| APC | APC Regulator Of WNT Signaling Pathway | Protein Coding | P25054 | 62 | GC05P112707 |
| TRIM22 | Tripartite Motif Containing 22 | Protein Coding | Q8IYM9 | 51 | GC11P005689 |
| ACE2 | Angiotensin Converting Enzyme 2 | Protein Coding | Q9BYF1 | 62 | GC0XM015494 |
| SLPI | Secretory Leukocyte Peptidase Inhibitor | Protein Coding | P03973 | 52 | GC20M045252 |
| HIF1A | Hypoxia Inducible Factor 1 Subunit Alpha | Protein Coding | Q16665 | 61 | GC14P061695 |
| MIR140 | MicroRNA 140 | RNA Gene |  | 33 | GC16P106041 |
| FMO3 | Flavin Containing Dimethylaniline Monoxygenase 3 | Protein Coding | P31513 | 58 | GC01P171090 |
| MX1 | MX Dynamin Like GTPase 1 | Protein Coding | P20591 | 52 | GC21P041420 |
| CD276 | CD276 Molecule | Protein Coding | Q5ZPR3 | 55 | GC15P073683 |
| IFNAR1 | Interferon Alpha And Beta Receptor Subunit 1 | Protein Coding | P17181 | 61 | GC21P033324 |
| PIK3CB | Phosphatidylinositol-4,5-Bisphosphate 3-Kinase Catalytic Subunit Beta | Protein Coding | P42338 | 62 | GC03M138652 |
| GAS8 | Growth Arrest Specific 8 | Protein Coding | O95995 | 51 | GC16P090019 |
| ETS1 | ETS Proto-Oncogene 1, Transcription Factor | Protein Coding | P14921 | 61 | GC11M128458 |
| IGF1R | Insulin Like Growth Factor 1 Receptor | Protein Coding | P08069 | 68 | GC15P098648 |
| GSS | Glutathione Synthetase | Protein Coding | P48637 | 59 | GC20M034928 |
| P2RX7 | Purinergic Receptor P2X 7 | Protein Coding | Q99572 | 59 | GC12P136098 |
| CASP3 | Caspase 3 | Protein Coding | P42574 | 62 | GC04M184627 |
| RSPH1 | Radial Spoke Head Component 1 | Protein Coding | Q8WYR4 | 50 | GC21M042472 |
| IFNL3 | Interferon Lambda 3 | Protein Coding | Q8IZI9 | 48 | GC19M039243 |
| MSH2 | MutS Homolog 2 | Protein Coding | P43246 | 61 | GC02P047403 |
| PAX5 | Paired Box 5 | Protein Coding | Q02548 | 59 | GC09M037094 |
| LBR | Lamin B Receptor | Protein Coding | Q14739 | 59 | GC01M225401 |
| RPS28 | Ribosomal Protein S28 | Protein Coding | P62857 | 51 | GC19P132169 |
| RSPH4A | Radial Spoke Head Component 4A | Protein Coding | Q5TD94 | 48 | GC06P116616 |
| RPL18 | Ribosomal Protein L18 | Protein Coding | Q07020 | 55 | GC19M048615 |
| APOE | Apolipoprotein E | Protein Coding | P02649 | 62 | GC19P133238 |
| PRKAR1A | Protein Kinase CAMP-Dependent Type I Regulatory Subunit Alpha | Protein Coding | P10644 | 63 | GC17P134455 |
| CFAP300 | Cilia And Flagella Associated Protein 300 | Protein Coding | Q9BRQ4 | 44 | GC11P102077 |
| DNAAF3 | Dynein Axonemal Assembly Factor 3 | Protein Coding | Q8N9W5 | 47 | GC19M055158 |
| CCDC103 | Coiled-Coil Domain Containing 103 | Protein Coding | Q8IW40 | 45 | GC17P133861 |
| GTF2H5 | General Transcription Factor IIH Subunit 5 | Protein Coding | Q6ZYL4 | 51 | GC06P158168 |
| SCNN1G | Sodium Channel Epithelial 1 Subunit Gamma | Protein Coding | P51170 | 59 | GC16P023182 |
| RNF113A | Ring Finger Protein 113A | Protein Coding | O15541 | 49 | GC0XM119870 |
| HP | Haptoglobin | Protein Coding | P00738 | 58 | GC16P106129 |
| PTPN2 | Protein Tyrosine Phosphatase Non-Receptor Type 2 | Protein Coding | P17706 | 59 | GC18M036022 |
| AFG2A | AFG2 AAA ATPase Homolog A | Protein Coding | Q8NB90 | 52 | GC04P122923 |
| PRL | Prolactin | Protein Coding | P01236 | 56 | GC06M022287 |
| GTF3A | General Transcription Factor IIIA | Protein Coding | Q92664 | 45 | GC13P028175 |
| GPI | Glucose-6-Phosphate Isomerase | Protein Coding | P06744 | 59 | GC19P034359 |
| B3GALT6 | Beta-1,3-Galactosyltransferase 6 | Protein Coding | Q96L58 | 51 | GC01P001232 |
| FCER2 | Fc Epsilon Receptor II | Protein Coding | P06734 | 55 | GC19M100150 |
| KARS1 | Lysyl-TRNA Synthetase 1 | Protein Coding | Q15046 | 58 | GC16M076057 |
| KIR2DL4 | Killer Cell Immunoglobulin Like Receptor, Two Ig Domains And Long Cytoplasmic Tail 4 | Protein Coding | Q99706 | 48 | GC19P133683 |
| WDR1 | WD Repeat Domain 1 | Protein Coding | O75083 | 52 | GC04M010073 |
| CFAP418 | Cilia And Flagella Associated Protein 418 | Protein Coding | Q96NL8 | 43 | GC08M095245 |
| ITGB3 | Integrin Subunit Beta 3 | Protein Coding | P05106 | 65 | GC17P134000 |
| LCN2 | Lipocalin 2 | Protein Coding | P80188 | 58 | GC09P128149 |
| KIR2DL3 | Killer Cell Immunoglobulin Like Receptor, Two Ig Domains And Long Cytoplasmic Tail 3 | Protein Coding | P43628 | 48 | GC19P134624 |
| BLK | BLK Proto-Oncogene, Src Family Tyrosine Kinase | Protein Coding | P51451 | 61 | GC08P011486 |
| TAB2 | TGF-Beta Activated Kinase 1 (MAP3K7) Binding Protein 2 | Protein Coding | Q9NYJ8 | 60 | GC06P163603 |
| SNAI2 | Snail Family Transcriptional Repressor 2 | Protein Coding | O43623 | 55 | GC08M048917 |
| TREM1 | Triggering Receptor Expressed On Myeloid Cells 1 | Protein Coding | Q9NP99 | 55 | GC06M041267 |
| VTCN1 | V-Set Domain Containing T Cell Activation Inhibitor 1 | Protein Coding | Q7Z7D3 | 52 | GC01M117143 |
| TRIM5 | Tripartite Motif Containing 5 | Protein Coding | Q9C035 | 54 | GC11M013462 |
| IL27 | Interleukin 27 | Protein Coding | Q8NEV9 | 48 | GC16M045528 |
| CSF1R | Colony Stimulating Factor 1 Receptor | Protein Coding | P07333 | 65 | GC05M150053 |
| IRAK3 | Interleukin 1 Receptor Associated Kinase 3 | Protein Coding | Q9Y616 | 59 | GC12P068109 |
| CLEC4D | C-Type Lectin Domain Family 4 Member D | Protein Coding | Q8WXI8 | 47 | GC12P008509 |
| PTGS2 | Prostaglandin-Endoperoxide Synthase 2 | Protein Coding | P35354 | 62 | GC01M186671 |
| LEPR | Leptin Receptor | Protein Coding | P48357 | 62 | GC01P067643 |
| CX3CR1 | C-X3-C Motif Chemokine Receptor 1 | Protein Coding | P49238 | 57 | GC03M039279 |
| RIT1 | Ras Like Without CAAX 1 | Protein Coding | Q92963 | 58 | GC01M155897 |
| TSR2 | TSR2 Ribosome Maturation Factor | Protein Coding | Q969E8 | 47 | GC0XP059491 |
| ICAM2 | Intercellular Adhesion Molecule 2 | Protein Coding | P13598 | 56 | GC17M064002 |
| DNMT3A | DNA Methyltransferase 3 Alpha | Protein Coding | Q9Y6K1 | 64 | GC02M025228 |
| DHX58 | DExH-Box Helicase 58 | Protein Coding | Q96C10 | 50 | GC17M042101 |
| FOS | Fos Proto-Oncogene, AP-1 Transcription Factor Subunit | Protein Coding | P01100 | 63 | GC14P075278 |
| CD44 | CD44 Molecule (IN Blood Group) | Protein Coding | P16070 | 61 | GC11P035139 |
| NME8 | NME/NM23 Family Member 8 | Protein Coding | Q8N427 | 51 | GC07P037892 |
| CCDC65 | Coiled-Coil Domain Containing 65 | Protein Coding | Q8IXS2 | 48 | GC12P048904 |
| ODAD1 | Outer Dynein Arm Docking Complex Subunit 1 | Protein Coding | Q96M63 | 44 | GC19M101235 |
| CXCL1 | C-X-C Motif Chemokine Ligand 1 | Protein Coding | P09341 | 56 | GC04P073869 |
| SQSTM1 | Sequestosome 1 | Protein Coding | Q13501 | 62 | GC05P179806 |
| MECP2 | Methyl-CpG Binding Protein 2 | Protein Coding | P51608 | 59 | GC0XM154021 |
| NCSTN | Nicastrin | Protein Coding | Q92542 | 61 | GC01P160343 |
| DEFB1 | Defensin Beta 1 | Protein Coding | P60022 | 50 | GC08M006870 |
| XYLT1 | Xylosyltransferase 1 | Protein Coding | Q86Y38 | 56 | GC16M017101 |
| LACC1 | Laccase Domain Containing 1 | Protein Coding | Q8IV20 | 47 | GC13P043879 |
| MLH1 | MutL Homolog 1 | Protein Coding | P40692 | 62 | GC03P036993 |
| DDR2 | Discoidin Domain Receptor Tyrosine Kinase 2 | Protein Coding | Q16832 | 63 | GC01P170843 |
| LGALS3 | Galectin 3 | Protein Coding | P17931 | 57 | GC14P055124 |
| CEACAM1 | CEA Cell Adhesion Molecule 1 | Protein Coding | P13688 | 56 | GC19M042507 |
| TSHR | Thyroid Stimulating Hormone Receptor | Protein Coding | P16473 | 61 | GC14P080954 |
| NTRK1 | Neurotrophic Receptor Tyrosine Kinase 1 | Protein Coding | P04629 | 62 | GC01P156815 |
| LILRB4 | Leukocyte Immunoglobulin Like Receptor B4 | Protein Coding | Q8NHJ6 | 50 | GC19P054643 |
| LMNA | Lamin A/C | Protein Coding | P02545 | 62 | GC01P156082 |
| BCR | BCR Activator Of RhoGEF And GTPase | Protein Coding | P11274 | 65 | GC22P023179 |
| FCN2 | Ficolin 2 | Protein Coding | Q15485 | 55 | GC09P134864 |
| LINC-ROR | Long Intergenic Non-Protein Coding RNA, Regulator Of Reprogramming | RNA Gene |  | 25 | GC18M057054 |
| HSP90AA1 | Heat Shock Protein 90 Alpha Family Class A Member 1 | Protein Coding | P07900 | 64 | GC14M102080 |
| TRIM25 | Tripartite Motif Containing 25 | Protein Coding | Q14258 | 57 | GC17M056836 |
| RHOH | Ras Homolog Family Member H | Protein Coding | Q15669 | 54 | GC04P040255 |
| CRIPT | CXXC Repeat Containing Interactor Of PDZ3 Domain | Protein Coding | Q9P021 | 51 | GC02P046616 |
| RSPH3 | Radial Spoke Head 3 | Protein Coding | Q86UC2 | 47 | GC06M158962 |
| PGR-AS1 | PGR Antisense RNA 1 | RNA Gene |  | 19 | GC11P101136 |
| ABCD4 | ATP Binding Cassette Subfamily D Member 4 | Protein Coding | O14678 | 54 | GC14M074285 |
| MICB | MHC Class I Polypeptide-Related Sequence B | Protein Coding | Q29980 | 52 | GC06P156239 |
| CD1D | CD1d Molecule | Protein Coding | P15813 | 55 | GC01P158178 |
| CFAP298 | Cilia And Flagella Associated Protein 298 | Protein Coding | P57076 | 50 | GC21M032593 |
| CCL20 | C-C Motif Chemokine Ligand 20 | Protein Coding | P78556 | 54 | GC02P227846 |
| MME | Membrane Metalloendopeptidase | Protein Coding | P08473 | 62 | GC03P155024 |
| CHEK2 | Checkpoint Kinase 2 | Protein Coding | O96017 | 67 | GC22M028687 |
| ANXA1 | Annexin A1 | Protein Coding | P04083 | 60 | GC09P073151 |
| SLAMF1 | Signaling Lymphocytic Activation Molecule Family Member 1 | Protein Coding | Q13291 | 53 | GC01M160608 |
| RNASEL | Ribonuclease L | Protein Coding | Q05823 | 56 | GC01M182573 |
| CLU | Clusterin | Protein Coding | P10909 | 60 | GC08M027596 |
| OXCT1 | 3-Oxoacid CoA-Transferase 1 | Protein Coding | P55809 | 58 | GC05M041732 |
| HMOX1 | Heme Oxygenase 1 | Protein Coding | P09601 | 65 | GC22P035380 |
| LRRC56 | Leucine Rich Repeat Containing 56 | Protein Coding | Q8IYG6 | 47 | GC11P014008 |
| PIGA | Phosphatidylinositol Glycan Anchor Biosynthesis Class A | Protein Coding | P37287 | 55 | GC0XM015319 |
| ABL1 | ABL Proto-Oncogene 1, Non-Receptor Tyrosine Kinase | Protein Coding | P00519 | 65 | GC09P130713 |
| CCR3 | C-C Motif Chemokine Receptor 3 | Protein Coding | P51677 | 57 | GC03P061350 |
| NFATC1 | Nuclear Factor Of Activated T Cells 1 | Protein Coding | O95644 | 61 | GC18P079395 |
| GBA1 | Glucosylceramidase Beta 1 | Protein Coding | P04062 | 62 | GC01M165117 |
| HYOU1 | Hypoxia Up-Regulated 1 | Protein Coding | Q9Y4L1 | 58 | GC11M133869 |
| ITGB1 | Integrin Subunit Beta 1 | Protein Coding | P05556 | 62 | GC10M036398 |
| POMC | Proopiomelanocortin | Protein Coding | P01189 | 61 | GC02M025160 |
| TGFBR2 | Transforming Growth Factor Beta Receptor 2 | Protein Coding | P37173 | 65 | GC03P030623 |
| COL11A1 | Collagen Type XI Alpha 1 Chain | Protein Coding | P12107 | 55 | GC01M102876 |
| DNMT1 | DNA Methyltransferase 1 | Protein Coding | P26358 | 65 | GC19M010133 |
| CTSA | Cathepsin A | Protein Coding | P10619 | 60 | GC20P045890 |
| PLP1 | Proteolipid Protein 1 | Protein Coding | P60201 | 55 | GC0XP103773 |
| BMP6 | Bone Morphogenetic Protein 6 | Protein Coding | P22004 | 56 | GC06P007726 |
| FCGRT | Fc Gamma Receptor And Transporter | Protein Coding | P55899 | 56 | GC19P049506 |
| CALR | Calreticulin | Protein Coding | P27797 | 64 | GC19P012938 |
| DNAAF6 | Dynein Axonemal Assembly Factor 6 | Protein Coding | Q9NQM4 | 40 | GC0XP107207 |
| SOS1 | SOS Ras/Rac Guanine Nucleotide Exchange Factor 1 | Protein Coding | Q07889 | 64 | GC02M039339 |
| MMP2 | Matrix Metallopeptidase 2 | Protein Coding | P08253 | 66 | GC16P105536 |
| NR1H4 | Nuclear Receptor Subfamily 1 Group H Member 4 | Protein Coding | Q96RI1 | 60 | GC12P100473 |
| FGFR3 | Fibroblast Growth Factor Receptor 3 | Protein Coding | P22607 | 68 | GC04P005899 |
| CD36 | CD36 Molecule (CD36 Blood Group) | Protein Coding | P16671 | 63 | GC07P080369 |
| BTLA | B And T Lymphocyte Associated | Protein Coding | Q7Z6A9 | 52 | GC03M112463 |
| HLA-F | Major Histocompatibility Complex, Class I, F | Protein Coding | P30511 | 51 | GC06P156190 |
| FCRL4 | Fc Receptor Like 4 | Protein Coding | Q96PJ5 | 47 | GC01M157573 |
| HBB | Hemoglobin Subunit Beta | Protein Coding | P68871 | 58 | GC11M013431 |
| CD226 | CD226 Molecule | Protein Coding | Q15762 | 54 | GC18M069831 |
| SPTAN1 | Spectrin Alpha, Non-Erythrocytic 1 | Protein Coding | Q13813 | 61 | GC09P128552 |
| PRKCQ | Protein Kinase C Theta | Protein Coding | Q04759 | 61 | GC10M007057 |
| NSD1 | Nuclear Receptor Binding SET Domain Protein 1 | Protein Coding | Q96L73 | 56 | GC05P188518 |
| TRB | T Cell Receptor Beta Locus | Protein Coding | P0DSE2 | 29 | GC07P163097 |
| MIRLET7B | MicroRNA Let-7b | RNA Gene |  | 29 | GC22P081742 |
| ITGA4 | Integrin Subunit Alpha 4 | Protein Coding | P13612 | 62 | GC02P181483 |
| PPP1R21 | Protein Phosphatase 1 Regulatory Subunit 21 | Protein Coding | Q6ZMI0 | 45 | GC02P048440 |
| ODAD4 | Outer Dynein Arm Docking Complex Subunit 4 | Protein Coding | Q96NG3 | 44 | GC17P133769 |
| CANT1 | Calcium Activated Nucleotidase 1 | Protein Coding | Q8WVQ1 | 56 | GC17M090491 |
| FCN1 | Ficolin 1 | Protein Coding | O00602 | 51 | GC09M135855 |
| CXCL11 | C-X-C Motif Chemokine Ligand 11 | Protein Coding | O14625 | 52 | GC04M076033 |
| NLRC5 | NLR Family CARD Domain Containing 5 | Protein Coding | Q86WI3 | 48 | GC16P105591 |
| CD47 | CD47 Molecule | Protein Coding | Q08722 | 58 | GC03M108043 |
| VAV1 | Vav Guanine Nucleotide Exchange Factor 1 | Protein Coding | P15498 | 58 | GC19P006772 |
| RB1 | RB Transcriptional Corepressor 1 | Protein Coding | P06400 | 61 | GC13P048303 |
| SDHA | Succinate Dehydrogenase Complex Flavoprotein Subunit A | Protein Coding | P31040 | 61 | GC05P000235 |
| MSH6 | MutS Homolog 6 | Protein Coding | P52701 | 61 | GC02P047695 |
| MIR132 | MicroRNA 132 | RNA Gene |  | 31 | GC17M002049 |
| IRGC | Immunity Related GTPase Cinema | Protein Coding | Q6NXR0 | 43 | GC19P043716 |
| CBLB | Cbl Proto-Oncogene B | Protein Coding | Q13191 | 58 | GC03M105655 |
| TNFRSF11A | TNF Receptor Superfamily Member 11a | Protein Coding | Q9Y6Q6 | 59 | GC18P062325 |
| PARP1 | Poly(ADP-Ribose) Polymerase 1 | Protein Coding | P09874 | 63 | GC01M226360 |
| ENG | Endoglin | Protein Coding | P17813 | 60 | GC09M130980 |
| HNF1A | HNF1 Homeobox A | Protein Coding | P20823 | 59 | GC12P120978 |
| KITLG | KIT Ligand | Protein Coding | P21583 | 60 | GC12M088492 |
| PVT1 | Pvt1 Oncogene | RNA Gene |  | 34 | GC08P128246 |
| COL11A2 | Collagen Type XI Alpha 2 Chain | Protein Coding | P13942 | 55 | GC06M033162 |
| DYM | Dymeclin | Protein Coding | Q7RTS9 | 51 | GC18M049041 |
| CLEC4E | C-Type Lectin Domain Family 4 Member E | Protein Coding | Q9ULY5 | 47 | GC12M032984 |
| CHST3 | Carbohydrate Sulfotransferase 3 | Protein Coding | Q7LGC8 | 55 | GC10P071964 |
| S100A8 | S100 Calcium Binding Protein A8 | Protein Coding | P05109 | 54 | GC01M165010 |
| LIF | LIF Interleukin 6 Family Cytokine | Protein Coding | P15018 | 56 | GC22M030240 |
| PCYT1A | Phosphate Cytidylyltransferase 1A, Choline | Protein Coding | P49585 | 59 | GC03M196214 |
| KIF22 | Kinesin Family Member 22 | Protein Coding | Q14807 | 56 | GC16P104905 |
| SMAD3 | SMAD Family Member 3 | Protein Coding | P84022 | 66 | GC15P067063 |
| MMP9 | Matrix Metallopeptidase 9 | Protein Coding | P14780 | 66 | GC20P046008 |
| XRCC5 | X-Ray Repair Cross Complementing 5 | Protein Coding | P13010 | 56 | GC02P216107 |
| IRGQ | Immunity Related GTPase Q | Protein Coding | Q8WZA9 | 43 | GC19M043584 |
| LGALS9 | Galectin 9 | Protein Coding | O00182 | 51 | GC17P027629 |
| HDAC4 | Histone Deacetylase 4 | Protein Coding | P56524 | 65 | GC02M239048 |
| IL16 | Interleukin 16 | Protein Coding | Q14005 | 54 | GC15P081159 |
| MIR150 | MicroRNA 150 | RNA Gene |  | 30 | GC19M049500 |
| COMT | Catechol-O-Methyltransferase | Protein Coding | P21964 | 63 | GC22P019941 |
| IGH | Immunoglobulin Heavy Locus | Protein Coding |  | 24 | GC14M122251 |
| PAPSS2 | 3'-Phosphoadenosine 5'-Phosphosulfate Synthase 2 | Protein Coding | O95340 | 54 | GC10P087659 |
| ITPR1 | Inositol 1,4,5-Trisphosphate Receptor Type 1 | Protein Coding | Q14643 | 62 | GC03P004486 |
| XRCC6 | X-Ray Repair Cross Complementing 6 | Protein Coding | P12956 | 58 | GC22P081632 |
| ERAP1 | Endoplasmic Reticulum Aminopeptidase 1 | Protein Coding | Q9NZ08 | 57 | GC05M096760 |
| GINS1 | GINS Complex Subunit 1 | Protein Coding | Q14691 | 51 | GC20P026429 |
| PRDM1 | PR/SET Domain 1 | Protein Coding | O75626 | 58 | GC06P157373 |
| VCP | Valosin Containing Protein | Protein Coding | P55072 | 62 | GC09M035431 |
| MIR221 | MicroRNA 221 | RNA Gene |  | 30 | GC0XM045746 |
| PTCH1 | Patched 1 | Protein Coding | Q13635 | 63 | GC09M095442 |
| AIM2 | Absent In Melanoma 2 | Protein Coding | O14862 | 52 | GC01M165270 |
| CD1A | CD1a Molecule | Protein Coding | P06126 | 51 | GC01P170661 |
| PADI4 | Peptidyl Arginine Deiminase 4 | Protein Coding | Q9UM07 | 58 | GC01P017308 |
| AKT3 | AKT Serine/Threonine Kinase 3 | Protein Coding | Q9Y243 | 68 | GC01M243488 |
| SOD2-OT1 | SOD2 Overlapping Transcript 1 | RNA Gene |  | 20 | GC06M159772 |
| ERCC3 | ERCC Excision Repair 3, TFIIH Core Complex Helicase Subunit | Protein Coding | P19447 | 60 | GC02M127257 |
| SLK | STE20 Like Kinase | Protein Coding | Q9H2G2 | 54 | GC10P103967 |
| TSC1 | TSC Complex Subunit 1 | Protein Coding | Q92574 | 61 | GC09M132891 |
| LIG1 | DNA Ligase 1 | Protein Coding | P18858 | 60 | GC19M048115 |
| SFTA3 | Surfactant Associated 3 | RNA Gene | P0C7M3 | 43 | GC14M036518 |
| CBS | Cystathionine Beta-Synthase | Protein Coding | P35520 | 63 | GC21M043053 |
| FGF2 | Fibroblast Growth Factor 2 | Protein Coding | P09038 | 58 | GC04P122826 |
| TGFBR1 | Transforming Growth Factor Beta Receptor 1 | Protein Coding | P36897 | 66 | GC09P103929 |
| RHD | Rh Blood Group D Antigen | Protein Coding | Q02161 | 53 | GC01P025272 |
| POP1 | POP1 Homolog, Ribonuclease P/MRP Subunit | Protein Coding | Q99575 | 50 | GC08P098117 |
| FGFR1 | Fibroblast Growth Factor Receptor 1 | Protein Coding | P11362 | 68 | GC08M038400 |
| MIR29A | MicroRNA 29a | RNA Gene |  | 30 | GC07M130876 |
| TRAPPC2 | Trafficking Protein Particle Complex Subunit 2 | Protein Coding | P0DI81 | 51 | GC0XM013712 |
| MIR122 | MicroRNA 122 | RNA Gene |  | 30 | GC18P058451 |
| MUC1 | Mucin 1, Cell Surface Associated | Protein Coding | P15941 | 61 | GC01M155185 |
| CEBPB | CCAAT Enhancer Binding Protein Beta | Protein Coding | P17676 | 57 | GC20P050190 |
| CXCL2 | C-X-C Motif Chemokine Ligand 2 | Protein Coding | P19875 | 52 | GC04M074097 |
| GIMAP6 | GTPase, IMAP Family Member 6 | Protein Coding | Q6P9H5 | 44 | GC07M150625 |
| MATN3 | Matrilin 3 | Protein Coding | O15232 | 54 | GC02M019992 |
| COL3A1 | Collagen Type III Alpha 1 Chain | Protein Coding | P02461 | 61 | GC02P188974 |
| PYCARD | PYD And CARD Domain Containing | Protein Coding | Q9ULZ3 | 54 | GC16M031201 |
| CCL18 | C-C Motif Chemokine Ligand 18 | Protein Coding | P55774 | 48 | GC17P036064 |
| SLAMF7 | SLAM Family Member 7 | Protein Coding | Q9NQ25 | 54 | GC01P170748 |
| VEGFA | Vascular Endothelial Growth Factor A | Protein Coding | P15692 | 61 | GC06P043770 |
| GJA1 | Gap Junction Protein Alpha 1 | Protein Coding | P17302 | 63 | GC06P157880 |
| CD160 | CD160 Molecule | Protein Coding | O95971 | 52 | GC01P145719 |
| TRA | T Cell Receptor Alpha Locus | Protein Coding | P0DSE1 | 30 | GC14P021621 |
| TNFRSF18 | TNF Receptor Superfamily Member 18 | Protein Coding | Q9Y5U5 | 55 | GC01M001203 |
| IFNA6 | Interferon Alpha 6 | Protein Coding | P05013 | 48 | GC09M022230 |
| IFNA8 | Interferon Alpha 8 | Protein Coding | P32881 | 48 | GC09P021409 |
| IFNA13 | Interferon Alpha 13 | Protein Coding | P01562 | 44 | GC09M021367 |
| MIR20A | MicroRNA 20a | RNA Gene |  | 29 | GC13P091717 |
| CLEC12A | C-Type Lectin Domain Family 12 Member A | Protein Coding | Q5QGZ9 | 50 | GC12P009951 |
| TNFRSF8 | TNF Receptor Superfamily Member 8 | Protein Coding | P28908 | 56 | GC01P012063 |
| CLCN7 | Chloride Voltage-Gated Channel 7 | Protein Coding | P51798 | 57 | GC16M001444 |
| CD74 | CD74 Molecule | Protein Coding | P04233 | 57 | GC05M150378 |
| PIK3R2 | Phosphoinositide-3-Kinase Regulatory Subunit 2 | Protein Coding | O00459 | 62 | GC19P018153 |
| THSD1 | Thrombospondin Type 1 Domain Containing 1 | Protein Coding | Q9NS62 | 51 | GC13M052377 |
| MIR210 | MicroRNA 210 | RNA Gene |  | 30 | GC11M013222 |
| ESR1 | Estrogen Receptor 1 | Protein Coding | P03372 | 66 | GC06P151656 |
| IFNA14 | Interferon Alpha 14 | Protein Coding | P01570 | 48 | GC09M021239 |
| DPP4 | Dipeptidyl Peptidase 4 | Protein Coding | P27487 | 62 | GC02M161992 |
| NKX3-2 | NK3 Homeobox 2 | Protein Coding | P78367 | 48 | GC04M013542 |
| RYR1 | Ryanodine Receptor 1 | Protein Coding | P21817 | 61 | GC19P132993 |
| RHOA | Ras Homolog Family Member A | Protein Coding | P61586 | 62 | GC03M049359 |
| TNIP1 | TNFAIP3 Interacting Protein 1 | Protein Coding | Q15025 | 51 | GC05M151029 |
| CXCL13 | C-X-C Motif Chemokine Ligand 13 | Protein Coding | O43927 | 52 | GC04P077511 |
| BIRC2 | Baculoviral IAP Repeat Containing 2 | Protein Coding | Q13490 | 60 | GC11P102347 |
| ATP7A | ATPase Copper Transporting Alpha | Protein Coding | Q04656 | 58 | GC0XP078214 |
| TTR | Transthyretin | Protein Coding | P02766 | 61 | GC18P031557 |
| FCRL3 | Fc Receptor Like 3 | Protein Coding | Q96P31 | 49 | GC01M157674 |
| GRB2 | Growth Factor Receptor Bound Protein 2 | Protein Coding | P62993 | 59 | GC17M075318 |
| SH3BP2 | SH3 Domain Binding Protein 2 | Protein Coding | P78314 | 52 | GC04P005964 |
| CDKN1A | Cyclin Dependent Kinase Inhibitor 1A | Protein Coding | P38936 | 63 | GC06P156447 |
| IL19 | Interleukin 19 | Protein Coding | Q9UHD0 | 52 | GC01P206770 |
| SDHC | Succinate Dehydrogenase Complex Subunit C | Protein Coding | Q99643 | 55 | GC01P161314 |
| IFNA5 | Interferon Alpha 5 | Protein Coding | P01569 | 51 | GC09M021304 |
| HLA-DMA | Major Histocompatibility Complex, Class II, DM Alpha | Protein Coding | P28067 | 51 | GC06M100377 |
| TNFSF10 | TNF Superfamily Member 10 | Protein Coding | P50591 | 57 | GC03M172505 |
| UBE2L3 | Ubiquitin Conjugating Enzyme E2 L3 | Protein Coding | P68036 | 58 | GC22P021549 |
| FOXO1 | Forkhead Box O1 | Protein Coding | Q12778 | 64 | GC13M040555 |
| HSPB1 | Heat Shock Protein Family B (Small) Member 1 | Protein Coding | P04792 | 64 | GC07P076302 |
| IL18BP | Interleukin 18 Binding Protein | Protein Coding | O95998 | 52 | GC11P071998 |
| GIMAP5 | GTPase, IMAP Family Member 5 | Protein Coding | Q96F15 | 49 | GC07P150722 |
| FLT3LG | Fms Related Receptor Tyrosine Kinase 3 Ligand | Protein Coding | P49771 | 53 | GC19P133413 |
| MEG3 | Maternally Expressed 3 | RNA Gene |  | 36 | GC14P119042 |
| FN1 | Fibronectin 1 | Protein Coding | P02751 | 63 | GC02M215360 |
| ALOX15 | Arachidonate 15-Lipoxygenase | Protein Coding | P16050 | 58 | GC17M004630 |
| TCF4 | Transcription Factor 4 | Protein Coding | P15884 | 59 | GC18M055222 |
| LOC126805749 | BRD4-Independent Group 4 Enhancer GRCh37_chr1:65312286-65313485 | Functional Element |  | 10 | GC01P064846 |
| SLAMF6 | SLAM Family Member 6 | Protein Coding | Q96DU3 | 50 | GC01M165314 |
| HEXB | Hexosaminidase Subunit Beta | Protein Coding | P07686 | 60 | GC05P074640 |
| ATG5 | Autophagy Related 5 | Protein Coding | Q9H1Y0 | 58 | GC06M106045 |
| DCLRE1B | DNA Cross-Link Repair 1B | Protein Coding | Q9H816 | 48 | GC01P113905 |
| PTK2B | Protein Tyrosine Kinase 2 Beta | Protein Coding | Q14289 | 62 | GC08P027311 |
| CD33 | CD33 Molecule | Protein Coding | P20138 | 58 | GC19P133505 |
| VHL | Von Hippel-Lindau Tumor Suppressor | Protein Coding | P40337 | 60 | GC03P024217 |
| PDCD1LG2 | Programmed Cell Death 1 Ligand 2 | Protein Coding | Q9BQ51 | 55 | GC09P005510 |
| SERPINE1 | Serpin Family E Member 1 | Protein Coding | P05121 | 63 | GC07P101127 |
| CD5 | CD5 Molecule | Protein Coding | P06127 | 55 | GC11P098588 |
| GSN | Gelsolin | Protein Coding | P06396 | 62 | GC09P121201 |
| CLEC4A | C-Type Lectin Domain Family 4 Member A | Protein Coding | Q9UMR7 | 48 | GC12P046819 |
| IL32 | Interleukin 32 | Protein Coding | P24001 | 50 | GC16P104084 |
| CCN6 | Cellular Communication Network Factor 6 | Protein Coding | O95389 | 52 | GC06P157639 |
| PSENEN | Presenilin Enhancer, Gamma-Secretase Subunit | Protein Coding | Q9NZ42 | 56 | GC19P132929 |
| FPR1 | Formyl Peptide Receptor 1 | Protein Coding | P21462 | 58 | GC19M051745 |
| OCA2 | OCA2 Melanosomal Transmembrane Protein | Protein Coding | Q04671 | 56 | GC15M028289 |
| LTBR | Lymphotoxin Beta Receptor | Protein Coding | P36941 | 54 | GC12P006375 |
| EPHB4 | EPH Receptor B4 | Protein Coding | P54760 | 66 | GC07M106119 |
| IL25 | Interleukin 25 | Protein Coding | Q9H293 | 50 | GC14P051703 |
| DDX3X | DEAD-Box Helicase 3 X-Linked | Protein Coding | O00571 | 62 | GC0XP041333 |
| TNFRSF17 | TNF Receptor Superfamily Member 17 | Protein Coding | Q02223 | 60 | GC16P011965 |
| TNFRSF11B | TNF Receptor Superfamily Member 11b | Protein Coding | O00300 | 59 | GC08M118923 |
| BAX | BCL2 Associated X, Apoptosis Regulator | Protein Coding | Q07812 | 64 | GC19P048954 |
| CYP24A1 | Cytochrome P450 Family 24 Subfamily A Member 1 | Protein Coding | Q07973 | 59 | GC20M054153 |
| SELP | Selectin P | Protein Coding | P16109 | 58 | GC01M169558 |
| CRKL | CRK Like Proto-Oncogene, Adaptor Protein | Protein Coding | P46109 | 59 | GC22P020917 |
| MEN1 | Menin 1 | Protein Coding | O00255 | 58 | GC11M064803 |
| F3 | Coagulation Factor III, Tissue Factor | Protein Coding | P13726 | 58 | GC01M094956 |
| CEACAM3 | CEA Cell Adhesion Molecule 3 | Protein Coding | P40198 | 54 | GC19P041796 |
| BST2 | Bone Marrow Stromal Cell Antigen 2 | Protein Coding | Q10589 | 52 | GC19M100564 |
| MMP1 | Matrix Metallopeptidase 1 | Protein Coding | P03956 | 62 | GC11M133503 |
| CLEC6A | C-Type Lectin Domain Containing 6A | Protein Coding | Q6EIG7 | 48 | GC12P008455 |
| CSK | C-Terminal Src Kinase | Protein Coding | P41240 | 59 | GC15P074782 |
| BPNT2 | 3'(2'), 5'-Bisphosphate Nucleotidase 2 | Protein Coding | Q9NX62 | 53 | GC08M056958 |
| SLC39A13 | Solute Carrier Family 39 Member 13 | Protein Coding | Q96H72 | 51 | GC11P047407 |
| CCL22 | C-C Motif Chemokine Ligand 22 | Protein Coding | O00626 | 49 | GC16P105604 |
| HLA-DRB4 | Major Histocompatibility Complex, Class II, DR Beta 4 | Protein Coding | P13762 | 39 | GC06Mo03851 |
| INSR | Insulin Receptor | Protein Coding | P06213 | 66 | GC19M007112 |
| SNCA | Synuclein Alpha | Protein Coding | P37840 | 65 | GC04M089724 |
| SETX | Senataxin | Protein Coding | Q7Z333 | 54 | GC09M132261 |
| APP | Amyloid Beta Precursor Protein | Protein Coding | P05067 | 63 | GC21M025880 |
| FCER1A | Fc Epsilon Receptor Ia | Protein Coding | P12319 | 54 | GC01P159283 |
| RAB33B | RAB33B, Member RAS Oncogene Family | Protein Coding | Q9H082 | 51 | GC04P139439 |
| VTRNA1-1 | Vault RNA 1-1 | RNA Gene |  | 18 | GC05P156893 |
| CD96 | CD96 Molecule | Protein Coding | P40200 | 55 | GC03P111292 |
| GNRH1 | Gonadotropin Releasing Hormone 1 | Protein Coding | P01148 | 52 | GC08M025419 |
| WWOX | WW Domain Containing Oxidoreductase | Protein Coding | Q9NZC7 | 59 | GC16P078099 |
| BCL3 | BCL3 Transcription Coactivator | Protein Coding | P20749 | 54 | GC19P044747 |
| EGF | Epidermal Growth Factor | Protein Coding | P01133 | 64 | GC04P109912 |
| CNR2 | Cannabinoid Receptor 2 | Protein Coding | P34972 | 57 | GC01M023870 |
| PLG | Plasminogen | Protein Coding | P00747 | 61 | GC06P160702 |
| ERCC1 | ERCC Excision Repair 1, Endonuclease Non-Catalytic Subunit | Protein Coding | P07992 | 59 | GC19M101124 |
| GAPDH | Glyceraldehyde-3-Phosphate Dehydrogenase | Protein Coding | P04406 | 63 | GC12P046751 |
| CTSG | Cathepsin G | Protein Coding | P08311 | 56 | GC14M024573 |
| SETD2 | SET Domain Containing 2, Histone Lysine Methyltransferase | Protein Coding | Q9BYW2 | 59 | GC03M047033 |
| MIR214 | MicroRNA 214 | RNA Gene |  | 31 | GC01M172234 |
| IL11 | Interleukin 11 | Protein Coding | P20809 | 52 | GC19M055364 |
| TNFSF13 | TNF Superfamily Member 13 | Protein Coding | O75888 | 56 | GC17P007558 |
| TIGIT | T Cell Immunoreceptor With Ig And ITIM Domains | Protein Coding | Q495A1 | 52 | GC03P114276 |
| C3AR1 | Complement C3a Receptor 1 | Protein Coding | Q16581 | 56 | GC12M032957 |
| AIP | Aryl Hydrocarbon Receptor Interacting Protein | Protein Coding | O00170 | 55 | GC11P067468 |
| SIRPA | Signal Regulatory Protein Alpha | Protein Coding | P78324 | 58 | GC20P001894 |
| GCK | Glucokinase | Protein Coding | P35557 | 62 | GC07M045514 |
| IL1RL1 | Interleukin 1 Receptor Like 1 | Protein Coding | Q01638 | 54 | GC02P102294 |
| GZMA | Granzyme A | Protein Coding | P12544 | 55 | GC05P055102 |
| GLB1 | Galactosidase Beta 1 | Protein Coding | P16278 | 62 | GC03M032963 |
| ANKZF1 | Ankyrin Repeat And Zinc Finger Peptidyl TRNA Hydrolase 1 | Protein Coding | Q9H8Y5 | 48 | GC02P219229 |
| CYBC1 | Cytochrome B-245 Chaperone 1 | Protein Coding | Q9BQA9 | 47 | GC17M090663 |
| MAF | MAF BZIP Transcription Factor | Protein Coding | O75444 | 58 | GC16M079204 |
| HK1 | Hexokinase 1 | Protein Coding | P19367 | 63 | GC10P069269 |
| TRIM8 | Tripartite Motif Containing 8 | Protein Coding | Q9BZR9 | 50 | GC10P116560 |
| IL18RAP | Interleukin 18 Receptor Accessory Protein | Protein Coding | O95256 | 50 | GC02P102418 |
| TNFSF9 | TNF Superfamily Member 9 | Protein Coding | P41273 | 50 | GC19P006531 |
| SNAP29 | Synaptosome Associated Protein 29 | Protein Coding | O95721 | 54 | GC22P020859 |
| CFHR1 | Complement Factor H Related 1 | Protein Coding | Q03591 | 54 | GC01P196822 |
| TEC | Tec Protein Tyrosine Kinase | Protein Coding | P42680 | 58 | GC04M048332 |
| MIRLET7C | MicroRNA Let-7c | RNA Gene |  | 31 | GC21P019897 |
| MDM2 | MDM2 Proto-Oncogene | Protein Coding | Q00987 | 66 | GC12P068808 |
| CD22 | CD22 Molecule | Protein Coding | P20273 | 59 | GC19P035319 |
| MIR16-1 | MicroRNA 16-1 | RNA Gene |  | 29 | GC13M050048 |
| GALC | Galactosylceramidase | Protein Coding | P54803 | 57 | GC14M087837 |
| ITPR3 | Inositol 1,4,5-Trisphosphate Receptor Type 3 | Protein Coding | Q14573 | 58 | GC06P033620 |
| MRTFA | Myocardin Related Transcription Factor A | Protein Coding | Q969V6 | 52 | GC22M082416 |
| C11orf65 | Chromosome 11 Open Reading Frame 65 | Protein Coding | Q8NCR3 | 42 | GC11M108308 |
| MIR19A | MicroRNA 19a | RNA Gene |  | 29 | GC13P091713 |
| F8 | Coagulation Factor VIII | Protein Coding | P00451 | 59 | GC0XM154835 |
| CCR4 | C-C Motif Chemokine Receptor 4 | Protein Coding | P51679 | 57 | GC03P032951 |
| IFNA17 | Interferon Alpha 17 | Protein Coding | P01571 | 44 | GC09M021227 |
| SOX2 | SRY-Box Transcription Factor 2 | Protein Coding | P48431 | 58 | GC03P181711 |
| CLEC4M | C-Type Lectin Domain Family 4 Member M | Protein Coding | Q9H2X3 | 52 | GC19P007763 |
| SEMA4D | Semaphorin 4D | Protein Coding | Q92854 | 58 | GC09M089360 |
| DNASE1 | Deoxyribonuclease 1 | Protein Coding | P24855 | 56 | GC16P003611 |
| LOC107303343 | Adenosine Deaminase Intronic Regulatory Elements | Functional Element |  | 8 | GC20P044629 |
| FMR1 | Fragile X Messenger Ribonucleoprotein 1 | Protein Coding | Q06787 | 56 | GC0XP148000 |
| TRAF5 | TNF Receptor Associated Factor 5 | Protein Coding | O00463 | 54 | GC01P211326 |
| KLRC2 | Killer Cell Lectin Like Receptor C2 | Protein Coding | P26717 | 46 | GC12M033117 |
| POLE2 | DNA Polymerase Epsilon 2, Accessory Subunit | Protein Coding | P56282 | 54 | GC14M049643 |
| RBPJ | Recombination Signal Binding Protein For Immunoglobulin Kappa J Region | Protein Coding | Q06330 | 59 | GC04P026105 |
| ACOD1 | Aconitate Decarboxylase 1 | Protein Coding | A6NK06 | 38 | GC13P076949 |
| MARCHF8 | Membrane Associated Ring-CH-Type Finger 8 | Protein Coding | Q5T0T0 | 45 | GC10M048783 |
| PAK1 | P21 (RAC1) Activated Kinase 1 | Protein Coding | Q13153 | 63 | GC11M133198 |
| ITGAX | Integrin Subunit Alpha X | Protein Coding | P20702 | 59 | GC16P105024 |
| ELF4 | E74 Like ETS Transcription Factor 4 | Protein Coding | Q99607 | 51 | GC0XM130064 |
| PECAM1 | Platelet And Endothelial Cell Adhesion Molecule 1 | Protein Coding | P16284 | 53 | GC17M064319 |
| PSMB4 | Proteasome 20S Subunit Beta 4 | Protein Coding | P28070 | 58 | GC01P151399 |
| SDHB | Succinate Dehydrogenase Complex Iron Sulfur Subunit B | Protein Coding | P21912 | 61 | GC01M020330 |
| TNFSF15 | TNF Superfamily Member 15 | Protein Coding | O95150 | 55 | GC09M114784 |
| S100A12 | S100 Calcium Binding Protein A12 | Protein Coding | P80511 | 50 | GC01M153373 |
| SELE | Selectin E | Protein Coding | P16581 | 56 | GC01M169722 |
| LAMP2 | Lysosomal Associated Membrane Protein 2 | Protein Coding | P13473 | 57 | GC0XM120426 |
| ATRIP | ATR Interacting Protein | Protein Coding | Q8WXE1 | 55 | GC03P061480 |
| MIR106B | MicroRNA 106b | RNA Gene |  | 30 | GC07M106065 |
| GSK3B | Glycogen Synthase Kinase 3 Beta | Protein Coding | P49841 | 63 | GC03M119821 |
| MOG | Myelin Oligodendrocyte Glycoprotein | Protein Coding | Q16653 | 58 | GC06P156186 |
| DHX9 | DExH-Box Helicase 9 | Protein Coding | Q08211 | 56 | GC01P182839 |
| KDM6A | Lysine Demethylase 6A | Protein Coding | O15550 | 59 | GC0XP044873 |
| ERCC5 | ERCC Excision Repair 5, Endonuclease | Protein Coding | P28715 | 58 | GC13P102845 |
| CDC73 | Cell Division Cycle 73 | Protein Coding | Q6P1J9 | 58 | GC01P193121 |
| RAPSN | Receptor Associated Protein Of The Synapse | Protein Coding | Q13702 | 54 | GC11M132471 |
| FLG | Filaggrin | Protein Coding | P20930 | 54 | GC01M164987 |
| CCL19 | C-C Motif Chemokine Ligand 19 | Protein Coding | Q99731 | 52 | GC09M035507 |
| TOLLIP | Toll Interacting Protein | Protein Coding | Q9H0E2 | 56 | GC11M001274 |
| RNASE3 | Ribonuclease A Family Member 3 | Protein Coding | P12724 | 52 | GC14P020891 |
| TANK | TRAF Family Member Associated NFKB Activator | Protein Coding | Q92844 | 55 | GC02P161136 |
| IL17C | Interleukin 17C | Protein Coding | Q9P0M4 | 47 | GC16P088638 |
| HSALR1 | HSP90AB1 Associated LncRNA 1 | RNA Gene |  | 17 | GC16P106973 |
| APOH | Apolipoprotein H | Protein Coding | P02749 | 58 | GC17M066212 |
| CCRL2 | C-C Motif Chemokine Receptor Like 2 | Protein Coding | O00421 | 50 | GC03P046407 |
| ADAMTS13 | ADAM Metallopeptidase With Thrombospondin Type 1 Motif 13 | Protein Coding | Q76LX8 | 61 | GC09P133414 |
| VIP | Vasoactive Intestinal Peptide | Protein Coding | P01282 | 56 | GC06P152750 |
| DHCR24 | 24-Dehydrocholesterol Reductase | Protein Coding | Q15392 | 57 | GC01M054849 |
| POLG | DNA Polymerase Gamma, Catalytic Subunit | Protein Coding | P54098 | 60 | GC15M156028 |
| MIR126 | MicroRNA 126 | RNA Gene |  | 31 | GC09P136670 |
| CD163 | CD163 Molecule | Protein Coding | Q86VB7 | 57 | GC12M032935 |
| RSAD2 | Radical S-Adenosyl Methionine Domain Containing 2 | Protein Coding | Q8WXG1 | 52 | GC02P006865 |
| C5AR2 | Complement C5a Receptor 2 | Protein Coding | Q9P296 | 50 | GC19P133321 |
| MAP2K2 | Mitogen-Activated Protein Kinase Kinase 2 | Protein Coding | P36507 | 67 | GC19M004090 |
| C9 | Complement C9 | Protein Coding | P02748 | 56 | GC05M039320 |
| POLD1 | DNA Polymerase Delta 1, Catalytic Subunit | Protein Coding | P28340 | 59 | GC19P133473 |
| BRD4 | Bromodomain Containing 4 | Protein Coding | O60885 | 59 | GC19M100458 |
| FCER1G | Fc Epsilon Receptor Ig | Protein Coding | P30273 | 52 | GC01P161215 |
| CASR | Calcium Sensing Receptor | Protein Coding | P41180 | 63 | GC03P122183 |
| NOS1 | Nitric Oxide Synthase 1 | Protein Coding | P29475 | 62 | GC12M117208 |
| MMP3 | Matrix Metallopeptidase 3 | Protein Coding | P08254 | 62 | GC11M102835 |
| CXCR5 | C-X-C Motif Chemokine Receptor 5 | Protein Coding | P32302 | 54 | GC11P123529 |
| MIRLET7E | MicroRNA Let-7e | RNA Gene |  | 29 | GC19P133538 |
| SOX10 | SRY-Box Transcription Factor 10 | Protein Coding | P56693 | 57 | GC22M083306 |
| KDR | Kinase Insert Domain Receptor | Protein Coding | P35968 | 66 | GC04M055078 |
| FLT4 | Fms Related Receptor Tyrosine Kinase 4 | Protein Coding | P35916 | 65 | GC05M182771 |
| CD58 | CD58 Molecule | Protein Coding | P19256 | 54 | GC01M116514 |
| MIR149 | MicroRNA 149 | RNA Gene |  | 31 | GC02P240456 |
| OPTN | Optineurin | Protein Coding | Q96CV9 | 56 | GC10P013099 |
| APTX | Aprataxin | Protein Coding | Q7Z2E3 | 56 | GC09M032886 |
| BECN1 | Beclin 1 | Protein Coding | Q14457 | 59 | GC17M042810 |
| PIM1 | Pim-1 Proto-Oncogene, Serine/Threonine Kinase | Protein Coding | P11309 | 62 | GC06P156456 |
| PIK3R5 | Phosphoinositide-3-Kinase Regulatory Subunit 5 | Protein Coding | Q8WYR1 | 56 | GC17M008878 |
| HSP90B1 | Heat Shock Protein 90 Beta Family Member 1 | Protein Coding | P14625 | 60 | GC12P103930 |
| OFD1 | OFD1 Centriole And Centriolar Satellite Protein | Protein Coding | O75665 | 54 | GC0XP013714 |
| HDAC1 | Histone Deacetylase 1 | Protein Coding | Q13547 | 63 | GC01P032292 |
| NPM1 | Nucleophosmin 1 | Protein Coding | P06748 | 63 | GC05P171387 |
| HLA-DQA2 | Major Histocompatibility Complex, Class II, DQ Alpha 2 | Protein Coding | P01906 | 50 | GC06P032741 |
| TYR | Tyrosinase | Protein Coding | P14679 | 61 | GC11P089177 |
| PF4 | Platelet Factor 4 | Protein Coding | P02776 | 52 | GC04M073980 |
| THPO | Thrombopoietin | Protein Coding | P40225 | 56 | GC03M184371 |
| SYNE1 | Spectrin Repeat Containing Nuclear Envelope Protein 1 | Protein Coding | Q8NF91 | 52 | GC06M152121 |
| APOBEC3G | Apolipoprotein B MRNA Editing Enzyme Catalytic Subunit 3G | Protein Coding | Q9HC16 | 52 | GC22P081519 |
| SASH3 | SAM And SH3 Domain Containing 3 | Protein Coding | O75995 | 43 | GC0XP129781 |
| CCL7 | C-C Motif Chemokine Ligand 7 | Protein Coding | P80098 | 55 | GC17P034270 |
| MIR15A | MicroRNA 15a | RNA Gene |  | 25 | GC13M050049 |
| CD34 | CD34 Molecule | Protein Coding | P28906 | 58 | GC01M207880 |
| KLRB1 | Killer Cell Lectin Like Receptor B1 | Protein Coding | Q12918 | 51 | GC12M033104 |
| CD84 | CD84 Molecule | Protein Coding | Q9UIB8 | 53 | GC01M160541 |
| ACKR2 | Atypical Chemokine Receptor 2 | Protein Coding | O00590 | 51 | GC03P042804 |
| IFNA21 | Interferon Alpha 21 | Protein Coding | P01568 | 47 | GC09M021165 |
| IL15RA | Interleukin 15 Receptor Subunit Alpha | Protein Coding | Q13261 | 51 | GC10M005943 |
| DBR1 | Debranching RNA Lariats 1 | Protein Coding | Q9UK59 | 48 | GC03M138160 |
| MAPK11 | Mitogen-Activated Protein Kinase 11 | Protein Coding | Q15759 | 60 | GC22M050263 |
| TRA-TGC7-1 | TRNA-Ala (Anticodon TGC) 7-1 | RNA Gene |  | 16 | GC06M100125 |
| BAP1 | BRCA1 Associated Deubiquitinase 1 | Protein Coding | Q92560 | 59 | GC03M052401 |
| IFNA4 | Interferon Alpha 4 | Protein Coding | P05014 | 46 | GC09M021186 |
| IFNA7 | Interferon Alpha 7 | Protein Coding | P01567 | 44 | GC09M022226 |
| COL1A1 | Collagen Type I Alpha 1 Chain | Protein Coding | P02452 | 62 | GC17M089744 |
| APOA1 | Apolipoprotein A1 | Protein Coding | P02647 | 63 | GC11M116835 |
| CD93 | CD93 Molecule | Protein Coding | Q9NPY3 | 52 | GC20M023079 |
| ABCA1 | ATP Binding Cassette Subfamily A Member 1 | Protein Coding | O95477 | 61 | GC09M104781 |
| MAPK10 | Mitogen-Activated Protein Kinase 10 | Protein Coding | P53779 | 60 | GC04M085990 |
| PTH | Parathyroid Hormone | Protein Coding | P01270 | 58 | GC11M013492 |
| CD38 | CD38 Molecule | Protein Coding | P28907 | 59 | GC04P026843 |
| SOD2 | Superoxide Dismutase 2 | Protein Coding | P04179 | 61 | GC06M159669 |
| VEGFC | Vascular Endothelial Growth Factor C | Protein Coding | P49767 | 59 | GC04M176683 |
| PSMA7 | Proteasome 20S Subunit Alpha 7 | Protein Coding | O14818 | 57 | GC20M062136 |
| GNLY | Granulysin | Protein Coding | P22749 | 49 | GC02P085685 |
| HPRT1 | Hypoxanthine Phosphoribosyltransferase 1 | Protein Coding | P00492 | 61 | GC0XP134460 |
| ADIPOQ | Adiponectin, C1Q And Collagen Domain Containing | Protein Coding | Q15848 | 59 | GC03P186842 |
| WRN | WRN RecQ Like Helicase | Protein Coding | Q14191 | 60 | GC08P031033 |
| VTN | Vitronectin | Protein Coding | P04004 | 56 | GC17M088839 |
| MS4A2 | Membrane Spanning 4-Domains A2 | Protein Coding | Q01362 | 50 | GC11P060088 |
| IFNA16 | Interferon Alpha 16 | Protein Coding | P05015 | 43 | GC09M021216 |
| CHRNA1 | Cholinergic Receptor Nicotinic Alpha 1 Subunit | Protein Coding | P02708 | 58 | GC02M174747 |
| MIR24-1 | MicroRNA 24-1 | RNA Gene |  | 27 | GC09P095086 |
| C1QBP | Complement C1q Binding Protein | Protein Coding | Q07021 | 58 | GC17M005432 |
| MIR30A | MicroRNA 30a | RNA Gene |  | 28 | GC06M071403 |
| CCL21 | C-C Motif Chemokine Ligand 21 | Protein Coding | O00585 | 54 | GC09M034709 |
| CXCL5 | C-X-C Motif Chemokine Ligand 5 | Protein Coding | P42830 | 51 | GC04M073995 |
| SCG5 | Secretogranin V | Protein Coding | P05408 | 51 | GC15P032641 |
| CTSK | Cathepsin K | Protein Coding | P43235 | 62 | GC01M164912 |
| FH | Fumarate Hydratase | Protein Coding | P07954 | 59 | GC01M241499 |
| NCAM1 | Neural Cell Adhesion Molecule 1 | Protein Coding | P13591 | 61 | GC11P112961 |
| CCL17 | C-C Motif Chemokine Ligand 17 | Protein Coding | Q92583 | 52 | GC16P105611 |
| POT1 | Protection Of Telomeres 1 | Protein Coding | Q9NUX5 | 55 | GC07M124822 |
| ATRX | ATRX Chromatin Remodeler | Protein Coding | P46100 | 58 | GC0XM077504 |
| KIR2DL1 | Killer Cell Immunoglobulin Like Receptor, Two Ig Domains And Long Cytoplasmic Tail 1 | Protein Coding | P43626 | 47 | GC19P133681 |
| INAVA | Innate Immunity Activator | Protein Coding | Q3KP66 | 45 | GC01P200892 |
| CRH | Corticotropin Releasing Hormone | Protein Coding | P06850 | 55 | GC08M066176 |
| PHOX2B | Paired Like Homeobox 2B | Protein Coding | Q99453 | 56 | GC04M041746 |
| SUFU | SUFU Negative Regulator Of Hedgehog Signaling | Protein Coding | Q9UMX1 | 55 | GC10P116553 |
| APOL1 | Apolipoprotein L1 | Protein Coding | O14791 | 55 | GC22P036253 |
| AFG3L2 | AFG3 Like Matrix AAA Peptidase Subunit 2 | Protein Coding | Q9Y4W6 | 57 | GC18M012328 |
| RNF216 | Ring Finger Protein 216 | Protein Coding | Q9NWF9 | 55 | GC07M005620 |
| BANK1 | B Cell Scaffold Protein With Ankyrin Repeats 1 | Protein Coding | Q8NDB2 | 48 | GC04P101411 |
| SPTBN2 | Spectrin Beta, Non-Erythrocytic 2 | Protein Coding | O15020 | 54 | GC11M132892 |
| MAPT | Microtubule Associated Protein Tau | Protein Coding | P10636 | 62 | GC17P045894 |
| MIR222 | MicroRNA 222 | RNA Gene |  | 29 | GC0XM045747 |
| IFIT1 | Interferon Induced Protein With Tetratricopeptide Repeats 1 | Protein Coding | P09914 | 50 | GC10P116138 |
| BARD1 | BRCA1 Associated RING Domain 1 | Protein Coding | Q99728 | 59 | GC02M214725 |
| RIPK3 | Receptor Interacting Serine/Threonine Kinase 3 | Protein Coding | Q9Y572 | 56 | GC14M024336 |
| KIR2DL2 | Killer Cell Immunoglobulin Like Receptor, Two Ig Domains And Long Cytoplasmic Tail 2 | Protein Coding | P43627 | 36 | GC19MR00107 |
| IFNL1 | Interferon Lambda 1 | Protein Coding | Q8IU54 | 48 | GC19P039296 |
| PDGFRB | Platelet Derived Growth Factor Receptor Beta | Protein Coding | P09619 | 66 | GC05M150113 |
| ICAM3 | Intercellular Adhesion Molecule 3 | Protein Coding | P32942 | 54 | GC19M100265 |
| CASP9 | Caspase 9 | Protein Coding | P55211 | 60 | GC01M019993 |
| IL36G | Interleukin 36 Gamma | Protein Coding | Q9NZH8 | 51 | GC02P112973 |
| PNKP | Polynucleotide Kinase 3'-Phosphatase | Protein Coding | Q96T60 | 59 | GC19M101302 |
| SARM1 | Sterile Alpha And TIR Motif Containing 1 | Protein Coding | Q6SZW1 | 54 | GC17P028364 |
| CX3CL1 | C-X3-C Motif Chemokine Ligand 1 | Protein Coding | P78423 | 56 | GC16P057372 |
| TPO | Thyroid Peroxidase | Protein Coding | P07202 | 61 | GC02P001374 |
| RUBCN | Rubicon Autophagy Regulator | Protein Coding | Q92622 | 52 | GC03M199067 |
| TG | Thyroglobulin | Protein Coding | P01266 | 57 | GC08P132866 |
| IL5RA | Interleukin 5 Receptor Subunit Alpha | Protein Coding | Q01344 | 58 | GC03M003066 |
| AGER | Advanced Glycosylation End-Product Specific Receptor | Protein Coding | Q15109 | 59 | GC06M032180 |
| NOS3 | Nitric Oxide Synthase 3 | Protein Coding | P29474 | 62 | GC07P161731 |
| NFKBIB | NFKB Inhibitor Beta | Protein Coding | Q15653 | 52 | GC19P038899 |
| LMNB1 | Lamin B1 | Protein Coding | P20700 | 59 | GC05P126776 |
| CCR9 | C-C Motif Chemokine Receptor 9 | Protein Coding | P51686 | 51 | GC03P061342 |
| DNAJC3 | DnaJ Heat Shock Protein Family (Hsp40) Member C3 | Protein Coding | Q13217 | 52 | GC13P095677 |
| GHR | Growth Hormone Receptor | Protein Coding | P10912 | 58 | GC05P042429 |
| VWF | Von Willebrand Factor | Protein Coding | P04275 | 60 | GC12M032666 |
| CHIT1 | Chitinase 1 | Protein Coding | Q13231 | 56 | GC01M203213 |
| SLC2A1 | Solute Carrier Family 2 Member 1 | Protein Coding | P11166 | 66 | GC01M042925 |
| BDNF | Brain Derived Neurotrophic Factor | Protein Coding | P23560 | 62 | GC11M027654 |
| PRKN | Parkin RBR E3 Ubiquitin Protein Ligase | Protein Coding | O60260 | 62 | GC06M161348 |
| MAPK9 | Mitogen-Activated Protein Kinase 9 | Protein Coding | P45984 | 61 | GC05M182811 |
| PMEL | Premelanosome Protein | Protein Coding | P40967 | 56 | GC12M055954 |
| XPA | XPA, DNA Damage Recognition And Repair Factor | Protein Coding | P23025 | 58 | GC09M097654 |
| CREB1 | CAMP Responsive Element Binding Protein 1 | Protein Coding | P16220 | 62 | GC02P207529 |
| HLA-DQB2 | Major Histocompatibility Complex, Class II, DQ Beta 2 | Protein Coding | P05538 | 47 | GC06M032756 |
| AP3D1 | Adaptor Related Protein Complex 3 Subunit Delta 1 | Protein Coding | O14617 | 54 | GC19M099946 |
| MIR200B | MicroRNA 200b | RNA Gene |  | 30 | GC01P001167 |
| GBE1 | 1,4-Alpha-Glucan Branching Enzyme 1 | Protein Coding | Q04446 | 56 | GC03M081489 |
| GSDMD | Gasdermin D | Protein Coding | P57764 | 52 | GC08P143553 |
| PI3 | Peptidase Inhibitor 3 | Protein Coding | P19957 | 50 | GC20P045174 |
| SIGLEC10 | Sialic Acid Binding Ig Like Lectin 10 | Protein Coding | Q96LC7 | 49 | GC19M051410 |
| IKZF3 | IKAROS Family Zinc Finger 3 | Protein Coding | Q9UKT9 | 54 | GC17M089263 |
| EZR | Ezrin | Protein Coding | P15311 | 59 | GC06M158765 |
| SIGLEC1 | Sialic Acid Binding Ig Like Lectin 1 | Protein Coding | Q9BZZ2 | 52 | GC20M003686 |
| IFI27 | Interferon Alpha Inducible Protein 27 | Protein Coding | P40305 | 50 | GC14P094104 |
| F2 | Coagulation Factor II, Thrombin | Protein Coding | P00734 | 63 | GC11P048362 |
| CD200 | CD200 Molecule | Protein Coding | P41217 | 53 | GC03P112332 |
| LIPA | Lipase A, Lysosomal Acid Type | Protein Coding | P38571 | 61 | GC10M089213 |
| SIRT1 | Sirtuin 1 | Protein Coding | Q96EB6 | 62 | GC10P067884 |
| MIR92A1 | MicroRNA 92a-1 | RNA Gene |  | 28 | GC13P091718 |
| HSPA4 | Heat Shock Protein Family A (Hsp70) Member 4 | Protein Coding | P34932 | 55 | GC05P133097 |
| IL1R2 | Interleukin 1 Receptor Type 2 | Protein Coding | P27930 | 58 | GC02P101991 |
| SERPINA1 | Serpin Family A Member 1 | Protein Coding | P01009 | 62 | GC14M094376 |
| FFAR2 | Free Fatty Acid Receptor 2 | Protein Coding | O15552 | 52 | GC19P132918 |
| TRIM32 | Tripartite Motif Containing 32 | Protein Coding | Q13049 | 55 | GC09P116687 |
| IDH2 | Isocitrate Dehydrogenase (NADP(+)) 2 | Protein Coding | P48735 | 65 | GC15M090083 |
| MIR23B | MicroRNA 23b | RNA Gene |  | 29 | GC09P095085 |
| IL17RE | Interleukin 17 Receptor E | Protein Coding | Q8NFR9 | 50 | GC03P024187 |
| RORA | RAR Related Orphan Receptor A | Protein Coding | P35398 | 61 | GC15M060488 |
| ARG2 | Arginase 2 | Protein Coding | P78540 | 59 | GC14P067619 |
| ABCB1 | ATP Binding Cassette Subfamily B Member 1 | Protein Coding | P08183 | 64 | GC07M087504 |
| MIR23A | MicroRNA 23a | RNA Gene |  | 30 | GC19M100412 |
| CCL24 | C-C Motif Chemokine Ligand 24 | Protein Coding | O00175 | 50 | GC07M079197 |
| NALT1 | NOTCH1 Associated LncRNA In T Cell Acute Lymphoblastic Leukemia 1 | RNA Gene |  | 21 | GC09P147106 |
| ACE | Angiotensin I Converting Enzyme | Protein Coding | P12821 | 64 | GC17P063477 |
| TOM1 | Target Of Myb1 Membrane Trafficking Protein | Protein Coding | O60784 | 51 | GC22P035299 |
| PSMB10 | Proteasome 20S Subunit Beta 10 | Protein Coding | P40306 | 56 | GC16M067937 |
| CTSD | Cathepsin D | Protein Coding | P07339 | 65 | GC11M001752 |
| MIR335 | MicroRNA 335 | RNA Gene |  | 29 | GC07P130496 |
| PSEN1 | Presenilin 1 | Protein Coding | P49768 | 66 | GC14P073136 |
| THBS1 | Thrombospondin 1 | Protein Coding | P07996 | 58 | GC15P039581 |
| CLEC10A | C-Type Lectin Domain Containing 10A | Protein Coding | Q8IUN9 | 52 | GC17M007074 |
| KLRG1 | Killer Cell Lectin Like Receptor G1 | Protein Coding | Q96E93 | 50 | GC12P008950 |
| DNAH9 | Dynein Axonemal Heavy Chain 9 | Protein Coding | Q9NYC9 | 50 | GC17P011598 |
| UBE2N | Ubiquitin Conjugating Enzyme E2 N | Protein Coding | P61088 | 59 | GC12M093406 |
| PML | PML Nuclear Body Scaffold | Protein Coding | P29590 | 59 | GC15P073994 |
| NOTCH2 | Notch Receptor 2 | Protein Coding | Q04721 | 65 | GC01M119911 |
| BPI | Bactericidal Permeability Increasing Protein | Protein Coding | P17213 | 54 | GC20P038304 |
| TRAT1 | T Cell Receptor Associated Transmembrane Adaptor 1 | Protein Coding | Q6PIZ9 | 47 | GC03P108823 |
| TIMP1 | TIMP Metallopeptidase Inhibitor 1 | Protein Coding | P01033 | 58 | GC0XP059202 |
| CA2 | Carbonic Anhydrase 2 | Protein Coding | P00918 | 64 | GC08P085463 |
| MIR30E | MicroRNA 30e | RNA Gene |  | 31 | GC01P040754 |
| SCARNA5 | Small Cajal Body-Specific RNA 5 | RNA Gene |  | 28 | GC02P233275 |
| RNF135 | Ring Finger Protein 135 | Protein Coding | Q8IUD6 | 52 | GC17P030958 |
| MIR199A1 | MicroRNA 199a-1 | RNA Gene |  | 29 | GC19M010817 |
| PPM1D | Protein Phosphatase, Mg2+/Mn2+ Dependent 1D | Protein Coding | O15297 | 60 | GC17P060600 |
| MIR181A1 | MicroRNA 181a-1 | RNA Gene |  | 27 | GC01M198860 |
| OSM | Oncostatin M | Protein Coding | P13725 | 55 | GC22M030262 |
| CD1C | CD1c Molecule | Protein Coding | P29017 | 50 | GC01P158289 |
| MIR326 | MicroRNA 326 | RNA Gene |  | 30 | GC11M075335 |
| CCL13 | C-C Motif Chemokine Ligand 13 | Protein Coding | Q99616 | 48 | GC17P034356 |
| NCR1 | Natural Cytotoxicity Triggering Receptor 1 | Protein Coding | O76036 | 51 | GC19P133689 |
| CACNA1C | Calcium Voltage-Gated Channel Subunit Alpha1 C | Protein Coding | Q13936 | 61 | GC12P001970 |
| EMSLR | E2F1 MRNA Stabilizing LncRNA | RNA Gene |  | 20 | GC07P108819 |
| TFAP2A | Transcription Factor AP-2 Alpha | Protein Coding | P05549 | 61 | GC06M010393 |
| CD2 | CD2 Molecule | Protein Coding | P06729 | 56 | GC01P116754 |
| NCKAP1L | NCK Associated Protein 1 Like | Protein Coding | P55160 | 48 | GC12P054497 |
| FOXO3 | Forkhead Box O3 | Protein Coding | O43524 | 60 | GC06P108559 |
| BIRC5 | Baculoviral IAP Repeat Containing 5 | Protein Coding | O15392 | 58 | GC17P078214 |
| STXBP3 | Syntaxin Binding Protein 3 | Protein Coding | O00186 | 52 | GC01P108746 |
| PLA2G2A | Phospholipase A2 Group IIA | Protein Coding | P14555 | 59 | GC01M019975 |
| CISH | Cytokine Inducible SH2 Containing Protein | Protein Coding | Q9NSE2 | 57 | GC03M054344 |
| MTHFR | Methylenetetrahydrofolate Reductase | Protein Coding | P42898 | 61 | GC01M011785 |
| FAS-AS1 | FAS Antisense RNA 1 | RNA Gene |  | 22 | GC10M088991 |
| IFITM1 | Interferon Induced Transmembrane Protein 1 | Protein Coding | P13164 | 52 | GC11P013987 |
| ZEB1 | Zinc Finger E-Box Binding Homeobox 1 | Protein Coding | P37275 | 62 | GC10P031318 |
| FPR2 | Formyl Peptide Receptor 2 | Protein Coding | P25090 | 58 | GC19P051752 |
| CALM3 | Calmodulin 3 | Protein Coding | P0DP25 | 55 | GC19P046601 |
| ITGA2B | Integrin Subunit Alpha 2b | Protein Coding | P08514 | 63 | GC17M089470 |
| SPG7 | SPG7 Matrix AAA Peptidase Subunit, Paraplegin | Protein Coding | Q9UQ90 | 55 | GC16P107070 |
| GBP1 | Guanylate Binding Protein 1 | Protein Coding | P32455 | 51 | GC01M089052 |
| IFITM3 | Interferon Induced Transmembrane Protein 3 | Protein Coding | Q01628 | 54 | GC11M000319 |
| MIR143 | MicroRNA 143 | RNA Gene |  | 33 | GC05P155623 |
| CD83 | CD83 Molecule | Protein Coding | Q01151 | 53 | GC06P014117 |
| ADORA2A | Adenosine A2a Receptor | Protein Coding | P29274 | 59 | GC22P024417 |
| C8G | Complement C8 Gamma Chain | Protein Coding | P07360 | 51 | GC09P147140 |
| NFKBIE | NFKB Inhibitor Epsilon | Protein Coding | O00221 | 51 | GC06M044258 |
| CD300LF | CD300 Molecule Like Family Member F | Protein Coding | Q8TDQ1 | 48 | GC17M074694 |
| TNFSF14 | TNF Superfamily Member 14 | Protein Coding | O43557 | 53 | GC19M100109 |
| AXIN2 | Axin 2 | Protein Coding | Q9Y2T1 | 62 | GC17M065528 |
| CCL8 | C-C Motif Chemokine Ligand 8 | Protein Coding | P80075 | 51 | GC17P034319 |
| IL31 | Interleukin 31 | Protein Coding | Q6EBC2 | 50 | GC12M124303 |
| MAX | MYC Associated Factor X | Protein Coding | P61244 | 59 | GC14M065368 |
| SMAD2 | SMAD Family Member 2 | Protein Coding | Q15796 | 65 | GC18M047809 |
| MIR99B | MicroRNA 99b | RNA Gene |  | 29 | GC19P051692 |
| CHI3L1 | Chitinase 3 Like 1 | Protein Coding | P36222 | 58 | GC01M203148 |
| FAAP24 | FA Core Complex Associated Protein 24 | Protein Coding | Q9BTP7 | 47 | GC19P132862 |
| ERVW-1 | Endogenous Retrovirus Group W Member 1, Envelope | Protein Coding | Q9UQF0 | 47 | GC07M092468 |
| TASL | TLR Adaptor Interacting With Endolysosomal SLC15A4 | Protein Coding | Q9HAI6 | 37 | GC0XM030559 |
| EPX | Eosinophil Peroxidase | Protein Coding | P11678 | 56 | GC17P058192 |
| MIR18A | MicroRNA 18a | RNA Gene |  | 27 | GC13P091719 |
| CCL26 | C-C Motif Chemokine Ligand 26 | Protein Coding | Q9Y258 | 50 | GC07M075769 |
| MICA | MHC Class I Polypeptide-Related Sequence A | Protein Coding | Q29983 | 51 | GC06P031399 |
| PRKCZ | Protein Kinase C Zeta | Protein Coding | Q05513 | 61 | GC01P059904 |
| ITGA2 | Integrin Subunit Alpha 2 | Protein Coding | P17301 | 58 | GC05P052989 |
| CAV1 | Caveolin 1 | Protein Coding | Q03135 | 61 | GC07P116524 |
| SOS2 | SOS Ras/Rho Guanine Nucleotide Exchange Factor 2 | Protein Coding | Q07890 | 56 | GC14M050117 |
| PPIA | Peptidylprolyl Isomerase A | Protein Coding | P62937 | 58 | GC07P044839 |
| IL1F10 | Interleukin 1 Family Member 10 | Protein Coding | Q8WWZ1 | 48 | GC02P113067 |
| AR | Androgen Receptor | Protein Coding | P10275 | 65 | GC0XP067544 |
| HIRA | Histone Cell Cycle Regulator | Protein Coding | P54198 | 54 | GC22M019330 |
| IFIT2 | Interferon Induced Protein With Tetratricopeptide Repeats 2 | Protein Coding | P09913 | 49 | GC10P116137 |
| SMARCE1 | SWI/SNF Related, Matrix Associated, Actin Dependent Regulator Of Chromatin, Subfamily E, Member 1 | Protein Coding | Q969G3 | 58 | GC17M040624 |
| PDGFB | Platelet Derived Growth Factor Subunit B | Protein Coding | P01127 | 63 | GC22M082423 |
| PKM | Pyruvate Kinase M1/2 | Protein Coding | P14618 | 62 | GC15M072199 |
| SELPLG | Selectin P Ligand | Protein Coding | Q14242 | 56 | GC12M108621 |
| MYH9 | Myosin Heavy Chain 9 | Protein Coding | P35579 | 60 | GC22M036281 |
| HEXA | Hexosaminidase Subunit Alpha | Protein Coding | P06865 | 59 | GC15M072340 |
| PRKACA | Protein Kinase CAMP-Activated Catalytic Subunit Alpha | Protein Coding | P17612 | 66 | GC19M100419 |
| AXL | AXL Receptor Tyrosine Kinase | Protein Coding | P30530 | 64 | GC19P041219 |
| FTH1 | Ferritin Heavy Chain 1 | Protein Coding | P02794 | 63 | GC11M061959 |
| CXCR1 | C-X-C Motif Chemokine Receptor 1 | Protein Coding | P25024 | 56 | GC02M218162 |
| MIR195 | MicroRNA 195 | RNA Gene |  | 28 | GC17M088262 |
| MIR130A | MicroRNA 130a | RNA Gene |  | 30 | GC11P057641 |
| RECQL | RecQ Like Helicase | Protein Coding | P46063 | 54 | GC12M021468 |
| PTGER4 | Prostaglandin E Receptor 4 | Protein Coding | P35408 | 58 | GC05P040679 |
| MIR27A | MicroRNA 27a | RNA Gene |  | 31 | GC19M100411 |
| RAB7A | RAB7A, Member RAS Oncogene Family | Protein Coding | P51149 | 62 | GC03P140877 |
| CALCRL | Calcitonin Receptor Like Receptor | Protein Coding | Q16602 | 58 | GC02M187341 |
| FGF10 | Fibroblast Growth Factor 10 | Protein Coding | O15520 | 60 | GC05M045056 |
| RUNX2 | RUNX Family Transcription Factor 2 | Protein Coding | Q13950 | 59 | GC06P156520 |
| IL24 | Interleukin 24 | Protein Coding | Q13007 | 53 | GC01P206897 |
| FXN | Frataxin | Protein Coding | Q16595 | 59 | GC09P069035 |
| CTSB | Cathepsin B | Protein Coding | P07858 | 63 | GC08M011842 |
| CD48 | CD48 Molecule | Protein Coding | P09326 | 52 | GC01M165315 |
| MAPK13 | Mitogen-Activated Protein Kinase 13 | Protein Coding | O15264 | 59 | GC06P156438 |
| FGA | Fibrinogen Alpha Chain | Protein Coding | P02671 | 62 | GC04M154583 |
| LRP1 | LDL Receptor Related Protein 1 | Protein Coding | Q07954 | 61 | GC12P057128 |
| SLC22A4 | Solute Carrier Family 22 Member 4 | Protein Coding | Q9H015 | 57 | GC05P132294 |
| PIGR | Polymeric Immunoglobulin Receptor | Protein Coding | P01833 | 54 | GC01M206928 |
| IGF2 | Insulin Like Growth Factor 2 | Protein Coding | P01344 | 61 | GC11M013301 |
| EDA | Ectodysplasin A | Protein Coding | Q92838 | 54 | GC0XP069618 |
| MUC2 | Mucin 2, Oligomeric Mucus/Gel-Forming | Protein Coding | Q02817 | 52 | GC11P001074 |
| IL4I1 | Interleukin 4 Induced 1 | Protein Coding | Q96RQ9 | 50 | GC19M101308 |
| GRID2 | Glutamate Ionotropic Receptor Delta Type Subunit 2 | Protein Coding | O43424 | 57 | GC04P092304 |
| MIR20B | MicroRNA 20b | RNA Gene |  | 23 | GC0XM134523 |
| IL1RAP | Interleukin 1 Receptor Accessory Protein | Protein Coding | Q9NPH3 | 58 | GC03P190514 |
| IL36A | Interleukin 36 Alpha | Protein Coding | Q9UHA7 | 48 | GC02P113005 |
| MIR93 | MicroRNA 93 | RNA Gene |  | 30 | GC07M106066 |
| MIR106A | MicroRNA 106a | RNA Gene |  | 27 | GC0XM134526 |
| CCL1 | C-C Motif Chemokine Ligand 1 | Protein Coding | P22362 | 49 | GC17M088983 |
| PPBP | Pro-Platelet Basic Protein | Protein Coding | P02775 | 55 | GC04M073986 |
| AARS1 | Alanyl-TRNA Synthetase 1 | Protein Coding | P49588 | 56 | GC16M073843 |
| GP1BB | Glycoprotein Ib Platelet Subunit Beta | Protein Coding | P13224 | 55 | GC22P080833 |
| IL13RA2 | Interleukin 13 Receptor Subunit Alpha 2 | Protein Coding | Q14627 | 50 | GC0XM115003 |
| CD1B | CD1b Molecule | Protein Coding | P29016 | 50 | GC01M165259 |
| CRLF1 | Cytokine Receptor Like Factor 1 | Protein Coding | O75462 | 56 | GC19M018572 |
| MIRLET7D | MicroRNA Let-7d | RNA Gene |  | 31 | GC09P094178 |
| GP2 | Glycoprotein 2 | Protein Coding | P55259 | 51 | GC16M020309 |
| TTN | Titin | Protein Coding | Q8WZ42 | 61 | GC02M178525 |
| MIR22 | MicroRNA 22 | RNA Gene |  | 31 | GC17M001713 |
| ITIH4 | Inter-Alpha-Trypsin Inhibitor Heavy Chain 4 | Protein Coding | Q14624 | 54 | GC03M052812 |
| IFI6 | Interferon Alpha Inducible Protein 6 | Protein Coding | P09912 | 47 | GC01M027666 |
| HARS1 | Histidyl-TRNA Synthetase 1 | Protein Coding | P12081 | 56 | GC05M140673 |
| IRAK2 | Interleukin 1 Receptor Associated Kinase 2 | Protein Coding | O43187 | 52 | GC03P024216 |
| LY9 | Lymphocyte Antigen 9 | Protein Coding | Q9HBG7 | 50 | GC01P160796 |
| CD24 | CD24 Molecule | Protein Coding | P25063 | 46 | GC06M106969 |
| MIR373 | MicroRNA 373 | RNA Gene |  | 29 | GC19P133648 |
| TDP1 | Tyrosyl-DNA Phosphodiesterase 1 | Protein Coding | Q9NUW8 | 57 | GC14P089954 |
| MCL1 | MCL1 Apoptosis Regulator, BCL2 Family Member | Protein Coding | Q07820 | 61 | GC01M164881 |
| FCAR | Fc Alpha Receptor | Protein Coding | P24071 | 52 | GC19P133687 |
| DST | Dystonin | Protein Coding | Q03001 | 55 | GC06M056457 |
| MAP3K1 | Mitogen-Activated Protein Kinase Kinase Kinase 1 | Protein Coding | Q13233 | 62 | GC05P056815 |
| IFNLR1 | Interferon Lambda Receptor 1 | Protein Coding | Q8IU57 | 45 | GC01M032031 |
| MIR29C | MicroRNA 29c | RNA Gene |  | 27 | GC01M207838 |
| POLR3A | RNA Polymerase III Subunit A | Protein Coding | O14802 | 57 | GC10M079241 |
| ISG20 | Interferon Stimulated Exonuclease Gene 20 | Protein Coding | Q96AZ6 | 50 | GC15P088635 |
| GRN | Granulin Precursor | Protein Coding | P28799 | 61 | GC17P044345 |
| CYP27A1 | Cytochrome P450 Family 27 Subfamily A Member 1 | Protein Coding | Q02318 | 59 | GC02P218781 |
| SACS | Sacsin Molecular Chaperone | Protein Coding | Q9NZJ4 | 50 | GC13M023288 |
| SPHK1 | Sphingosine Kinase 1 | Protein Coding | Q9NYA1 | 58 | GC17P076376 |
| SDHD | Succinate Dehydrogenase Complex Subunit D | Protein Coding | O14521 | 57 | GC11P112740 |
| HGF | Hepatocyte Growth Factor | Protein Coding | P14210 | 65 | GC07M081699 |
| SIGIRR | Single Ig And TIR Domain Containing | Protein Coding | Q6IA17 | 52 | GC11M013212 |
| CANX | Calnexin | Protein Coding | P27824 | 58 | GC05P179678 |
| TUG1 | Taurine Up-Regulated 1 | Protein Coding | A0A6I8PU40 | 33 | GC22P030969 |
| PAX6 | Paired Box 6 | Protein Coding | P26367 | 61 | GC11M031784 |
| SP1 | Sp1 Transcription Factor | Protein Coding | P08047 | 58 | GC12P053380 |
| ZC3H12A | Zinc Finger CCCH-Type Containing 12A | Protein Coding | Q5D1E8 | 49 | GC01P037474 |
| UBA5 | Ubiquitin Like Modifier Activating Enzyme 5 | Protein Coding | Q9GZZ9 | 52 | GC03P132654 |
| COQ8A | Coenzyme Q8A | Protein Coding | Q8NI60 | 55 | GC01P226939 |
| TNFSF18 | TNF Superfamily Member 18 | Protein Coding | Q9UNG2 | 47 | GC01M173009 |
| MIR125B1 | MicroRNA 125b-1 | RNA Gene |  | 30 | GC11M134117 |
| OCLN | Occludin | Protein Coding | Q16625 | 58 | GC05P069492 |
| MUC5B | Mucin 5B, Oligomeric Mucus/Gel-Forming | Protein Coding | Q9HC84 | 54 | GC11P014074 |
| HDAC2 | Histone Deacetylase 2 | Protein Coding | Q92769 | 65 | GC06M113933 |
| TAB1 | TGF-Beta Activated Kinase 1 (MAP3K7) Binding Protein 1 | Protein Coding | Q15750 | 55 | GC22P081529 |
| MYB | MYB Proto-Oncogene, Transcription Factor | Protein Coding | P10242 | 62 | GC06P135181 |
| UBN1 | Ubinuclein 1 | Protein Coding | Q9NPG3 | 50 | GC16P104129 |
| EXO1 | Exonuclease 1 | Protein Coding | Q9UQ84 | 55 | GC01P241847 |
| IFIT3 | Interferon Induced Protein With Tetratricopeptide Repeats 3 | Protein Coding | O14879 | 50 | GC10P089327 |
| PRKCB | Protein Kinase C Beta | Protein Coding | P05771 | 62 | GC16P104638 |
| IFNL2 | Interferon Lambda 2 | Protein Coding | Q8IZJ0 | 44 | GC19P039268 |
| MIR25 | MicroRNA 25 | RNA Gene |  | 29 | GC07M100093 |
| MIR141 | MicroRNA 141 | RNA Gene |  | 30 | GC12P046793 |
| COL17A1 | Collagen Type XVII Alpha 1 Chain | Protein Coding | Q9UMD9 | 58 | GC10M104031 |
| MIR148A | MicroRNA 148a | RNA Gene |  | 29 | GC07M025950 |
| EOMES | Eomesodermin | Protein Coding | O95936 | 56 | GC03M027715 |
| MSR1 | Macrophage Scavenger Receptor 1 | Protein Coding | P21757 | 58 | GC08M016107 |
| SFTPA1 | Surfactant Protein A1 | Protein Coding | Q8IWL2 | 58 | GC10P119662 |
| MIR34C | MicroRNA 34c | RNA Gene |  | 30 | GC11P112711 |
| NT5E | 5'-Nucleotidase Ecto | Protein Coding | P21589 | 65 | GC06P085449 |
| PRPS1 | Phosphoribosyl Pyrophosphate Synthetase 1 | Protein Coding | P60891 | 59 | GC0XP107628 |
| F2RL1 | F2R Like Trypsin Receptor 1 | Protein Coding | P55085 | 56 | GC05P076818 |
| UBA1 | Ubiquitin Like Modifier Activating Enzyme 1 | Protein Coding | P22314 | 61 | GC0XP047190 |
| FCAMR | Fc Alpha And Mu Receptor | Protein Coding | Q8WWV6 | 45 | GC01M206957 |
| IRS1 | Insulin Receptor Substrate 1 | Protein Coding | P35568 | 61 | GC02M226731 |
| PAK2 | P21 (RAC1) Activated Kinase 2 | Protein Coding | Q13177 | 59 | GC03P196739 |
| HSPA8 | Heat Shock Protein Family A (Hsp70) Member 8 | Protein Coding | P11142 | 62 | GC11M123057 |
| SST | Somatostatin | Protein Coding | P61278 | 52 | GC03M187668 |
| LGALS1 | Galectin 1 | Protein Coding | P09382 | 56 | GC22P037675 |
| UBC | Ubiquitin C | Protein Coding | P0CG48 | 56 | GC12M124911 |
| CLEC16A | C-Type Lectin Domain Containing 16A | Protein Coding | Q2KHT3 | 50 | GC16P010944 |
| XPO1 | Exportin 1 | Protein Coding | O14980 | 61 | GC02M061445 |
| TXK | TXK Tyrosine Kinase | Protein Coding | P42681 | 56 | GC04M048066 |
| AP1S3 | Adaptor Related Protein Complex 1 Subunit Sigma 3 | Protein Coding | Q96PC3 | 49 | GC02M223667 |
| OPRM1 | Opioid Receptor Mu 1 | Protein Coding | P35372 | 61 | GC06P163868 |
| MIR342 | MicroRNA 342 | RNA Gene |  | 29 | GC14P100109 |
| MIR424 | MicroRNA 424 | RNA Gene |  | 27 | GC0XM134901 |
| IFNA10 | Interferon Alpha 10 | Protein Coding | P01566 | 47 | GC09M022227 |
| TIFA | TRAF Interacting Protein With Forkhead Associated Domain | Protein Coding | Q96CG3 | 44 | GC04M112274 |
| BATF | Basic Leucine Zipper ATF-Like Transcription Factor | Protein Coding | Q16520 | 51 | GC14P075523 |
| PGK1 | Phosphoglycerate Kinase 1 | Protein Coding | P00558 | 62 | GC0XP078216 |
| BMP2 | Bone Morphogenetic Protein 2 | Protein Coding | P12643 | 58 | GC20P006767 |
| CFHR3 | Complement Factor H Related 3 | Protein Coding | Q02985 | 52 | GC01P196774 |
| FERMT1 | FERM Domain Containing Kindlin 1 | Protein Coding | Q9BQL6 | 54 | GC20M006074 |
| FLNA | Filamin A | Protein Coding | P21333 | 61 | GC0XM154348 |
| EBI3 | Epstein-Barr Virus Induced 3 | Protein Coding | Q14213 | 48 | GC19P131973 |
| MERTK | MER Proto-Oncogene, Tyrosine Kinase | Protein Coding | Q12866 | 63 | GC02P111898 |
| GDNF | Glial Cell Derived Neurotrophic Factor | Protein Coding | P39905 | 62 | GC05M037812 |
| CCL27 | C-C Motif Chemokine Ligand 27 | Protein Coding | Q9Y4X3 | 47 | GC09M035512 |
| LYZ | Lysozyme | Protein Coding | P61626 | 59 | GC12P069348 |
| MIR155HG | MIR155 Host Gene | RNA Gene | C0HMA1 | 30 | GC21P025787 |
| TMPO | Thymopoietin | Protein Coding | P42166 | 58 | GC12P098515 |
| APOB | Apolipoprotein B | Protein Coding | P04114 | 58 | GC02M020956 |
| MIR200A | MicroRNA 200a | RNA Gene |  | 29 | GC01P059855 |
| YAP1 | Yes1 Associated Transcriptional Regulator | Protein Coding | P46937 | 61 | GC11P102110 |
| PRKCG | Protein Kinase C Gamma | Protein Coding | P05129 | 64 | GC19P053879 |
| ITGAV | Integrin Subunit Alpha V | Protein Coding | P06756 | 60 | GC02P186589 |
| MIR127 | MicroRNA 127 | RNA Gene |  | 30 | GC14P118051 |
| PCNA | Proliferating Cell Nuclear Antigen | Protein Coding | P12004 | 63 | GC20M005114 |
| MIR10A | MicroRNA 10a | RNA Gene |  | 30 | GC17M048579 |
| HSPA5 | Heat Shock Protein Family A (Hsp70) Member 5 | Protein Coding | P11021 | 61 | GC09M125234 |
| CAT | Catalase | Protein Coding | P04040 | 64 | GC11P034460 |
| PLA2G10 | Phospholipase A2 Group X | Protein Coding | O15496 | 54 | GC16M014672 |
| BMP4 | Bone Morphogenetic Protein 4 | Protein Coding | P12644 | 62 | GC14M053949 |
| CD68 | CD68 Molecule | Protein Coding | P34810 | 52 | GC17P007579 |
| RARA | Retinoic Acid Receptor Alpha | Protein Coding | P10276 | 62 | GC17P040309 |
| GAD1 | Glutamate Decarboxylase 1 | Protein Coding | Q99259 | 62 | GC02P170813 |
| CTSF | Cathepsin F | Protein Coding | Q9UBX1 | 62 | GC11M132878 |
| SMPD1 | Sphingomyelin Phosphodiesterase 1 | Protein Coding | P17405 | 61 | GC11P006390 |
| ATP6V0A2 | ATPase H+ Transporting V0 Subunit A2 | Protein Coding | Q9Y487 | 55 | GC12P123712 |
| PRNP | Prion Protein (Kanno Blood Group) | Protein Coding | F7VJQ1 | 61 | GC20P004686 |
| PGLYRP3 | Peptidoglycan Recognition Protein 3 | Protein Coding | Q96LB9 | 45 | GC01M153297 |
| KRT1 | Keratin 1 | Protein Coding | P04264 | 60 | GC12M052674 |
| PGM1 | Phosphoglucomutase 1 | Protein Coding | P36871 | 60 | GC01P063593 |
| VIM | Vimentin | Protein Coding | P08670 | 63 | GC10P017227 |
| IL34 | Interleukin 34 | Protein Coding | Q6ZMJ4 | 51 | GC16P106078 |
| ERAP2 | Endoplasmic Reticulum Aminopeptidase 2 | Protein Coding | Q6P179 | 51 | GC05P096875 |
| AAAS | Aladin WD Repeat Nucleoporin | Protein Coding | Q9NRG9 | 51 | GC12M053307 |
| PTAFR | Platelet Activating Factor Receptor | Protein Coding | P25105 | 54 | GC01M028147 |
| MADCAM1 | Mucosal Vascular Addressin Cell Adhesion Molecule 1 | Protein Coding | Q13477 | 52 | GC19P131779 |
| BSG | Basigin (Ok Blood Group) | Protein Coding | P35613 | 56 | GC19P000571 |
| MIR183 | MicroRNA 183 | RNA Gene |  | 27 | GC07M129892 |
| GNE | Glucosamine (UDP-N-Acetyl)-2-Epimerase/N-Acetylmannosamine Kinase | Protein Coding | Q9Y223 | 54 | GC09M036214 |
| ADAM10 | ADAM Metallopeptidase Domain 10 | Protein Coding | O14672 | 66 | GC15M058588 |
| S100B | S100 Calcium Binding Protein B | Protein Coding | P04271 | 57 | GC21M054265 |
| MRC1 | Mannose Receptor C-Type 1 | Protein Coding | P22897 | 51 | GC10P017809 |
| HPS6 | HPS6 Biogenesis Of Lysosomal Organelles Complex 2 Subunit 3 | Protein Coding | Q86YV9 | 49 | GC10P102065 |
| SNX14 | Sorting Nexin 14 | Protein Coding | Q9Y5W7 | 49 | GC06M101046 |
| CD6 | CD6 Molecule | Protein Coding | P30203 | 52 | GC11P060971 |
| KIR3DL2 | Killer Cell Immunoglobulin Like Receptor, Three Ig Domains And Long Cytoplasmic Tail 2 | Protein Coding | P43630 | 50 | GC19P133686 |
| FHL1 | Four And A Half LIM Domains 1 | Protein Coding | Q13642 | 58 | GC0XP136146 |
| MX2 | MX Dynamin Like GTPase 2 | Protein Coding | P20592 | 49 | GC21P041361 |
| RPS6KB1 | Ribosomal Protein S6 Kinase B1 | Protein Coding | P23443 | 62 | GC17P059893 |
| IL26 | Interleukin 26 | Protein Coding | Q9NPH9 | 47 | GC12M068201 |
| MIR99A | MicroRNA 99a | RNA Gene |  | 30 | GC21P016539 |
| MAP3K5 | Mitogen-Activated Protein Kinase Kinase Kinase 5 | Protein Coding | Q99683 | 61 | GC06M136557 |
| SHH | Sonic Hedgehog Signaling Molecule | Protein Coding | Q15465 | 63 | GC07M155799 |
| GNAS | GNAS Complex Locus | Protein Coding | P84996 | 62 | GC20P058839 |
| ANXA5 | Annexin A5 | Protein Coding | P08758 | 59 | GC04M121667 |
| MAOA | Monoamine Oxidase A | Protein Coding | P21397 | 62 | GC0XP043654 |
| HSP90AB1 | Heat Shock Protein 90 Alpha Family Class B Member 1 | Protein Coding | P08238 | 62 | GC06P044246 |
| TUBB3 | Tubulin Beta 3 Class III | Protein Coding | Q13509 | 63 | GC16P107097 |
| PPP3CA | Protein Phosphatase 3 Catalytic Subunit Alpha | Protein Coding | Q08209 | 64 | GC04M101024 |
| PLAUR | Plasminogen Activator, Urokinase Receptor | Protein Coding | Q03405 | 57 | GC19M043646 |
| FGB | Fibrinogen Beta Chain | Protein Coding | P02675 | 59 | GC04P154702 |
| UBB | Ubiquitin B | Protein Coding | P0CG47 | 56 | GC17P016380 |
| OSCAR | Osteoclast Associated Ig-Like Receptor | Protein Coding | Q8IYS5 | 49 | GC19M054094 |
| IPO8 | Importin 8 | Protein Coding | O15397 | 51 | GC12M030628 |
| EGR1 | Early Growth Response 1 | Protein Coding | P18146 | 57 | GC05P138465 |
| BMPR1A | Bone Morphogenetic Protein Receptor Type 1A | Protein Coding | P36894 | 64 | GC10P116052 |
| OAS2 | 2'-5'-Oligoadenylate Synthetase 2 | Protein Coding | P29728 | 49 | GC12P112978 |
| NEFL | Neurofilament Light Chain | Protein Coding | P07196 | 58 | GC08M024950 |
| ABCB7 | ATP Binding Cassette Subfamily B Member 7 | Protein Coding | O75027 | 57 | GC0XM075053 |
| GZMM | Granzyme M | Protein Coding | P51124 | 51 | GC19P000544 |
| GAA | Alpha Glucosidase | Protein Coding | P10253 | 63 | GC17P080101 |
| SLC26A3 | Solute Carrier Family 26 Member 3 | Protein Coding | P40879 | 58 | GC07M107765 |
| MARS2 | Methionyl-TRNA Synthetase 2, Mitochondrial | Protein Coding | Q96GW9 | 54 | GC02P197705 |
| SCN10A | Sodium Voltage-Gated Channel Alpha Subunit 10 | Protein Coding | Q9Y5Y9 | 61 | GC03M038781 |
| ANKRD26 | Ankyrin Repeat Domain Containing 26 | Protein Coding | Q9UPS8 | 51 | GC10M026947 |
| SLC15A4 | Solute Carrier Family 15 Member 4 | Protein Coding | Q8N697 | 48 | GC12M128793 |
| SCARB2 | Scavenger Receptor Class B Member 2 | Protein Coding | Q14108 | 57 | GC04M076158 |
| CDK1 | Cyclin Dependent Kinase 1 | Protein Coding | P06493 | 61 | GC10P060772 |
| ZBTB16 | Zinc Finger And BTB Domain Containing 16 | Protein Coding | Q05516 | 55 | GC11P114059 |
| CEACAM5 | CEA Cell Adhesion Molecule 5 | Protein Coding | P06731 | 56 | GC19P133123 |
| TRAF1 | TNF Receptor Associated Factor 1 | Protein Coding | Q13077 | 53 | GC09M120902 |
| C4BPA | Complement Component 4 Binding Protein Alpha | Protein Coding | P04003 | 54 | GC01P207105 |
| TRBV11-2 | T Cell Receptor Beta Variable 11-2 | Protein Coding | A0A584 | 16 | GC07P163102 |
| SNORD15A | Small Nucleolar RNA, C/D Box 15A | RNA Gene |  | 25 | GC11P075400 |
| ANPEP | Alanyl Aminopeptidase, Membrane | Protein Coding | P15144 | 62 | GC15M089784 |
| MPZ | Myelin Protein Zero | Protein Coding | P25189 | 55 | GC01M165339 |
| RNASET2 | Ribonuclease T2 | Protein Coding | O00584 | 56 | GC06M166924 |
| XPC | XPC Complex Subunit, DNA Damage Recognition And Repair Factor | Protein Coding | Q01831 | 58 | GC03M027401 |
| RN7SL1 | RNA Component Of Signal Recognition Particle 7SL1 | RNA Gene |  | 27 | GC14P052227 |
| CSNK2A1 | Casein Kinase 2 Alpha 1 | Protein Coding | P68400 | 63 | GC20M000472 |
| CTSL | Cathepsin L | Protein Coding | P07711 | 60 | GC09P087725 |
| GPC3 | Glypican 3 | Protein Coding | P51654 | 59 | GC0XM133535 |
| ERCC8 | ERCC Excision Repair 8, CSA Ubiquitin Ligase Complex Subunit | Protein Coding | Q13216 | 56 | GC05M060917 |
| IRF2 | Interferon Regulatory Factor 2 | Protein Coding | P14316 | 56 | GC04M184387 |
| LTB | Lymphotoxin Beta | Protein Coding | Q06643 | 52 | GC06M100300 |
| ITGB7 | Integrin Subunit Beta 7 | Protein Coding | P26010 | 56 | GC12M053191 |
| USP7 | Ubiquitin Specific Peptidase 7 | Protein Coding | Q93009 | 62 | GC16M008892 |
| PPARA | Peroxisome Proliferator Activated Receptor Alpha | Protein Coding | Q07869 | 56 | GC22P046150 |
| SLC9A1 | Solute Carrier Family 9 Member A1 | Protein Coding | P19634 | 62 | GC01M032272 |
| MIR491 | MicroRNA 491 | RNA Gene |  | 29 | GC09P020716 |
| ROCK1 | Rho Associated Coiled-Coil Containing Protein Kinase 1 | Protein Coding | Q13464 | 62 | GC18M036048 |
| MDFIC | MyoD Family Inhibitor Domain Containing | Protein Coding | Q9P1T7 | 47 | GC07P114922 |
| MIR139 | MicroRNA 139 | RNA Gene |  | 30 | GC11M072615 |
| ALPL | Alkaline Phosphatase, Biomineralization Associated | Protein Coding | P05186 | 63 | GC01P021508 |
| MTPAP | Mitochondrial Poly(A) Polymerase | Protein Coding | Q9NVV4 | 52 | GC10M036341 |
| PURA | Purine Rich Element Binding Protein A | Protein Coding | Q00577 | 57 | GC05P155393 |
| CDK6 | Cyclin Dependent Kinase 6 | Protein Coding | Q00534 | 64 | GC07M092604 |
| BCL2L11 | BCL2 Like 11 | Protein Coding | O43521 | 58 | GC02P111119 |
| TXN | Thioredoxin | Protein Coding | P10599 | 58 | GC09M110243 |
| GOSR2 | Golgi SNAP Receptor Complex Member 2 | Protein Coding | O14653 | 54 | GC17P133967 |
| TNFRSF10A | TNF Receptor Superfamily Member 10a | Protein Coding | O00220 | 57 | GC08M023190 |
| MUC5AC | Mucin 5AC, Oligomeric Mucus/Gel-Forming | Protein Coding | P98088 | 51 | GC11P014072 |
| CARD8 | Caspase Recruitment Domain Family Member 8 | Protein Coding | Q9Y2G2 | 50 | GC19M101228 |
| FLT1 | Fms Related Receptor Tyrosine Kinase 1 | Protein Coding | P17948 | 63 | GC13M028300 |
| MIR192 | MicroRNA 192 | RNA Gene |  | 30 | GC11M064891 |
| PWAR1 | Prader Willi/Angelman Region RNA 1 | RNA Gene |  | 23 | GC15P025135 |
| CNTNAP1 | Contactin Associated Protein 1 | Protein Coding | P78357 | 55 | GC17P042682 |
| NCK1 | NCK Adaptor Protein 1 | Protein Coding | P16333 | 55 | GC03P136862 |
| TNFAIP6 | TNF Alpha Induced Protein 6 | Protein Coding | P98066 | 52 | GC02P151357 |
| NEDD4 | NEDD4 E3 Ubiquitin Protein Ligase | Protein Coding | P46934 | 60 | GC15M055826 |
| MUTYH | MutY DNA Glycosylase | Protein Coding | Q9UIF7 | 57 | GC01M045329 |
| KAT5 | Lysine Acetyltransferase 5 | Protein Coding | Q92993 | 63 | GC11P065711 |
| NSUN2 | NOP2/Sun RNA Methyltransferase 2 | Protein Coding | Q08J23 | 55 | GC05M006599 |
| GFAP | Glial Fibrillary Acidic Protein | Protein Coding | P14136 | 61 | GC17M089491 |
| CCL25 | C-C Motif Chemokine Ligand 25 | Protein Coding | O15444 | 49 | GC19P008052 |
| MAPK12 | Mitogen-Activated Protein Kinase 12 | Protein Coding | P53778 | 59 | GC22M081719 |
| NCR3 | Natural Cytotoxicity Triggering Receptor 3 | Protein Coding | O14931 | 52 | GC06M031588 |
| HAVCR1 | Hepatitis A Virus Cellular Receptor 1 | Protein Coding | Q96D42 | 55 | GC05M157028 |
| ALPK1 | Alpha Kinase 1 | Protein Coding | Q96QP1 | 50 | GC04P112285 |
| HCST | Hematopoietic Cell Signal Transducer | Protein Coding | Q9UBK5 | 46 | GC19P132937 |
| ARID1B | AT-Rich Interaction Domain 1B | Protein Coding | Q8NFD5 | 56 | GC06P164661 |
| GIMAP4 | GTPase, IMAP Family Member 4 | Protein Coding | Q9NUV9 | 45 | GC07P161723 |
| GP1BA | Glycoprotein Ib Platelet Subunit Alpha | Protein Coding | P07359 | 59 | GC17P004932 |
| S1PR1 | Sphingosine-1-Phosphate Receptor 1 | Protein Coding | P21453 | 58 | GC01P101236 |
| ZBP1 | Z-DNA Binding Protein 1 | Protein Coding | Q9H171 | 52 | GC20M057603 |
| NAGLU | N-Acetyl-Alpha-Glucosaminidase | Protein Coding | P54802 | 56 | GC17P133798 |
| FLCN | Folliculin | Protein Coding | Q8NFG4 | 54 | GC17M017212 |
| ABCC4 | ATP Binding Cassette Subfamily C Member 4 (PEL Blood Group) | Protein Coding | O15439 | 59 | GC13M095019 |
| SPN | Sialophorin | Protein Coding | P16150 | 53 | GC16P029662 |
| MIR32 | MicroRNA 32 | RNA Gene |  | 31 | GC09M109046 |
| FBXW7 | F-Box And WD Repeat Domain Containing 7 | Protein Coding | Q969H0 | 58 | GC04M152321 |
| PIK3C3 | Phosphatidylinositol 3-Kinase Catalytic Subunit Type 3 | Protein Coding | Q8NEB9 | 61 | GC18P041955 |
| TMPRSS2 | Transmembrane Serine Protease 2 | Protein Coding | O15393 | 60 | GC21M041464 |
| ADRB2 | Adrenoceptor Beta 2 | Protein Coding | P07550 | 61 | GC05P155594 |
| JMJD1C | Jumonji Domain Containing 1C | Protein Coding | Q15652 | 53 | GC10M063167 |
| PTK2 | Protein Tyrosine Kinase 2 | Protein Coding | Q05397 | 61 | GC08M140657 |
| SEMA3A | Semaphorin 3A | Protein Coding | Q14563 | 58 | GC07M083955 |
| BTRC | Beta-Transducin Repeat Containing E3 Ubiquitin Protein Ligase | Protein Coding | Q9Y297 | 56 | GC10P101354 |
| TGIF1 | TGFB Induced Factor Homeobox 1 | Protein Coding | Q15583 | 58 | GC18P003411 |
| NCR2 | Natural Cytotoxicity Triggering Receptor 2 | Protein Coding | O95944 | 51 | GC06P156478 |
| HSPA1B | Heat Shock Protein Family A (Hsp70) Member 1B | Protein Coding | P0DMV9 | 52 | GC06P156261 |
| TYRO3 | TYRO3 Protein Tyrosine Kinase | Protein Coding | Q06418 | 59 | GC15P041557 |
| DUOX2 | Dual Oxidase 2 | Protein Coding | Q9NRD8 | 55 | GC15M045092 |
| LSM11 | LSM11, U7 Small Nuclear RNA Associated | Protein Coding | P83369 | 47 | GC05P157743 |
| MUSK | Muscle Associated Receptor Tyrosine Kinase | Protein Coding | O15146 | 61 | GC09P110668 |
| FGF8 | Fibroblast Growth Factor 8 | Protein Coding | P55075 | 61 | GC10M101770 |
| LGALS3BP | Galectin 3 Binding Protein | Protein Coding | Q08380 | 54 | GC17M078971 |
| FGG | Fibrinogen Gamma Chain | Protein Coding | P02679 | 60 | GC04M154604 |
| HAMP | Hepcidin Antimicrobial Peptide | Protein Coding | P81172 | 55 | GC19P132915 |
| MIRLET7I | MicroRNA Let-7i | RNA Gene |  | 30 | GC12P065553 |
| TYMS | Thymidylate Synthetase | Protein Coding | P04818 | 62 | GC18P000657 |
| APAF1 | Apoptotic Peptidase Activating Factor 1 | Protein Coding | O14727 | 60 | GC12P098645 |
| IL22RA1 | Interleukin 22 Receptor Subunit Alpha 1 | Protein Coding | Q8N6P7 | 51 | GC01M024119 |
| LAIR1 | Leukocyte Associated Immunoglobulin Like Receptor 1 | Protein Coding | Q6GTX8 | 52 | GC19M054351 |
| NLRP6 | NLR Family Pyrin Domain Containing 6 | Protein Coding | P59044 | 48 | GC11P000278 |
| PGLYRP4 | Peptidoglycan Recognition Protein 4 | Protein Coding | Q96LB8 | 46 | GC01M165007 |
| CD177 | CD177 Molecule | Protein Coding | Q8N6Q3 | 51 | GC19P043353 |
| NLRC3 | NLR Family CARD Domain Containing 3 | Protein Coding | Q7RTR2 | 45 | GC16M003539 |
| DYNC1H1 | Dynein Cytoplasmic 1 Heavy Chain 1 | Protein Coding | Q14204 | 57 | GC14P118101 |
| RETN | Resistin | Protein Coding | Q9HD89 | 54 | GC19P007669 |
| PLA2G4A | Phospholipase A2 Group IVA | Protein Coding | P47712 | 61 | GC01P186798 |
| MIR100 | MicroRNA 100 | RNA Gene |  | 30 | GC11M122152 |
| SMIM30 | Small Integral Membrane Protein 30 | Protein Coding | A4D0T7 | 28 | GC07M113117 |
| CLEC4C | C-Type Lectin Domain Family 4 Member C | Protein Coding | Q8WTT0 | 45 | GC12M032942 |
| MIR9-1 | MicroRNA 9-1 | RNA Gene |  | 30 | GC01M156420 |
| MIR26B | MicroRNA 26b | RNA Gene |  | 31 | GC02P218402 |
| PDYN | Prodynorphin | Protein Coding | P01213 | 57 | GC20M001978 |
| MIR197 | MicroRNA 197 | RNA Gene |  | 28 | GC01P109598 |
| REN | Renin | Protein Coding | P00797 | 61 | GC01M204154 |
| TJP2 | Tight Junction Protein 2 | Protein Coding | Q9UDY2 | 57 | GC09P069121 |
| SEPSECS | Sep (O-Phosphoserine) TRNA:Sec (Selenocysteine) TRNA Synthase | Protein Coding | Q9HD40 | 55 | GC04M025121 |
| APRT | Adenine Phosphoribosyltransferase | Protein Coding | P07741 | 59 | GC16M088810 |
| DLAT | Dihydrolipoamide S-Acetyltransferase | Protein Coding | P10515 | 58 | GC11P112736 |
| S100A7 | S100 Calcium Binding Protein A7 | Protein Coding | P31151 | 51 | GC01M153457 |
| SERPINC1 | Serpin Family C Member 1 | Protein Coding | P01008 | 62 | GC01M175056 |
| ERCC6 | ERCC Excision Repair 6, Chromatin Remodeling Factor | Protein Coding | Q03468 | 59 | GC10M049454 |
| IL3RA | Interleukin 3 Receptor Subunit Alpha | Protein Coding | P26951 | 56 | GC0XP001336 |
| UMOD | Uromodulin | Protein Coding | P07911 | 55 | GC16M027663 |
| CXCL6 | C-X-C Motif Chemokine Ligand 6 | Protein Coding | P80162 | 49 | GC04P073837 |
| RANBP2 | RAN Binding Protein 2 | Protein Coding | P49792 | 56 | GC02P108719 |
| BANF1 | Barrier To Autointegration Nuclear Assembly Factor 1 | Protein Coding | O75531 | 55 | GC11P066002 |
| SP100 | SP100 Nuclear Antigen | Protein Coding | P23497 | 52 | GC02P230415 |
| CD200R1 | CD200 Receptor 1 | Protein Coding | Q8TD46 | 51 | GC03M112921 |
| MIR15B | MicroRNA 15b | RNA Gene |  | 29 | GC03P160404 |
| TPMT | Thiopurine S-Methyltransferase | Protein Coding | P51580 | 59 | GC06M018128 |
| IFNK | Interferon Kappa | Protein Coding | Q9P0W0 | 47 | GC09P027514 |
| MTR | 5-Methyltetrahydrofolate-Homocysteine Methyltransferase | Protein Coding | Q99707 | 60 | GC01P236795 |
| DLL4 | Delta Like Canonical Notch Ligand 4 | Protein Coding | Q9NR61 | 60 | GC15P040929 |
| MIR193A | MicroRNA 193a | RNA Gene |  | 29 | GC17P031559 |
| SPIB | Spi-B Transcription Factor | Protein Coding | Q01892 | 48 | GC19P050418 |
| ATG7 | Autophagy Related 7 | Protein Coding | O95352 | 55 | GC03P024229 |
| HCP5 | HLA Complex P5 | RNA Gene | Q6MZN7 | 37 | GC06P031400 |
| GLA | Galactosidase Alpha | Protein Coding | P06280 | 62 | GC0XM101393 |
| FOXP1 | Forkhead Box P1 | Protein Coding | Q9H334 | 57 | GC03M070954 |
| MIR200C | MicroRNA 200c | RNA Gene |  | 30 | GC12P046792 |
| BLOC1S6 | Biogenesis Of Lysosomal Organelles Complex 1 Subunit 6 | Protein Coding | Q9UL45 | 50 | GC15P175260 |
| LRRK2 | Leucine Rich Repeat Kinase 2 | Protein Coding | Q5S007 | 62 | GC12P040196 |
| MEG8 | Maternally Expressed 8, Small Nucleolar RNA Host Gene | RNA Gene |  | 28 | GC14P119043 |
| MIR212 | MicroRNA 212 | RNA Gene |  | 30 | GC17M002050 |
| PRKCA | Protein Kinase C Alpha | Protein Coding | P17252 | 64 | GC17P066302 |
| HNRNPA2B1 | Heterogeneous Nuclear Ribonucleoprotein A2/B1 | Protein Coding | P22626 | 59 | GC07M026174 |
| HTN3 | Histatin 3 | Protein Coding | P15516 | 43 | GC04P070028 |
| YY1 | YY1 Transcription Factor | Protein Coding | P25490 | 61 | GC14P100238 |
| CHAT | Choline O-Acetyltransferase | Protein Coding | P28329 | 61 | GC10P049609 |
| EEF1A1 | Eukaryotic Translation Elongation Factor 1 Alpha 1 | Protein Coding | P68104 | 56 | GC06M100852 |
| TAT | Tyrosine Aminotransferase | Protein Coding | P17735 | 57 | GC16M071565 |
| CFHR5 | Complement Factor H Related 5 | Protein Coding | Q9BXR6 | 51 | GC01P196977 |
| LPO | Lactoperoxidase | Protein Coding | P22079 | 52 | GC17P058218 |
| MIR151A | MicroRNA 151a | RNA Gene |  | 28 | GC08M140980 |
| FGFR2 | Fibroblast Growth Factor Receptor 2 | Protein Coding | P21802 | 68 | GC10M121478 |
| ULBP1 | UL16 Binding Protein 1 | Protein Coding | Q9BZM6 | 50 | GC06P149963 |
| UFD1 | Ubiquitin Recognition Factor In ER Associated Degradation 1 | Protein Coding | Q92890 | 56 | GC22M020427 |
| KIR2DL5A | Killer Cell Immunoglobulin Like Receptor, Two Ig Domains And Long Cytoplasmic Tail 5A | Protein Coding | Q8N109 | 30 | GC19MR00046 |
| OLR1 | Oxidized Low Density Lipoprotein Receptor 1 | Protein Coding | P78380 | 55 | GC12M033112 |
| SYT14 | Synaptotagmin 14 | Protein Coding | Q8NB59 | 48 | GC01P209900 |
| SKP2 | S-Phase Kinase Associated Protein 2 | Protein Coding | Q13309 | 58 | GC05P036151 |
| SERPINA3 | Serpin Family A Member 3 | Protein Coding | P01011 | 56 | GC14P094612 |
| CAMK4 | Calcium/Calmodulin Dependent Protein Kinase IV | Protein Coding | Q16566 | 59 | GC05P111223 |
| ENTPD1 | Ectonucleoside Triphosphate Diphosphohydrolase 1 | Protein Coding | P49961 | 62 | GC10P116239 |
| PEX10 | Peroxisomal Biogenesis Factor 10 | Protein Coding | O60683 | 53 | GC01M002403 |
| LOC130064510 | ATAC-STARR-Seq Lymphoblastoid Silent Region 10661 | Functional Element |  | 8 | GC19P139800 |
| MIR30B | MicroRNA 30b | RNA Gene |  | 30 | GC08M134800 |
| GRM1 | Glutamate Metabotropic Receptor 1 | Protein Coding | Q13255 | 63 | GC06P163537 |
| MYCN | MYCN Proto-Oncogene, BHLH Transcription Factor | Protein Coding | P04198 | 59 | GC02P015972 |
| CD300LB | CD300 Molecule Like Family Member B | Protein Coding | A8K4G0 | 45 | GC17M074521 |
| SAA1 | Serum Amyloid A1 | Protein Coding | P0DJI8 | 51 | GC11P019454 |
| EDN1 | Endothelin 1 | Protein Coding | P05305 | 61 | GC06P012497 |
| KIF19 | Kinesin Family Member 19 | Protein Coding | Q2TAC6 | 44 | GC17P074326 |
| ANXA2 | Annexin A2 | Protein Coding | P07355 | 59 | GC15M060347 |
| MIR501 | MicroRNA 501 | RNA Gene |  | 26 | GC0XP059334 |
| PLAU | Plasminogen Activator, Urokinase | Protein Coding | P00749 | 64 | GC10P073909 |
| KMT2C | Lysine Methyltransferase 2C | Protein Coding | Q8NEZ4 | 55 | GC07M152134 |
| PRKCE | Protein Kinase C Epsilon | Protein Coding | Q02156 | 61 | GC02P045651 |
| HDAC9 | Histone Deacetylase 9 | Protein Coding | Q9UKV0 | 63 | GC07P018086 |
| FGF23 | Fibroblast Growth Factor 23 | Protein Coding | Q9GZV9 | 58 | GC12M004368 |
| POU2AF1 | POU Class 2 Homeobox Associating Factor 1 | Protein Coding | Q16633 | 47 | GC11M111352 |
| POLR3B | RNA Polymerase III Subunit B | Protein Coding | Q9NW08 | 55 | GC12P106357 |
| MIR224 | MicroRNA 224 | RNA Gene |  | 26 | GC0XM151958 |
| RARRES2 | Retinoic Acid Receptor Responder 2 | Protein Coding | Q99969 | 51 | GC07M150333 |
| MIR18B | MicroRNA 18b | RNA Gene |  | 24 | GC0XM134525 |
| PTPN3 | Protein Tyrosine Phosphatase Non-Receptor Type 3 | Protein Coding | P26045 | 55 | GC09M109375 |
| MIR362 | MicroRNA 362 | RNA Gene |  | 24 | GC0XP059333 |
| UCP2 | Uncoupling Protein 2 | Protein Coding | P55851 | 58 | GC11M073974 |
| MLPH | Melanophilin | Protein Coding | Q9BV36 | 53 | GC02P237485 |
| IL1RL2 | Interleukin 1 Receptor Like 2 | Protein Coding | Q9HB29 | 54 | GC02P102186 |
| SDC1 | Syndecan 1 | Protein Coding | P18827 | 55 | GC02M020200 |
| MIAT | Myocardial Infarction Associated Transcript | RNA Gene |  | 34 | GC22P026646 |
| SELENON | Selenoprotein N | Protein Coding | Q9NZV5 | 50 | GC01P025800 |
| MIR96 | MicroRNA 96 | RNA Gene |  | 30 | GC07M129774 |
| CTSS | Cathepsin S | Protein Coding | P25774 | 58 | GC01M150730 |
| ABCD1 | ATP Binding Cassette Subfamily D Member 1 | Protein Coding | P33897 | 59 | GC0XP153724 |
| LOC106804612 | Hemoglobin Subunit Alpha 2 Recombination Region | Functional Element |  | 11 | GC16P107838 |
| GNAQ | G Protein Subunit Alpha Q | Protein Coding | P50148 | 61 | GC09M077716 |
| TNFSF8 | TNF Superfamily Member 8 | Protein Coding | P32971 | 46 | GC09M114893 |
| TNFRSF25 | TNF Receptor Superfamily Member 25 | Protein Coding | Q93038 | 55 | GC01M006460 |
| PTGDR2 | Prostaglandin D2 Receptor 2 | Protein Coding | Q9Y5Y4 | 54 | GC11M060850 |
| ARSA | Arylsulfatase A | Protein Coding | P15289 | 59 | GC22M050622 |
| SHOC2 | SHOC2 Leucine Rich Repeat Scaffold Protein | Protein Coding | Q9UQ13 | 53 | GC10P110919 |
| OASL | 2'-5'-Oligoadenylate Synthetase Like | Protein Coding | Q15646 | 50 | GC12M124264 |
| STX3 | Syntaxin 3 | Protein Coding | Q13277 | 54 | GC11P059713 |
| IL13RA1 | Interleukin 13 Receptor Subunit Alpha 1 | Protein Coding | P78552 | 53 | GC0XP118727 |
| GH1 | Growth Hormone 1 | Protein Coding | P01241 | 55 | GC17M063917 |
| CHIA | Chitinase Acidic | Protein Coding | Q9BZP6 | 53 | GC01P111295 |
| CASP4 | Caspase 4 | Protein Coding | P49662 | 58 | GC11M104942 |
| TRV-AAC1-4 | TRNA-Val (Anticodon AAC) 1-4 | RNA Gene |  | 9 | GC05M181218 |
| AGT | Angiotensinogen | Protein Coding | P01019 | 62 | GC01M230690 |
| GP6 | Glycoprotein VI Platelet | Protein Coding | Q9HCN6 | 58 | GC19M055013 |
| IL17D | Interleukin 17D | Protein Coding | Q8TAD2 | 48 | GC13P020884 |
| KLRC4 | Killer Cell Lectin Like Receptor C4 | Protein Coding | O43908 | 47 | GC12M033114 |
| ANO10 | Anoctamin 10 | Protein Coding | Q9NW15 | 50 | GC03M043355 |
| GAD2 | Glutamate Decarboxylase 2 | Protein Coding | Q05329 | 56 | GC10P026216 |
| IL9R | Interleukin 9 Receptor | Protein Coding | Q01113 | 47 | GC0XP155997 |
| MIR211 | MicroRNA 211 | RNA Gene |  | 30 | GC15M031065 |
| CXCL16 | C-X-C Motif Chemokine Ligand 16 | Protein Coding | Q9H2A7 | 50 | GC17M004733 |
| PMPCA | Peptidase, Mitochondrial Processing Subunit Alpha | Protein Coding | Q10713 | 55 | GC09P136410 |
| DGUOK | Deoxyguanosine Kinase | Protein Coding | Q16854 | 55 | GC02P073926 |
| DEFB103A | Defensin Beta 103A | Protein Coding | P81534 | 36 | GC08P007881 |
| KIF5A | Kinesin Family Member 5A | Protein Coding | Q12840 | 57 | GC12P065445 |
| XRCC4 | X-Ray Repair Cross Complementing 4 | Protein Coding | Q13426 | 56 | GC05P083077 |
| KEAP1 | Kelch Like ECH Associated Protein 1 | Protein Coding | Q14145 | 61 | GC19M010486 |
| PRDX1 | Peroxiredoxin 1 | Protein Coding | Q06830 | 62 | GC01M045828 |
| LY75 | Lymphocyte Antigen 75 | Protein Coding | O60449 | 50 | GC02M159803 |
| ASAH1 | N-Acylsphingosine Amidohydrolase 1 | Protein Coding | Q13510 | 62 | GC08M018055 |
| CDK2 | Cyclin Dependent Kinase 2 | Protein Coding | P24941 | 63 | GC12P055966 |
| HNF4A | Hepatocyte Nuclear Factor 4 Alpha | Protein Coding | P41235 | 62 | GC20P044355 |
| ORM1 | Orosomucoid 1 | Protein Coding | P02763 | 52 | GC09P114323 |
| HLA-DMB | Major Histocompatibility Complex, Class II, DM Beta | Protein Coding | P28068 | 51 | GC06M032934 |
| DEFB103B | Defensin Beta 103B | Protein Coding | P81534 | 37 | GC08M007430 |
| CCR8 | C-C Motif Chemokine Receptor 8 | Protein Coding | P51685 | 54 | GC03P041180 |
| APOBEC3F | Apolipoprotein B MRNA Editing Enzyme Catalytic Subunit 3F | Protein Coding | Q8IUX4 | 43 | GC22P039040 |
| DEFA1 | Defensin Alpha 1 | Protein Coding | P59665 | 49 | GC08M006977 |
| TNFRSF10B | TNF Receptor Superfamily Member 10b | Protein Coding | O14763 | 62 | GC08M023020 |
| SYP | Synaptophysin | Protein Coding | P08247 | 59 | GC0XM049187 |
| FLG2 | Filaggrin 2 | Protein Coding | Q5D862 | 48 | GC01M164988 |
| NAMPT | Nicotinamide Phosphoribosyltransferase | Protein Coding | P43490 | 60 | GC07M106248 |
| CA8 | Carbonic Anhydrase 8 | Protein Coding | P35219 | 58 | GC08M060187 |
| CD9 | CD9 Molecule | Protein Coding | P21926 | 56 | GC12P046724 |
| HOXB13 | Homeobox B13 | Protein Coding | Q92826 | 54 | GC17M089707 |
| CDKN1C | Cyclin Dependent Kinase Inhibitor 1C | Protein Coding | P49918 | 58 | GC11M013326 |
| RGS1 | Regulator Of G Protein Signaling 1 | Protein Coding | Q08116 | 50 | GC01P192575 |
| TPP1 | Tripeptidyl Peptidase 1 | Protein Coding | O14773 | 58 | GC11M013521 |
| TAC1 | Tachykinin Precursor 1 | Protein Coding | P20366 | 54 | GC07P097734 |
| TMEM127 | Transmembrane Protein 127 | Protein Coding | O75204 | 50 | GC02M096248 |
| EDAR | Ectodysplasin A Receptor | Protein Coding | Q9UNE0 | 53 | GC02M108894 |
| GAL | Galanin And GMAP Prepropeptide | Protein Coding | P22466 | 57 | GC11P099196 |
| PRICKLE1 | Prickle Planar Cell Polarity Protein 1 | Protein Coding | Q96MT3 | 56 | GC12M042456 |
| MYH14 | Myosin Heavy Chain 14 | Protein Coding | Q7Z406 | 58 | GC19P133465 |
| CNTF | Ciliary Neurotrophic Factor | Protein Coding | P26441 | 54 | GC11P058622 |
| BCL11A | BCL11 Transcription Factor A | Protein Coding | Q9H165 | 56 | GC02M060451 |
| IFNE | Interferon Epsilon | Protein Coding | Q86WN2 | 46 | GC09M021480 |
| DEFA5 | Defensin Alpha 5 | Protein Coding | Q01523 | 48 | GC08M007057 |
| SOX9 | SRY-Box Transcription Factor 9 | Protein Coding | P48436 | 58 | GC17P072121 |
| CHEK1 | Checkpoint Kinase 1 | Protein Coding | O14757 | 65 | GC11P125625 |
| TARBP2 | TARBP2 Subunit Of RISC Loading Complex | Protein Coding | Q15633 | 49 | GC12P065221 |
| IFI35 | Interferon Induced Protein 35 | Protein Coding | P80217 | 47 | GC17P043006 |
| CCL3L1 | C-C Motif Chemokine Ligand 3 Like 1 | Protein Coding | P16619 | 37 | GC17Mj00250 |
| CFLAR | CASP8 And FADD Like Apoptosis Regulator | Protein Coding | O15519 | 56 | GC02P204216 |
| SDHAF2 | Succinate Dehydrogenase Complex Assembly Factor 2 | Protein Coding | Q9NX18 | 54 | GC11P061430 |
| SREBF1 | Sterol Regulatory Element Binding Transcription Factor 1 | Protein Coding | P36956 | 61 | GC17M017810 |
| MST1R | Macrophage Stimulating 1 Receptor | Protein Coding | Q04912 | 62 | GC03M054300 |
| TWNK | Twinkle MtDNA Helicase | Protein Coding | Q96RR1 | 54 | GC10P116450 |
| GPR183 | G Protein-Coupled Receptor 183 | Protein Coding | P32249 | 48 | GC13M099479 |
| MIR31 | MicroRNA 31 | RNA Gene |  | 29 | GC09M022239 |
| DIAPH1 | Diaphanous Related Formin 1 | Protein Coding | O60610 | 59 | GC05M141516 |
| TF | Transferrin | Protein Coding | P02787 | 63 | GC03P140959 |
| ALPI | Alkaline Phosphatase, Intestinal | Protein Coding | P09923 | 59 | GC02P232456 |
| MIR33A | MicroRNA 33a | RNA Gene |  | 28 | GC22P041900 |
| PRKG1 | Protein Kinase CGMP-Dependent 1 | Protein Coding | Q13976 | 63 | GC10P051005 |
| XBP1 | X-Box Binding Protein 1 | Protein Coding | P17861 | 59 | GC22M028794 |
| CYP19A1 | Cytochrome P450 Family 19 Subfamily A Member 1 | Protein Coding | P11511 | 61 | GC15M051208 |
| MMP8 | Matrix Metallopeptidase 8 | Protein Coding | P22894 | 60 | GC11M133497 |
| NLRX1 | NLR Family Member X1 | Protein Coding | Q86UT6 | 50 | GC11P119166 |
| CYP7B1 | Cytochrome P450 Family 7 Subfamily B Member 1 | Protein Coding | O75881 | 58 | GC08M064587 |
| ADAMTS3 | ADAM Metallopeptidase With Thrombospondin Type 1 Motif 3 | Protein Coding | O15072 | 52 | GC04M072280 |
| CYCS | Cytochrome C, Somatic | Protein Coding | P99999 | 61 | GC07M025118 |
| COL4A3 | Collagen Type IV Alpha 3 Chain | Protein Coding | Q01955 | 58 | GC02P227164 |
| RUNX3 | RUNX Family Transcription Factor 3 | Protein Coding | Q13761 | 55 | GC01M024899 |
| JUND | JunD Proto-Oncogene, AP-1 Transcription Factor Subunit | Protein Coding | P17535 | 54 | GC19M018279 |
| SMAD7 | SMAD Family Member 7 | Protein Coding | O15105 | 56 | GC18M048919 |
| CCNA2 | Cyclin A2 | Protein Coding | P20248 | 58 | GC04M121816 |
| MIR483 | MicroRNA 483 | RNA Gene |  | 29 | GC11M013306 |
| PGF | Placental Growth Factor | Protein Coding | P49763 | 56 | GC14M074941 |
| TTPA | Alpha Tocopherol Transfer Protein | Protein Coding | P49638 | 54 | GC08M063048 |
| CD207 | CD207 Molecule | Protein Coding | Q9UJ71 | 50 | GC02M070830 |
| NUP214 | Nucleoporin 214 | Protein Coding | P35658 | 57 | GC09P131125 |
| KCNJ11 | Potassium Inwardly Rectifying Channel Subfamily J Member 11 | Protein Coding | Q14654 | 58 | GC11M018031 |
| FZD6 | Frizzled Class Receptor 6 | Protein Coding | O60353 | 57 | GC08P103298 |
| ELN | Elastin | Protein Coding | P15502 | 55 | GC07P074027 |
| GATA6 | GATA Binding Protein 6 | Protein Coding | Q92908 | 59 | GC18P022169 |
| CLDN5 | Claudin 5 | Protein Coding | O00501 | 53 | GC22M019523 |
| TRADD | TNFRSF1A Associated Via Death Domain | Protein Coding | Q15628 | 55 | GC16M067154 |
| MC1R | Melanocortin 1 Receptor | Protein Coding | Q01726 | 59 | GC16P089912 |
| LPL | Lipoprotein Lipase | Protein Coding | P06858 | 63 | GC08P019901 |
| PRKACB | Protein Kinase CAMP-Activated Catalytic Subunit Beta | Protein Coding | P22694 | 61 | GC01P084078 |
| CD180 | CD180 Molecule | Protein Coding | Q99467 | 51 | GC05M067181 |
| CCR10 | C-C Motif Chemokine Receptor 10 | Protein Coding | P46092 | 48 | GC17M042678 |
| OAS3 | 2'-5'-Oligoadenylate Synthetase 3 | Protein Coding | Q9Y6K5 | 51 | GC12P112938 |
| AMACR | Alpha-Methylacyl-CoA Racemase | Protein Coding | Q9UHK6 | 58 | GC05M033986 |
| RBP3 | Retinol Binding Protein 3 | Protein Coding | P10745 | 54 | GC10P047348 |
| TJP1 | Tight Junction Protein 1 | Protein Coding | Q07157 | 58 | GC15M029699 |
| GBP5 | Guanylate Binding Protein 5 | Protein Coding | Q96PP8 | 47 | GC01M089259 |
| PLA2G7 | Phospholipase A2 Group VII | Protein Coding | Q13093 | 63 | GC06M046704 |
| NTRK2 | Neurotrophic Receptor Tyrosine Kinase 2 | Protein Coding | Q16620 | 66 | GC09P084668 |
| DEFA6 | Defensin Alpha 6 | Protein Coding | Q01524 | 43 | GC08M006924 |
| MAP2K3 | Mitogen-Activated Protein Kinase Kinase 3 | Protein Coding | P46734 | 62 | GC17P133125 |
| PMP22 | Peripheral Myelin Protein 22 | Protein Coding | Q01453 | 55 | GC17M015229 |
| AGTR1 | Angiotensin II Receptor Type 1 | Protein Coding | P30556 | 65 | GC03P148697 |
| TAGAP | T Cell Activation RhoGTPase Activating Protein | Protein Coding | Q8N103 | 50 | GC06M159034 |
| PIGC | Phosphatidylinositol Glycan Anchor Biosynthesis Class C | Protein Coding | Q92535 | 50 | GC01M172339 |
| EPO | Erythropoietin | Protein Coding | P01588 | 55 | GC07P100720 |
| MIR152 | MicroRNA 152 | RNA Gene |  | 30 | GC17M048037 |
| NUP98 | Nucleoporin 98 And 96 Precursor | Protein Coding | P52948 | 58 | GC11M003671 |
| DDOST | Dolichyl-Diphosphooligosaccharide--Protein Glycosyltransferase Non-Catalytic Subunit | Protein Coding | P39656 | 57 | GC01M020651 |
| PIBF1 | Progesterone Immunomodulatory Binding Factor 1 | Protein Coding | Q8WXW3 | 54 | GC13P072782 |
| ABCG2 | ATP Binding Cassette Subfamily G Member 2 (JR Blood Group) | Protein Coding | Q9UNQ0 | 62 | GC04M088090 |
| TFEB | Transcription Factor EB | Protein Coding | P19484 | 55 | GC06M100519 |
| TGM2 | Transglutaminase 2 | Protein Coding | P21980 | 60 | GC20M038127 |
| CSN1S1 | Casein Alpha S1 | Protein Coding | P47710 | 49 | GC04P070063 |
| EBAG9 | Estrogen Receptor Binding Site Associated Antigen 9 | Protein Coding | O00559 | 47 | GC08P109554 |
| TRIM56 | Tripartite Motif Containing 56 | Protein Coding | Q9BRZ2 | 44 | GC07P101085 |
| ENO1 | Enolase 1 | Protein Coding | P06733 | 61 | GC01M008861 |
| F11R | F11 Receptor | Protein Coding | Q9Y624 | 54 | GC01M160995 |
| FCRL5 | Fc Receptor Like 5 | Protein Coding | Q96RD9 | 50 | GC01M165238 |
| NTHL1 | Nth Like DNA Glycosylase 1 | Protein Coding | P78549 | 55 | GC16M026904 |
| CLDN1 | Claudin 1 | Protein Coding | O95832 | 59 | GC03M190305 |
| AGRN | Agrin | Protein Coding | O00468 | 58 | GC01P001020 |
| SGK1 | Serum/Glucocorticoid Regulated Kinase 1 | Protein Coding | O00141 | 62 | GC06M134169 |
| KAT2B | Lysine Acetyltransferase 2B | Protein Coding | Q92831 | 61 | GC03P024437 |
| MAPKAPK2 | MAPK Activated Protein Kinase 2 | Protein Coding | P49137 | 60 | GC01P206684 |
| LDLR | Low Density Lipoprotein Receptor | Protein Coding | P01130 | 64 | GC19P132305 |
| MIR144 | MicroRNA 144 | RNA Gene |  | 26 | GC17M088846 |
| ROCK2 | Rho Associated Coiled-Coil Containing Protein Kinase 2 | Protein Coding | O75116 | 58 | GC02M011539 |
| NSD2 | Nuclear Receptor Binding SET Domain Protein 2 | Protein Coding | O96028 | 57 | GC04P005902 |
| ATG16L1 | Autophagy Related 16 Like 1 | Protein Coding | Q676U5 | 55 | GC02P233858 |
| TIMP2 | TIMP Metallopeptidase Inhibitor 2 | Protein Coding | P16035 | 54 | GC17M078852 |
| CMKLR1 | Chemerin Chemokine-Like Receptor 1 | Protein Coding | Q99788 | 50 | GC12M108288 |
| MIR296 | MicroRNA 296 | RNA Gene |  | 26 | GC20M058817 |
| NR1H2 | Nuclear Receptor Subfamily 1 Group H Member 2 | Protein Coding | P55055 | 58 | GC19P050329 |
| GGH | Gamma-Glutamyl Hydrolase | Protein Coding | Q92820 | 55 | GC08M063014 |
| MIR27B | MicroRNA 27b | RNA Gene |  | 30 | GC09P103712 |
| FSCN1 | Fascin Actin-Bundling Protein 1 | Protein Coding | Q16658 | 56 | GC07P005592 |
| ATP2B3 | ATPase Plasma Membrane Ca2+ Transporting 3 | Protein Coding | Q16720 | 58 | GC0XP153517 |
| LRP4 | LDL Receptor Related Protein 4 | Protein Coding | O75096 | 56 | GC11M132457 |
| ACP1 | Acid Phosphatase 1 | Protein Coding | P24666 | 55 | GC02P001030 |
| MED12 | Mediator Complex Subunit 12 | Protein Coding | Q93074 | 56 | GC0XP071118 |
| F9 | Coagulation Factor IX | Protein Coding | P00740 | 60 | GC0XP139530 |
| LDHA | Lactate Dehydrogenase A | Protein Coding | P00338 | 63 | GC11P018394 |
| TNFRSF12A | TNF Receptor Superfamily Member 12A | Protein Coding | Q9NP84 | 54 | GC16P003018 |
| TNFRSF21 | TNF Receptor Superfamily Member 21 | Protein Coding | O75509 | 57 | GC06M047231 |
| CCL15-CCL14 | CCL15-CCL14 Readthrough (NMD Candidate) | RNA Gene |  | 18 | GC17M035983 |
| RREB1 | Ras Responsive Element Binding Protein 1 | Protein Coding | Q92766 | 52 | GC06P007107 |
| DLL1 | Delta Like Canonical Notch Ligand 1 | Protein Coding | O00548 | 57 | GC06M170282 |
| AFP | Alpha Fetoprotein | Protein Coding | P02771 | 59 | GC04P073431 |
| CD300LD | CD300 Molecule Like Family Member D | Protein Coding | Q6UXZ3 | 40 | GC17M090309 |
| NGFR | Nerve Growth Factor Receptor | Protein Coding | P08138 | 60 | GC17P049495 |
| CNR1 | Cannabinoid Receptor 1 | Protein Coding | P21554 | 59 | GC06M088139 |
| FLVCR1 | FLVCR Choline And Heme Transporter 1 | Protein Coding | Q9Y5Y0 | 51 | GC01P212858 |
| L1CAM | L1 Cell Adhesion Molecule | Protein Coding | P32004 | 60 | GC0XM153864 |
| KRT14 | Keratin 14 | Protein Coding | P02533 | 59 | GC17M041582 |
| ALDH18A1 | Aldehyde Dehydrogenase 18 Family Member A1 | Protein Coding | P54886 | 58 | GC10M095605 |
| ATG12 | Autophagy Related 12 | Protein Coding | O94817 | 52 | GC05M115828 |
| GALNT14 | Polypeptide N-Acetylgalactosaminyltransferase 14 | Protein Coding | Q96FL9 | 50 | GC02M030888 |
| MTRR | 5-Methyltetrahydrofolate-Homocysteine Methyltransferase Reductase | Protein Coding | Q9UBK8 | 56 | GC05P007851 |
| ATP1A1 | ATPase Na+/K+ Transporting Subunit Alpha 1 | Protein Coding | P05023 | 62 | GC01P116372 |
| DHX15 | DEAH-Box Helicase 15 | Protein Coding | O43143 | 51 | GC04M024519 |
| SPRED1 | Sprouty Related EVH1 Domain Containing 1 | Protein Coding | Q7Z699 | 54 | GC15P038252 |
| RO60 | Ro60, Y RNA Binding Protein | Protein Coding | P10155 | 48 | GC01P193059 |
| KRT18 | Keratin 18 | Protein Coding | P05783 | 62 | GC12P052948 |
| CNP | 2',3'-Cyclic Nucleotide 3' Phosphodiesterase | Protein Coding | P09543 | 56 | GC17P041966 |
| COL18A1 | Collagen Type XVIII Alpha 1 Chain | Protein Coding | P39060 | 58 | GC21P045405 |
| MIR133B | MicroRNA 133b | RNA Gene |  | 31 | GC06P052148 |
| CLCF1 | Cardiotrophin Like Cytokine Factor 1 | Protein Coding | Q9UBD9 | 54 | GC11M067364 |
| ARNT | Aryl Hydrocarbon Receptor Nuclear Translocator | Protein Coding | P27540 | 58 | GC01M150809 |
| CEP290 | Centrosomal Protein 290 | Protein Coding | O15078 | 52 | GC12M088049 |
| GLUL | Glutamate-Ammonia Ligase | Protein Coding | P15104 | 61 | GC01M182378 |
| TK2 | Thymidine Kinase 2 | Protein Coding | O00142 | 54 | GC16M066508 |
| EIF4G1 | Eukaryotic Translation Initiation Factor 4 Gamma 1 | Protein Coding | Q04637 | 59 | GC03P184314 |
| TYMP | Thymidine Phosphorylase | Protein Coding | P19971 | 59 | GC22M050525 |
| CALM1 | Calmodulin 1 | Protein Coding | P0DP23 | 58 | GC14P090396 |
| KDM5C | Lysine Demethylase 5C | Protein Coding | P41229 | 59 | GC0XM053176 |
| REG3G | Regenerating Family Member 3 Gamma | Protein Coding | Q6UW15 | 44 | GC02P079025 |
| B4GALT1 | Beta-1,4-Galactosyltransferase 1 | Protein Coding | P15291 | 58 | GC09M033100 |
| PTS | 6-Pyruvoyltetrahydropterin Synthase | Protein Coding | Q03393 | 58 | GC11P112226 |
| RXRA | Retinoid X Receptor Alpha | Protein Coding | P19793 | 61 | GC09P134317 |
| DNAH14 | Dynein Axonemal Heavy Chain 14 | Protein Coding | Q0VDD8 | 43 | GC01P224896 |
| HADHA | Hydroxyacyl-CoA Dehydrogenase Trifunctional Multienzyme Complex Subunit Alpha | Protein Coding | P40939 | 59 | GC02M026190 |
| SLC19A1 | Solute Carrier Family 19 Member 1 | Protein Coding | P41440 | 59 | GC21M045493 |
| NPY | Neuropeptide Y | Protein Coding | P01303 | 57 | GC07P024449 |
| IDO2 | Indoleamine 2,3-Dioxygenase 2 | Protein Coding | Q6ZQW0 | 52 | GC08P040951 |
| TRIM38 | Tripartite Motif Containing 38 | Protein Coding | O00635 | 51 | GC06P025962 |
| PCSK1 | Proprotein Convertase Subtilisin/Kexin Type 1 | Protein Coding | P29120 | 62 | GC05M096391 |
| TRIM14 | Tripartite Motif Containing 14 | Protein Coding | Q14142 | 46 | GC09M098035 |
| GLI1 | GLI Family Zinc Finger 1 | Protein Coding | P08151 | 61 | GC12P065433 |
| BSCL2 | BSCL2 Lipid Droplet Biogenesis Associated, Seipin | Protein Coding | Q96G97 | 55 | GC11M132685 |
| CDK5 | Cyclin Dependent Kinase 5 | Protein Coding | Q00535 | 65 | GC07M151053 |
| PSD | Pleckstrin And Sec7 Domain Containing | Protein Coding | A5PKW4 | 50 | GC10M102403 |
| RNASE2 | Ribonuclease A Family Member 2 | Protein Coding | P10153 | 52 | GC14P051512 |
| GPT | Glutamic--Pyruvic Transaminase | Protein Coding | P24298 | 54 | GC08P144502 |
| REG3A | Regenerating Family Member 3 Alpha | Protein Coding | Q06141 | 49 | GC02M079157 |
| BPIFB1 | BPI Fold Containing Family B Member 1 | Protein Coding | Q8TDL5 | 45 | GC20P033273 |
| DEFB4B | Defensin Beta 4B | Protein Coding | O15263 | 37 | GC08M007414 |
| TYRP1 | Tyrosinase Related Protein 1 | Protein Coding | P17643 | 58 | GC09P012683 |
| EGR2 | Early Growth Response 2 | Protein Coding | P11161 | 58 | GC10M062811 |
| IGHG2 | Immunoglobulin Heavy Constant Gamma 2 (G2m Marker) | Protein Coding | P01859 | 39 | GC14M122254 |
| MRPS34 | Mitochondrial Ribosomal Protein S34 | Protein Coding | P82930 | 48 | GC16M001771 |
| KRT17 | Keratin 17 | Protein Coding | Q04695 | 58 | GC17M041619 |
| CDH5 | Cadherin 5 | Protein Coding | P33151 | 59 | GC16P066366 |
| SEC24C | SEC24 Homolog C, COPII Coat Complex Component | Protein Coding | P53992 | 56 | GC10P073744 |
| ORMDL3 | ORMDL Sphingolipid Biosynthesis Regulator 3 | Protein Coding | Q8N138 | 49 | GC17M039921 |
| IL20 | Interleukin 20 | Protein Coding | Q9NYY1 | 51 | GC01P206866 |
| C1orf105 | Chromosome 1 Open Reading Frame 105 | Protein Coding | O95561 | 39 | GC01P172454 |
| LOC126862505 | BRD4-Independent Group 4 Enhancer GRCh37_chr17:11572478-11573677 | Functional Element |  | 5 | GC17P135982 |
| PIN1 | Peptidylprolyl Cis/Trans Isomerase, NIMA-Interacting 1 | Protein Coding | Q13526 | 60 | GC19P009835 |
| GNPTAB | N-Acetylglucosamine-1-Phosphate Transferase Subunits Alpha And Beta | Protein Coding | Q3T906 | 54 | GC12M101745 |
| FGF7 | Fibroblast Growth Factor 7 | Protein Coding | P21781 | 56 | GC15P049423 |
| PTPRN | Protein Tyrosine Phosphatase Receptor Type N | Protein Coding | Q16849 | 56 | GC02M219289 |
| IL22RA2 | Interleukin 22 Receptor Subunit Alpha 2 | Protein Coding | Q969J5 | 50 | GC06M137143 |
| SHC1 | SHC Adaptor Protein 1 | Protein Coding | P29353 | 57 | GC01M154962 |
| KRT16 | Keratin 16 | Protein Coding | P08779 | 55 | GC17M041609 |
| MRAP | Melanocortin 2 Receptor Accessory Protein | Protein Coding | Q8TCY5 | 47 | GC21P032291 |
| SNORD118 | Small Nucleolar RNA, C/D Box 118 | RNA Gene |  | 28 | GC17M088330 |
| RFX1 | Regulatory Factor X1 | Protein Coding | P22670 | 51 | GC19M013961 |
| PCSK9 | Proprotein Convertase Subtilisin/Kexin Type 9 | Protein Coding | Q8NBP7 | 62 | GC01P055039 |
| NFKBIZ | NFKB Inhibitor Zeta | Protein Coding | Q9BYH8 | 49 | GC03P101827 |
| PSAP | Prosaposin | Protein Coding | P07602 | 61 | GC10M071816 |
| MIR10B | MicroRNA 10b | RNA Gene |  | 31 | GC02P176150 |
| MIR124-1 | MicroRNA 124-1 | RNA Gene |  | 29 | GC08M009903 |
| HMBS | Hydroxymethylbilane Synthase | Protein Coding | P08397 | 57 | GC11P119084 |
| ULK1 | Unc-51 Like Autophagy Activating Kinase 1 | Protein Coding | O75385 | 56 | GC12P131894 |
| KCNN4 | Potassium Calcium-Activated Channel Subfamily N Member 4 | Protein Coding | O15554 | 60 | GC19M101081 |
| TAX1BP1 | Tax1 Binding Protein 1 | Protein Coding | Q86VP1 | 54 | GC07P027739 |
| PTPN1 | Protein Tyrosine Phosphatase Non-Receptor Type 1 | Protein Coding | P18031 | 62 | GC20P050510 |
| RHOG | Ras Homolog Family Member G | Protein Coding | P84095 | 52 | GC11M013377 |
| MIR203A | MicroRNA 203a | RNA Gene |  | 28 | GC14P118123 |
| OSMR | Oncostatin M Receptor | Protein Coding | Q99650 | 57 | GC05P038845 |
| MIR409 | MicroRNA 409 | RNA Gene |  | 28 | GC14P119069 |
| CCAT1 | Colon Cancer Associated Transcript 1 | RNA Gene |  | 22 | GC08M127207 |
| MIR511 | MicroRNA 511 | RNA Gene |  | 25 | GC10P017845 |
| PLAT | Plasminogen Activator, Tissue Type | Protein Coding | P00750 | 62 | GC08M042174 |
| ATP8A2 | ATPase Phospholipid Transporting 8A2 | Protein Coding | Q9NTI2 | 51 | GC13P028060 |
| CCL23 | C-C Motif Chemokine Ligand 23 | Protein Coding | P55773 | 44 | GC17M036013 |
| CCN2 | Cellular Communication Network Factor 2 | Protein Coding | P29279 | 58 | GC06M131948 |
| LRP5 | LDL Receptor Related Protein 5 | Protein Coding | O75197 | 62 | GC11P068298 |
| DLEU2 | Deleted In Lymphocytic Leukemia 2 | RNA Gene |  | 31 | GC13M049913 |
| SUMO1 | Small Ubiquitin Like Modifier 1 | Protein Coding | P63165 | 59 | GC02M202206 |
| XCL1 | X-C Motif Chemokine Ligand 1 | Protein Coding | P47992 | 46 | GC01P168576 |
| CLDN2 | Claudin 2 | Protein Coding | P57739 | 52 | GC0XP106900 |
| FUT2 | Fucosyltransferase 2 (H Blood Group) | Protein Coding | Q10981 | 57 | GC19P048695 |
| TRIM62 | Tripartite Motif Containing 62 | Protein Coding | Q9BVG3 | 43 | GC01M034085 |
| NR4A1 | Nuclear Receptor Subfamily 4 Group A Member 1 | Protein Coding | P22736 | 60 | GC12P052022 |
| CD7 | CD7 Molecule | Protein Coding | P09564 | 52 | GC17M082314 |
| NUP210 | Nucleoporin 210 | Protein Coding | Q8TEM1 | 51 | GC03M027393 |
| CCNB1 | Cyclin B1 | Protein Coding | P14635 | 59 | GC05P069167 |
| ATCAY | ATCAY Kinesin Light Chain Interacting Caytaxin | Protein Coding | Q86WG3 | 50 | GC19P131965 |
| RAD51D | RAD51 Paralog D | Protein Coding | O75771 | 52 | GC17M035092 |
| SOX11 | SRY-Box Transcription Factor 11 | Protein Coding | P35716 | 53 | GC02P005707 |
| FSTL1 | Follistatin Like 1 | Protein Coding | Q12841 | 53 | GC03M120392 |
| NPHS1 | NPHS1 Adhesion Molecule, Nephrin | Protein Coding | O60500 | 59 | GC19M035825 |
| NOX4 | NADPH Oxidase 4 | Protein Coding | Q9NPH5 | 55 | GC11M089324 |
| CFL1 | Cofilin 1 | Protein Coding | P23528 | 58 | GC11M065823 |
| DCT | Dopachrome Tautomerase | Protein Coding | P40126 | 55 | GC13M094436 |
| NGLY1 | N-Glycanase 1 | Protein Coding | Q96IV0 | 55 | GC03M025718 |
| LIFR | LIF Receptor Subunit Alpha | Protein Coding | P42702 | 59 | GC05M038645 |
| CXCL3 | C-X-C Motif Chemokine Ligand 3 | Protein Coding | P19876 | 50 | GC04M074036 |
| KRT10 | Keratin 10 | Protein Coding | P13645 | 56 | GC17M040818 |
| IGBP1 | Immunoglobulin Binding Protein 1 | Protein Coding | P78318 | 54 | GC0XP070133 |
| BPIFA1 | BPI Fold Containing Family A Member 1 | Protein Coding | Q9NP55 | 48 | GC20P033235 |
| SCARB1 | Scavenger Receptor Class B Member 1 | Protein Coding | Q8WTV0 | 60 | GC12M124776 |
| SSB | Small RNA Binding Exonuclease Protection Factor La | Protein Coding | P05455 | 54 | GC02P169791 |
| CCND3 | Cyclin D3 | Protein Coding | P30281 | 59 | GC06M041934 |
| GC | GC Vitamin D Binding Protein | Protein Coding | P02774 | 58 | GC04M071741 |
| E2F1 | E2F Transcription Factor 1 | Protein Coding | Q01094 | 56 | GC20M033675 |
| FABP5 | Fatty Acid Binding Protein 5 | Protein Coding | Q01469 | 55 | GC08P081282 |
| XRCC1 | X-Ray Repair Cross Complementing 1 | Protein Coding | P18887 | 58 | GC19M043543 |
| INO80 | INO80 Complex ATPase Subunit | Protein Coding | Q9ULG1 | 49 | GC15M042050 |
| TH | Tyrosine Hydroxylase | Protein Coding | P07101 | 63 | GC11M002163 |
| NEDD4L | NEDD4 Like E3 Ubiquitin Protein Ligase | Protein Coding | Q96PU5 | 58 | GC18P058044 |
| MIR188 | MicroRNA 188 | RNA Gene |  | 26 | GC0XP050003 |
| GRIN2A | Glutamate Ionotropic Receptor NMDA Type Subunit 2A | Protein Coding | Q12879 | 64 | GC16M009753 |
| LAMA2 | Laminin Subunit Alpha 2 | Protein Coding | P24043 | 56 | GC06P158062 |
| APCS | Amyloid P Component, Serum | Protein Coding | P02743 | 55 | GC01P159587 |
| IFI44 | Interferon Induced Protein 44 | Protein Coding | Q8TCB0 | 48 | GC01P078649 |
| FGR | FGR Proto-Oncogene, Src Family Tyrosine Kinase | Protein Coding | P09769 | 59 | GC01M032311 |
| CD8B | CD8 Subunit Beta | Protein Coding | P10966 | 54 | GC02M086815 |
| MIR338 | MicroRNA 338 | RNA Gene |  | 27 | GC17M090574 |
| MRPL28 | Mitochondrial Ribosomal Protein L28 | Protein Coding | Q13084 | 47 | GC16M000366 |
| SRF | Serum Response Factor | Protein Coding | P11831 | 56 | GC06P043171 |
| DSP | Desmoplakin | Protein Coding | P15924 | 63 | GC06P007541 |
| PDIA3 | Protein Disulfide Isomerase Family A Member 3 | Protein Coding | P30101 | 56 | GC15P043746 |
| SUOX | Sulfite Oxidase | Protein Coding | P51687 | 57 | GC12P055997 |
| CRLF2 | Cytokine Receptor Like Factor 2 | Protein Coding | Q9HC73 | 51 | GC0XM001190 |
| COL1A2 | Collagen Type I Alpha 2 Chain | Protein Coding | P08123 | 61 | GC07P094394 |
| PGR | Progesterone Receptor | Protein Coding | P06401 | 62 | GC11M133477 |
| MORC3 | MORC Family CW-Type Zinc Finger 3 | Protein Coding | Q14149 | 49 | GC21P036320 |
| DRD2 | Dopamine Receptor D2 | Protein Coding | P14416 | 62 | GC11M113409 |
| UCHL1 | Ubiquitin C-Terminal Hydrolase L1 | Protein Coding | P09936 | 65 | GC04P041256 |
| CHGA | Chromogranin A | Protein Coding | P10645 | 56 | GC14P093923 |
| ORM2 | Orosomucoid 2 | Protein Coding | P19652 | 51 | GC09P114329 |
| ADSL | Adenylosuccinate Lyase | Protein Coding | P30566 | 59 | GC22P040346 |
| MIR98 | MicroRNA 98 | RNA Gene |  | 27 | GC0XM053894 |
| MIR216A | MicroRNA 216a | RNA Gene |  | 29 | GC02M055988 |
| KRT5 | Keratin 5 | Protein Coding | P13647 | 58 | GC12M052514 |
| PSMC3 | Proteasome 26S Subunit, ATPase 3 | Protein Coding | P17980 | 56 | GC11M132470 |
| VSIR | V-Set Immunoregulatory Receptor | Protein Coding | Q9H7M9 | 52 | GC10M071748 |
| VEGFD | Vascular Endothelial Growth Factor D | Protein Coding | O43915 | 52 | GC0XM015345 |
| SNAP25 | Synaptosome Associated Protein 25 | Protein Coding | P60880 | 62 | GC20P010228 |
| ITGA6 | Integrin Subunit Alpha 6 | Protein Coding | P23229 | 62 | GC02P172241 |
| CD63 | CD63 Molecule | Protein Coding | P08962 | 56 | GC12M059805 |
| GSTP1 | Glutathione S-Transferase Pi 1 | Protein Coding | P09211 | 62 | GC11P067583 |
| MIR494 | MicroRNA 494 | RNA Gene |  | 26 | GC14P119078 |
| LOC102723407 | Immunoglobulin Heavy Variable 4-38-2-Like | Protein Coding |  | 16 | GC14P8B0041 |
| DNM2 | Dynamin 2 | Protein Coding | P50570 | 63 | GC19P010718 |
| DUSP1 | Dual Specificity Phosphatase 1 | Protein Coding | P28562 | 58 | GC05M172768 |
| GDF15 | Growth Differentiation Factor 15 | Protein Coding | Q99988 | 56 | GC19P132620 |
| NLRP10 | NLR Family Pyrin Domain Containing 10 | Protein Coding | Q86W26 | 45 | GC11M013571 |
| SPPL2A | Signal Peptide Peptidase Like 2A | Protein Coding | Q8TCT8 | 52 | GC15M050702 |
| TIMD4 | T Cell Immunoglobulin And Mucin Domain Containing 4 | Protein Coding | Q96H15 | 49 | GC05M156919 |
| KIR2DS5 | Killer Cell Immunoglobulin Like Receptor, Two Ig Domains And Short Cytoplasmic Tail 5 | Protein Coding | Q14953 | 29 | GC19MR00030 |
| NANOG | Nanog Homeobox | Protein Coding | Q9H9S0 | 50 | GC12P007787 |
| IMPDH2 | Inosine Monophosphate Dehydrogenase 2 | Protein Coding | P12268 | 61 | GC03M054250 |
| RPA1 | Replication Protein A1 | Protein Coding | P27694 | 59 | GC17P001829 |
| CEBPD | CCAAT Enhancer Binding Protein Delta | Protein Coding | P49716 | 52 | GC08M047759 |
| TEK | TEK Receptor Tyrosine Kinase | Protein Coding | Q02763 | 64 | GC09P027109 |
| EDNRA | Endothelin Receptor Type A | Protein Coding | P25101 | 62 | GC04P147480 |
| PTPRD | Protein Tyrosine Phosphatase Receptor Type D | Protein Coding | P23468 | 58 | GC09M008307 |
| MIR324 | MicroRNA 324 | RNA Gene |  | 30 | GC17M007223 |
| AOC1 | Amine Oxidase Copper Containing 1 | Protein Coding | P19801 | 55 | GC07P150824 |
| SLC39A7 | Solute Carrier Family 39 Member 7 | Protein Coding | Q92504 | 54 | GC06P033200 |
| NRP1 | Neuropilin 1 | Protein Coding | O14786 | 60 | GC10M033177 |
| NEFH | Neurofilament Heavy Chain | Protein Coding | P12036 | 58 | GC22P029480 |
| TXNIP | Thioredoxin Interacting Protein | Protein Coding | Q9H3M7 | 52 | GC01M145992 |
| MYSM1 | Myb Like, SWIRM And MPN Domains 1 | Protein Coding | Q5VVJ2 | 51 | GC01M058730 |
| DSC1 | Desmocollin 1 | Protein Coding | Q08554 | 50 | GC18M031129 |
| AOAH | Acyloxyacyl Hydrolase | Protein Coding | P28039 | 51 | GC07M036519 |
| TSPO | Translocator Protein | Protein Coding | B1AH88 | 57 | GC22P043151 |
| OXA1L | OXA1L Mitochondrial Inner Membrane Protein | Protein Coding | Q15070 | 50 | GC14P022766 |
| MIR29B1 | MicroRNA 29b-1 | RNA Gene |  | 29 | GC07M130877 |
| NR4A2 | Nuclear Receptor Subfamily 4 Group A Member 2 | Protein Coding | P43354 | 58 | GC02M156324 |
| PRG2 | Proteoglycan 2, Pro Eosinophil Major Basic Protein | Protein Coding | P13727 | 52 | GC11M057386 |
| CTCF | CCCTC-Binding Factor | Protein Coding | P49711 | 59 | GC16P105895 |
| CST3 | Cystatin C | Protein Coding | P01034 | 56 | GC20M024131 |
| CADM1 | Cell Adhesion Molecule 1 | Protein Coding | Q9BY67 | 57 | GC11M115169 |
| PELI3 | Pellino E3 Ubiquitin Protein Ligase Family Member 3 | Protein Coding | Q8N2H9 | 43 | GC11P066466 |
| UBA7 | Ubiquitin Like Modifier Activating Enzyme 7 | Protein Coding | P41226 | 52 | GC03M049805 |
| CDKN3 | Cyclin Dependent Kinase Inhibitor 3 | Protein Coding | Q16667 | 53 | GC14P054417 |
| P4HB | Prolyl 4-Hydroxylase Subunit Beta | Protein Coding | P07237 | 62 | GC17M081843 |
| MIR328 | MicroRNA 328 | RNA Gene |  | 29 | GC16M067652 |
| LILRA2 | Leukocyte Immunoglobulin Like Receptor A2 | Protein Coding | Q8N149 | 50 | GC19P054572 |
| CLEC4G | C-Type Lectin Domain Family 4 Member G | Protein Coding | Q6UXB4 | 49 | GC19M007728 |
| PPP2CA | Protein Phosphatase 2 Catalytic Subunit Alpha | Protein Coding | P67775 | 63 | GC05M134194 |
| MIR382 | MicroRNA 382 | RNA Gene |  | 26 | GC14P119068 |
| NR1H3 | Nuclear Receptor Subfamily 1 Group H Member 3 | Protein Coding | Q13133 | 59 | GC11P047248 |
| KIR2DS2 | Killer Cell Immunoglobulin Like Receptor, Two Ig Domains And Short Cytoplasmic Tail 2 | Protein Coding | P43631 | 27 | GC19Mr00125 |
| SLC6A4 | Solute Carrier Family 6 Member 4 | Protein Coding | P31645 | 61 | GC17M030194 |
| C9orf72 | C9orf72-SMCR8 Complex Subunit | Protein Coding | Q96LT7 | 54 | GC09M029889 |
| NPC2 | NPC Intracellular Cholesterol Transporter 2 | Protein Coding | P61916 | 56 | GC14M074476 |
| ITLN1 | Intelectin 1 | Protein Coding | Q8WWA0 | 50 | GC01M160876 |
| DHODH | Dihydroorotate Dehydrogenase (Quinone) | Protein Coding | Q02127 | 59 | GC16P072008 |
| CNPY3 | Canopy FGF Signaling Regulator 3 | Protein Coding | Q9BT09 | 52 | GC06P156496 |
| NUP107 | Nucleoporin 107 | Protein Coding | P57740 | 56 | GC12P068686 |
| DOP1A | DOP1 Leucine Zipper Like Protein A | Protein Coding | Q5JWR5 | 42 | GC06P156981 |
| EPRS1 | Glutamyl-Prolyl-TRNA Synthetase 1 | Protein Coding | P07814 | 56 | GC01M219969 |
| FGF5 | Fibroblast Growth Factor 5 | Protein Coding | P12034 | 56 | GC04P080266 |
| PANX1 | Pannexin 1 | Protein Coding | Q96RD7 | 58 | GC11P094128 |
| AIFM1 | Apoptosis Inducing Factor Mitochondria Associated 1 | Protein Coding | O95831 | 63 | GC0XM130129 |
| RYR3 | Ryanodine Receptor 3 | Protein Coding | Q15413 | 54 | GC15P033310 |
| CRBN | Cereblon | Protein Coding | Q96SW2 | 58 | GC03M003144 |
| MIR134 | MicroRNA 134 | RNA Gene |  | 27 | GC14P119048 |
| MIR182 | MicroRNA 182 | RNA Gene |  | 31 | GC07M129770 |
| H3C12 | H3 Clustered Histone 12 | Protein Coding | P68431 | 47 | GC06M101383 |
| H3C13 | H3 Clustered Histone 13 | Protein Coding | Q71DI3 | 45 | GC01M164846 |
| PTMA | Prothymosin Alpha | Protein Coding | P06454 | 53 | GC02P233767 |
| MYLK | Myosin Light Chain Kinase | Protein Coding | Q15746 | 65 | GC03M123610 |
| IL17B | Interleukin 17B | Protein Coding | Q9UHF5 | 52 | GC05M149371 |
| MIR532 | MicroRNA 532 | RNA Gene |  | 25 | GC0XP059332 |
| AHSG | Alpha 2-HS Glycoprotein | Protein Coding | P02765 | 57 | GC03P186673 |
| CLEC1A | C-Type Lectin Domain Family 1 Member A | Protein Coding | Q8NC01 | 50 | GC12M033110 |
| PDPN | Podoplanin | Protein Coding | Q86YL7 | 54 | GC01P013583 |
| HLA-DOB | Major Histocompatibility Complex, Class II, DO Beta | Protein Coding | P13765 | 44 | GC06M100369 |
| MIR331 | MicroRNA 331 | RNA Gene |  | 29 | GC12P095308 |
| RNF213 | Ring Finger Protein 213 | Protein Coding | Q63HN8 | 50 | GC17P080260 |
| GLUD1 | Glutamate Dehydrogenase 1 | Protein Coding | P00367 | 63 | GC10M087050 |
| KIF1C | Kinesin Family Member 1C | Protein Coding | O43896 | 54 | GC17P132526 |
| MIR361 | MicroRNA 361 | RNA Gene |  | 27 | GC0XM085903 |
| MIR451A | MicroRNA 451a | RNA Gene |  | 26 | GC17M028861 |
| HOTAIR | HOX Transcript Antisense RNA | RNA Gene |  | 31 | GC12M053962 |
| IARS2 | Isoleucyl-TRNA Synthetase 2, Mitochondrial | Protein Coding | Q9NSE4 | 54 | GC01P220094 |
| MIR202 | MicroRNA 202 | RNA Gene |  | 27 | GC10M133247 |
| MIR30D | MicroRNA 30d | RNA Gene |  | 26 | GC08M134804 |
| CACNA1G-AS1 | CACNA1G Antisense RNA 1 | RNA Gene |  | 23 | GC17M089771 |
| SAG | S-Antigen Visual Arrestin | Protein Coding | P10523 | 58 | GC02P233868 |
| TRIM31 | Tripartite Motif Containing 31 | Protein Coding | Q9BZY9 | 48 | GC06M100201 |
| HDAC3 | Histone Deacetylase 3 | Protein Coding | O15379 | 62 | GC05M141620 |
| NRON | Non-Coding Repressor Of NFAT | RNA Gene |  | 25 | GC09M126407 |
| POLR3F | RNA Polymerase III Subunit F | Protein Coding | Q9H1D9 | 54 | GC20P019656 |
| FOSL2 | FOS Like 2, AP-1 Transcription Factor Subunit | Protein Coding | P15408 | 54 | GC02P028392 |
| ATRIP-TREX1 | ATRIP-TREX1 Readthrough | RNA Gene |  | 14 | GC03P061481 |
| GPBAR1 | G Protein-Coupled Bile Acid Receptor 1 | Protein Coding | Q8TDU6 | 52 | GC02P218259 |
| RNF125 | Ring Finger Protein 125 | Protein Coding | Q96EQ8 | 52 | GC18P032523 |
| FUT4 | Fucosyltransferase 4 | Protein Coding | P22083 | 52 | GC11P099906 |
| MEF2C | Myocyte Enhancer Factor 2C | Protein Coding | Q06413 | 62 | GC05M088718 |
| BTN3A2 | Butyrophilin Subfamily 3 Member A2 | Protein Coding | P78410 | 46 | GC06P026365 |
| GZMH | Granzyme H | Protein Coding | P20718 | 49 | GC14M024606 |
| MIR130B | MicroRNA 130b | RNA Gene |  | 28 | GC22P085687 |
| TDO2 | Tryptophan 2,3-Dioxygenase | Protein Coding | P48775 | 58 | GC04P155854 |
| SRSF2 | Serine And Arginine Rich Splicing Factor 2 | Protein Coding | Q01130 | 55 | GC17M076734 |
| SLC5A6 | Solute Carrier Family 5 Member 6 | Protein Coding | Q9Y289 | 56 | GC02M027201 |
| GM2A | Ganglioside GM2 Activator | Protein Coding | P17900 | 55 | GC05P151212 |
| DDB1 | Damage Specific DNA Binding Protein 1 | Protein Coding | Q16531 | 56 | GC11M132628 |
| OTUD5 | OTU Deubiquitinase 5 | Protein Coding | Q96G74 | 50 | GC0XM048922 |
| HLA-DOA | Major Histocompatibility Complex, Class II, DO Alpha | Protein Coding | P06340 | 47 | GC06M033004 |
| VCL | Vinculin | Protein Coding | P18206 | 60 | GC10P073995 |
| PGLYRP2 | Peptidoglycan Recognition Protein 2 | Protein Coding | Q96PD5 | 46 | GC19M015468 |
| PROS1 | Protein S | Protein Coding | P07225 | 61 | GC03M093873 |
| LILRA4 | Leukocyte Immunoglobulin Like Receptor A4 | Protein Coding | P59901 | 48 | GC19M054333 |
| MIRLET7A1 | MicroRNA Let-7a-1 | RNA Gene |  | 31 | GC09P094175 |
| ITGAE | Integrin Subunit Alpha E | Protein Coding | P38570 | 52 | GC17M088106 |
| HEXIM1 | HEXIM P-TEFb Complex Subunit 1 | Protein Coding | O94992 | 47 | GC17P133879 |
| IL1RAPL2 | Interleukin 1 Receptor Accessory Protein Like 2 | Protein Coding | Q9NP60 | 50 | GC0XP104566 |
| PXDN | Peroxidasin | Protein Coding | Q92626 | 56 | GC02M001635 |
| ERN1 | Endoplasmic Reticulum To Nucleus Signaling 1 | Protein Coding | O75460 | 60 | GC17M064039 |
| IL36B | Interleukin 36 Beta | Protein Coding | Q9NZH7 | 45 | GC02M113022 |
| TGFA | Transforming Growth Factor Alpha | Protein Coding | P01135 | 58 | GC02M070447 |
| CYP2R1 | Cytochrome P450 Family 2 Subfamily R Member 1 | Protein Coding | Q6VVX0 | 55 | GC11M014877 |
| MPEG1 | Macrophage Expressed 1 | Protein Coding | Q2M385 | 46 | GC11M059208 |
| ALAD | Aminolevulinate Dehydratase | Protein Coding | P13716 | 58 | GC09M113386 |
| WNT5A | Wnt Family Member 5A | Protein Coding | P41221 | 63 | GC03M055465 |
| SPTLC2 | Serine Palmitoyltransferase Long Chain Base Subunit 2 | Protein Coding | O15270 | 59 | GC14M077505 |
| G3BP1 | G3BP Stress Granule Assembly Factor 1 | Protein Coding | Q13283 | 54 | GC05P151771 |
| MIR205 | MicroRNA 205 | RNA Gene |  | 29 | GC01P209432 |
| IGK | Immunoglobulin Kappa Locus | Protein Coding |  | 21 | GC02P088857 |
| KMT2A | Lysine Methyltransferase 2A | Protein Coding | Q03164 | 60 | GC11P118436 |
| MMP14 | Matrix Metallopeptidase 14 | Protein Coding | P50281 | 63 | GC14P051698 |
| ATF3 | Activating Transcription Factor 3 | Protein Coding | P18847 | 57 | GC01P212565 |
| KLK3 | Kallikrein Related Peptidase 3 | Protein Coding | P07288 | 59 | GC19P050854 |
| PCAT1 | Prostate Cancer Associated Transcript 1 | RNA Gene |  | 25 | GC08P126553 |
| THY1 | Thy-1 Cell Surface Antigen | Protein Coding | P04216 | 56 | GC11M134063 |
| PRIM1 | DNA Primase Subunit 1 | Protein Coding | P49642 | 54 | GC12M059864 |
| SCO2 | Synthesis Of Cytochrome C Oxidase 2 | Protein Coding | O43819 | 57 | GC22M050523 |
| ALG13 | ALG13 UDP-N-Acetylglucosaminyltransferase Subunit | Protein Coding | Q9NP73 | 51 | GC0XP111665 |
| LAIR2 | Leukocyte Associated Immunoglobulin Like Receptor 2 | Protein Coding | Q6ISS4 | 43 | GC19P133669 |
| LEF1 | Lymphoid Enhancer Binding Factor 1 | Protein Coding | Q9UJU2 | 61 | GC04M108047 |
| SCN9A | Sodium Voltage-Gated Channel Alpha Subunit 9 | Protein Coding | Q15858 | 60 | GC02M166195 |
| WASF1 | WASP Family Member 1 | Protein Coding | Q92558 | 57 | GC06M110099 |
| PIP | Prolactin Induced Protein | Protein Coding | P12273 | 50 | GC07P143132 |
| MIR181A2 | MicroRNA 181a-2 | RNA Gene |  | 29 | GC09P124692 |
| SMC3 | Structural Maintenance Of Chromosomes 3 | Protein Coding | Q9UQE7 | 60 | GC10P110567 |
| PSIP1 | PC4 And SRSF1 Interacting Protein 1 | Protein Coding | O75475 | 52 | GC09M015464 |
| PMS1 | PMS1 Homolog 1, Mismatch Repair System Component | Protein Coding | P54277 | 52 | GC02P189784 |
| NQO1 | NAD(P)H Quinone Dehydrogenase 1 | Protein Coding | P15559 | 60 | GC16M069706 |
| MIR503 | MicroRNA 503 | RNA Gene |  | 28 | GC0XM134898 |
| HDAC6 | Histone Deacetylase 6 | Protein Coding | Q9UBN7 | 66 | GC0XP048801 |
| RYR2 | Ryanodine Receptor 2 | Protein Coding | Q92736 | 59 | GC01P237042 |
| KIR2DS1 | Killer Cell Immunoglobulin Like Receptor, Two Ig Domains And Short Cytoplasmic Tail 1 | Protein Coding | Q14954 | 27 | GC19Mr00063 |
| GNB1 | G Protein Subunit Beta 1 | Protein Coding | P62873 | 58 | GC01M001785 |
| GRIN2B | Glutamate Ionotropic Receptor NMDA Type Subunit 2B | Protein Coding | Q13224 | 65 | GC12M013437 |
| YBX1 | Y-Box Binding Protein 1 | Protein Coding | P67809 | 53 | GC01P042682 |
| TNFAIP8L2 | TNF Alpha Induced Protein 8 Like 2 | Protein Coding | Q6P589 | 38 | GC01P151156 |
| REEP1 | Receptor Accessory Protein 1 | Protein Coding | Q9H902 | 52 | GC02M086213 |
| KIF1A | Kinesin Family Member 1A | Protein Coding | Q12756 | 56 | GC02M240713 |
| CD300E | CD300e Molecule | Protein Coding | Q496F6 | 44 | GC17M074609 |
| SNHG1 | Small Nucleolar RNA Host Gene 1 | RNA Gene |  | 29 | GC11M132691 |
| FBXO38 | F-Box Protein 38 | Protein Coding | Q6PIJ6 | 48 | GC05P148383 |
| ACKR1 | Atypical Chemokine Receptor 1 (Duffy Blood Group) | Protein Coding | Q16570 | 55 | GC01P170698 |
| HTT | Huntingtin | Protein Coding | P42858 | 56 | GC04P003041 |
| ALG6 | ALG6 Alpha-1,3-Glucosyltransferase | Protein Coding | Q9Y672 | 52 | GC01P063367 |
| PTGS1 | Prostaglandin-Endoperoxide Synthase 1 | Protein Coding | P23219 | 59 | GC09P122370 |
| DDB2 | Damage Specific DNA Binding Protein 2 | Protein Coding | Q92466 | 59 | GC11P048386 |
| LTB4R | Leukotriene B4 Receptor | Protein Coding | Q15722 | 54 | GC14P024311 |
| EIF4E | Eukaryotic Translation Initiation Factor 4E | Protein Coding | P06730 | 62 | GC04M098879 |
| ABCB4 | ATP Binding Cassette Subfamily B Member 4 | Protein Coding | P21439 | 59 | GC07M087365 |
| RARS1 | Arginyl-TRNA Synthetase 1 | Protein Coding | P54136 | 56 | GC05P168487 |
| H3-3B | H3.3 Histone B | Protein Coding | P84243 | 51 | GC17M090374 |
| ARHGEF2 | Rho/Rac Guanine Nucleotide Exchange Factor 2 | Protein Coding | Q92974 | 59 | GC01M155946 |
| CAMLG | Calcium Modulating Ligand | Protein Coding | P49069 | 52 | GC05P134738 |
| BMAL1 | Basic Helix-Loop-Helix ARNT Like 1 | Protein Coding | O00327 | 54 | GC11P014689 |
| NUP153 | Nucleoporin 153 | Protein Coding | P49790 | 54 | GC06M017615 |
| ODC1 | Ornithine Decarboxylase 1 | Protein Coding | P11926 | 61 | GC02M010432 |
| TBL1XR1 | TBL1X/Y Related 1 | Protein Coding | Q9BZK7 | 58 | GC03M177019 |
| ATIC | 5-Aminoimidazole-4-Carboxamide Ribonucleotide Formyltransferase/IMP Cyclohydrolase | Protein Coding | P31939 | 58 | GC02P215311 |
| MAG | Myelin Associated Glycoprotein | Protein Coding | P20916 | 59 | GC19P035292 |
| YWHAZ | Tyrosine 3-Monooxygenase/Tryptophan 5-Monooxygenase Activation Protein Zeta | Protein Coding | P63104 | 59 | GC08M100957 |
| MIRLET7G | MicroRNA Let-7g | RNA Gene |  | 28 | GC03M052268 |
| ZFP36 | ZFP36 Ring Finger Protein | Protein Coding | P26651 | 49 | GC19P039406 |
| CEP57 | Centrosomal Protein 57 | Protein Coding | Q86XR8 | 54 | GC11P095789 |
| TGM1 | Transglutaminase 1 | Protein Coding | P22735 | 58 | GC14M024249 |
| PRSS3 | Serine Protease 3 | Protein Coding | P35030 | 54 | GC09P033750 |
| SOCS2 | Suppressor Of Cytokine Signaling 2 | Protein Coding | O14508 | 55 | GC12P093569 |
| STX4 | Syntaxin 4 | Protein Coding | Q12846 | 56 | GC16P104996 |
| SFTPC | Surfactant Protein C | Protein Coding | P11686 | 52 | GC08P022156 |
| CD1E | CD1e Molecule | Protein Coding | P15812 | 49 | GC01P170665 |
| LRP2 | LDL Receptor Related Protein 2 | Protein Coding | P98164 | 61 | GC02M169127 |
| ATF2 | Activating Transcription Factor 2 | Protein Coding | P15336 | 60 | GC02M175072 |
| H2BC21 | H2B Clustered Histone 21 | Protein Coding | Q16778 | 52 | GC01M164856 |
| NKX2-5 | NK2 Homeobox 5 | Protein Coding | P52952 | 57 | GC05M173232 |
| CTSE | Cathepsin E | Protein Coding | P14091 | 55 | GC01M206009 |
| GPR35 | G Protein-Coupled Receptor 35 | Protein Coding | Q9HC97 | 54 | GC02P240605 |
| CLTCL1 | Clathrin Heavy Chain Like 1 | Protein Coding | P53675 | 52 | GC22M020415 |
| HSPA9 | Heat Shock Protein Family A (Hsp70) Member 9 | Protein Coding | P38646 | 61 | GC05M138554 |
| SETBP1 | SET Binding Protein 1 | Protein Coding | Q9Y6X0 | 54 | GC18P044680 |
| FURIN | Furin, Paired Basic Amino Acid Cleaving Enzyme | Protein Coding | P09958 | 61 | GC15P090868 |
| HACE1 | HECT Domain And Ankyrin Repeat Containing E3 Ubiquitin Protein Ligase 1 | Protein Coding | Q8IYU2 | 54 | GC06M104728 |
| MIR204 | MicroRNA 204 | RNA Gene |  | 31 | GC09M070809 |
| GARS1 | Glycyl-TRNA Synthetase 1 | Protein Coding | P41250 | 58 | GC07P030580 |
| TRBV11-1 | T Cell Receptor Beta Variable 11-1 | Protein Coding | A0A0K0K1C0 | 14 | GC07P163101 |
| TRBV11-3 | T Cell Receptor Beta Variable 11-3 | Protein Coding | A0A5A6 | 12 | GC07P163103 |
| CALM2 | Calmodulin 2 | Protein Coding | P0DP24 | 56 | GC02M047160 |
| H1-4 | H1.4 Linker Histone, Cluster Member | Protein Coding | P10412 | 55 | GC06P164126 |
| BUB1 | BUB1 Mitotic Checkpoint Serine/Threonine Kinase | Protein Coding | O43683 | 61 | GC02M110637 |
| CTBP1 | C-Terminal Binding Protein 1 | Protein Coding | Q13363 | 61 | GC04M001211 |
| TP73 | Tumor Protein P73 | Protein Coding | O15350 | 58 | GC01P003652 |
| IDUA | Alpha-L-Iduronidase | Protein Coding | P35475 | 58 | GC04P000986 |
| CMA1 | Chymase 1 | Protein Coding | P23946 | 56 | GC14M027988 |
| TKT | Transketolase | Protein Coding | P29401 | 58 | GC03M053224 |
| TRIM29 | Tripartite Motif Containing 29 | Protein Coding | Q14134 | 51 | GC11M120111 |
| NOTCH3 | Notch Receptor 3 | Protein Coding | Q9UM47 | 64 | GC19M015159 |
| INF2 | Inverted Formin 2 | Protein Coding | Q27J81 | 52 | GC14P118131 |
| NONO | Non-POU Domain Containing Octamer Binding | Protein Coding | Q15233 | 58 | GC0XP071267 |
| ITGA1 | Integrin Subunit Alpha 1 | Protein Coding | P56199 | 58 | GC05P052788 |
| LINC00426 | Long Intergenic Non-Protein Coding RNA 426 | RNA Gene |  | 22 | GC13M030593 |
| SERPINB3 | Serpin Family B Member 3 | Protein Coding | P29508 | 52 | GC18M063655 |
| CXCR6 | C-X-C Motif Chemokine Receptor 6 | Protein Coding | O00574 | 51 | GC03P061345 |
| KLF4 | KLF Transcription Factor 4 | Protein Coding | O43474 | 59 | GC09M107484 |
| PRKRA | Protein Activator Of Interferon Induced Protein Kinase EIF2AK2 | Protein Coding | O75569 | 54 | GC02M178431 |
| CYLD-AS1 | CYLD Antisense RNA 1 | RNA Gene |  | 20 | GC16M050736 |
| TIA1 | TIA1 Cytotoxic Granule Associated RNA Binding Protein | Protein Coding | P31483 | 54 | GC02M070209 |
| ARRB1 | Arrestin Beta 1 | Protein Coding | P49407 | 56 | GC11M133131 |
| CDK9 | Cyclin Dependent Kinase 9 | Protein Coding | P50750 | 59 | GC09P146597 |
| GHRH | Growth Hormone Releasing Hormone | Protein Coding | P01286 | 50 | GC20M037251 |
| TBP | TATA-Box Binding Protein | Protein Coding | P20226 | 62 | GC06P170554 |
| GRIN1 | Glutamate Ionotropic Receptor NMDA Type Subunit 1 | Protein Coding | Q05586 | 63 | GC09P137138 |
| FIG4 | FIG4 Phosphoinositide 5-Phosphatase | Protein Coding | Q92562 | 56 | GC06P157520 |
| CALML5 | Calmodulin Like 5 | Protein Coding | Q9NZT1 | 49 | GC10M005498 |
| DHPS | Deoxyhypusine Synthase | Protein Coding | P49366 | 56 | GC19M100369 |
| C4BPB | Complement Component 4 Binding Protein Beta | Protein Coding | P20851 | 54 | GC01P207088 |
| CTAG1B | Cancer/Testis Antigen 1B | Protein Coding | P78358 | 40 | GC0XM154617 |
| B3GAT1 | Beta-1,3-Glucuronyltransferase 1 | Protein Coding | Q9P2W7 | 57 | GC11M134378 |
| DMBT1 | Deleted In Malignant Brain Tumors 1 | Protein Coding | Q9UGM3 | 52 | GC10P122560 |
| SFTPB | Surfactant Protein B | Protein Coding | P07988 | 55 | GC02M085657 |
| SNRPN | Small Nuclear Ribonucleoprotein Polypeptide N | Protein Coding | P63162 | 56 | GC15P024823 |
| MMP12 | Matrix Metallopeptidase 12 | Protein Coding | P39900 | 58 | GC11M102862 |
| PIK3R4 | Phosphoinositide-3-Kinase Regulatory Subunit 4 | Protein Coding | Q99570 | 58 | GC03M130678 |
| IL20RB | Interleukin 20 Receptor Subunit Beta | Protein Coding | Q6UXL0 | 45 | GC03P136946 |
| ACTA2 | Actin Alpha 2, Smooth Muscle | Protein Coding | P62736 | 61 | GC10M088935 |
| USP14 | Ubiquitin Specific Peptidase 14 | Protein Coding | P54578 | 56 | GC18P000158 |
| ATN1 | Atrophin 1 | Protein Coding | P54259 | 56 | GC12P046787 |
| KNG1 | Kininogen 1 | Protein Coding | P01042 | 61 | GC03P186717 |
| CYP2D6 | Cytochrome P450 Family 2 Subfamily D Member 6 | Protein Coding | P10635 | 60 | GC22M042126 |
| GYPC | Glycophorin C (Gerbich Blood Group) | Protein Coding | P04921 | 55 | GC02P148782 |
| FBN1 | Fibrillin 1 | Protein Coding | P35555 | 60 | GC15M048408 |
| IL20RA | Interleukin 20 Receptor Subunit Alpha | Protein Coding | Q9UHF4 | 48 | GC06M136999 |
| MAFB | MAF BZIP Transcription Factor B | Protein Coding | Q9Y5Q3 | 55 | GC20M040685 |
| TCF7 | Transcription Factor 7 | Protein Coding | P36402 | 54 | GC05P134344 |
| LILRB3 | Leukocyte Immunoglobulin Like Receptor B3 | Protein Coding | O75022 | 47 | GC19M054216 |
| MIR320A | MicroRNA 320a | RNA Gene |  | 30 | GC08M022379 |
| AGK | Acylglycerol Kinase | Protein Coding | Q53H12 | 52 | GC07P141551 |
| GSR | Glutathione-Disulfide Reductase | Protein Coding | P00390 | 62 | GC08M030678 |
| VSIG4 | V-Set And Immunoglobulin Domain Containing 4 | Protein Coding | Q9Y279 | 49 | GC0XM066021 |
| RAP1A | RAP1A, Member Of RAS Oncogene Family | Protein Coding | P62834 | 56 | GC01P111542 |
| KCNJ10 | Potassium Inwardly Rectifying Channel Subfamily J Member 10 | Protein Coding | P78508 | 58 | GC01M159998 |
| ESR2 | Estrogen Receptor 2 | Protein Coding | Q92731 | 61 | GC14M064084 |
| RRM2B | Ribonucleotide Reductase Regulatory TP53 Inducible Subunit M2B | Protein Coding | Q7LG56 | 61 | GC08M102204 |
| BGN | Biglycan | Protein Coding | P21810 | 57 | GC0XP153494 |
| JAG1 | Jagged Canonical Notch Ligand 1 | Protein Coding | P78504 | 63 | GC20M010637 |
| CCK | Cholecystokinin | Protein Coding | P06307 | 54 | GC03M042274 |
| CDKN2C | Cyclin Dependent Kinase Inhibitor 2C | Protein Coding | P42773 | 56 | GC01P050960 |
| KCNQ1OT1 | KCNQ1 Opposite Strand/Antisense Transcript 1 | RNA Gene |  | 36 | GC11M013323 |
| HOTTIP | HOXA Distal Transcript Antisense RNA | RNA Gene |  | 30 | GC07P027198 |
| GATA4 | GATA Binding Protein 4 | Protein Coding | P43694 | 62 | GC08P011676 |
| CASP7 | Caspase 7 | Protein Coding | P55210 | 62 | GC10P113679 |
| VIPR1 | Vasoactive Intestinal Peptide Receptor 1 | Protein Coding | P32241 | 59 | GC03P042490 |
| DGCR8 | DGCR8 Microprocessor Complex Subunit | Protein Coding | Q8WYQ5 | 55 | GC22P020080 |
| MIR17HG | MiR-17-92a-1 Cluster Host Gene | RNA Gene | Q75NE6 | 36 | GC13P091347 |
| PIK3C2A | Phosphatidylinositol-4-Phosphate 3-Kinase Catalytic Subunit Type 2 Alpha | Protein Coding | O00443 | 59 | GC11M018024 |
| TIMP3 | TIMP Metallopeptidase Inhibitor 3 | Protein Coding | P35625 | 56 | GC22P081341 |
| FBXO11 | F-Box Protein 11 | Protein Coding | Q86XK2 | 54 | GC02M047789 |
| YWHAE | Tyrosine 3-Monooxygenase/Tryptophan 5-Monooxygenase Activation Protein Epsilon | Protein Coding | P62258 | 62 | GC17M087979 |
| GJC2 | Gap Junction Protein Gamma 2 | Protein Coding | Q5T442 | 54 | GC01P232240 |
| SEC23A | SEC23 Homolog A, COPII Coat Complex Component | Protein Coding | Q15436 | 55 | GC14M039031 |
| MID1 | Midline 1 | Protein Coding | O15344 | 55 | GC0XM010445 |
| P2RX4 | Purinergic Receptor P2X 4 | Protein Coding | Q99571 | 57 | GC12P136100 |
| MIR196A1 | MicroRNA 196a-1 | RNA Gene |  | 30 | GC17M048632 |
| HERC2 | HECT And RLD Domain Containing E3 Ubiquitin Protein Ligase 2 | Protein Coding | O95714 | 57 | GC15M028111 |
| UBASH3A | Ubiquitin Associated And SH3 Domain Containing A | Protein Coding | P57075 | 50 | GC21P042403 |
| JCHAIN | Joining Chain Of Multimeric IgA And IgM | Protein Coding | P01591 | 47 | GC04M070655 |
| MIR19B1 | MicroRNA 19b-1 | RNA Gene |  | 26 | GC13P091714 |
| MIR486-1 | MicroRNA 486-1 | RNA Gene |  | 28 | GC08M041660 |
| CCND2 | Cyclin D2 | Protein Coding | P30279 | 61 | GC12P046708 |
| MIR125B2 | MicroRNA 125b-2 | RNA Gene |  | 30 | GC21P016590 |
| TARDBP | TAR DNA Binding Protein | Protein Coding | Q13148 | 59 | GC01P060311 |
| CPOX | Coproporphyrinogen Oxidase | Protein Coding | P36551 | 57 | GC03M098576 |
| ELAVL1 | ELAV Like RNA Binding Protein 1 | Protein Coding | Q15717 | 54 | GC19M007958 |
| POLH | DNA Polymerase Eta | Protein Coding | Q9Y253 | 58 | GC06P043576 |
| NEUROG3 | Neurogenin 3 | Protein Coding | Q9Y4Z2 | 51 | GC10M069571 |
| RHBDF2 | Rhomboid 5 Homolog 2 | Protein Coding | Q6PJF5 | 51 | GC17M076470 |
| SKAP2 | Src Kinase Associated Phosphoprotein 2 | Protein Coding | O75563 | 48 | GC07M026654 |
| NUP85 | Nucleoporin 85 | Protein Coding | Q9BW27 | 52 | GC17P075205 |
| KIR3DS1 | Killer Cell Immunoglobulin Like Receptor, Three Ig Domains And Short Cytoplasmic Tail 1 | Protein Coding | Q14943 | 26 | GC19MR00058 |
| MT-CO1 | Mitochondrially Encoded Cytochrome C Oxidase I | Protein Coding | P00395 | 46 | GCMTP005906 |
| SMC1A | Structural Maintenance Of Chromosomes 1A | Protein Coding | Q14683 | 61 | GC0XM053374 |
| IGHV3-21 | Immunoglobulin Heavy Variable 3-21 | Protein Coding | A0A0B4J1V1 | 23 | GC14M122317 |
| TRIM26 | Tripartite Motif Containing 26 | Protein Coding | Q12899 | 46 | GC06M030184 |
| FEN1 | Flap Structure-Specific Endonuclease 1 | Protein Coding | P39748 | 58 | GC11P098665 |
| MGAT2 | Alpha-1,6-Mannosyl-Glycoprotein 2-Beta-N-Acetylglucosaminyltransferase | Protein Coding | Q10469 | 56 | GC14P049620 |
| BGLAP | Bone Gamma-Carboxyglutamate Protein | Protein Coding | P02818 | 52 | GC01P156242 |
| SEMA4A | Semaphorin 4A | Protein Coding | Q9H3S1 | 54 | GC01P156147 |
| ASXL1 | ASXL Transcriptional Regulator 1 | Protein Coding | Q8IXJ9 | 55 | GC20P044308 |
| CEL | Carboxyl Ester Lipase | Protein Coding | P19835 | 58 | GC09P133061 |
| RDX | Radixin | Protein Coding | P35241 | 61 | GC11M109864 |
| MIR148B | MicroRNA 148b | RNA Gene |  | 30 | GC12P054337 |
| ANKRD17 | Ankyrin Repeat Domain 17 | Protein Coding | O75179 | 50 | GC04M073074 |
| HMGCR | 3-Hydroxy-3-Methylglutaryl-CoA Reductase | Protein Coding | P04035 | 61 | GC05P075336 |
| FUS | FUS RNA Binding Protein | Protein Coding | P35637 | 58 | GC16P031180 |
| DNTT | DNA Nucleotidylexotransferase | Protein Coding | P04053 | 54 | GC10P096304 |
| HSD11B1 | Hydroxysteroid 11-Beta Dehydrogenase 1 | Protein Coding | P28845 | 63 | GC01P209686 |
| GBP2 | Guanylate Binding Protein 2 | Protein Coding | P32456 | 44 | GC01M089106 |
| GOT2 | Glutamic-Oxaloacetic Transaminase 2 | Protein Coding | P00505 | 60 | GC16M058707 |
| FTO | FTO Alpha-Ketoglutarate Dependent Dioxygenase | Protein Coding | Q9C0B1 | 58 | GC16P105500 |
| SUCLA2 | Succinate-CoA Ligase ADP-Forming Subunit Beta | Protein Coding | Q9P2R7 | 58 | GC13M047745 |
| PSMD5 | Proteasome 26S Subunit, Non-ATPase 5 | Protein Coding | Q16401 | 50 | GC09M120815 |
| DEFA3 | Defensin Alpha 3 | Protein Coding | P59666 | 44 | GC08M007015 |
| CFHR4 | Complement Factor H Related 4 | Protein Coding | Q92496 | 47 | GC01P196888 |
| ABCB11 | ATP Binding Cassette Subfamily B Member 11 | Protein Coding | O95342 | 59 | GC02M168922 |
| ACKR4 | Atypical Chemokine Receptor 4 | Protein Coding | Q9NPB9 | 45 | GC03P132597 |
| CD82 | CD82 Molecule | Protein Coding | P27701 | 56 | GC11P044564 |
| SURF1 | SURF1 Cytochrome C Oxidase Assembly Factor | Protein Coding | Q15526 | 54 | GC09M133351 |
| ATL1 | Atlastin GTPase 1 | Protein Coding | Q8WXF7 | 52 | GC14P050532 |
| NPC1 | NPC Intracellular Cholesterol Transporter 1 | Protein Coding | O15118 | 62 | GC18M023506 |
| MIR378A | MicroRNA 378a | RNA Gene |  | 30 | GC05P149732 |
| RPS27A | Ribosomal Protein S27a | Protein Coding | P62979 | 55 | GC02P055231 |
| SLC30A2 | Solute Carrier Family 30 Member 2 | Protein Coding | Q9BRI3 | 52 | GC01M032186 |
| NFKBIL1 | NFKB Inhibitor Like 1 | Protein Coding | Q9UBC1 | 46 | GC06P156241 |
| TFG | Trafficking From ER To Golgi Regulator | Protein Coding | Q92734 | 55 | GC03P100709 |
| POLR3C | RNA Polymerase III Subunit C | Protein Coding | Q9BUI4 | 49 | GC01P145824 |
| GPR55 | G Protein-Coupled Receptor 55 | Protein Coding | Q9Y2T6 | 52 | GC02M230907 |
| ARVCF | ARVCF Delta Catenin Family Member | Protein Coding | O00192 | 51 | GC22M020445 |
| HUWE1 | HECT, UBA And WWE Domain Containing E3 Ubiquitin Protein Ligase 1 | Protein Coding | Q7Z6Z7 | 59 | GC0XM053532 |
| TACR3 | Tachykinin Receptor 3 | Protein Coding | P29371 | 62 | GC04M103586 |
| LMO2 | LIM Domain Only 2 | Protein Coding | P25791 | 55 | GC11M033858 |
| ATP7B | ATPase Copper Transporting Beta | Protein Coding | P35670 | 61 | GC13M051930 |
| PINK1 | PTEN Induced Kinase 1 | Protein Coding | Q9BXM7 | 59 | GC01P060852 |
| MIR455 | MicroRNA 455 | RNA Gene |  | 29 | GC09P114209 |
| SERPINB2 | Serpin Family B Member 2 | Protein Coding | P05120 | 56 | GC18P063871 |
| NBAS | NBAS Subunit Of NRZ Tethering Complex | Protein Coding | A2RRP1 | 52 | GC02M014800 |
| OPA1 | OPA1 Mitochondrial Dynamin Like GTPase | Protein Coding | O60313 | 56 | GC03P193594 |
| NCL | Nucleolin | Protein Coding | P19338 | 56 | GC02M231453 |
| DKK1 | Dickkopf WNT Signaling Pathway Inhibitor 1 | Protein Coding | O94907 | 56 | GC10P052314 |
| NAIP | NLR Family Apoptosis Inhibitory Protein | Protein Coding | Q13075 | 51 | GC05M070968 |
| HERC5 | HECT And RLD Domain Containing E3 Ubiquitin Protein Ligase 5 | Protein Coding | Q9UII4 | 50 | GC04P088457 |
| NKX2-1 | NK2 Homeobox 1 | Protein Coding | P43699 | 61 | GC14M036516 |
| PIAS1 | Protein Inhibitor Of Activated STAT 1 | Protein Coding | O75925 | 58 | GC15P068054 |
| MAP1LC3B | Microtubule Associated Protein 1 Light Chain 3 Beta | Protein Coding | Q9GZQ8 | 54 | GC16P106811 |
| TFE3 | Transcription Factor Binding To IGHM Enhancer 3 | Protein Coding | P19532 | 58 | GC0XM049028 |
| BMP7 | Bone Morphogenetic Protein 7 | Protein Coding | P18075 | 60 | GC20M057168 |
| ATP1A2 | ATPase Na+/K+ Transporting Subunit Alpha 2 | Protein Coding | P50993 | 61 | GC01P160115 |
| KIF11 | Kinesin Family Member 11 | Protein Coding | P52732 | 61 | GC10P092574 |
| MIR186 | MicroRNA 186 | RNA Gene |  | 28 | GC01M071067 |
| LYAR | Ly1 Antibody Reactive | Protein Coding | Q9NX58 | 48 | GC04M005814 |
| FCHO1 | FCH And Mu Domain Containing Endocytic Adaptor 1 | Protein Coding | O14526 | 48 | GC19P132606 |
| SLC5A7 | Solute Carrier Family 5 Member 7 | Protein Coding | Q9GZV3 | 55 | GC02P107969 |
| MIR574 | MicroRNA 574 | RNA Gene |  | 29 | GC04P039253 |
| SH2D1B | SH2 Domain Containing 1B | Protein Coding | O14796 | 47 | GC01M162395 |
| CCL28 | C-C Motif Chemokine Ligand 28 | Protein Coding | Q9NRJ3 | 48 | GC05M043356 |
| AZU1 | Azurocidin 1 | Protein Coding | P20160 | 51 | GC19P000825 |
| MCM3AP | Minichromosome Maintenance Complex Component 3 Associated Protein | Protein Coding | O60318 | 52 | GC21M046235 |
| UBE3A | Ubiquitin Protein Ligase E3A | Protein Coding | Q05086 | 61 | GC15M025333 |
| ALG3 | ALG3 Alpha-1,3- Mannosyltransferase | Protein Coding | Q92685 | 54 | GC03M184244 |
| FGF17 | Fibroblast Growth Factor 17 | Protein Coding | O60258 | 58 | GC08P024852 |
| ALDOA | Aldolase, Fructose-Bisphosphate A | Protein Coding | P04075 | 62 | GC16P030064 |
| ABCC2 | ATP Binding Cassette Subfamily C Member 2 | Protein Coding | Q92887 | 61 | GC10P099782 |
| H1-0 | H1.0 Linker Histone | Protein Coding | P07305 | 52 | GC22P081464 |
| CLEC5A | C-Type Lectin Domain Containing 5A | Protein Coding | Q9NY25 | 43 | GC07M141927 |
| LMX1B | LIM Homeobox Transcription Factor 1 Beta | Protein Coding | O60663 | 55 | GC09P146469 |
| CARD11-AS1 | CARD11 Antisense RNA 1 | RNA Gene |  | 20 | GC07P015278 |
| REST | RE1 Silencing Transcription Factor | Protein Coding | Q13127 | 55 | GC04P056907 |
| MCTS1 | MCTS1 Re-Initiation And Release Factor | Protein Coding | Q9ULC4 | 47 | GC0XP120594 |
| MUC7 | Mucin 7, Secreted | Protein Coding | Q8TAX7 | 47 | GC04P070430 |
| MIR423 | MicroRNA 423 | RNA Gene |  | 28 | GC17P030117 |
| PEX7 | Peroxisomal Biogenesis Factor 7 | Protein Coding | O00628 | 55 | GC06P136822 |
| GIMAP2 | GTPase, IMAP Family Member 2 | Protein Coding | Q9UG22 | 44 | GC07P161724 |
| TRAF7 | TNF Receptor Associated Factor 7 | Protein Coding | Q6Q0C0 | 52 | GC16P104056 |
| AGPAT2 | 1-Acylglycerol-3-Phosphate O-Acyltransferase 2 | Protein Coding | O15120 | 56 | GC09M136673 |
| ZFAS1 | ZNFX1 Antisense RNA 1 | RNA Gene |  | 29 | GC20P049276 |
| RALGAPA1 | Ral GTPase Activating Protein Catalytic Subunit Alpha 1 | Protein Coding | Q6GYQ0 | 53 | GC14M035538 |
| MIR138-1 | MicroRNA 138-1 | RNA Gene |  | 29 | GC03P044115 |
| MIR191 | MicroRNA 191 | RNA Gene |  | 30 | GC03M054249 |
| ATP6AP2 | ATPase H+ Transporting Accessory Protein 2 | Protein Coding | O75787 | 57 | GC0XP040634 |
| RELN | Reelin | Protein Coding | P78509 | 56 | GC07M103471 |
| EXT2 | Exostosin Glycosyltransferase 2 | Protein Coding | Q93063 | 57 | GC11P044095 |
| TRP-AGG2-6 | TRNA-Pro (Anticodon AGG) 2-6 | RNA Gene |  | 15 | GC14M027784 |
| MIR26A1 | MicroRNA 26a-1 | RNA Gene |  | 30 | GC03P037969 |
| GHRL | Ghrelin And Obestatin Prepropeptide | Protein Coding | Q9UBU3 | 54 | GC03M010285 |
| PLA2G2D | Phospholipase A2 Group IID | Protein Coding | Q9UNK4 | 47 | GC01M020111 |
| SEC61A1 | SEC61 Translocon Subunit Alpha 1 | Protein Coding | P61619 | 54 | GC03P128051 |
| DUOX1 | Dual Oxidase 1 | Protein Coding | Q9NRD9 | 54 | GC15P045129 |
| CD300A | CD300a Molecule | Protein Coding | Q9UGN4 | 48 | GC17P074466 |
| STAR | Steroidogenic Acute Regulatory Protein | Protein Coding | P49675 | 58 | GC08M038358 |
| FGF4 | Fibroblast Growth Factor 4 | Protein Coding | P08620 | 56 | GC11M132993 |
| CACNA1A | Calcium Voltage-Gated Channel Subunit Alpha1 A | Protein Coding | O00555 | 60 | GC19M013206 |
| MIR590 | MicroRNA 590 | RNA Gene |  | 29 | GC07P074191 |
| TWIST1 | Twist Family BHLH Transcription Factor 1 | Protein Coding | Q15672 | 59 | GC07M019020 |
| DNM1L | Dynamin 1 Like | Protein Coding | O00429 | 61 | GC12P032679 |
| TRIM6 | Tripartite Motif Containing 6 | Protein Coding | Q9C030 | 44 | GC11P005596 |
| DNAH8 | Dynein Axonemal Heavy Chain 8 | Protein Coding | Q96JB1 | 50 | GC06P156466 |
| DCTN1 | Dynactin Subunit 1 | Protein Coding | Q14203 | 59 | GC02M074361 |
| MPV17 | Mitochondrial Inner Membrane Protein MPV17 | Protein Coding | P39210 | 53 | GC02M027309 |
| BATF3 | Basic Leucine Zipper ATF-Like Transcription Factor 3 | Protein Coding | Q9NR55 | 46 | GC01M212686 |
| MIR374A | MicroRNA 374a | RNA Gene |  | 26 | GC0XM074300 |
| IGHV3-23 | Immunoglobulin Heavy Variable 3-23 | Protein Coding | P01764 | 23 | GC14M106268 |
| CD52 | CD52 Molecule | Protein Coding | P31358 | 50 | GC01P026317 |
| COX10 | Cytochrome C Oxidase Assembly Factor Heme A:Farnesyltransferase COX10 | Protein Coding | Q12887 | 55 | GC17P014069 |
| DCSTAMP | Dendrocyte Expressed Seven Transmembrane Protein | Protein Coding | Q9H295 | 46 | GC08P104339 |
| PON1 | Paraoxonase 1 | Protein Coding | P27169 | 60 | GC07M095297 |
| RNPC3 | RNA Binding Region (RNP1, RRM) Containing 3 | Protein Coding | Q96LT9 | 47 | GC01P103525 |
| SFTPA2 | Surfactant Protein A2 | Protein Coding | Q8IWL1 | 50 | GC10M084114 |
| BIN1 | Bridging Integrator 1 | Protein Coding | O00499 | 59 | GC02M127048 |
| CDH2 | Cadherin 2 | Protein Coding | P19022 | 63 | GC18M036283 |
| AGO2 | Argonaute RISC Catalytic Component 2 | Protein Coding | Q9UKV8 | 55 | GC08M140975 |
| OTC | Ornithine Transcarbamylase | Protein Coding | P00480 | 59 | GC0XP038345 |
| DSG3 | Desmoglein 3 | Protein Coding | P32926 | 53 | GC18P031447 |
| CRISP3 | Cysteine Rich Secretory Protein 3 | Protein Coding | P54108 | 56 | GC06M049696 |
| KCNAB2 | Potassium Voltage-Gated Channel Subfamily A Regulatory Beta Subunit 2 | Protein Coding | Q13303 | 54 | GC01P060005 |
| APLN | Apelin | Protein Coding | Q9ULZ1 | 48 | GC0XM129645 |
| DIABLO | Diablo IAP-Binding Mitochondrial Protein | Protein Coding | Q9NR28 | 59 | GC12M124304 |
| DGCR5 | DiGeorge Syndrome Critical Region Gene 5 | RNA Gene |  | 29 | GC22P080816 |
| HVCN1 | Hydrogen Voltage Gated Channel 1 | Protein Coding | Q96D96 | 49 | GC12M110627 |
| SI | Sucrase-Isomaltase | Protein Coding | P14410 | 57 | GC03M164978 |
| ANGPT1 | Angiopoietin 1 | Protein Coding | Q15389 | 58 | GC08M107246 |
| IL17RB | Interleukin 17 Receptor B | Protein Coding | Q9NRM6 | 52 | GC03P061700 |
| RLIM | Ring Finger Protein, LIM Domain Interacting | Protein Coding | Q9NVW2 | 50 | GC0XM074821 |
| SPTBN4 | Spectrin Beta, Non-Erythrocytic 4 | Protein Coding | Q9H254 | 52 | GC19P040466 |
| NFATC4 | Nuclear Factor Of Activated T Cells 4 | Protein Coding | Q14934 | 55 | GC14P024365 |
| HSPA1A | Heat Shock Protein Family A (Hsp70) Member 1A | Protein Coding | P0DMV8 | 59 | GC06P156262 |
| TACR1 | Tachykinin Receptor 1 | Protein Coding | P25103 | 58 | GC02M075743 |
| PSMC4 | Proteasome 26S Subunit, ATPase 4 | Protein Coding | P43686 | 54 | GC19P133034 |
| N4BP1 | NEDD4 Binding Protein 1 | Protein Coding | O75113 | 44 | GC16M048538 |
| PPP2R5C | Protein Phosphatase 2 Regulatory Subunit B'Gamma | Protein Coding | Q13362 | 54 | GC14P119153 |
| POLB | DNA Polymerase Beta | Protein Coding | P06746 | 59 | GC08P042338 |
| FFAR3 | Free Fatty Acid Receptor 3 | Protein Coding | O14843 | 47 | GC19P134074 |
| CNBP | CCHC-Type Zinc Finger Nucleic Acid Binding Protein | Protein Coding | P62633 | 55 | GC03M129167 |
| PLEK | Pleckstrin | Protein Coding | P08567 | 52 | GC02P068365 |
| PIGT | Phosphatidylinositol Glycan Anchor Biosynthesis Class T | Protein Coding | Q969N2 | 52 | GC20P045416 |
| TRAV24 | T Cell Receptor Alpha Variable 24 | Protein Coding | A0A0B4J272 | 16 | GC14P022105 |
| TRPV1 | Transient Receptor Potential Cation Channel Subfamily V Member 1 | Protein Coding | Q8NER1 | 61 | GC17M003565 |
| CYP1A1 | Cytochrome P450 Family 1 Subfamily A Member 1 | Protein Coding | P04798 | 59 | GC15M074719 |
| SIRT3 | Sirtuin 3 | Protein Coding | Q9NTG7 | 60 | GC11M000215 |
| NTF3 | Neurotrophin 3 | Protein Coding | P20783 | 56 | GC12P046718 |
| SCN11A | Sodium Voltage-Gated Channel Alpha Subunit 11 | Protein Coding | Q9UI33 | 55 | GC03M038862 |
| DUT | Deoxyuridine Triphosphatase | Protein Coding | P33316 | 55 | GC15P048331 |
| CDC25C | Cell Division Cycle 25C | Protein Coding | P30307 | 61 | GC05M138285 |
| SIRT2 | Sirtuin 2 | Protein Coding | Q8IXJ6 | 59 | GC19M038878 |
| MIR128-1 | MicroRNA 128-1 | RNA Gene |  | 27 | GC02P135665 |
| NFATC3 | Nuclear Factor Of Activated T Cells 3 | Protein Coding | Q12968 | 55 | GC16P068084 |
| GZMK | Granzyme K | Protein Coding | P49863 | 46 | GC05P055024 |
| NT5C3A | 5'-Nucleotidase, Cytosolic IIIA | Protein Coding | Q9H0P0 | 57 | GC07M033014 |
| TBX2 | T-Box Transcription Factor 2 | Protein Coding | Q13207 | 59 | GC17P061399 |
| TLN1 | Talin 1 | Protein Coding | Q9Y490 | 55 | GC09M035696 |
| TFF3 | Trefoil Factor 3 | Protein Coding | Q07654 | 53 | GC21M042311 |
| CLDN4 | Claudin 4 | Protein Coding | O14493 | 54 | GC07P073799 |
| TRIP12 | Thyroid Hormone Receptor Interactor 12 | Protein Coding | Q14669 | 54 | GC02M229763 |
| PPP2R1A | Protein Phosphatase 2 Scaffold Subunit Aalpha | Protein Coding | P30153 | 60 | GC19P133553 |
| FUT8 | Fucosyltransferase 8 | Protein Coding | Q9BYC5 | 56 | GC14P065356 |
| CTSH | Cathepsin H | Protein Coding | P09668 | 58 | GC15M155718 |
| SCGB1A1 | Secretoglobin Family 1A Member 1 | Protein Coding | P11684 | 54 | GC11P062405 |
| KIR2DS4 | Killer Cell Immunoglobulin Like Receptor, Two Ig Domains And Short Cytoplasmic Tail 4 | Protein Coding | P43632 | 42 | GC19P133685 |
| PPL | Periplakin | Protein Coding | O60437 | 50 | GC16M027123 |
| ACKR3 | Atypical Chemokine Receptor 3 | Protein Coding | P25106 | 53 | GC02P236537 |
| LALBA | Lactalbumin Alpha | Protein Coding | P00709 | 50 | GC12M048567 |
| TRIM2 | Tripartite Motif Containing 2 | Protein Coding | Q9C040 | 52 | GC04P153152 |
| ERVFRD-1 | Endogenous Retrovirus Group FRD Member 1, Envelope | Protein Coding | P60508 | 46 | GC06M011103 |
| PI4KA | Phosphatidylinositol 4-Kinase Alpha | Protein Coding | P42356 | 61 | GC22M020707 |
| ZNFX1 | Zinc Finger NFX1-Type Containing 1 | Protein Coding | Q9P2E3 | 43 | GC20M049800 |
| POSTN | Periostin | Protein Coding | Q15063 | 55 | GC13M037562 |
| TICAM2 | TIR Domain Containing Adaptor Molecule 2 | Protein Coding | Q86XR7 | 45 | GC05M115579 |
| CLDN3 | Claudin 3 | Protein Coding | O15551 | 54 | GC07M073768 |
| ACHE | Acetylcholinesterase (Yt Blood Group) | Protein Coding | P22303 | 61 | GC07M100889 |
| CASP5 | Caspase 5 | Protein Coding | P51878 | 55 | GC11M133543 |
| LCP1 | Lymphocyte Cytosolic Protein 1 | Protein Coding | P13796 | 54 | GC13M046132 |
| MATR3 | Matrin 3 | Protein Coding | P43243 | 55 | GC05P139274 |
| PLAA | Phospholipase A2 Activating Protein | Protein Coding | Q9Y263 | 56 | GC09M026903 |
| IPO7 | Importin 7 | Protein Coding | O95373 | 52 | GC11P009384 |
| LOC654780 | Splicing Factor Proline/Glutamine-Rich | RNA Gene |  | 23 | GC16M084193 |
| WDR19 | WD Repeat Domain 19 | Protein Coding | Q8NEZ3 | 50 | GC04P039272 |
| ITPR2 | Inositol 1,4,5-Trisphosphate Receptor Type 2 | Protein Coding | Q14571 | 57 | GC12M033324 |
| LILRB5 | Leukocyte Immunoglobulin Like Receptor B5 | Protein Coding | O75023 | 48 | GC19M101516 |
| AURKA | Aurora Kinase A | Protein Coding | O14965 | 65 | GC20M056370 |
| GPX3 | Glutathione Peroxidase 3 | Protein Coding | P22352 | 53 | GC05P155691 |
| FOSL1 | FOS Like 1, AP-1 Transcription Factor Subunit | Protein Coding | P15407 | 57 | GC11M132841 |
| ZFAT-AS1 | ZFAT Antisense RNA 1 | RNA Gene |  | 18 | GC08P134704 |
| TRBV17 | T Cell Receptor Beta Variable 17 (Non-Functional) | Protein Coding | A0A087X0K7 | 11 | GC07P163113 |
| RPS6KA3 | Ribosomal Protein S6 Kinase A3 | Protein Coding | P51812 | 65 | GC0XM020149 |
| CD72 | CD72 Molecule | Protein Coding | P21854 | 51 | GC09M035610 |
| RPTOR | Regulatory Associated Protein Of MTOR Complex 1 | Protein Coding | Q8N122 | 56 | GC17P080544 |
| PRSS2 | Serine Protease 2 | Protein Coding | P07478 | 54 | GC07P161569 |
| MGMT | O-6-Methylguanine-DNA Methyltransferase | Protein Coding | P16455 | 62 | GC10P129467 |
| ECSIT | ECSIT Signaling Integrator | Protein Coding | Q9BQ95 | 50 | GC19M011505 |
| MYDGF | Myeloid Derived Growth Factor | Protein Coding | Q969H8 | 45 | GC19M004641 |
| FNIP1 | Folliculin Interacting Protein 1 | Protein Coding | Q8TF40 | 50 | GC05M131641 |
| FGF14 | Fibroblast Growth Factor 14 | Protein Coding | Q92915 | 56 | GC13M101710 |
| PF4V1 | Platelet Factor 4 Variant 1 | Protein Coding | P10720 | 47 | GC04P073853 |
| ULBP3 | UL16 Binding Protein 3 | Protein Coding | Q9BZM4 | 47 | GC06M150062 |
| POU5F1 | POU Class 5 Homeobox 1 | Protein Coding | Q01860 | 59 | GC06M100249 |
| IL31RA | Interleukin 31 Receptor A | Protein Coding | Q8NI17 | 55 | GC05P055840 |
| PSMC1 | Proteasome 26S Subunit, ATPase 1 | Protein Coding | P62191 | 55 | GC14P090256 |
| PDE4A | Phosphodiesterase 4A | Protein Coding | P27815 | 56 | GC19P010416 |
| MED25 | Mediator Complex Subunit 25 | Protein Coding | Q71SY5 | 51 | GC19P133439 |
| ZCCHC3 | Zinc Finger CCHC-Type Containing 3 | Protein Coding | Q9NUD5 | 41 | GC20P000297 |
| GIMAP8 | GTPase, IMAP Family Member 8 | Protein Coding | Q8ND71 | 41 | GC07P150450 |
| SPTLC1 | Serine Palmitoyltransferase Long Chain Base Subunit 1 | Protein Coding | O15269 | 58 | GC09M118059 |
| DIS3 | DIS3 Homolog, Exosome Endoribonuclease And 3'-5' Exoribonuclease | Protein Coding | Q9Y2L1 | 52 | GC13M072752 |
| TBC1D23 | TBC1 Domain Family Member 23 | Protein Coding | Q9NUY8 | 50 | GC03P100260 |
| ERBB3 | Erb-B2 Receptor Tyrosine Kinase 3 | Protein Coding | P21860 | 67 | GC12P065362 |
| HRH4 | Histamine Receptor H4 | Protein Coding | Q9H3N8 | 55 | GC18P024460 |
| PSMB11 | Proteasome Subunit Beta 11 | Protein Coding | A5LHX3 | 43 | GC14P054019 |
| SEMA7A | Semaphorin 7A (JohnMiltonHagen Blood Group) | Protein Coding | O75326 | 56 | GC15M074409 |
| LY86 | Lymphocyte Antigen 86 | Protein Coding | O95711 | 49 | GC06P006588 |
| ENPP2 | Ectonucleotide Pyrophosphatase/Phosphodiesterase 2 | Protein Coding | Q13822 | 57 | GC08M119556 |
| CYP2E1 | Cytochrome P450 Family 2 Subfamily E Member 1 | Protein Coding | P05181 | 58 | GC10P133520 |
| MIR181B1 | MicroRNA 181b-1 | RNA Gene |  | 28 | GC01M198858 |
| CABIN1 | Calcineurin Binding Protein 1 | Protein Coding | Q9Y6J0 | 53 | GC22P024011 |
| SOST | Sclerostin | Protein Coding | Q9BQB4 | 55 | GC17M043753 |
| ATP6V1B2 | ATPase H+ Transporting V1 Subunit B2 | Protein Coding | P21281 | 59 | GC08P020197 |
| JAM3 | Junctional Adhesion Molecule 3 | Protein Coding | Q9BX67 | 55 | GC11P134083 |
| PPARGC1A | PPARG Coactivator 1 Alpha | Protein Coding | Q9UBK2 | 59 | GC04M023755 |
| MIR135A1 | MicroRNA 135a-1 | RNA Gene |  | 29 | GC03M054393 |
| RAD21 | RAD21 Cohesin Complex Component | Protein Coding | O60216 | 57 | GC08M116846 |
| CKB | Creatine Kinase B | Protein Coding | P12277 | 57 | GC14M103519 |
| GAN | Gigaxonin | Protein Coding | Q9H2C0 | 52 | GC16P106376 |
| TRG | T Cell Receptor Gamma Locus | Protein Coding |  | 20 | GC07M038240 |
| KRIT1 | KRIT1 Ankyrin Repeat Containing | Protein Coding | O00522 | 54 | GC07M092198 |
| GNS | Glucosamine (N-Acetyl)-6-Sulfatase | Protein Coding | P15586 | 57 | GC12M064713 |
| MIR485 | MicroRNA 485 | RNA Gene |  | 26 | GC14P119073 |
| TMED1 | Transmembrane P24 Trafficking Protein 1 | Protein Coding | Q13445 | 48 | GC19M010832 |
| PXK | PX Domain Containing Serine/Threonine Kinase Like | Protein Coding | Q7Z7A4 | 49 | GC03P061821 |
| PPP3CB | Protein Phosphatase 3 Catalytic Subunit Beta | Protein Coding | P16298 | 57 | GC10M073436 |
| H2AC18 | H2A Clustered Histone 18 | Protein Coding | Q6FI13 | 41 | GC01M164851 |
| BUB3 | BUB3 Mitotic Checkpoint Protein | Protein Coding | O43684 | 59 | GC10P123154 |
| SIGMAR1 | Sigma Non-Opioid Intracellular Receptor 1 | Protein Coding | Q99720 | 58 | GC09M034634 |
| ALMS1 | ALMS1 Centrosome And Basal Body Associated Protein | Protein Coding | Q8TCU4 | 51 | GC02P073385 |
| CENPB | Centromere Protein B | Protein Coding | P07199 | 50 | GC20M003783 |
| SLC7A11 | Solute Carrier Family 7 Member 11 | Protein Coding | Q9UPY5 | 57 | GC04M138164 |
| IGFBP1 | Insulin Like Growth Factor Binding Protein 1 | Protein Coding | P08833 | 55 | GC07P050144 |
| CWF19L1 | CWF19 Like Cell Cycle Control Factor 1 | Protein Coding | Q69YN2 | 50 | GC10M100232 |
| CEACAM8 | CEA Cell Adhesion Molecule 8 | Protein Coding | P31997 | 51 | GC19M042580 |
| S100A4 | S100 Calcium Binding Protein A4 | Protein Coding | P26447 | 57 | GC01M153543 |
| NFIL3 | Nuclear Factor, Interleukin 3 Regulated | Protein Coding | Q16649 | 48 | GC09M091409 |
| NPHS2 | NPHS2 Stomatin Family Member, Podocin | Protein Coding | Q9NP85 | 54 | GC01M179554 |
| TMCO1 | Transmembrane And Coiled-Coil Domains 1 | Protein Coding | Q9UM00 | 50 | GC01M165724 |
| RALA | RAS Like Proto-Oncogene A | Protein Coding | P11233 | 59 | GC07P039622 |
| GLS | Glutaminase | Protein Coding | O94925 | 62 | GC02P190880 |
| POLR3E | RNA Polymerase III Subunit E | Protein Coding | Q9NVU0 | 47 | GC16P104600 |
| IGHG4 | Immunoglobulin Heavy Constant Gamma 4 (G4m Marker) | Protein Coding | P01861 | 39 | GC14M122253 |
| IFI44L | Interferon Induced Protein 44 Like | Protein Coding | Q53G44 | 44 | GC01P078619 |
| AP4B1-AS1 | AP4B1 Antisense RNA 1 | RNA Gene |  | 23 | GC01P113837 |
| ZDHHC1 | Zinc Finger DHHC-Type Containing 1 | Protein Coding | Q8WTX9 | 43 | GC16M067394 |
| PQBP1 | Polyglutamine Binding Protein 1 | Protein Coding | O60828 | 51 | GC0XP048890 |
| KRT19 | Keratin 19 | Protein Coding | P08727 | 56 | GC17M041523 |
| IGLL5 | Immunoglobulin Lambda Like Polypeptide 5 | Protein Coding | B9A064 | 41 | GC22P081015 |
| PRKACG | Protein Kinase CAMP-Activated Catalytic Subunit Gamma | Protein Coding | P22612 | 58 | GC09M071422 |
| ELF1 | E74 Like ETS Transcription Factor 1 | Protein Coding | P32519 | 52 | GC13M041721 |
| KDM6B | Lysine Demethylase 6B | Protein Coding | O15054 | 57 | GC17P007834 |
| SLC7A5 | Solute Carrier Family 7 Member 5 | Protein Coding | Q01650 | 56 | GC16M087830 |
| EXT1 | Exostosin Glycosyltransferase 1 | Protein Coding | Q16394 | 59 | GC08M117798 |
| MIR330 | MicroRNA 330 | RNA Gene |  | 30 | GC19M101140 |
| ERV3-1 | Endogenous Retrovirus Group 3 Member 1, Envelope | Protein Coding | Q14264 | 45 | GC07M072466 |
| KPNA2 | Karyopherin Subunit Alpha 2 | Protein Coding | P52292 | 58 | GC17P068035 |
| DIS3L2 | DIS3 Like 3'-5' Exoribonuclease 2 | Protein Coding | Q8IYB7 | 54 | GC02P231961 |
| VDAC1 | Voltage Dependent Anion Channel 1 | Protein Coding | P21796 | 58 | GC05M133975 |
| CMTM6 | CKLF Like MARVEL Transmembrane Domain Containing 6 | Protein Coding | Q9NX76 | 41 | GC03M032499 |
| RAB5A | RAB5A, Member RAS Oncogene Family | Protein Coding | P20339 | 57 | GC03P024432 |
| MGAM | Maltase-Glucoamylase | Protein Coding | O43451 | 56 | GC07P162878 |
| PEX5 | Peroxisomal Biogenesis Factor 5 | Protein Coding | P50542 | 57 | GC12P046803 |
| EIF2AK4 | Eukaryotic Translation Initiation Factor 2 Alpha Kinase 4 | Protein Coding | Q9P2K8 | 57 | GC15P039934 |
| SPHK2 | Sphingosine Kinase 2 | Protein Coding | Q9NRA0 | 56 | GC19P048619 |
| TUBA1A | Tubulin Alpha 1a | Protein Coding | Q71U36 | 61 | GC12M049184 |
| SLC2A4 | Solute Carrier Family 2 Member 4 | Protein Coding | P14672 | 59 | GC17P132635 |
| CFL2 | Cofilin 2 | Protein Coding | Q9Y281 | 56 | GC14M034706 |
| NOX1 | NADPH Oxidase 1 | Protein Coding | Q9Y5S8 | 56 | GC0XM100843 |
| SNRNP70 | Small Nuclear Ribonucleoprotein U1 Subunit 70 | Protein Coding | P08621 | 51 | GC19P049085 |
| MIR16-2 | MicroRNA 16-2 | RNA Gene |  | 28 | GC03P160418 |
| F10 | Coagulation Factor X | Protein Coding | P00742 | 63 | GC13P113122 |
| MIR369 | MicroRNA 369 | RNA Gene |  | 28 | GC14P119058 |
| FGF22 | Fibroblast Growth Factor 22 | Protein Coding | Q9HCT0 | 49 | GC19P000639 |
| IKZF2 | IKAROS Family Zinc Finger 2 | Protein Coding | Q9UKS7 | 50 | GC02M213001 |
| DPEP1 | Dipeptidase 1 | Protein Coding | P16444 | 56 | GC16P089613 |
| CLDN7 | Claudin 7 | Protein Coding | O95471 | 52 | GC17M007259 |
| CYP2C9 | Cytochrome P450 Family 2 Subfamily C Member 9 | Protein Coding | P11712 | 59 | GC10P094938 |
| MMEL1 | Membrane Metalloendopeptidase Like 1 | Protein Coding | Q495T6 | 49 | GC01M002590 |
| ANK1 | Ankyrin 1 | Protein Coding | P16157 | 58 | GC08M041653 |
| ORAI2 | ORAI Calcium Release-Activated Calcium Modulator 2 | Protein Coding | Q96SN7 | 48 | GC07P102433 |
| PADI2 | Peptidyl Arginine Deiminase 2 | Protein Coding | Q9Y2J8 | 55 | GC01M017066 |
| SLC25A13 | Solute Carrier Family 25 Member 13 | Protein Coding | Q9UJS0 | 58 | GC07M096120 |
| C4B_2 | Complement Component 4B (Chido/Rodgers Blood Group), Copy 2 | Protein Coding | P0C0L5 | 25 | GC06Po03283 |
| SLC17A5 | Solute Carrier Family 17 Member 5 | Protein Coding | Q9NRA2 | 57 | GC06M073593 |
| CRTAM | Cytotoxic And Regulatory T Cell Molecule | Protein Coding | O95727 | 40 | GC11P123711 |
| MFGE8 | Milk Fat Globule EGF And Factor V/VIII Domain Containing | Protein Coding | Q08431 | 55 | GC15M088898 |
| VRK1 | VRK Serine/Threonine Kinase 1 | Protein Coding | Q99986 | 60 | GC14P096797 |
| KLKB1 | Kallikrein B1 | Protein Coding | P03952 | 58 | GC04P186395 |
| LMNB2 | Lamin B2 | Protein Coding | Q03252 | 55 | GC19M099961 |
| SELENOK | Selenoprotein K | Protein Coding | Q9Y6D0 | 40 | GC03M053884 |
| AREG | Amphiregulin | Protein Coding | P15514 | 55 | GC04P074445 |
| RNF217 | Ring Finger Protein 217 | Protein Coding | Q8TC41 | 42 | GC06P124962 |
| PIK3R3 | Phosphoinositide-3-Kinase Regulatory Subunit 3 | Protein Coding | Q92569 | 55 | GC01M046041 |
| SIGLEC15 | Sialic Acid Binding Ig Like Lectin 15 | Protein Coding | Q6ZMC9 | 49 | GC18P045825 |
| GAST | Gastrin | Protein Coding | P01350 | 52 | GC17P041712 |
| CEACAM6 | CEA Cell Adhesion Molecule 6 | Protein Coding | P40199 | 53 | GC19P041750 |
| TSC22D3 | TSC22 Domain Family Member 3 | Protein Coding | Q99576 | 51 | GC0XM107713 |
| F2R | Coagulation Factor II Thrombin Receptor | Protein Coding | P25116 | 58 | GC05P076716 |
| MIR339 | MicroRNA 339 | RNA Gene |  | 30 | GC07M001022 |
| WDR11 | WD Repeat Domain 11 | Protein Coding | Q9BZH6 | 50 | GC10P120851 |
| EME2 | Essential Meiotic Structure-Specific Endonuclease Subunit 2 | Protein Coding | A4GXA9 | 44 | GC16P104038 |
| UBE2I | Ubiquitin Conjugating Enzyme E2 I | Protein Coding | P63279 | 59 | GC16P104002 |
| B4GALNT1 | Beta-1,4-N-Acetyl-Galactosaminyltransferase 1 | Protein Coding | Q00973 | 58 | GC12M057623 |
| GPHN | Gephyrin | Protein Coding | Q9NQX3 | 61 | GC14P066507 |
| SLC9A3 | Solute Carrier Family 9 Member A3 | Protein Coding | P48764 | 59 | GC05M000472 |
| IQGAP1 | IQ Motif Containing GTPase Activating Protein 1 | Protein Coding | P46940 | 55 | GC15P090388 |
| IGF2R | Insulin Like Growth Factor 2 Receptor | Protein Coding | P11717 | 59 | GC06P159969 |
| COG5 | Component Of Oligomeric Golgi Complex 5 | Protein Coding | Q9UP83 | 51 | GC07M107201 |
| SLC30A5 | Solute Carrier Family 30 Member 5 | Protein Coding | Q8TAD4 | 51 | GC05P069093 |
| AIF1 | Allograft Inflammatory Factor 1 | Protein Coding | P55008 | 51 | GC06P156243 |
| MIR185 | MicroRNA 185 | RNA Gene |  | 31 | GC22P080847 |
| MARCO | Macrophage Receptor With Collagenous Structure | Protein Coding | Q9UEW3 | 47 | GC02P118942 |
| CSN2 | Casein Beta | Protein Coding | P05814 | 45 | GC04M069955 |
| TSPAN32 | Tetraspanin 32 | Protein Coding | Q96QS1 | 44 | GC11P014106 |
| ARRB2 | Arrestin Beta 2 | Protein Coding | P32121 | 57 | GC17P132513 |
| BCHE | Butyrylcholinesterase | Protein Coding | P06276 | 62 | GC03M165772 |
| EHMT1 | Euchromatic Histone Lysine Methyltransferase 1 | Protein Coding | Q9H9B1 | 58 | GC09P137618 |
| HULC | Hepatocellular Carcinoma Up-Regulated Long Non-Coding RNA | RNA Gene |  | 29 | GC06P008703 |
| HHLA2 | HHLA2 Member Of B7 Family | Protein Coding | Q9UM44 | 44 | GC03P108296 |
| ADCY10 | Adenylate Cyclase 10 | Protein Coding | Q96PN6 | 55 | GC01M167809 |
| COG4 | Component Of Oligomeric Golgi Complex 4 | Protein Coding | Q9H9E3 | 51 | GC16M073853 |
| GPX1 | Glutathione Peroxidase 1 | Protein Coding | P07203 | 56 | GC03M054269 |
| EIF2S1 | Eukaryotic Translation Initiation Factor 2 Subunit Alpha | Protein Coding | P05198 | 57 | GC14P067359 |
| MAP3K8 | Mitogen-Activated Protein Kinase Kinase Kinase 8 | Protein Coding | P41279 | 59 | GC10P030915 |
| MOCS3 | Molybdenum Cofactor Synthesis 3 | Protein Coding | O95396 | 48 | GC20P050958 |
| NLRP2 | NLR Family Pyrin Domain Containing 2 | Protein Coding | Q9NX02 | 55 | GC19P054953 |
| SPARC | Secreted Protein Acidic And Cysteine Rich | Protein Coding | P09486 | 62 | GC05M151661 |
| MYO9B | Myosin IXB | Protein Coding | Q13459 | 55 | GC19P132592 |
| GNB4 | G Protein Subunit Beta 4 | Protein Coding | Q9HAV0 | 54 | GC03M179397 |
| NIPA1 | NIPA Magnesium Transporter 1 | Protein Coding | Q7RTP0 | 50 | GC15P022773 |
| RAET1E | Retinoic Acid Early Transcript 1E | Protein Coding | Q8TD07 | 44 | GC06M149883 |
| ARPC5 | Actin Related Protein 2/3 Complex Subunit 5 | Protein Coding | O15511 | 52 | GC01M186361 |
| CXCL14 | C-X-C Motif Chemokine Ligand 14 | Protein Coding | O95715 | 48 | GC05M135617 |
| SF3B1 | Splicing Factor 3b Subunit 1 | Protein Coding | O75533 | 57 | GC02M197453 |
| TNPO3 | Transportin 3 | Protein Coding | Q9Y5L0 | 53 | GC07M128954 |
| GIMAP7 | GTPase, IMAP Family Member 7 | Protein Coding | Q8NHV1 | 40 | GC07P150514 |
| TRC-GCA24-1 | TRNA-Cys (GCA) 24-1 | RNA Gene |  | 15 | GC17M089223 |
| S1PR4 | Sphingosine-1-Phosphate Receptor 4 | Protein Coding | O95977 | 51 | GC19P131936 |
| TFAM | Transcription Factor A, Mitochondrial | Protein Coding | Q00059 | 57 | GC10P058385 |
| RNF170 | Ring Finger Protein 170 | Protein Coding | Q96K19 | 48 | GC08M044377 |
| MAP2K4 | Mitogen-Activated Protein Kinase Kinase 4 | Protein Coding | P45985 | 62 | GC17P012020 |
| S100A1 | S100 Calcium Binding Protein A1 | Protein Coding | P23297 | 51 | GC01P153627 |
| MKS1 | MKS Transition Zone Complex Subunit 1 | Protein Coding | Q9NXB0 | 52 | GC17M058205 |
| PRKD1 | Protein Kinase D1 | Protein Coding | Q15139 | 62 | GC14M029576 |
| TMEM165 | Transmembrane Protein 165 | Protein Coding | Q9HC07 | 51 | GC04P055395 |
| KIAA0319L | KIAA0319 Like | Protein Coding | Q8IZA0 | 48 | GC01M035393 |
| SLC6A3 | Solute Carrier Family 6 Member 3 | Protein Coding | Q01959 | 63 | GC05M001392 |
| CLEC9A | C-Type Lectin Domain Containing 9A | Protein Coding | Q6UXN8 | 43 | GC12P010030 |
| ALPP | Alkaline Phosphatase, Placental | Protein Coding | P05187 | 56 | GC02P232378 |
| H3C1 | H3 Clustered Histone 1 | Protein Coding | P68431 | 50 | GC06P156030 |
| MCOLN1 | Mucolipin TRP Cation Channel 1 | Protein Coding | Q9GZU1 | 55 | GC19P132117 |
| SEPTIN9 | Septin 9 | Protein Coding | Q9UHD8 | 53 | GC17P134677 |
| CCNT1 | Cyclin T1 | Protein Coding | O60563 | 51 | GC12M048688 |
| SLC46A2 | Solute Carrier Family 46 Member 2 | Protein Coding | Q9BY10 | 42 | GC09M112878 |
| NUP133 | Nucleoporin 133 | Protein Coding | Q8WUM0 | 54 | GC01M230432 |
| SHANK3 | SH3 And Multiple Ankyrin Repeat Domains 3 | Protein Coding | Q9BYB0 | 51 | GC22P081853 |
| TRRAP | Transformation/Transcription Domain Associated Protein | Protein Coding | Q9Y4A5 | 57 | GC07P098877 |
| AQP3 | Aquaporin 3 (Gill Blood Group) | Protein Coding | Q92482 | 58 | GC09M033431 |
| SLC16A1 | Solute Carrier Family 16 Member 1 | Protein Coding | P53985 | 61 | GC01M113130 |
| DHFR2 | Dihydrofolate Reductase 2 | Protein Coding | Q86XF0 | 45 | GC03M094048 |
| MIR199B | MicroRNA 199b | RNA Gene |  | 29 | GC09M128244 |
| CAMK2B | Calcium/Calmodulin Dependent Protein Kinase II Beta | Protein Coding | Q13554 | 62 | GC07M045513 |
| DHX36 | DEAH-Box Helicase 36 | Protein Coding | Q9H2U1 | 52 | GC03M154272 |
| FGF13 | Fibroblast Growth Factor 13 | Protein Coding | Q92913 | 55 | GC0XM138615 |
| MIR215 | MicroRNA 215 | RNA Gene |  | 27 | GC01M220117 |
| MIR301A | MicroRNA 301a | RNA Gene |  | 29 | GC17M059151 |
| STXBP1 | Syntaxin Binding Protein 1 | Protein Coding | P61764 | 61 | GC09P146576 |
| DPP10 | Dipeptidyl Peptidase Like 10 | Protein Coding | Q8N608 | 55 | GC02P114442 |
| PHGDH | Phosphoglycerate Dehydrogenase | Protein Coding | O43175 | 63 | GC01P119674 |
| HCRT | Hypocretin Neuropeptide Precursor | Protein Coding | O43612 | 52 | GC17M089358 |
| MTAP | Methylthioadenosine Phosphorylase | Protein Coding | Q13126 | 62 | GC09P021887 |
| PSMD12 | Proteasome 26S Subunit, Non-ATPase 12 | Protein Coding | O00232 | 54 | GC17M067337 |
| CNTN2 | Contactin 2 | Protein Coding | Q02246 | 58 | GC01P205043 |
| MIR7-1 | MicroRNA 7-1 | RNA Gene |  | 25 | GC09M112905 |
| RPGRIP1L | RPGRIP1 Like | Protein Coding | Q68CZ1 | 54 | GC16M053759 |
| LOC126863274 | MED14-Independent Group 3 Enhancer GRCh37_chrX:70330888-70332087 | Functional Element |  | 8 | GC0XP071126 |
| MIR520A | MicroRNA 520a | RNA Gene |  | 26 | GC19P053690 |
| SPAST | Spastin | Protein Coding | Q9UBP0 | 54 | GC02P032063 |
| IRS2 | Insulin Receptor Substrate 2 | Protein Coding | Q9Y4H2 | 58 | GC13M109752 |
| RASA2 | RAS P21 Protein Activator 2 | Protein Coding | Q15283 | 51 | GC03P141487 |
| EDNRB | Endothelin Receptor Type B | Protein Coding | P24530 | 62 | GC13M077895 |
| DUSP6 | Dual Specificity Phosphatase 6 | Protein Coding | Q16828 | 60 | GC12M089347 |
| CRHR1 | Corticotropin Releasing Hormone Receptor 1 | Protein Coding | P34998 | 56 | GC17P045784 |
| GCLC | Glutamate-Cysteine Ligase Catalytic Subunit | Protein Coding | P48506 | 58 | GC06M053497 |
| ELK1 | ETS Transcription Factor ELK1 | Protein Coding | P19419 | 58 | GC0XM047635 |
| ATXN2 | Ataxin 2 | Protein Coding | Q99700 | 55 | GC12M111443 |
| ATP6V0A1 | ATPase H+ Transporting V0 Subunit A1 | Protein Coding | Q93050 | 55 | GC17P042458 |
| ALCAM | Activated Leukocyte Cell Adhesion Molecule | Protein Coding | Q13740 | 54 | GC03P105366 |
| SLC19A2 | Solute Carrier Family 19 Member 2 | Protein Coding | O60779 | 57 | GC01M169463 |
| FGF3 | Fibroblast Growth Factor 3 | Protein Coding | P11487 | 57 | GC11M132994 |
| ELMO1 | Engulfment And Cell Motility 1 | Protein Coding | Q92556 | 52 | GC07M036860 |
| AHI1 | Abelson Helper Integration Site 1 | Protein Coding | Q8N157 | 53 | GC06M135283 |
| SIGLEC5 | Sialic Acid Binding Ig Like Lectin 5 | Protein Coding | O15389 | 48 | GC19M101414 |
| SPG11 | SPG11 Vesicle Trafficking Associated, Spatacsin | Protein Coding | Q96JI7 | 52 | GC15M045104 |
| S1PR5 | Sphingosine-1-Phosphate Receptor 5 | Protein Coding | Q9H228 | 55 | GC19M010512 |
| DMXL2 | Dmx Like 2 | Protein Coding | Q8TDJ6 | 48 | GC15M051447 |
| SOX17 | SRY-Box Transcription Factor 17 | Protein Coding | Q9H6I2 | 55 | GC08P054457 |
| HPX | Hemopexin | Protein Coding | P02790 | 54 | GC11M013508 |
| ABCE1 | ATP Binding Cassette Subfamily E Member 1 | Protein Coding | P61221 | 51 | GC04P145097 |
| GPX2 | Glutathione Peroxidase 2 | Protein Coding | P18283 | 54 | GC14M064939 |
| TUBB | Tubulin Beta Class I | Protein Coding | P07437 | 62 | GC06P156224 |
| MIR28 | MicroRNA 28 | RNA Gene |  | 27 | GC03P188688 |
| CCDC107 | Coiled-Coil Domain Containing 107 | Protein Coding | Q8WV48 | 41 | GC09P035658 |
| IGFBP3 | Insulin Like Growth Factor Binding Protein 3 | Protein Coding | P17936 | 59 | GC07M045912 |
| CNTNAP2 | Contactin Associated Protein 2 | Protein Coding | Q9UHC6 | 58 | GC07P146116 |
| CIB1 | Calcium And Integrin Binding 1 | Protein Coding | Q99828 | 54 | GC15M090229 |
| RNASE7 | Ribonuclease A Family Member 7 | Protein Coding | Q9H1E1 | 45 | GC14P021042 |
| MPDU1 | Mannose-P-Dolichol Utilization Defect 1 | Protein Coding | O75352 | 54 | GC17P007583 |
| TRIM27 | Tripartite Motif Containing 27 | Protein Coding | P14373 | 52 | GC06M028903 |
| MUC4 | Mucin 4, Cell Surface Associated | Protein Coding | Q99102 | 52 | GC03M195746 |
| FGF1 | Fibroblast Growth Factor 1 | Protein Coding | P05230 | 59 | GC05M142555 |
| ACAA1 | Acetyl-CoA Acyltransferase 1 | Protein Coding | P09110 | 54 | GC03M038103 |
| WEE1 | WEE1 G2 Checkpoint Kinase | Protein Coding | P30291 | 61 | GC11P009573 |
| RNF115 | Ring Finger Protein 115 | Protein Coding | Q9Y4L5 | 43 | GC01M145738 |
| PIGN | Phosphatidylinositol Glycan Anchor Biosynthesis Class N | Protein Coding | O95427 | 52 | GC18M061905 |
| FLI1 | Fli-1 Proto-Oncogene, ETS Transcription Factor | Protein Coding | Q01543 | 60 | GC11P128686 |
| GAS6 | Growth Arrest Specific 6 | Protein Coding | Q14393 | 56 | GC13M113820 |
| H3-4 | H3.4 Histone, Cluster Member | Protein Coding | Q16695 | 51 | GC01M230370 |
| MIR345 | MicroRNA 345 | RNA Gene |  | 28 | GC14P100307 |
| IFN1@ | Interferon, Type 1, Cluster | Gene Cluster |  | 6 | GC09U990039 |
| DHCR7 | 7-Dehydrocholesterol Reductase | Protein Coding | Q9UBM7 | 61 | GC11M071428 |
| PROM1 | Prominin 1 | Protein Coding | O43490 | 59 | GC04M015965 |
| UBE2V1 | Ubiquitin Conjugating Enzyme E2 V1 | Protein Coding | Q13404 | 54 | GC20M050082 |
| IRF6 | Interferon Regulatory Factor 6 | Protein Coding | O14896 | 55 | GC01M209785 |
| DYRK1A | Dual Specificity Tyrosine Phosphorylation Regulated Kinase 1A | Protein Coding | Q13627 | 63 | GC21P037365 |
| TRAFD1 | TRAF-Type Zinc Finger Domain Containing 1 | Protein Coding | O14545 | 45 | GC12P112125 |
| GBP7 | Guanylate Binding Protein 7 | Protein Coding | Q8N8V2 | 42 | GC01M089131 |
| GOLPH3 | Golgi Phosphoprotein 3 | Protein Coding | Q9H4A6 | 51 | GC05M032124 |
| DCN | Decorin | Protein Coding | P07585 | 59 | GC12M091140 |
| ARF1 | ADP Ribosylation Factor 1 | Protein Coding | P84077 | 58 | GC01P228082 |
| RNU7-1 | RNA, U7 Small Nuclear 1 | RNA Gene |  | 22 | GC12P046795 |
| SMARCA2 | SWI/SNF Related, Matrix Associated, Actin Dependent Regulator Of Chromatin, Subfamily A, Member 2 | Protein Coding | P51531 | 62 | GC09P001980 |
| H2AC20 | H2A Clustered Histone 20 | Protein Coding | Q16777 | 49 | GC01P170165 |
| ATF6 | Activating Transcription Factor 6 | Protein Coding | P18850 | 61 | GC01P161766 |
| WFS1 | Wolframin ER Transmembrane Glycoprotein | Protein Coding | O76024 | 58 | GC04P006269 |
| LRG1 | Leucine Rich Alpha-2-Glycoprotein 1 | Protein Coding | P02750 | 50 | GC19M100034 |
| MIR381 | MicroRNA 381 | RNA Gene |  | 27 | GC14P119066 |
| HLTF | Helicase Like Transcription Factor | Protein Coding | Q14527 | 52 | GC03M149030 |
| GALT | Galactose-1-Phosphate Uridylyltransferase | Protein Coding | P07902 | 59 | GC09P060027 |
| FUBP1 | Far Upstream Element Binding Protein 1 | Protein Coding | Q96AE4 | 51 | GC01M077944 |
| MLKL | Mixed Lineage Kinase Domain Like Pseudokinase | Protein Coding | Q8NB16 | 55 | GC16M074892 |
| GGT1 | Gamma-Glutamyltransferase 1 | Protein Coding | P19440 | 62 | GC22P024583 |
| TP63 | Tumor Protein P63 | Protein Coding | Q9H3D4 | 58 | GC03P189598 |
| MYH6 | Myosin Heavy Chain 6 | Protein Coding | P13533 | 59 | GC14M023381 |
| GIMAP1 | GTPase, IMAP Family Member 1 | Protein Coding | Q8WWP7 | 40 | GC07P161726 |
| FUCA1 | Alpha-L-Fucosidase 1 | Protein Coding | P04066 | 61 | GC01M023845 |
| MYO5B | Myosin VB | Protein Coding | Q9ULV0 | 54 | GC18M049822 |
| JAZF1 | JAZF Zinc Finger 1 | Protein Coding | Q86VZ6 | 49 | GC07M027830 |
| CYP21A2 | Cytochrome P450 Family 21 Subfamily A Member 2 | Protein Coding | P08686 | 55 | GC06P156268 |
| ENO2 | Enolase 2 | Protein Coding | P09104 | 60 | GC12P006913 |
| PMP2 | Peripheral Myelin Protein 2 | Protein Coding | P02689 | 54 | GC08M081440 |
| POMP | Proteasome Maturation Protein | Protein Coding | Q9Y244 | 51 | GC13P028659 |
| PROCR | Protein C Receptor | Protein Coding | Q9UNN8 | 54 | GC20P035171 |
| RBBP8 | RB Binding Protein 8, Endonuclease | Protein Coding | Q99708 | 56 | GC18P022798 |
| CUL3 | Cullin 3 | Protein Coding | Q13618 | 61 | GC02M224470 |
| FAT1 | FAT Atypical Cadherin 1 | Protein Coding | Q14517 | 52 | GC04M186587 |
| YTHDF2 | YTH N6-Methyladenosine RNA Binding Protein F2 | Protein Coding | Q9Y5A9 | 49 | GC01P061385 |
| PPOX | Protoporphyrinogen Oxidase | Protein Coding | P50336 | 56 | GC01P170788 |
| ERF | ETS2 Repressor Factor | Protein Coding | P50548 | 52 | GC19M042247 |
| LINC01554 | Long Intergenic Non-Protein Coding RNA 1554 | RNA Gene | Q52M75 | 31 | GC05P095838 |
| SLC2A2 | Solute Carrier Family 2 Member 2 | Protein Coding | P11168 | 62 | GC03M170996 |
| DPYD | Dihydropyrimidine Dehydrogenase | Protein Coding | Q12882 | 64 | GC01M097015 |
| MYOM2 | Myomesin 2 | Protein Coding | P54296 | 50 | GC08P002045 |
| TTC4 | Tetratricopeptide Repeat Domain 4 | Protein Coding | O95801 | 47 | GC01P054715 |
| LSM2 | LSM2 Homolog, U6 Small Nuclear RNA And MRNA Degradation Associated | Protein Coding | Q9Y333 | 50 | GC06M100313 |
| LIG3 | DNA Ligase 3 | Protein Coding | P49916 | 57 | GC17P034980 |
| PTGER3 | Prostaglandin E Receptor 3 | Protein Coding | P43115 | 58 | GC01M070852 |
| IKZF4 | IKAROS Family Zinc Finger 4 | Protein Coding | Q9H2S9 | 45 | GC12P056007 |
| MIR664A | MicroRNA 664a | RNA Gene |  | 19 | GC01M220200 |
| MFN2 | Mitofusin 2 | Protein Coding | O95140 | 61 | GC01P011980 |
| SATB1 | SATB Homeobox 1 | Protein Coding | Q01826 | 56 | GC03M027493 |
| NBEAL2 | Neurobeachin Like 2 | Protein Coding | Q6ZNJ1 | 52 | GC03P046979 |
| GPX4 | Glutathione Peroxidase 4 | Protein Coding | P36969 | 59 | GC19P001103 |
| DOLK | Dolichol Kinase | Protein Coding | Q9UPQ8 | 51 | GC09M128945 |
| FKBP1A | FKBP Prolyl Isomerase 1A | Protein Coding | P62942 | 61 | GC20M001369 |
| F5 | Coagulation Factor V | Protein Coding | P12259 | 59 | GC01M169511 |
| GREM1 | Gremlin 1, DAN Family BMP Antagonist | Protein Coding | O60565 | 56 | GC15P174912 |
| RNF182 | Ring Finger Protein 182 | Protein Coding | Q8N6D2 | 42 | GC06P013924 |
| HNRNPA1 | Heterogeneous Nuclear Ribonucleoprotein A1 | Protein Coding | P09651 | 60 | GC12P054280 |
| TUBB2B | Tubulin Beta 2B Class IIb | Protein Coding | Q9BVA1 | 56 | GC06M003950 |
| DANCR | Differentiation Antagonizing Non-Protein Coding RNA | RNA Gene | P0C864 | 31 | GC04P052712 |
| FBXO9 | F-Box Protein 9 | Protein Coding | Q9UK97 | 47 | GC06P156610 |
| DDIT3 | DNA Damage Inducible Transcript 3 | Protein Coding | P35638 | 59 | GC12M057516 |
| METTL3 | Methyltransferase 3, N6-Adenosine-Methyltransferase Complex Catalytic Subunit | Protein Coding | Q86U44 | 53 | GC14M021498 |
| LOC130008987 | ATAC-STARR-Seq Lymphoblastoid Silent Region 4981 | Functional Element |  | 8 | GC12P137032 |
| KCNA3 | Potassium Voltage-Gated Channel Subfamily A Member 3 | Protein Coding | P22001 | 55 | GC01M110654 |
| IFI30 | IFI30 Lysosomal Thiol Reductase | Protein Coding | P13284 | 48 | GC19P018173 |
| CCT5 | Chaperonin Containing TCP1 Subunit 5 | Protein Coding | P48643 | 56 | GC05P010249 |
| EIF4EBP1 | Eukaryotic Translation Initiation Factor 4E Binding Protein 1 | Protein Coding | Q13541 | 59 | GC08P040885 |
| XCR1 | X-C Motif Chemokine Receptor 1 | Protein Coding | P46094 | 48 | GC03M046016 |
| DEFB118 | Defensin Beta 118 | Protein Coding | Q96PH6 | 36 | GC20P031370 |
| ALDH2 | Aldehyde Dehydrogenase 2 Family Member | Protein Coding | P05091 | 63 | GC12P111766 |
| LHCGR | Luteinizing Hormone/Choriogonadotropin Receptor | Protein Coding | P22888 | 59 | GC02M048686 |
| HNMT | Histamine N-Methyltransferase | Protein Coding | P50135 | 57 | GC02P137964 |
| NTS | Neurotensin | Protein Coding | P30990 | 53 | GC12P085876 |
| ABCC8 | ATP Binding Cassette Subfamily C Member 8 | Protein Coding | Q09428 | 58 | GC11M017392 |
| PDCD6IP | Programmed Cell Death 6 Interacting Protein | Protein Coding | Q8WUM4 | 55 | GC03P033798 |
| ABCG1 | ATP Binding Cassette Subfamily G Member 1 | Protein Coding | P45844 | 56 | GC21P042199 |
| IGLV5-52 | Immunoglobulin Lambda Variable 5-52 | Protein Coding | A0A0A0MRZ9 | 16 | GC22P022318 |
| CDH11 | Cadherin 11 | Protein Coding | P55287 | 59 | GC16M064943 |
| PRG4 | Proteoglycan 4 | Protein Coding | Q92954 | 54 | GC01P186296 |
| RECK | Reversion Inducing Cysteine Rich Protein With Kazal Motifs | Protein Coding | O95980 | 51 | GC09P036036 |
| CALCR | Calcitonin Receptor | Protein Coding | P30988 | 61 | GC07M093424 |
| ID3 | Inhibitor Of DNA Binding 3 | Protein Coding | Q02535 | 51 | GC01M023557 |
| EPHB2 | EPH Receptor B2 | Protein Coding | P29323 | 65 | GC01P022710 |
| KIF23 | Kinesin Family Member 23 | Protein Coding | Q02241 | 55 | GC15P069414 |
| HPSE | Heparanase | Protein Coding | Q9Y251 | 57 | GC04M083292 |
| LAMA5 | Laminin Subunit Alpha 5 | Protein Coding | O15230 | 56 | GC20M062307 |
| ID2 | Inhibitor Of DNA Binding 2 | Protein Coding | Q02363 | 55 | GC02P008678 |
| CDR2 | Cerebellar Degeneration Related Protein 2 | Protein Coding | Q01850 | 51 | GC16M027810 |
| KCNA1 | Potassium Voltage-Gated Channel Subfamily A Member 1 | Protein Coding | Q09470 | 58 | GC12P046714 |
| NECTIN2 | Nectin Cell Adhesion Molecule 2 | Protein Coding | Q92692 | 57 | GC19P133236 |
| TRIM28 | Tripartite Motif Containing 28 | Protein Coding | Q13263 | 57 | GC19P058544 |
| MUC6 | Mucin 6, Oligomeric Mucus/Gel-Forming | Protein Coding | Q6W4X9 | 51 | GC11M001012 |
| MID2 | Midline 2 | Protein Coding | Q9UJV3 | 50 | GC0XP107825 |
| FGF20 | Fibroblast Growth Factor 20 | Protein Coding | Q9NP95 | 51 | GC08M016992 |
| GJB1 | Gap Junction Protein Beta 1 | Protein Coding | P08034 | 60 | GC0XP071212 |
| HPGDS | Hematopoietic Prostaglandin D Synthase | Protein Coding | O60760 | 54 | GC04M094298 |
| MPI | Mannose Phosphate Isomerase | Protein Coding | P34949 | 56 | GC15P074890 |
| FA2H | Fatty Acid 2-Hydroxylase | Protein Coding | Q7L5A8 | 57 | GC16M074712 |
| SLC25A46 | Solute Carrier Family 25 Member 46 | Protein Coding | Q96AG3 | 50 | GC05P110738 |
| SYT2 | Synaptotagmin 2 | Protein Coding | Q8N9I0 | 56 | GC01M202559 |
| LGI1 | Leucine Rich Glioma Inactivated 1 | Protein Coding | O95970 | 57 | GC10P093757 |
| CSNK2A2 | Casein Kinase 2 Alpha 2 | Protein Coding | P19784 | 58 | GC16M058157 |
| POLR1C | RNA Polymerase I And III Subunit C | Protein Coding | O15160 | 56 | GC06P156505 |
| KL | Klotho | Protein Coding | Q9UEF7 | 61 | GC13P033016 |
| PLEK2 | Pleckstrin 2 | Protein Coding | Q9NYT0 | 49 | GC14M067386 |
| IGHJ1 | Immunoglobulin Heavy Joining 1 | Protein Coding | A0A0C4DH62 | 18 | GC14M122279 |
| PDSS2 | Decaprenyl Diphosphate Synthase Subunit 2 | Protein Coding | Q86YH6 | 53 | GC06M107152 |
| TAB3 | TGF-Beta Activated Kinase 1 (MAP3K7) Binding Protein 3 | Protein Coding | Q8N5C8 | 51 | GC0XM031044 |
| POLG2 | DNA Polymerase Gamma 2, Accessory Subunit | Protein Coding | Q9UHN1 | 52 | GC17M064477 |
| TK1 | Thymidine Kinase 1 | Protein Coding | P04183 | 57 | GC17M090467 |
| MIR302C | MicroRNA 302c | RNA Gene |  | 28 | GC04M112950 |
| TCF7L2 | Transcription Factor 7 Like 2 | Protein Coding | Q9NQB0 | 58 | GC10P112950 |
| PSME3 | Proteasome Activator Subunit 3 | Protein Coding | P61289 | 52 | GC17P042824 |
| FGF9 | Fibroblast Growth Factor 9 | Protein Coding | P31371 | 56 | GC13P021671 |
| RICTOR | RPTOR Independent Companion Of MTOR Complex 2 | Protein Coding | Q6R327 | 56 | GC05M038937 |
| NECTIN1 | Nectin Cell Adhesion Molecule 1 | Protein Coding | Q15223 | 59 | GC11M134069 |
| APPL1 | Adaptor Protein, Phosphotyrosine Interacting With PH Domain And Leucine Zipper 1 | Protein Coding | Q9UKG1 | 57 | GC03P057227 |
| DPM1 | Dolichyl-Phosphate Mannosyltransferase Subunit 1, Catalytic | Protein Coding | O60762 | 55 | GC20M050934 |
| EOGT | EGF Domain Specific O-Linked N-Acetylglucosamine Transferase | Protein Coding | Q5NDL2 | 51 | GC03M068975 |
| KCNN3 | Potassium Calcium-Activated Channel Subfamily N Member 3 | Protein Coding | Q9UGI6 | 55 | GC01M154697 |
| CDH23 | Cadherin Related 23 | Protein Coding | Q9H251 | 56 | GC10P071396 |
| SLX1A-SULT1A3 | SLX1A-SULT1A3 Readthrough (NMD Candidate) | RNA Gene |  | 21 | GC16P104939 |
| PPARD | Peroxisome Proliferator Activated Receptor Delta | Protein Coding | Q03181 | 58 | GC06P156423 |
| LOX | Lysyl Oxidase | Protein Coding | P28300 | 60 | GC05M122063 |
| HRH1 | Histamine Receptor H1 | Protein Coding | P35367 | 58 | GC03P024228 |
| RAD54L | RAD54 Like | Protein Coding | Q92698 | 58 | GC01P062557 |
| CBX5 | Chromobox 5 | Protein Coding | P45973 | 52 | GC12M054230 |
| KYNU | Kynureninase | Protein Coding | Q16719 | 61 | GC02P142877 |
| SPINK1 | Serine Peptidase Inhibitor Kazal Type 1 | Protein Coding | P00995 | 54 | GC05M147825 |
| COMP | Cartilage Oligomeric Matrix Protein | Protein Coding | P49747 | 59 | GC19M100624 |
| RRAS | RAS Related | Protein Coding | P10301 | 54 | GC19M049635 |
| ADORA3 | Adenosine A3 Receptor | Protein Coding | P0DMS8 | 55 | GC01M111499 |
| IAPP | Islet Amyloid Polypeptide | Protein Coding | P10997 | 52 | GC12P021354 |
| LETM1 | Leucine Zipper And EF-Hand Containing Transmembrane Protein 1 | Protein Coding | O95202 | 56 | GC04M001811 |
| KMT2E | Lysine Methyltransferase 2E (Inactive) | Protein Coding | Q8IZD2 | 52 | GC07P108911 |
| MAP2 | Microtubule Associated Protein 2 | Protein Coding | P11137 | 57 | GC02P209424 |
| LAGE3 | L Antigen Family Member 3 | Protein Coding | Q14657 | 48 | GC0XM154477 |
| ADGRE5 | Adhesion G Protein-Coupled Receptor E5 | Protein Coding | P48960 | 56 | GC19P132505 |
| ZFYVE26 | Zinc Finger FYVE-Type Containing 26 | Protein Coding | Q68DK2 | 51 | GC14M067727 |
| PAX1 | Paired Box 1 | Protein Coding | P15863 | 54 | GC20P021705 |
| NLRP9 | NLR Family Pyrin Domain Containing 9 | Protein Coding | Q7RTR0 | 44 | GC19M101600 |
| PSMB5 | Proteasome 20S Subunit Beta 5 | Protein Coding | P28074 | 55 | GC14M023016 |
| TRD-GTC9-1 | TRNA-Asp (Anticodon GTC) 9-1 | RNA Gene |  | 14 | GC01P170816 |
| CLTC | Clathrin Heavy Chain | Protein Coding | Q00610 | 59 | GC17P059619 |
| TRPA1 | Transient Receptor Potential Cation Channel Subfamily A Member 1 | Protein Coding | O75762 | 60 | GC08M072019 |
| MIR193B | MicroRNA 193b | RNA Gene |  | 29 | GC16P104308 |
| CDKN2B | Cyclin Dependent Kinase Inhibitor 2B | Protein Coding | P42772 | 58 | GC09M022002 |
| BCS1L | BCS1 Homolog, Ubiquinol-Cytochrome C Reductase Complex Chaperone | Protein Coding | Q9Y276 | 56 | GC02P218658 |
| YARS1 | Tyrosyl-TRNA Synthetase 1 | Protein Coding | P54577 | 55 | GC01M032776 |
| PAH | Phenylalanine Hydroxylase | Protein Coding | P00439 | 60 | GC12M102836 |
| NEK9 | NIMA Related Kinase 9 | Protein Coding | Q8TD19 | 58 | GC14M075079 |
| LYVE1 | Lymphatic Vessel Endothelial Hyaluronan Receptor 1 | Protein Coding | Q9Y5Y7 | 54 | GC11M013661 |
| LRSAM1 | Leucine Rich Repeat And Sterile Alpha Motif Containing 1 | Protein Coding | Q6UWE0 | 51 | GC09P127451 |
| NAGA | Alpha-N-Acetylgalactosaminidase | Protein Coding | P17050 | 58 | GC22M042058 |
| PXDNL | Peroxidasin Like | Protein Coding | A1KZ92 | 45 | GC08M051319 |
| KCNA2 | Potassium Voltage-Gated Channel Subfamily A Member 2 | Protein Coding | P16389 | 58 | GC01M110519 |
| AP1S1 | Adaptor Related Protein Complex 1 Subunit Sigma 1 | Protein Coding | P61966 | 51 | GC07P101154 |
| H6PD | Hexose-6-Phosphate Dehydrogenase/Glucose 1-Dehydrogenase | Protein Coding | O95479 | 55 | GC01P009234 |
| IL11RA | Interleukin 11 Receptor Subunit Alpha | Protein Coding | Q14626 | 56 | GC09P060025 |
| MIR187 | MicroRNA 187 | RNA Gene |  | 27 | GC18M035904 |
| VPS13D | Vacuolar Protein Sorting 13 Homolog D | Protein Coding | Q5THJ4 | 47 | GC01P060404 |
| POLR3G | RNA Polymerase III Subunit G | Protein Coding | O15318 | 47 | GC05P090471 |
| UBE2D1 | Ubiquitin Conjugating Enzyme E2 D1 | Protein Coding | P51668 | 56 | GC10P058334 |
| PIGH | Phosphatidylinositol Glycan Anchor Biosynthesis Class H | Protein Coding | Q14442 | 50 | GC14M067581 |
| TRIP13 | Thyroid Hormone Receptor Interactor 13 | Protein Coding | Q15645 | 53 | GC05P000892 |
| ATL3 | Atlastin GTPase 3 | Protein Coding | Q6DD88 | 50 | GC11M063624 |
| KLF2 | KLF Transcription Factor 2 | Protein Coding | Q9Y5W3 | 53 | GC19P132577 |
| VAMP2 | Vesicle Associated Membrane Protein 2 | Protein Coding | P63027 | 58 | GC17M088327 |
| PLEC | Plectin | Protein Coding | Q15149 | 56 | GC08M146981 |
| ALG9 | ALG9 Alpha-1,2-Mannosyltransferase | Protein Coding | Q9H6U8 | 52 | GC11M133663 |
| AP2B1 | Adaptor Related Protein Complex 2 Subunit Beta 1 | Protein Coding | P63010 | 54 | GC17P035578 |
| NTAN1 | N-Terminal Asparagine Amidase | Protein Coding | Q96AB6 | 45 | GC16M015037 |
| SERPINA6 | Serpin Family A Member 6 | Protein Coding | P08185 | 58 | GC14M122025 |
| RAP1B | RAP1B, Member Of RAS Oncogene Family | Protein Coding | P61224 | 57 | GC12P068610 |
| NDN | Necdin, MAGE Family Member | Protein Coding | Q99608 | 55 | GC15M028063 |
| MARS1 | Methionyl-TRNA Synthetase 1 | Protein Coding | P56192 | 58 | GC12P065436 |
| CHRNG | Cholinergic Receptor Nicotinic Gamma Subunit | Protein Coding | P07510 | 54 | GC02P232539 |
| NCOR2 | Nuclear Receptor Corepressor 2 | Protein Coding | Q9Y618 | 55 | GC12M124324 |
| IGKV2D-29 | Immunoglobulin Kappa Variable 2D-29 | Protein Coding | A0A075B6S2 | 18 | GC02P093747 |
| FYB1 | FYN Binding Protein 1 | Protein Coding | O15117 | 55 | GC05M039105 |
| PACS1 | Phosphofurin Acidic Cluster Sorting Protein 1 | Protein Coding | Q6VY07 | 53 | GC11P099021 |
| XPR1 | Xenotropic And Polytropic Retrovirus Receptor 1 | Protein Coding | Q9UBH6 | 54 | GC01P180632 |
| ABCC1 | ATP Binding Cassette Subfamily C Member 1 (ABCC1 Blood Group) | Protein Coding | P33527 | 61 | GC16P015949 |
| ARCN1 | Archain 1 | Protein Coding | P48444 | 54 | GC11P118572 |
| FKRP | Fukutin Related Protein | Protein Coding | Q9H9S5 | 51 | GC19P133283 |
| PCNT | Pericentrin | Protein Coding | O95613 | 55 | GC21P046324 |
| ABHD12 | Abhydrolase Domain Containing 12, Lysophospholipase | Protein Coding | Q8N2K0 | 53 | GC20M025294 |
| FOXG1 | Forkhead Box G1 | Protein Coding | P55316 | 57 | GC14P051795 |
| TRP-AGG2-5 | TRNA-Pro (Anticodon AGG) 2-5 | RNA Gene |  | 17 | GC14M027774 |
| PDCD10 | Programmed Cell Death 10 | Protein Coding | Q9BUL8 | 53 | GC03M167683 |
| AP2M1 | Adaptor Related Protein Complex 2 Subunit Mu 1 | Protein Coding | Q96CW1 | 59 | GC03P184174 |
| BAG3 | BAG Cochaperone 3 | Protein Coding | O95817 | 55 | GC10P119651 |
| MLH3 | MutL Homolog 3 | Protein Coding | Q9UHC1 | 52 | GC14M075013 |
| RERE | Arginine-Glutamic Acid Dipeptide Repeats | Protein Coding | Q9P2R6 | 54 | GC01M019741 |
| IGHA1 | Immunoglobulin Heavy Constant Alpha 1 | Protein Coding | P01876 | 36 | GC14M122255 |
| ASS1 | Argininosuccinate Synthase 1 | Protein Coding | P00966 | 62 | GC09P130444 |
| GSDMB | Gasdermin B | Protein Coding | Q8TAX9 | 44 | GC17M039904 |
| INPPL1 | Inositol Polyphosphate Phosphatase Like 1 | Protein Coding | O15357 | 60 | GC11P072223 |
| UBAC2 | UBA Domain Containing 2 | Protein Coding | Q8NBM4 | 47 | GC13P099200 |
| MIR30C1 | MicroRNA 30c-1 | RNA Gene |  | 30 | GC01P040757 |
| H3-5 | H3.5 Histone | Protein Coding | Q6NXT2 | 44 | GC12M033463 |
| FLNB | Filamin B | Protein Coding | O75369 | 60 | GC03P058008 |
| MGA | MAX Dimerization Protein MGA | Protein Coding | Q8IWI9 | 47 | GC15P041621 |
| MIR101-1 | MicroRNA 101-1 | RNA Gene |  | 28 | GC01M065058 |
| TSG101 | Tumor Susceptibility 101 | Protein Coding | Q99816 | 56 | GC11M018468 |
| PPP6C | Protein Phosphatase 6 Catalytic Subunit | Protein Coding | O00743 | 53 | GC09M125212 |
| SLC1A3 | Solute Carrier Family 1 Member 3 | Protein Coding | P43003 | 62 | GC05P036712 |
| BCKDHA | Branched Chain Keto Acid Dehydrogenase E1 Subunit Alpha | Protein Coding | P12694 | 55 | GC19P133107 |
| PDPK1 | 3-Phosphoinositide Dependent Protein Kinase 1 | Protein Coding | O15530 | 61 | GC16P002537 |
| SSBP1 | Single Stranded DNA Binding Protein 1 | Protein Coding | Q04837 | 53 | GC07P163092 |
| STX1A | Syntaxin 1A | Protein Coding | Q16623 | 60 | GC07M079074 |
| EFTUD2 | Elongation Factor Tu GTP Binding Domain Containing 2 | Protein Coding | Q15029 | 54 | GC17M089486 |
| CSNK2B | Casein Kinase 2 Beta | Protein Coding | P67870 | 61 | GC06P156247 |
| H3-7 | H3.7 Histone (Putative) | Protein Coding | Q5TEC6 | 31 | GC01M164452 |
| HSPB8 | Heat Shock Protein Family B (Small) Member 8 | Protein Coding | Q9UJY1 | 57 | GC12P119173 |
| AHCY | Adenosylhomocysteinase | Protein Coding | P23526 | 61 | GC20M035962 |
| PFN1 | Profilin 1 | Protein Coding | P07737 | 59 | GC17M004945 |
| KAT2A | Lysine Acetyltransferase 2A | Protein Coding | Q92830 | 62 | GC17M042113 |
| LOC129930068 | ATAC-STARR-Seq Lymphoblastoid Active Region 705 | Functional Element |  | 8 | GC01P067145 |
| FAAH | Fatty Acid Amide Hydrolase | Protein Coding | O00519 | 61 | GC01P046394 |
| CENPE | Centromere Protein E | Protein Coding | Q02224 | 58 | GC04M103105 |
| GPR15 | G Protein-Coupled Receptor 15 | Protein Coding | P49685 | 45 | GC03P098531 |
| SNHG5 | Small Nucleolar RNA Host Gene 5 | RNA Gene |  | 29 | GC06M085650 |
| ABCA3 | ATP Binding Cassette Subfamily A Member 3 | Protein Coding | Q99758 | 61 | GC16M002275 |
| BAK1 | BCL2 Antagonist/Killer 1 | Protein Coding | Q16611 | 57 | GC06M033572 |
| HNRNPC | Heterogeneous Nuclear Ribonucleoprotein C | Protein Coding | P07910 | 55 | GC14M027828 |
| MIR133A1 | MicroRNA 133a-1 | RNA Gene |  | 26 | GC18M036072 |
| NPPA | Natriuretic Peptide A | Protein Coding | P01160 | 57 | GC01M019842 |
| TUBB2A | Tubulin Beta 2A Class IIa | Protein Coding | Q13885 | 59 | GC06M003153 |
| TUBB4A | Tubulin Beta 4A Class IVa | Protein Coding | P04350 | 59 | GC19M100096 |
| HOTAIRM1 | HOXA Transcript Antisense RNA, Myeloid-Specific 1 | RNA Gene |  | 28 | GC07P027095 |
| IGHV1-46 | Immunoglobulin Heavy Variable 1-46 | Protein Coding | P01743 | 21 | GC14M122327 |
| MIR181C | MicroRNA 181c | RNA Gene |  | 29 | GC19P132486 |
| NDRG1 | N-Myc Downstream Regulated 1 | Protein Coding | Q92597 | 57 | GC08M133237 |
| DCK | Deoxycytidine Kinase | Protein Coding | P27707 | 56 | GC04P070992 |
| NR1I2 | Nuclear Receptor Subfamily 1 Group I Member 2 | Protein Coding | O75469 | 55 | GC03P119780 |
| IGHV3-30 | Immunoglobulin Heavy Variable 3-30 | Protein Coding | P01768 | 19 | GC14M122509 |
| IGLC3 | Immunoglobulin Lambda Constant 3 (Kern-Oz+ Marker) | Protein Coding | P0DOY3 | 24 | GC22P081017 |
| LILRA3 | Leukocyte Immunoglobulin Like Receptor A3 | Protein Coding | Q8N6C8 | 34 | GC19Mp00270 |
| GNB2 | G Protein Subunit Beta 2 | Protein Coding | P62879 | 56 | GC07P100673 |
| GSTM1 | Glutathione S-Transferase Mu 1 | Protein Coding | P09488 | 55 | GC01P109687 |
| PVRIG | PVR Related Immunoglobulin Domain Containing | Protein Coding | Q6DKI7 | 38 | GC07P100218 |
| CTNNA1 | Catenin Alpha 1 | Protein Coding | P35221 | 60 | GC05P138743 |
| OGG1 | 8-Oxoguanine DNA Glycosylase | Protein Coding | O15527 | 59 | GC03P024175 |
| KCNMA1 | Potassium Calcium-Activated Channel Subfamily M Alpha 1 | Protein Coding | Q12791 | 60 | GC10M076869 |
| ACTG1 | Actin Gamma 1 | Protein Coding | P63261 | 60 | GC17M081509 |
| ACAN | Aggrecan | Protein Coding | P16112 | 59 | GC15P176217 |
| NUP205 | Nucleoporin 205 | Protein Coding | Q92621 | 52 | GC07P135557 |
| HCCAT5 | Hepatocellular Carcinoma Associated Transcript 5 | RNA Gene |  | 26 | GC16P073092 |
| ANKH | ANKH Inorganic Pyrophosphate Transport Regulator | Protein Coding | Q9HCJ1 | 51 | GC05M014838 |
| S1PR2 | Sphingosine-1-Phosphate Receptor 2 | Protein Coding | O95136 | 58 | GC19M100258 |
| WARS1 | Tryptophanyl-TRNA Synthetase 1 | Protein Coding | P23381 | 59 | GC14M122090 |
| ALOX12 | Arachidonate 12-Lipoxygenase, 12S Type | Protein Coding | P18054 | 56 | GC17P132604 |
| CYP2C19 | Cytochrome P450 Family 2 Subfamily C Member 19 | Protein Coding | P33261 | 57 | GC10P094762 |
| LPP | LIM Domain Containing Preferred Translocation Partner In Lipoma | Protein Coding | Q93052 | 56 | GC03P188153 |
| CTTN | Cortactin | Protein Coding | Q14247 | 56 | GC11P070398 |
| MB | Myoglobin | Protein Coding | P02144 | 58 | GC22M035606 |
| MT-TL1 | Mitochondrially Encoded TRNA-Leu (UUA/G) 1 | RNA Gene |  | 26 | GCMTP003232 |
| HLCS | Holocarboxylase Synthetase | Protein Coding | P50747 | 55 | GC21M036750 |
| GUCY2C | Guanylate Cyclase 2C | Protein Coding | P25092 | 57 | GC12M014612 |
| TFPI | Tissue Factor Pathway Inhibitor | Protein Coding | P10646 | 58 | GC02M187464 |
| SLC5A2 | Solute Carrier Family 5 Member 2 | Protein Coding | P31639 | 59 | GC16P105032 |
| PLCB1 | Phospholipase C Beta 1 | Protein Coding | Q9NQ66 | 61 | GC20P008077 |
| ULBP2 | UL16 Binding Protein 2 | Protein Coding | Q9BZM5 | 50 | GC06P163684 |
| MTRFR | Mitochondrial Translation Release Factor In Rescue | Protein Coding | Q9H3J6 | 45 | GC12P136130 |
| MIR582 | MicroRNA 582 | RNA Gene |  | 29 | GC05M059703 |
| IQCB1 | IQ Motif Containing B1 | Protein Coding | Q15051 | 54 | GC03M121769 |
| JUNB | JunB Proto-Oncogene, AP-1 Transcription Factor Subunit | Protein Coding | P17275 | 52 | GC19P012791 |
| IGLC2 | Immunoglobulin Lambda Constant 2 | Protein Coding | P0DOY2 | 29 | GC22P022900 |
| AP4E1 | Adaptor Related Protein Complex 4 Subunit Epsilon 1 | Protein Coding | Q9UPM8 | 48 | GC15P175366 |
| POLR3K | RNA Polymerase III Subunit K | Protein Coding | Q9Y2Y1 | 52 | GC16M000046 |
| BCAP31 | B Cell Receptor Associated Protein 31 | Protein Coding | P51572 | 53 | GC0XM153701 |
| CYSLTR1 | Cysteinyl Leukotriene Receptor 1 | Protein Coding | Q9Y271 | 54 | GC0XM078271 |
| SIRT6 | Sirtuin 6 | Protein Coding | Q8N6T7 | 56 | GC19M004174 |
| APOBEC3A | Apolipoprotein B MRNA Editing Enzyme Catalytic Subunit 3A | Protein Coding | P31941 | 49 | GC22P038952 |
| DSCAM-AS1 | DSCAM Antisense RNA 1 | RNA Gene |  | 23 | GC21P040383 |
| NUP93 | Nucleoporin 93 | Protein Coding | Q8N1F7 | 52 | GC16P105580 |
| NCKAP1 | NCK Associated Protein 1 | Protein Coding | Q9Y2A7 | 52 | GC02M182909 |
| PEX6 | Peroxisomal Biogenesis Factor 6 | Protein Coding | Q13608 | 53 | GC06M042963 |
| CAND1 | Cullin Associated And Neddylation Dissociated 1 | Protein Coding | Q86VP6 | 51 | GC12P068147 |
| EXOSC10 | Exosome Component 10 | Protein Coding | Q01780 | 49 | GC01M019810 |
| ATP2A2 | ATPase Sarcoplasmic/Endoplasmic Reticulum Ca2+ Transporting 2 | Protein Coding | P16615 | 63 | GC12P110280 |
| COG1 | Component Of Oligomeric Golgi Complex 1 | Protein Coding | Q8WTW3 | 51 | GC17P073193 |
| LOC130055497 | ATAC-STARR-Seq Lymphoblastoid Silent Region 5676 | Functional Element |  | 8 | GC14P053626 |
| OXT | Oxytocin/Neurophysin I Prepropeptide | Protein Coding | P01178 | 50 | GC20P009536 |
| FBL | Fibrillarin | Protein Coding | P22087 | 55 | GC19M039834 |
| SP140 | SP140 Nuclear Body Protein | Protein Coding | Q13342 | 47 | GC02P230203 |
| CYP3A4 | Cytochrome P450 Family 3 Subfamily A Member 4 | Protein Coding | P08684 | 61 | GC07M106050 |
| STIM2 | Stromal Interaction Molecule 2 | Protein Coding | Q9P246 | 51 | GC04P027156 |
| MUC16 | Mucin 16, Cell Surface Associated | Protein Coding | Q8WXI7 | 51 | GC19M008848 |
| MIR425 | MicroRNA 425 | RNA Gene |  | 29 | GC03M054248 |
| HYCC1 | Hyccin PI4KA Lipid Kinase Complex Subunit 1 | Protein Coding | Q9BYI3 | 48 | GC07M022936 |
| SRD5A3 | Steroid 5 Alpha-Reductase 3 | Protein Coding | Q9H8P0 | 53 | GC04P055346 |
| TRIP11 | Thyroid Hormone Receptor Interactor 11 | Protein Coding | Q15643 | 52 | GC14M091965 |
| LILRA1 | Leukocyte Immunoglobulin Like Receptor A1 | Protein Coding | O75019 | 43 | GC19P054593 |
| SHPK | Sedoheptulokinase | Protein Coding | Q9UHJ6 | 49 | GC17M003608 |
| KRT7 | Keratin 7 | Protein Coding | P08729 | 55 | GC12P052232 |
| TNNI3 | Troponin I3, Cardiac Type | Protein Coding | P19429 | 61 | GC19M055151 |
| SEC23B | SEC23 Homolog B, COPII Coat Complex Component | Protein Coding | Q15437 | 53 | GC20P018507 |
| VAMP7 | Vesicle Associated Membrane Protein 7 | Protein Coding | P51809 | 54 | GC0XP155881 |
| APEH | Acylaminoacyl-Peptide Hydrolase | Protein Coding | P13798 | 51 | GC03P049673 |
| TUBA8 | Tubulin Alpha 8 | Protein Coding | Q9NY65 | 56 | GC22P018110 |
| BAD | BCL2 Associated Agonist Of Cell Death | Protein Coding | Q92934 | 57 | GC11M132747 |
| DLG4 | Discs Large MAGUK Scaffold Protein 4 | Protein Coding | P78352 | 61 | GC17M088271 |
| TDP2 | Tyrosyl-DNA Phosphodiesterase 2 | Protein Coding | O95551 | 56 | GC06M024651 |
| HRH2 | Histamine Receptor H2 | Protein Coding | P25021 | 55 | GC05P175659 |
| NEDD9 | Neural Precursor Cell Expressed, Developmentally Down-Regulated 9 | Protein Coding | Q14511 | 53 | GC06M011183 |
| AASS | Aminoadipate-Semialdehyde Synthase | Protein Coding | Q9UDR5 | 54 | GC07M122073 |
| DOCK8-AS1 | DOCK8 Antisense RNA 1 | RNA Gene | Q5T8R8 | 33 | GC09M000603 |
| IGKV2-29 | Immunoglobulin Kappa Variable 2-29 | Pseudogene | A2NJV5 | 16 | GC02M092677 |
| GPX8 | Glutathione Peroxidase 8 (Putative) | Protein Coding | Q8TED1 | 50 | GC05P055160 |
| BTN3A1 | Butyrophilin Subfamily 3 Member A1 | Protein Coding | O00481 | 51 | GC06P026402 |
| RFT1 | RFT1 Homolog | Protein Coding | Q96AA3 | 50 | GC03M054418 |
| SOX4 | SRY-Box Transcription Factor 4 | Protein Coding | Q06945 | 55 | GC06P021593 |
| TRAPPC10 | Trafficking Protein Particle Complex Subunit 10 | Protein Coding | P48553 | 50 | GC21P044012 |
| CD109 | CD109 Molecule | Protein Coding | Q6YHK3 | 48 | GC06P156873 |
| SYNPO | Synaptopodin | Protein Coding | Q8N3V7 | 52 | GC05P155679 |
| GCG | Glucagon | Protein Coding | P01275 | 54 | GC02M162142 |
| CDK7 | Cyclin Dependent Kinase 7 | Protein Coding | P50613 | 58 | GC05P079458 |
| PLA2G1B | Phospholipase A2 Group IB | Protein Coding | P04054 | 56 | GC12M120322 |
| NAT2 | N-Acetyltransferase 2 | Protein Coding | P11245 | 55 | GC08P018391 |
| ALDH7A1 | Aldehyde Dehydrogenase 7 Family Member A1 | Protein Coding | P49419 | 60 | GC05M126541 |
| EBF1 | EBF Transcription Factor 1 | Protein Coding | Q9UH73 | 54 | GC05M158695 |
| TRE-TTC3-1 | TRNA-Glu (Anticodon TTC) 3-1 | RNA Gene |  | 18 | GC01P060692 |
| IGHV3-49 | Immunoglobulin Heavy Variable 3-49 | Protein Coding | A0A0A0MS15 | 18 | GC14M122332 |
| EXOSC9 | Exosome Component 9 | Protein Coding | Q06265 | 54 | GC04P121801 |
| NKRF | NFKB Repressing Factor | Protein Coding | O15226 | 48 | GC0XM119588 |
| MSTN | Myostatin | Protein Coding | O14793 | 59 | GC02M190055 |
| DLK1 | Delta Like Non-Canonical Notch Ligand 1 | Protein Coding | P80370 | 58 | GC14P118190 |
| RAD52 | RAD52 Homolog, DNA Repair Protein | Protein Coding | P43351 | 54 | GC12M000912 |
| BAG6 | BAG Cochaperone 6 | Protein Coding | P46379 | 51 | GC06M031639 |
| PRSS1 | Serine Protease 1 | Protein Coding | P07477 | 56 | GC07P161556 |
| MIR29B2 | MicroRNA 29b-2 | RNA Gene |  | 27 | GC01M207839 |
| PPP1CA | Protein Phosphatase 1 Catalytic Subunit Alpha | Protein Coding | P62136 | 61 | GC11M132909 |
| PHB1 | Prohibitin 1 | Protein Coding | P35232 | 59 | GC17M089732 |
| SLC1A2 | Solute Carrier Family 1 Member 2 | Protein Coding | P43004 | 62 | GC11M035267 |
| FLII | FLII Actin Remodeling Protein | Protein Coding | Q13045 | 55 | GC17M018244 |
| PLK1 | Polo Like Kinase 1 | Protein Coding | P53350 | 63 | GC16P104634 |
| DPYSL5 | Dihydropyrimidinase Like 5 | Protein Coding | Q9BPU6 | 52 | GC02P026847 |
| INHA | Inhibin Subunit Alpha | Protein Coding | P05111 | 56 | GC02P219569 |
| MIR379 | MicroRNA 379 | RNA Gene |  | 26 | GC14P119064 |
| RAB4B-EGLN2 | RAB4B-EGLN2 Readthrough (NMD Candidate) | RNA Gene |  | 20 | GC19P040778 |
| EIF2S3 | Eukaryotic Translation Initiation Factor 2 Subunit Gamma | Protein Coding | P41091 | 57 | GC0XP024054 |
| PPIG | Peptidylprolyl Isomerase G | Protein Coding | Q13427 | 52 | GC02P169584 |
| LILRA5 | Leukocyte Immunoglobulin Like Receptor A5 | Protein Coding | A6NI73 | 43 | GC19M054307 |
| UBE4A | Ubiquitination Factor E4A | Protein Coding | Q14139 | 51 | GC11P118359 |
| PHYH | Phytanoyl-CoA 2-Hydroxylase | Protein Coding | O14832 | 56 | GC10M013277 |
| APOBEC1 | Apolipoprotein B MRNA Editing Enzyme Catalytic Subunit 1 | Protein Coding | P41238 | 50 | GC12M007649 |
| WASHC5 | WASH Complex Subunit 5 | Protein Coding | Q12768 | 51 | GC08M137190 |
| GAB2 | GRB2 Associated Binding Protein 2 | Protein Coding | Q9UQC2 | 55 | GC11M078215 |
| RAD54B | RAD54 Homolog B | Protein Coding | Q9Y620 | 53 | GC08M094371 |
| ERVK-6 | Endogenous Retrovirus Group K Member 6, Envelope | Protein Coding | Q69384 | 23 | GC07U903184 |
| GSDMA | Gasdermin A | Protein Coding | Q96QA5 | 47 | GC17P133703 |
| ILF2 | Interleukin Enhancer Binding Factor 2 | Protein Coding | Q12905 | 49 | GC01M153661 |
| WRNIP1 | WRN Helicase Interacting Protein 1 | Protein Coding | Q96S55 | 47 | GC06P002846 |
| EMD | Emerin | Protein Coding | P50402 | 58 | GC0XP154379 |
| UBE3B | Ubiquitin Protein Ligase E3B | Protein Coding | Q7Z3V4 | 52 | GC12P109477 |
| NMI | N-Myc And STAT Interactor | Protein Coding | Q13287 | 47 | GC02M151270 |
| TOP2A | DNA Topoisomerase II Alpha | Protein Coding | P11388 | 63 | GC17M040388 |
| BMI1 | BMI1 Proto-Oncogene, Polycomb Ring Finger | Protein Coding | P35226 | 55 | GC10P022363 |
| LORICRIN | Loricrin Cornified Envelope Precursor Protein | Protein Coding | P23490 | 46 | GC01P170349 |
| ZMYND11 | Zinc Finger MYND-Type Containing 11 | Protein Coding | Q15326 | 52 | GC10P000134 |
| SIGLEC7 | Sialic Acid Binding Ig Like Lectin 7 | Protein Coding | Q9Y286 | 52 | GC19P051142 |
| LGR5 | Leucine Rich Repeat Containing G Protein-Coupled Receptor 5 | Protein Coding | O75473 | 56 | GC12P071439 |
| POLR2L | RNA Polymerase II, I And III Subunit L | Protein Coding | P62875 | 52 | GC11M013238 |
| COL7A1 | Collagen Type VII Alpha 1 Chain | Protein Coding | Q02388 | 56 | GC03M048564 |
| RHOD | Ras Homolog Family Member D | Protein Coding | O00212 | 48 | GC11P099089 |
| S100A7A | S100 Calcium Binding Protein A7A | Protein Coding | Q86SG5 | 44 | GC01P153416 |
| AKIRIN2 | Akirin 2 | Protein Coding | Q53H80 | 41 | GC06M087674 |
| CALML6 | Calmodulin Like 6 | Protein Coding | Q8TD86 | 44 | GC01P059900 |
| NES | Nestin | Protein Coding | P48681 | 52 | GC01M156668 |
| CD53 | CD53 Molecule | Protein Coding | P19397 | 48 | GC01P110871 |
| ZC3HAV1 | Zinc Finger CCCH-Type Containing, Antiviral 1 | Protein Coding | Q7Z2W4 | 49 | GC07M139127 |
| PNMA2 | PNMA Family Member 2 | Protein Coding | Q9UL42 | 48 | GC08M026504 |
| DEFA4 | Defensin Alpha 4 | Protein Coding | P12838 | 43 | GC08M006935 |
| PTGES | Prostaglandin E Synthase | Protein Coding | O14684 | 54 | GC09M129738 |
| CD151 | CD151 Molecule (Raph Blood Group) | Protein Coding | P48509 | 55 | GC11P014057 |
| PFKL | Phosphofructokinase, Liver Type | Protein Coding | P17858 | 58 | GC21P044300 |
| SKI | SKI Proto-Oncogene | Protein Coding | P12755 | 57 | GC01P059907 |
| MAP2K6 | Mitogen-Activated Protein Kinase Kinase 6 | Protein Coding | P52564 | 60 | GC17P069414 |
| NUP160 | Nucleoporin 160 | Protein Coding | Q12769 | 52 | GC11M132481 |
| GPT2 | Glutamic--Pyruvic Transaminase 2 | Protein Coding | Q8TD30 | 58 | GC16P105274 |
| AMPH | Amphiphysin | Protein Coding | P49418 | 56 | GC07M039633 |
| CLDN11 | Claudin 11 | Protein Coding | O75508 | 54 | GC03P170418 |
| TRAPPC9 | Trafficking Protein Particle Complex Subunit 9 | Protein Coding | Q96Q05 | 51 | GC08M139728 |
| SRSF1 | Serine And Arginine Rich Splicing Factor 1 | Protein Coding | Q07955 | 55 | GC17M089862 |
| DDA1 | DET1 And DDB1 Associated 1 | Protein Coding | Q9BW61 | 44 | GC19P132594 |
| CD300C | CD300c Molecule | Protein Coding | Q08708 | 48 | GC17M090306 |
| YES1 | YES Proto-Oncogene 1, Src Family Tyrosine Kinase | Protein Coding | P07947 | 59 | GC18M000721 |
| ADGRE3 | Adhesion G Protein-Coupled Receptor E3 | Protein Coding | Q9BY15 | 42 | GC19M100439 |
| TLN2 | Talin 2 | Protein Coding | Q9Y4G6 | 53 | GC15P062390 |
| CCNE1 | Cyclin E1 | Protein Coding | P24864 | 61 | GC19P029811 |
| P2RY12 | Purinergic Receptor P2Y12 | Protein Coding | Q9H244 | 60 | GC03M151336 |
| IGKV2D-40 | Immunoglobulin Kappa Variable 2D-40 | Protein Coding | P01614 | 17 | GC02P093730 |
| MST1 | Macrophage Stimulating 1 | Protein Coding | P26927 | 58 | GC03M049683 |
| ABCC5 | ATP Binding Cassette Subfamily C Member 5 | Protein Coding | O15440 | 55 | GC03M183919 |
| SLC25A15 | Solute Carrier Family 25 Member 15 | Protein Coding | Q9Y619 | 56 | GC13P040789 |
| APEX1 | Apurinic/Apyrimidinic Endodeoxyribonuclease 1 | Protein Coding | P27695 | 58 | GC14P020455 |
| MIR136 | MicroRNA 136 | RNA Gene |  | 29 | GC14P118054 |
| RAI1 | Retinoic Acid Induced 1 | Protein Coding | Q7Z5J4 | 51 | GC17P132989 |
| MECOM | MDS1 And EVI1 Complex Locus | Protein Coding | Q03112 | 60 | GC03M169083 |
| TBKBP1 | TBK1 Binding Protein 1 | Protein Coding | A7MCY6 | 47 | GC17P047694 |
| ECM1 | Extracellular Matrix Protein 1 | Protein Coding | Q16610 | 57 | GC01P150508 |
| MIR33B | MicroRNA 33b | RNA Gene |  | 27 | GC17M017813 |
| SLC3A2 | Solute Carrier Family 3 Member 2 | Protein Coding | P08195 | 55 | GC11P062856 |
| TRIM4 | Tripartite Motif Containing 4 | Protein Coding | Q9C037 | 37 | GC07M099876 |
| COQ8B | Coenzyme Q8B | Protein Coding | Q96D53 | 52 | GC19M100978 |
| A2M | Alpha-2-Macroglobulin | Protein Coding | P01023 | 59 | GC12M009067 |
| MAL | Mal, T Cell Differentiation Protein | Protein Coding | P21145 | 47 | GC02P095025 |
| UBE2D3 | Ubiquitin Conjugating Enzyme E2 D3 | Protein Coding | P61077 | 56 | GC04M102794 |
| SNX10 | Sorting Nexin 10 | Protein Coding | Q9Y5X0 | 51 | GC07P026291 |
| PCBP2 | Poly(RC) Binding Protein 2 | Protein Coding | Q15366 | 53 | GC12P053452 |
| TRBC2 | T Cell Receptor Beta Constant 2 | Protein Coding | A0A5B9 | 24 | GC07P161575 |
| IGKV1-33 | Immunoglobulin Kappa Variable 1-33 | Protein Coding | P01594 | 24 | GC02M089268 |
| DMD | Dystrophin | Protein Coding | P11532 | 58 | GC0XM031097 |
| RBM14 | RNA Binding Motif Protein 14 | Protein Coding | Q96PK6 | 51 | GC11P099068 |
| PIGL | Phosphatidylinositol Glycan Anchor Biosynthesis Class L | Protein Coding | Q9Y2B2 | 50 | GC17P016217 |
| PLXNC1 | Plexin C1 | Protein Coding | O60486 | 51 | GC12P094150 |
| UCA1 | Urothelial Cancer Associated 1 | RNA Gene |  | 30 | GC19P141717 |
| IGHD | Immunoglobulin Heavy Constant Delta | Protein Coding | P01880 | 37 | GC14M122261 |
| DNAJB1 | DnaJ Heat Shock Protein Family (Hsp40) Member B1 | Protein Coding | P25685 | 57 | GC19M014514 |
| LINC01139 | Long Intergenic Non-Protein Coding RNA 1139 | RNA Gene |  | 22 | GC01M238480 |
| SCP2 | Sterol Carrier Protein 2 | Protein Coding | P22307 | 58 | GC01P052927 |
| CYP7A1 | Cytochrome P450 Family 7 Subfamily A Member 1 | Protein Coding | P22680 | 55 | GC08M058490 |
| ZEB2 | Zinc Finger E-Box Binding Homeobox 2 | Protein Coding | O60315 | 61 | GC02M144384 |
| CPT2 | Carnitine Palmitoyltransferase 2 | Protein Coding | P23786 | 62 | GC01P053196 |
| PILRA | Paired Immunoglobin Like Type 2 Receptor Alpha | Protein Coding | Q9UKJ1 | 48 | GC07P100367 |
| IGLC6 | Immunoglobulin Lambda Constant 6 | Pseudogene | P0CF74 | 17 | GC22P022919 |
| NIPA2 | NIPA Magnesium Transporter 2 | Protein Coding | Q8N8Q9 | 48 | GC15P174542 |
| CHD4 | Chromodomain Helicase DNA Binding Protein 4 | Protein Coding | Q14839 | 58 | GC12M006570 |
| YWHAG | Tyrosine 3-Monooxygenase/Tryptophan 5-Monooxygenase Activation Protein Gamma | Protein Coding | P61981 | 61 | GC07M079217 |
| ERBB4 | Erb-B2 Receptor Tyrosine Kinase 4 | Protein Coding | Q15303 | 67 | GC02M211375 |
| TRPM2 | Transient Receptor Potential Cation Channel Subfamily M Member 2 | Protein Coding | O94759 | 52 | GC21P044350 |
| FGF21 | Fibroblast Growth Factor 21 | Protein Coding | Q9NSA1 | 51 | GC19P133377 |
| ACVRL1 | Activin A Receptor Like Type 1 | Protein Coding | P37023 | 63 | GC12P051906 |
| KHDRBS1 | KH RNA Binding Domain Containing, Signal Transduction Associated 1 | Protein Coding | Q07666 | 54 | GC01P032013 |
| PSME2 | Proteasome Activator Subunit 2 | Protein Coding | Q9UL46 | 49 | GC14M024143 |
| CALCOCO2 | Calcium Binding And Coiled-Coil Domain 2 | Protein Coding | Q13137 | 50 | GC17P134050 |
| IVL | Involucrin | Protein Coding | P07476 | 51 | GC01P170337 |
| MIR376A1 | MicroRNA 376a-1 | RNA Gene |  | 24 | GC14P119059 |
| IGHV3-11 | Immunoglobulin Heavy Variable 3-11 | Protein Coding | P01762 | 23 | GC14M106116 |
[truncated: 583,143 more chars]
