# Supplementary material for: Integrative bioinformatics and experimental validation unveil CRISP3 as a hypoxia-, epithelial mesenchymal transition-, and immune-related prognostic biomarker and therapeutic target in breast cancer
Source: Front Immunol. 2025 Oct 22;16:1634399. doi: 10.3389/fimmu.2025.1634399 (PMC12585952; doi:10.3389/fimmu.2025.1634399)
Supplement: Supplementary file 2 [file DataSheet1.pdf]

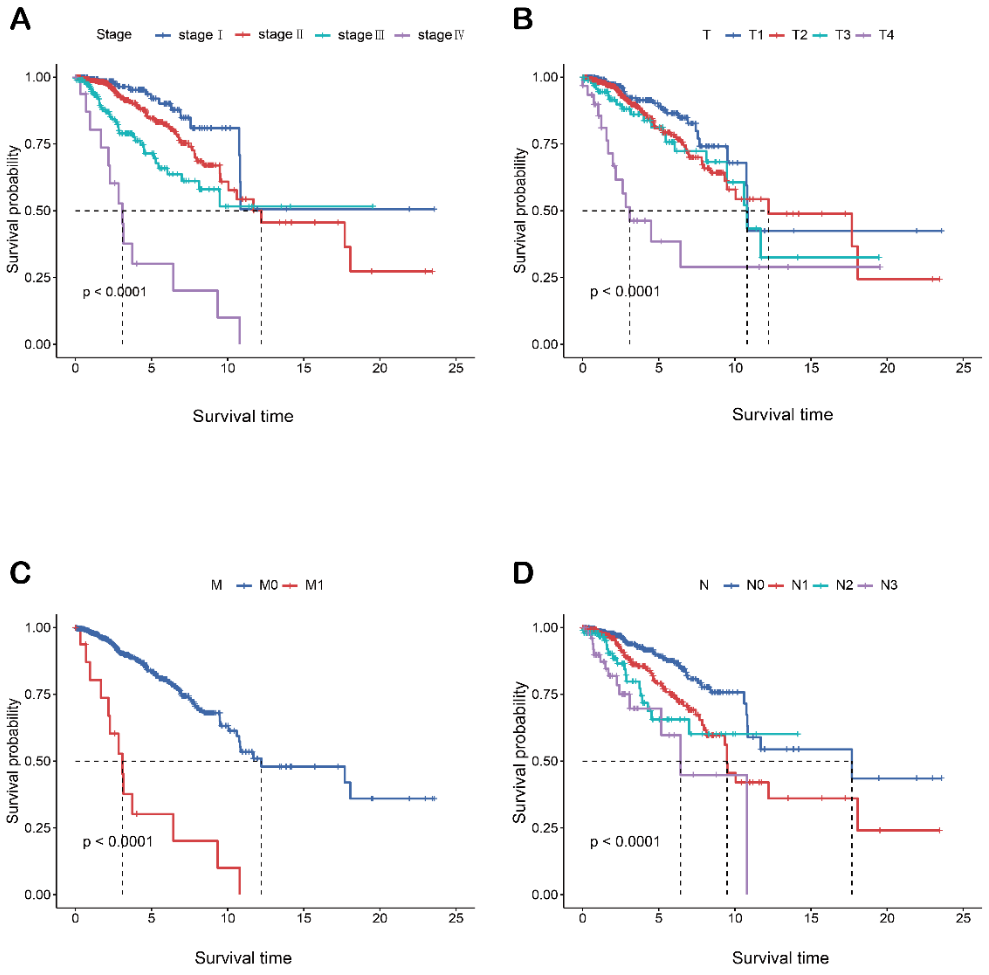


**Supplementary Figure S1. The Kaplan-Meier survival curves of TCGA BRCA cohort. (A)** stage I-IV, **(B)** stage T, **(C)** stage M, and **(D)** stage N.

**A**

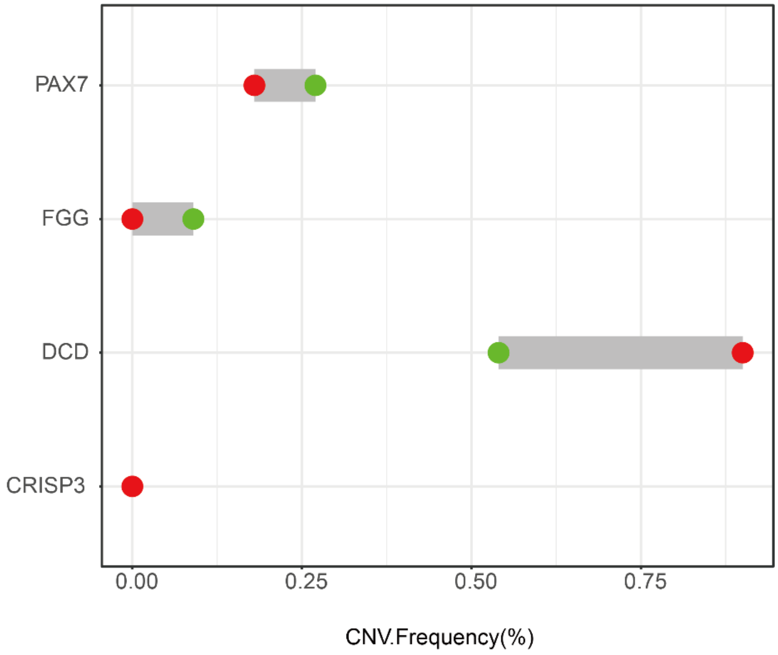


**Supplementary Figure S2. Genetic characteristics of HEMTIRGs in TCGA BRCA cohorts. (A)** Copy number variation frequency of HEMTIRGs. Copy numbers exceeding 2 are categorized as “GAIN”, while below 2 are classified as “LOSS”.

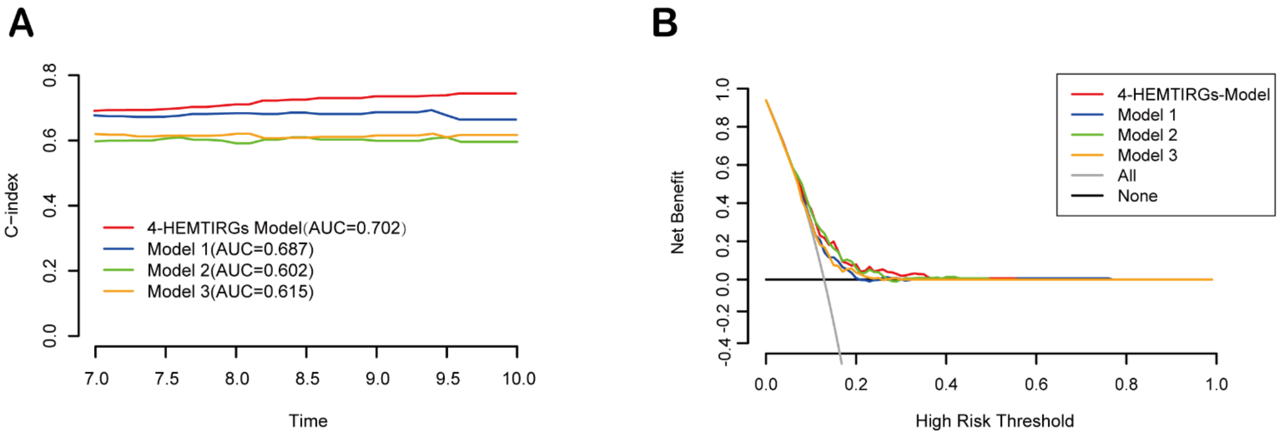


**Supplementary Figure S3. The comparison analysis of the HEMTIRGs model with the other three models. (A)** The C-index curves and **(B)** DCA profiles analyses of the HEMTIRGs model and Models 1-3.

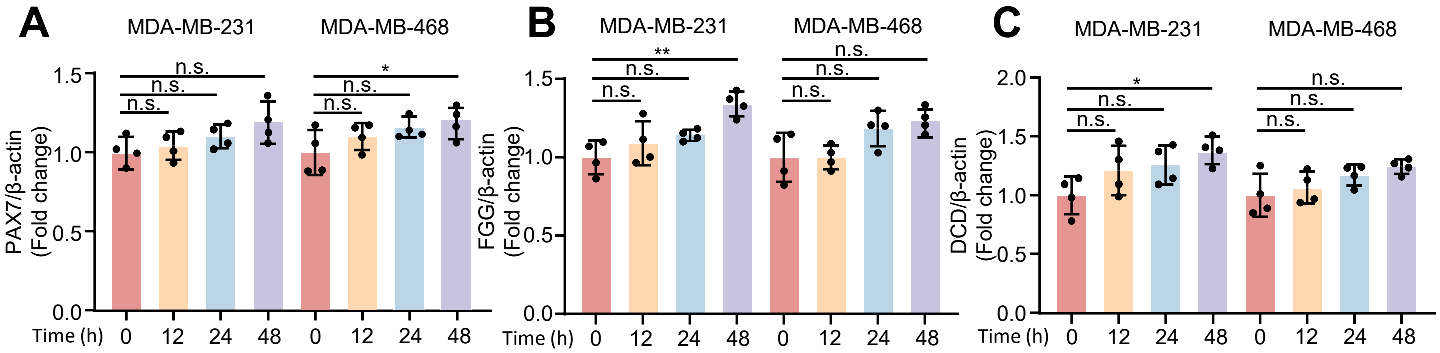


**Supplementary Figure S4. Expression of PAX7, FGG, and DCD in TNBC cells under hypoxic conditions.** qRT-PCR analysis of the mRNA expression of (A) PAX7, (B) FGG, and (C) DCD under hypoxia conditions in MDA-MB-231 and MDA-MB-468 cells. Statistical significance was determined using one-way ANOVA. Data are presented as mean ± SEM ($n = 4$). *: $p < 0.05$, **: $p < 0.01$, n.s. indicates not significant.

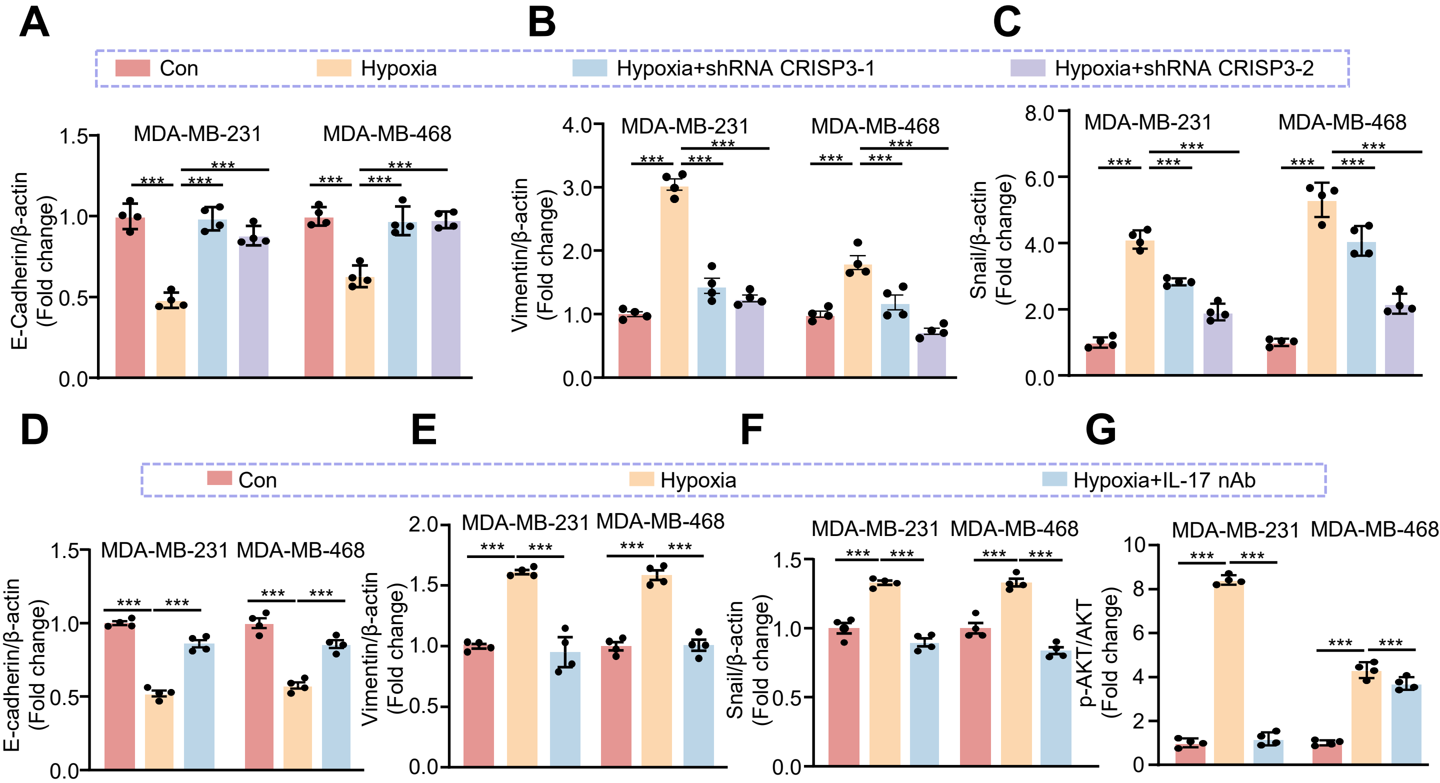


**Supplementary Figure S5. Quantification of EMT marker and p-AKT expression.** Western blot quantification corresponding to Figure 9D. MDA-MB-231 and MDA-MB-468 cells were transduced with pLKO.1-TRC shRNA as control or pLKO.1-CRISP3 shRNA to silence CRISP3 (shRNA CRISP3). **(A-C)** Bar graphs represent the quantifications of the relative protein levels of E-Cadherin, Vimentin, and Snail normalized to β-actin in MDA-MB-231 and MDA-MB-468 cells. Statistical significance was determined using one-way ANOVA. Data are presented as mean ± SEM (n = 4). ***: p < 0.001.

Western blot quantification corresponding to Figure 10F. MDA-MB-231 and MDA-MB-468 cells were exposed to hypoxia with IL-17 neutralizing antibodies (IL-17 nAb). **(D-G)** Bar graphs show the quantifications of the relative protein levels of E-Cadherin, Vimentin, Snail, and p-AKT normalized to β-actin or AKT in MDA-MB-231 and MDA-MB-468 cells. Statistical significance was determined using one-way ANOVA. Data are presented as mean ± SEM (n = 4). ***: p < 0.001.
